# Temporal dynamics of neurogenomic plasticity in response to social interactions in male threespined sticklebacks

**DOI:** 10.1371/journal.pgen.1006840

**Published:** 2017-07-13

**Authors:** Syed Abbas Bukhari, Michael C. Saul, Christopher H. Seward, Huimin Zhang, Miles Bensky, Noelle James, Sihai Dave Zhao, Sriram Chandrasekaran, Lisa Stubbs, Alison M. Bell

**Affiliations:** 1 Carl R. Woese Institute for Genomic Biology, University of Illinois, Urbana Champaign, Urbana, IL, United States of America; 2 Illinois Informatics Institute, University of Illinois, Urbana Champaign, Urbana, IL, United States of America; 3 Program in Ecology, Evolution and Conservation Biology, University of Illinois, Urbana Champaign, Urbana, IL, United States of America; 4 Neuroscience Program, University of Illinois, Urbana Champaign, Urbana, IL, United States of America; 5 Department of Statistics, University of Illinois, Urbana Champaign, Urbana, IL United States of America; 6 Harvard Society of Fellows, Harvard University, Cambridge, MA, United States of America; 7 Faculty of Arts and Sciences, Harvard University, Cambridge, MA, United States of America; 8 Broad Institute of MIT and Harvard, Cambridge, MA, United States of America; 9 Department of Cell and Developmental Biology, University of Illinois, Urbana Champaign, Urbana, IL, United States of America; Queen Mary University of London, UNITED KINGDOM

## Abstract

Animals exhibit dramatic immediate behavioral plasticity in response to social interactions, and brief social interactions can shape the future social landscape. However, the molecular mechanisms contributing to behavioral plasticity are unclear. Here, we show that the genome dynamically responds to social interactions with multiple waves of transcription associated with distinct molecular functions in the brain of male threespined sticklebacks, a species famous for its behavioral repertoire and evolution. Some biological functions (e.g., hormone activity) peaked soon after a brief territorial challenge and then declined, while others (e.g., immune response) peaked hours afterwards. We identify transcription factors that are predicted to coordinate waves of transcription associated with different components of behavioral plasticity. Next, using H3K27Ac as a marker of chromatin accessibility, we show that a brief territorial intrusion was sufficient to cause rapid and dramatic changes in the epigenome. Finally, we integrate the time course brain gene expression data with a transcriptional regulatory network, and link gene expression to changes in chromatin accessibility. This study reveals rapid and dramatic epigenomic plasticity in response to a brief, highly consequential social interaction.

## Author summary

Social interactions provoke changes in the brain and behavior but their underlying molecular mechanisms remain obscure. Male sticklebacks are small fish whose fitness depends on their ability to defend a territory. Here, by measuring the time course of gene expression in response to a territorial challenge in two brain regions, we show that a single brief territorial intrusion provoked waves of gene expression that persisted for hours afterwards, with waves of transcription associated with distinct biological processes. Moreover, a single territorial challenge caused dramatic changes to the epigenome. Changes in chromatin accessibility corresponded to changes in gene expression, and to the activity of transcription factors operating within gene regulatory networks. This study reveals rapid and dramatic epigenomic plasticity in response to a brief, highly consequential social interaction. These results suggest that meaningful social interactions (even brief ones) can provoke waves of transcription and changes to the epigenome which lead to changes in neural functioning, and those changes are a mechanism by which animals update their assessment of their social world.

## Introduction

Animals exhibit remarkable behavioral plasticity. Social interactions in particular can provoke moment-to-moment changes in behavior. These changes are coordinated at the neural level, but social interactions also elicit transcriptional changes within the brains of behaving animals [1]. For example, genome-wide transcription studies show that roughly ~10% of the genome responds to a mating opportunity [2–7], predation risk [8–10], or a territorial challenge [11–13].

However, we know little about the temporal and spatial dynamics of neurogenomic plasticity in response to social interactions. It is likely that there are waves of transcription associated with perceiving social information, responding to social information, maintaining a behavioral response, recovering from the social interaction and modifying future behavior [14, 15]. Static experiments that measure gene expression at a single time point can only catch a glimpse of what is probably a very dynamic and coordinated process.

Studies in development have linked changes in chromatin accessibility with the time course of changes in gene expression and the activity of transcription factors operating within gene regulatory networks [16, 17]. This tactic has also proven to be successful for examining acute, short-term responses of other types, for example, in response to pathogens [18, 19]. However, whether the same principles apply to behavioral stimuli, and social interactions in particular, is unknown.

Here, we test the hypothesis that a brief social interaction, albeit one with strong implications for fitness, is sufficient to induce transcriptomic and epigenomic responses that change over time. We test this hypothesis in threespined sticklebacks (*Gasterosteus aculeatus*), a species for which successful territorial defense is critical for Darwinian fitness. Sticklebacks are small fish whose behavioral repertoire has attracted attention since the early ethologists [20]. Freshwater sticklebacks must quickly establish territories because they have a short window of opportunity to breed in the spring, and die at the end of the breeding season. Male sticklebacks typically occur in neighborhoods and function in a dynamic social environment where they vigorously defend individual nesting territories against intrusions by rival males and predators. The territory is the hub of family life, where the father constructs a nest, attracts females to mate and where he rears the offspring without any help from the mother. If a male fails to defend a territory, he will not obtain a mate and he will not produce offspring therefore effective defense of that territory is necessary for reproductive success. Like other territorial animals, male sticklebacks exhibit experience-dependent changes in behavior following a territorial intrusion, as they learn the boundaries of their territory and how to detect and repel intruders [21, 22].

We provide evidence that the genome and the epigenome are highly responsive to social interactions during territory defense. We characterize transcriptomic and epigenomic plasticity in response to social interactions by measuring changes in gene expression at three points in time following a brief territorial intrusion using RNA-Seq. We compare expression in two parts of the brain containing nodes in the social decision-making network [23]: diencephalon and telencephalon. The diencephalon includes the hypothalamus–a key integrator of social information with the neuroendocrine system–while the telencephalon is a part of the forebrain, and includes the teleost homolog of the hippocampus. Using these data, we construct a transcriptional regulatory network that links temporal changes in gene expression in different parts of the brain to the activity of transcription factors operating within a gene regulatory network. Finally, we measure changes in chromatin accessibility in response to a social interaction on a genome-wide scale, using acetylated lysine 27 on histone H3 (H3K27Ac) as a marker of accessible chromatin, and link changes in chromatin accessibility to changes in gene expression. We show that many of the same principles that characterize transcriptomic and epigenomic changes unfolding over development [16, 17] also apply to the brain’s response to brief, but potent, social behavior.

## Results

### Spatiotemporal dynamics of the transcriptomic response to territorial challenge

**Fig 1:**
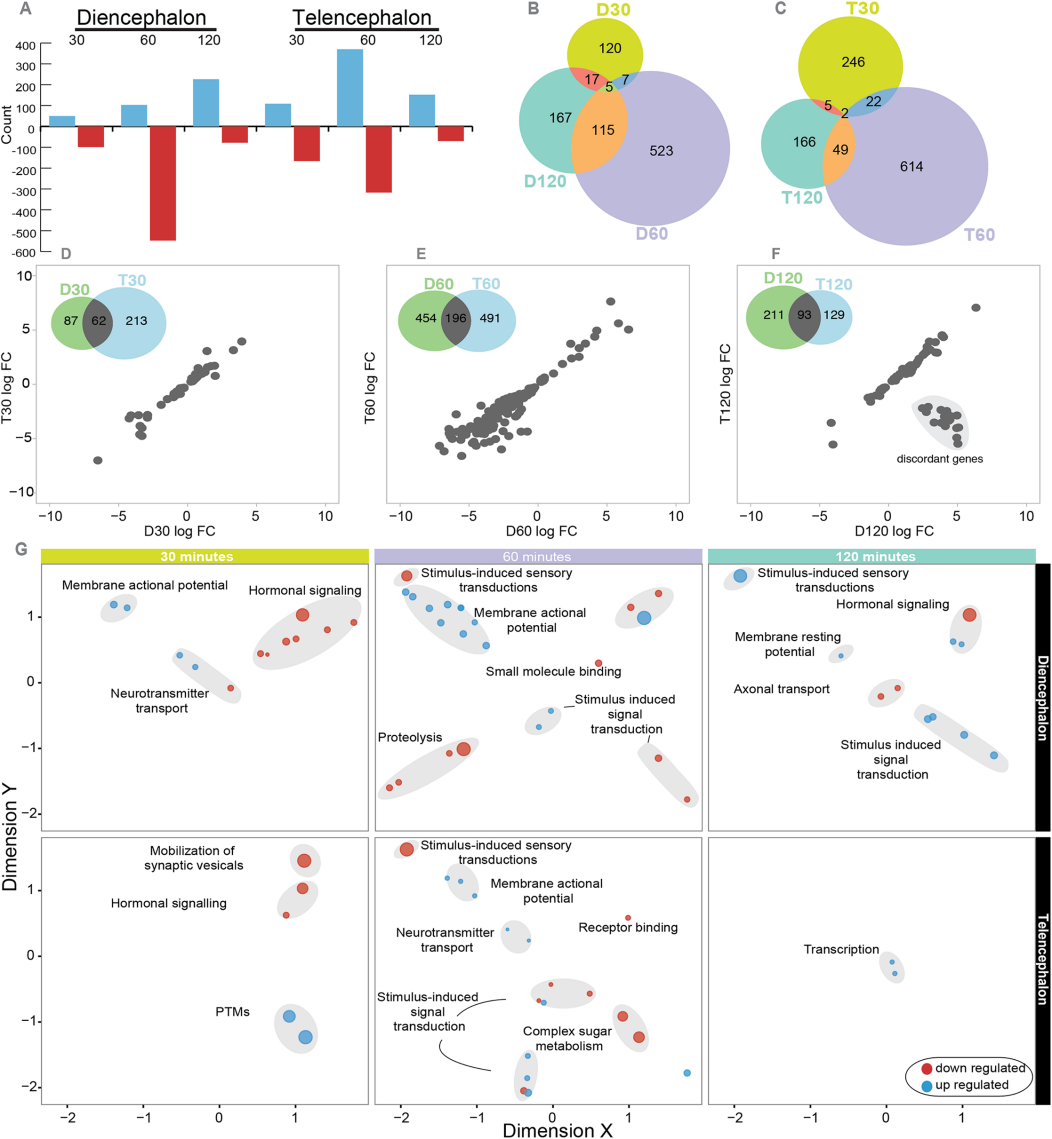
Brain region-specific changes in gene expression in response to a territorial challenge over time. (A) Numbers of up- (blue) and down (red)-regulated genes at 30, 60 and 120 minutes after a territorial challenge in diencephalon and telencephalon. Overlap between differentially expressed genes across time points in diencephalon (B) and telencephalon (C). Correlation between expression in diencephalon (X axis) and telencephalon (Y axis) at 30 min (D), 60 min (E) and 120 min (F) after a territorial challenge. The numbers in the Venn diagram indicate the number of differentially expressed genes in each brain region and the overlap between them at a given time. Scatterplots show the expression pattern of the genes that were shared between brain regions at a time point. Note the cluster of genes in the lower right corner of 1f, hereafter referred to as ‘discordant genes’, which were differentially expressed in both brain regions at 120 minutes but in opposite directions: they were upregulated in diencephalon and downregulated in telencephalon. (G) Functional enrichment of DEGs by time point (columns) and by brain region (rows), shown as revigo-like MDS graphs. Blue indicates enrichment of up-regulated genes, red indicates enrichment of down-regulated genes. Groups of terms with similar functions are highlighted.

Within both brain regions, we identified genes whose expression was influenced by a territorial challenge at three time points: 30, 60 and 120 minutes (S1 Table). The greatest transcriptional response to a territorial challenge occurred 60 minutes after the challenge (Fig 1A). Generally, gene expression was down-regulated 30 and 60 minutes after the social challenge but was up-regulated at the 120 minute time point in diencephalon (Fig 1A).

The transcriptomic response to a territorial challenge changed rapidly over time in both brain regions (Fig 1B and 1C); in fact, there was little overlap between the differentially expressed genes detected at each time point. Functional enrichment analysis revealed that early responding genes (30 minutes) were related to hormones and post-translational modifications (PTMs), whereas a strong signature of genes related to metabolism dominated at the 60 minute time point (consistent with [24]). By 120 minutes, differentially expressed functions shifted toward transcription, immune response and homeostasis (Fig 1G, S2 Table).

Not surprisingly, we detected strong differences in gene expression between brain regions. However, there were some genes that were differentially expressed in both brain regions, and these genes showed a remarkably concordant quantitative pattern of expression across brain regions at 30 and 60 minutes (Fig 1D and 1E). Specifically, genes that were strongly upregulated in diencephalon in response to a territorial challenge were also strongly upregulated in telencephalon (correlation > 0.9). The pattern at 120 minutes was different, with a subset of genes (n = 18, hereafter referred to as ‘discordant genes’, S3 Table) showing the opposite pattern of regulation in the two brain regions. These 18 genes, which were upregulated in diencephalon and downregulated in telencephalon after the territorial challenge (Fig 1F), are primarily related to visual perception and include retinal genes (e.g. *rom1b*, *rom 1a*, opsins), circadian genes (e.g. *crx*, opsins) and phosphodiesterases, which have been repeatedly duplicated in the stickleback genome and acquired new functions [25].

### Waves of transcription in response to a territorial challenge

In order to find genes that changed in a coordinated fashion in response to a territorial challenge, we first analyzed the gene expression data by testing for main effects and interactions between them. We built separate generalized linear models for each brain region and were particularly interested in genes whose time course of expression was influenced by the territorial intrusion (time x treatment interaction term). There were 758 and 739 such genes in diencephalon and telencephalon, respectively, hereafter referred to as DEG_x_ (FDR < 0.1, S4 Table).

**Fig 2:**
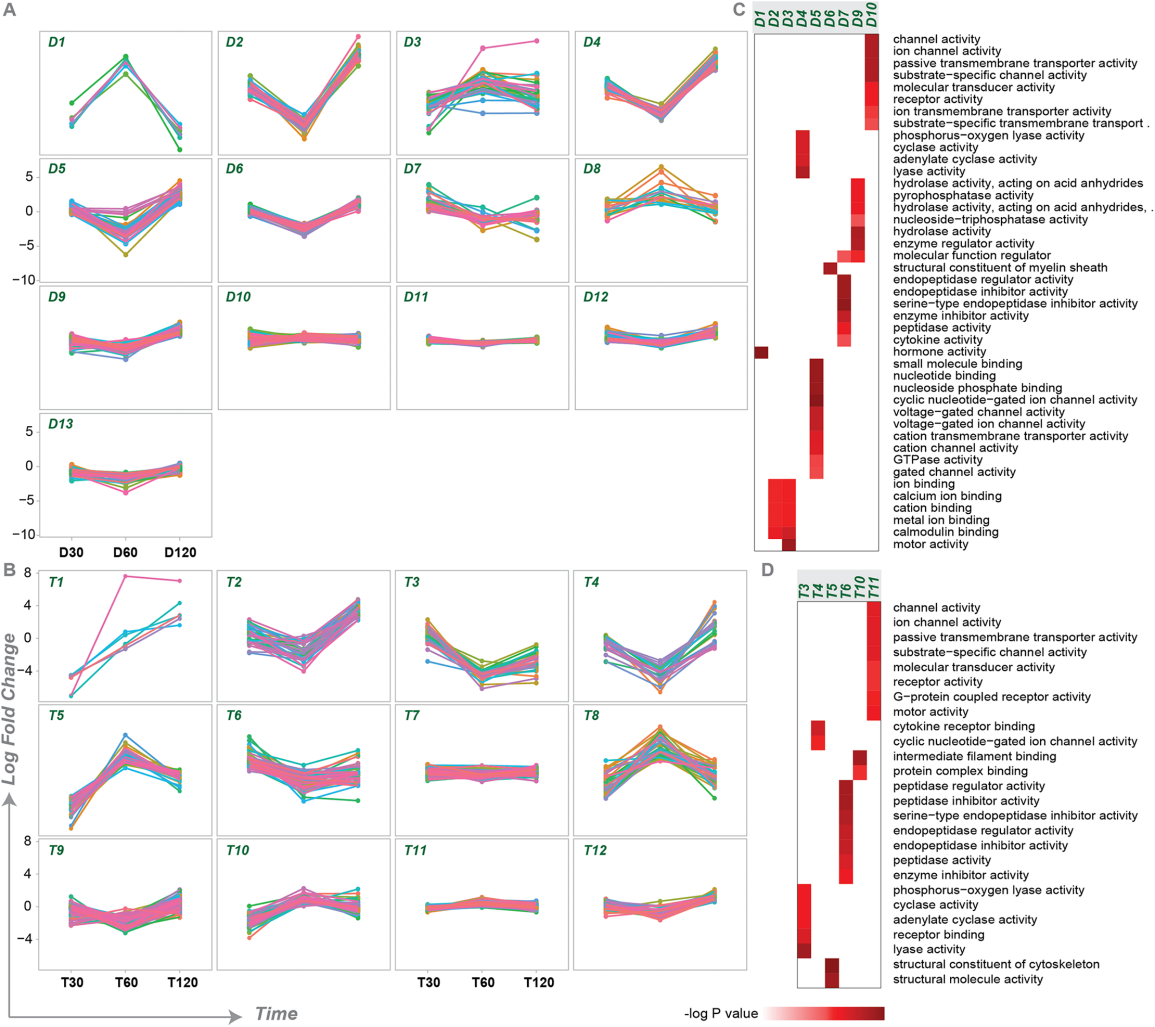
Hierarchical clustering of genes whose expression profiles changed over time in response to a territorial challenge (DEG_x_) and their functional enrichments. Hierarchical clustering grouped together genes with similar expression profiles over time. 13 clusters were identified in diencephalon (D1-D13, A). 12 clusters were identified in telencephalon (T1-T12, B). Each line represents the expression pattern of a different gene, where positive fold change indicates upregulation and negative fold change indicates downregulation in response to a territorial challenge. Clusters of genes with similar expression profiles (columns) had different GO molecular functions associated with them (rows); C) diencephalon; D) telencephalon. Some clusters did not have significant functional enrichment.

We next used hierarchical clustering of the DEG_x_ to determine whether there were clusters of genes that changed in concert together. We identified 12 and 13 clusters in diencephalon and telencephalon, respectively (Fig 2A and 2B, S4 Table). Each cluster had a particular expression profile over time in response to a territorial challenge. For example, cluster D1 comprised a set of genes that were downregulated at 30 mins, upregulated at 60 mins and downregulated again at 120 mins. On the other hand, cluster D2 comprised a set of genes that were upregulated at 30 mins, downregulated at 60 mins and then strongly upregulated at 120 mins.

Functional analysis revealed that genes with similar time-course profiles also tended to have similar functions. For instance, cluster D1 comprised hormonal genes such as *tshb*, *prl*, *cga*, *lhb* and *gh1*, and a nuclear receptor transcription factor *nr5a1b*, which binds to a *prl* (encoding prolactin) promoter [26]. In contrast, cluster T2 included transcription factors such as *pax7* (both *a* and *b* paralogs), *irx (2a*, *3a and 5a)*, *tfap2b*, *shox*, *sp5l*, *DMBX1* and *pou4f2* and two hormonal genes known to be very important to social behavior (*avp* and *oxt*). The clustering of hormonal genes with transcription factors suggests a complex interplay between hormones with transcription factors in response to a territorial challenge. Clusters D2 and T3 included the genes that exhibit the discordant pattern of expression across brain regions at 120 minutes (Fig 1F).

Functional enrichment analysis confirmed that each cluster of genes is associated with its own unique set of functions (Fig 2C and 2D, S5 Table). GO terms enriched in cluster D10, for example, were not shared with any other cluster, while cluster D4 also associated with its own unique set of GO terms. These results are consistent with the hypothesis that there are waves of transcription associated with different biological functions following a social interaction. Some biological functions peak early then subside (e.g., cluster T3), while others peak at 60 minutes (e.g., D1, D8, T5, T8); still others peak hours (120 mins, e.g., D2, D4, T1, T4) following a social interaction.

### Transcription factors within a gene regulatory network coordinate waves of transcription

Next, to identify genes that regulate transcriptional changes in the stickleback brain in response to a territorial challenge, we reconstructed a transcriptional regulatory network (TRN) model using the ASTRIX approach [27]. We used the gene expression data to identify regulatory interactions between transcription factors and their predicted target genes (see methods). ASTRIX infers a genome-scale TRN model capable of making quantitative predictions about the expression levels of genes given the expression values of the transcription factors. The full TRN is in S1 Fig.

**Fig 3:**
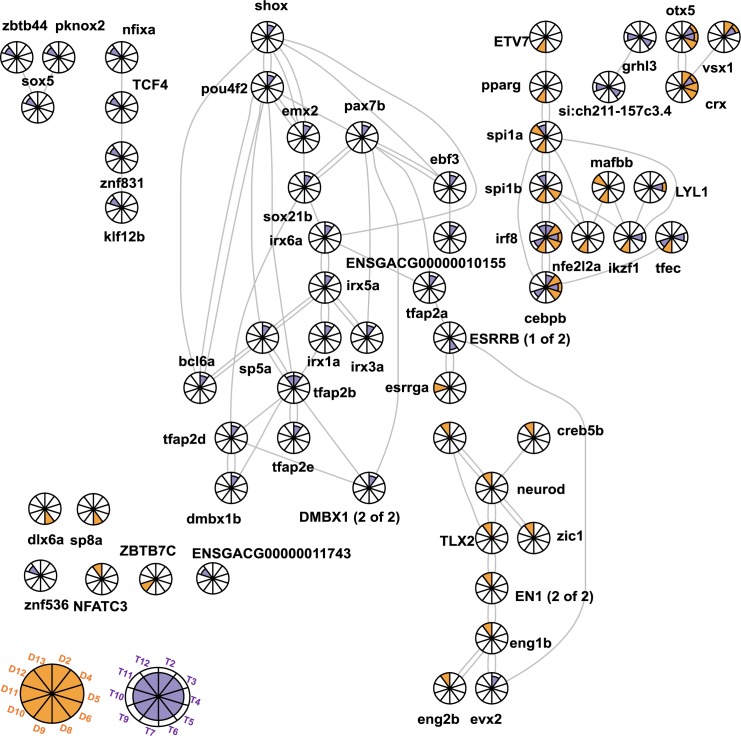
Network of interacting transcription factors (TFs) in the transcriptional regulatory network highlighting enrichments of TFs in clusters of DEG_x_. Each node represents a TF. Slices of pie correspond to different clusters in diencephalon or telencephalon; the key to the clusters is in the lower left corner. A full orange slice represents a diencephalon cluster. A purple half slice represents a telencephalon cluster, a purple and orange slice represents clusters in both brain regions. For example, *cebpb* is predicted to regulate D4, D5, D6, T3, T4, T9.

We then integrated the DEG_x_ from the hierarchical clustering analysis with the TRN in order to find transcription factors that are predicted to regulate the clusters. This integration proved to be insightful because it connected dynamic gene expression to interacting transcription factors within a gene regulatory network. For example, the transcription factors *dlx4a*, *grhl3* and *si*:*ch211-157c3*.*4* were predicted to regulate cluster D9 (enriched for energy metabolism and immune response) and were connected to each other in the network. This analysis therefore allows us to identify transcription factors within a gene regulatory network that we hypothesize are regulating clusters of genes that change in a coordinated fashion in response to a territorial challenge (Fig 3).

Indeed, closer examination of the dynamics of expression of transcription factors and their targets revealed that many of the transcription factors in the TRN showed expression patterns consistent with the cluster they were predicted to regulate. For example, the expression pattern over time of *irf8* and *cebpb* was very similar to the expression pattern of their predicted targets (D4, D5, D6, D9: no change, down, up).

The TRN offered a number of insights into the spatiotemporal dynamics of gene expression in response to a territorial challenge. For example, the TRN can help explain striking patterns in the gene expression results, such as the discordant genes that were upregulated in diencephalon and downregulated in telencephalon 120 minutes after a social challenge (Fig 1F). The discordant genes were in clusters D2 and T3, which were predicted to be regulated by the set of connected transcription factors *otx5*, *vsx1* and *crx*. *Crx*, implicated with circadian rhythm in addition to visual functions [28], is noteworthy because its expression profile was consistent with the expression pattern of the discordant genes: *crx* was upregulated in diencephalon and downregulated in telencephalon at 120 minutes. A full list of the TFs in the TRN that were enriched in the DEG_x_ is in S6 Table.

### Linking changes in gene expression to changes in chromatin accessibility

While chromatin is suspected to change relatively slowly compared to mRNA in adult tissues, changes in chromatin accessibility can be an important driver of changes in gene expression [29]. However, little is known about the impact of short-term behavioral interactions on the chromatin landscape.

To test the hypothesis that a brief social interaction has consequences for the epigenome, we used chromatin immunoprecipitation on histone H3 subunits with acetylated lysine 27 (H3k27Ac ChIP-Seq), a marker of accessible chromatin, to assess changes in genome-wide chromatin accessibility at two time points (30 and 120 minutes) following a territorial challenge in diencephalon. These experiments revealed tens of thousands of H3K27Ac peaks in each sample tested with robust *p* values and enrichment (S7 Table; see Methods for details). We distinguish between areas of the genome that were accessible in controls (‘baseline accessible peaks’) from areas of the genome whose accessibility changed in response to a territorial challenge (i.e. differed between control and experimental males, ‘differentially accessible peaks’, DAPs).

Most of the genes were accessible at baseline (S2 Fig). There were 23656 and 18797 baseline accessible peaks (≥4-fold change in peak difference between sample and input, and p < 10^−4^) associated with 12630 and 11723 genes (within 20kb) at 30 minutes and 120 minutes, respectively (S8 Table).

**Fig 4:**
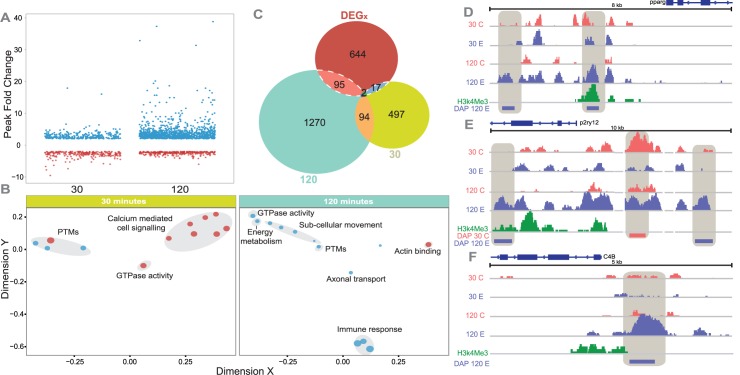
Connecting gene expression and chromatin accessibility in diencephalon. (A) Fold change of differentially accessible peaks at 30 minutes and 120 minutes; blue indicates up in experimental, red indicates down in experimental. (B) Functional enrichment (molecular function) of genes associated with differentially accessible peaks at 30 minutes and 120 minutes. Blue indicates up in challenged, red indicates down in challenged. (C) Overlap of genes whose expression profile changed over time in response to a social interaction (DEG_x_) with genes associated with differentially accessible peaks at 30 minutes and 120 minutes. The overlap between DEG_x_ and accessibility at 120 minutes is statistically significant (P<0.0001). (D-F) Examples of differentially accessible peaks around DEG_x_. Separate tracks are shown for H3K27Ac peaks in control 30 min, experimental 30 min, control 120 min, experimental 120 min, and H3k4Me3, which marks the location of the promoter. (D) *Pparg* (a TF in D9 and also present TRN) was more accessible at 120 minutes and was also up-regulated at 120 minutes. (E) *P2ry12* (cluster D9) is purinergic receptor involved in synaptic plasticity [65] that was more accessible in controls at 30 minutes then become more accessible in experimental animals at 120 minutes. *P2ry12* is known to stimulate microglia migration toward neuronal damage [66]. (F) *C4B* (cluster D9) was not accessible at baseline but became accessible at 120 mins.

However, there were a large number of genes whose accessibility was affected by a territorial challenge, particularly 120 minutes following the challenge (Fig 4A and 4B, S8 Table). There were 2868 differentially accessible peaks (DAPs) that were associated with 1975 genes (within 20kb, DAPs; 2-fold and p < 10^−4^). Representative DAPs are shown in Fig 4D, 4E and 4F. Many (n = 97) of the peaks that were differentially accessible at 120 minutes were near genes whose expression profile changed over time in response to a territorial challenge (DEG_x,_ Fig 4C). The DEG_x_ associated with nearby DAPs (hereafter referred to as DAPDEG_x_) were not a random set, but were enriched for specific functional categories: functions related to stimulus response, cell signaling and development were highly enriched in this gene set (S9 Table).

The territorial challenge had dramatic consequences for chromatin in terms of peak size, with fold enrichment or depletion in specific peaks after challenge as high as 30-40x (Fig 4A). There was a general trend toward more accessibility at the 120 min following a territorial challenge, consistent with the general pattern of up-regulation of DEGs in diencephalon at the 120 min time point (Fig 1A).

Computational analysis suggests a small set of peaks (associated with 24 genes) that were inaccessible in the control group but which became accessible in response to a territorial challenge at 120 minutes, and are possible pioneer factors (S9 Table). A representative sample is in Fig 4F, which shows a differential peak within 5kb upstream of *C4B*. The peaks that were inaccessible at baseline but became accessible in response to a territorial challenge included several that are near genes associated with the immune response, e.g. *irg1*, *lcp1*, *ccr8*.*1*, *pstpip1b*, *PRF1*, *C4B*, zc3h12a.

Table 1 illustrates how changes in chromatin are linked to the activity of transcription factors in the TRN and the expression of their targets over time. All of the transcription factors in Table 1 are in the TRN and the genes encoding these TFs were all associated with DAPs that either became accessible, or became more accessible, in response to a territorial challenge at 120 minutes. All but one of these TFs (*NFATC3*) regulate clusters of genes that are upregulated at 120 minutes (e.g. clusters D9, D12, D6, D12, D5). Several transcription factors (*NFATC3*, *irf8*, *pparg* and *cebpb*) were themselves differentially expressed over time, and their expression tracks the expression of their targets. The overall pattern of chromatin becoming more accessible at 120 minutes suggests that there are transcriptomic consequences of a brief territorial challenge that persist for more than two hours afterwards.

**Table 1:** Integrating TFs with DEG_x_ and chromatin accessibility. These TFs are in the TRN and are enriched in the DAPDEG_x_ with accessibility indicated. Some of the TFs (in bold) were differentially expressed and in a cluster. The general expression pattern of their cluster is indicated. A complete set of enrichments is in S10 Table.

*TF*	Description	Cluster	Expression pattern	Accessibility
***pparg***	peroxisome proliferator activated receptor gamma	D9	Up, down, up	More accessible at 120E
*ikzf1*	IKAROS family zinc finger 1 (Ikaros)			Became accessible at 120E
*ETV7*	ets variant 7			More accessible at 120E
*mafbb*	v-maf musculoaponeurotic fibrosarcoma oncogene family, protein B, duplicate b			Became accessible at 120E
***cebpb***	CCAAT/enhancer binding protein (C/EBP), beta	D9	No change, down, up	More accessible at 120E
*spi1b*	spleen focus forming virus (SFFV) proviral integration oncogene spi1b			Became accessible at 120E
***irf8***	interferon regulatory factor 8	D9	No change, down, up	Became accessible at 120E
***NFATC3***	nuclear factor of activated T-cells, cytoplasmic, calcineurin-dependent 3	D11	Down, down, no change	More accessible at 120E

## Discussion

By integrating different types of transcriptomic and epigenomic data (RNA-Seq, H3K27Ac ChIP-Seq) with rigorous computational analyses, we show heretofore underappreciated consequences of social interactions for the brain transcriptome and epigenome. We detected dramatic changes in gene expression over time in response to a brief territorial challenge: clusters of genes enriched for particular biological functions changed in a coordinated fashion, over a period extending for hours afterwards. Using a TRN and generalized linear model, we linked changes in gene expression to the activity of transcription factors, which we propose to be factors that regulate them. Moreover, we demonstrate that a brief social interaction was sufficient to cause changes in the accessibility of chromatin elements throughout the genome, including many linked to DEGs. While conventional wisdom is that chromatin changes relatively slowly in adult tissues, there is some precedent for our findings of rapid response in adult brain; for example, epigenetic responses to strong stimuli such as cocaine can happen quickly, e.g. within an hour [30]. Indeed, there is growing evidence from the learning and memory literature that chromatin can be very dynamic [31–34], and changes in chromatin accessibility in response to a social challenge have been reported in other species [35]. The magnitude of epigenomic plasticity we observed in response to a territorial challenge is also noteworthy. Hundreds of genes were closely linked to differentially accessible chromatin peaks, and for many of these we found dramatic differences in the degree of accessibility, measured by enrichment for H3K27Ac, following a social interaction (Fig 4B). Indeed, a territorial challenge was sufficient to cause some genes that were not clearly associated with accessible chromatin prior to a territorial challenge to become accessible afterwards (S10 Table). We hypothesize that a territorial intrusion provoked dramatic responses at the transcriptomic and epigenomic level in male sticklebacks because successful territory defense is so consequential in this species, with strong implications for fitness.

Changes in gene expression over time were similarly dramatic, consistent with the hypothesis that there are waves of transcription associated with different temporal aspects of behavioral plasticity–some genes are involved in the initial reaction to a conspecific, others with responding to social information and still others involved in recovery and preparing for the future. The early hormonal response parallels the time course of the neuroendocrine response to aggression, which involves both the hypothalamic-pituitary-adrenal (interrenal in fishes) axis and the hypothalamic-pituitary-gonadal axis in vertebrates [36], including in sticklebacks [37]. Interestingly, prolactin–a hormone associated with maternal care–was also recruited in response to a territorial challenge. This result is consistent with the hypothesis that aggression and parental care share common neuroendocrine and neurogenomic substrates [36]. The relatively large number of upregulated DEG and increased chromatin accessibility at the 120 minute time point implies that much of the neurogenomic response to a brief territorial intrusion is related to recovery and preparing for the future, rather than producing the immediate behavioral response [38]. The increased accessibility and expression of genes related to immunity and learning at 120 minutes is consistent with this idea. For example, GO terms related to learning (calmodulin binding and calcium ion binding, involved in the activation of CamK) were enriched in clusters of genes that peak at 120 mins (D2 and D3). Similarly, the expression of *CAMKK1* (important for long term memory [39], for example, changed over time in response to a territorial challenge and was upregulated at 120 minutes. Finally, actin binding, important for late long term potentiation and long term memory [40], was implicated in the differentially accessible genes (Fig 4C). Indeed, there is growing appreciation for the relationship between immunity and learning, especially spatial learning [41, 42]. Increased chromatin accessibility at 120 minutes is also consistent with the idea that the transcriptomic response to social interactions might be even faster in the future, i.e. priming.

The involvement of learning and memory-related genes makes sense in light of the biology of territorial animals [43]. During an intrusion, territory holders gain information about the spatial boundaries of their territory, the competitive ability of their neighbors and their own resource holding potential, and use that information to guide future behavior. Indeed, territorial animals improve their ability to detect and repel intruders with experience [44–47] and the behavioral literature is rife with examples of experience-dependent changes following a territorial challenge such as the prior residency advantage [48], the winner effect [49] and the dear enemy phenomenon [50]. Social interactions during territory defense are especially likely to influence spatial learning. For example, fishes actively patrol sites where they’ve had previous encounters with intruders [51, 52]. We speculate that a brief territorial challenge triggers the expression of learning-related genes and that changes in chromatin are associated with the formation of memories of where the social interaction occurred.

A growing number of studies are implicating metabolic genes with aggression [24, 53–55]. Consistent with this, the expression and accessibility of peroxisome proliferator activated receptors gamma (*pparg*), which participates in the regulation of lipid metabolism and glucose homeostasis, changed over time in response to a territorial challenge. *Cebpb* is another transcription factor enriched in the DAPDEG_x_ which directly binds at the *pparg* promoter and can regulate its expression [56]. Other studies have shown that *pparg* is expressed in the hypothalamus and is important for CNS energy balance [57, 58]. For instance, *pparg* agonists, which are insulin-sensitization drugs such as thiazolidinedione (TZD), are widely prescribed to diabetes mellitus 2 patients [57]. *Pparg* and its targets are downregulated at 60 min and then up-regulated at 120 minutes, possibly reflecting changes in energy balance and homeostasis following an aggressive interaction.

From an ethological perspective it is staggering to consider these results in light of the richness of social animals’ lives. Animals that live in social groups are constantly engaged in social interactions. Indeed, rates of territorial intrusions in natural populations of sticklebacks have been reported to be as high as 76 intrusions per hour [59]. Moreover, territory holders interact not only with competitors but also with predators, potential mates and offspring. How animals in natural populations behave during these interactions influences their current and future social environment as well as their fitness. Our results prompt the intriguing hypothesis that meaningful social interactions (even brief ones) can provoke waves of transcription and changes to the epigenome which lead to changes in neural functioning, and those changes are a mechanism by which animals update their assessment of their social world.

## Methods

### Animals

Adult males were collected from Putah Creek, a freshwater population, in spring 2013 and maintained in the lab on a 16:8 (L:D) photoperiod and at 18°C in separate 9-liter tanks. Males were provided with nesting material including algae, sand and gravel and were visually isolated from neighbors. All males were in the ‘territorial’ phase of the nesting cycle, i.e. defending a territory. Sneaking is rare in this population.

### Territorial challenge

We employed a method to simulate a territorial challenge initially developed by van Iersel [60] and used in previous studies [13, 24]. Males were randomly assigned to either the experimental or control group. Males in the experimental group were presented with a smaller, unrelated male intruder confined to a flask. Males in the control groups were presented with an empty flask. At the same time as a confined intruder was introduced to an experimental male’s tank, an empty flask was introduced into a paired control male’s tank. After 5 min the flask was removed, and after a predetermined period (see below) males were quickly netted and sacrificed by decapitation within seconds following an IACUC approved protocol (#15077) of the University of Illinois at Urbana-Champaign.

### RNA sequencing

#### Tissue preparation

Males for RNA Sequencing were collected 30, 60 or 120 minutes after the flask was introduced, with n = 10 males per time point. Heads were flash frozen in liquid nitrogen and the telencephalon and diencephalon were carefully dissected and placed individually in Eppendorf tubes containing 500 μL of TRIzol Reagent (Life Technologies). Total RNA was isolated immediately using TRIzol Reagent according to the manufacturer’s recommendation and subsequently purified on columns with the RNeasy kit (QIAGEN). RNA was eluted in a total volume of 30 μL in RNase-free water. Samples were treated with DNase (QIAGEN) to remove genomic DNA during the extraction procedure. RNA quantity was assessed using a Nanodrop spectrophotometer (Thermo Scientific), and RNA quality was assessed using the Agilent Bioanalyzer 2100 (RIN 7.5–10). RNA was immediately stored at −80°C until used in sequencing library preparation.

#### Library preparation

Poly-A RNA was enriched from 1–2 μg of total RNA by using Dynabeads Oligo(dT)25 (Life Technologies), following the manufacturer’s protocol. Two rounds of poly(A) enrichment were performed with a final elution in 14μL of water. The poly-A–enriched RNA was used to prepare RNA-Seq libraries, using the NEXTflex Directional RNA-seq Kit (dUTP based) with Illumina compatible adaptors (Bioo Scientific). Manufacturer’s instructions were followed and 13–15 cycles of PCR amplification were performed depending on the starting input of total RNA. Libraries were quantified on a Qubit fluorometer, using the dsDNA High Sensitivity Assay Kit (Life Technologies), and library size was assessed on a Bioanalyzer High Sensitivity DNA chip (Agilent). Libraries were pooled and diluted to a final concentration of 10 nM. Final library pools were quantified using real-time PCR, using the Illumina compatible kit and standards (KAPA) by the W. M. Keck Center for Comparative and Functional Genomics at the Roy J. Carver Biotechnology Center (University of Illinois). Single-end sequencing was performed on an Illumina HiSeq 2500 instrument by the W. M. Keck Center for Comparative and Functional Genomics at the Roy J. Carver Biotechnology Center (University of Illinois). The samples were sequenced on 20 lanes.

### ChIP sequencing

#### Tissue preparation

Diencephalons from a new set of males were collected for ChIP-seq at 30 or 120 minutes after the flask was introduced. Prior to nuclei isolation, brain tissue was pooled into groups of 5 and kept at 0°C in PBS with Protease Inhibitor Cocktail (PIC, Roche 04693132001). Tissue was homogenized by motor pestle and then fixed in PBS+PIC with 1% formaldehyde for 10 minutes. The fixing reaction was stopped with addition of Glycine to a final concentration of 0.125M. Fixed cells were washed 2x with PBS+PIC to remove formaldehyde. Washed cells were lysed to nuclei with L1 lysis solution– 50 mM Tris-HCl (pH 8.0), 2 mM EDTA, 0.1% v/v NP-40, 10% v/v glycerol, and protease inhibitor cocktail (PIC)–for 30 minutes on ice. Cell debris was washed away with PBS and PIC. Nuclei were then pelleted and frozen on dry ice. Prior to pelleting, a small (2 μL) sample of nuclei was taken, stained with Trypan, and checked for quality and quantification via hemacytometer. Nuclei were sonicated at high power for 7 x 7 minute cycles (30 s on, 30 s off) in a Diagenode Biorupter Sonicator (Diagenode). Remaining cellular debris was pelleted by centrifugation for 10 minutes at 13,000 x g.

Fragmented chromatin was processed in duplicate for histone H3K27Ac ChIP with Diagenode iDeal ChIP kits, according to manufacturer’s specifications with minor adjustments. Six million nuclei were used for each IP. 25 μl of each IP was reserved for input samples. Technical replicate inputs were pooled to 50 μl. 2 g of H3K27Ac antibody (Abcam ab4729) was used for each IP. An additional wash in TE buffer was performed after the initial four IP washes.

#### Library preparation

After ChIP, IP DNA was quantified by Qubit with a dsDNA High Sensitivity quantification kit (Invitrogen). Libraries were prepared using KAPA LTP library kits, with protocol as written, using Bioo index adapters. Libraries were size selected using AmpureXP beads, with protocol as written, selecting for DNA between 200-500bp in size. Library quality was checked by a Qubit flourometer and Bioanalyzer. Samples were sequenced with an Illumina HiSeq 2500 sequencer.

### RNA-Seq informatics

FASTQC (http://www.bioinformatics.babraham.ac.uk/projects/fastqc/) was used to assess the quality of the reads. Adaptor sequences and low quality bases were clipped from 100 bp single-end sequences using Trimmomatic. RNA-seq produced an average of ~59 million reads per sample. We aligned reads to the *Gasterosteus aculeatus* reference genome (the repeat masked reference genome, Ensembl release 75), using TopHat (2.0.8) and Bowtie (2.1.0) [61]. Reads were assigned to features according to the Ensembl release 75 gene annotation file (http://ftp.ensembl.org/pub/release-75/gtf/gasterosteus_aculeatus/)).

### ChIP-Seq informatics

Libraries from each technical replicate and the input control were sequenced with average depth of 7.6 M reads with average quality score > 35. Technical replicates were pooled with average sequence depth of 16M reads; numbers and QC scores for reads and peaks are summarized for each sample and pools in S11 Table. Sequence data were mapped with Bowtie2 [61] to the *Gasterosteus aculeatus* reference genome (the repeat masked reference genome, Ensembl release 75), using default settings, yielding 3.87–7.96 uniquely mapped reads (averaging approximately 1X whole genome coverage of the stickleback genome for each replicate). Mapped sequence data were analyzed for peaks using HOMER (Hypergeometric Optimization of Motif EnRichment) v4.7 [62]. Samples were converted into tag directories, and QC was performed using read mapping and GC bias statistics. Histone peaks were then called from the Tag Directories with default factor settings, except local filtering was disabled (-L 0) and input filtering was set at three-fold over background (-F 3), to increase the sensitivity of the peak calling and identify individual subunits of multi-histone peaks, identifying tens of thousands peaks for each sample with average tag counts ranging from 42.7–58.1. Replicates were assessed for correlation, displaying >80% correlation in these filtered peaks across the two samples, which were then pooled for final peak identification. Peaks were highly associated with annotated gene promoters with average distance to transcription start sites (TSS) ranging from 75–328.4 bp (S11 Table), as expected for H3K27Ac [62]; these data confirmed the robustness of the ChIP-Seq data. After peak calling, peak files were annotated to the stickleback genome using HOMER’s annotation script to assign peaks to genes, and associate peaks with differential expression data. BigWiggle pileup files were generated using HOMER’s makeBigWig.pl script with default settings.

### Defining differentially expressed genes (DEGs)

HTSeq read counts were generated for genes using stickleback genome annotation. Any reads that fell in multiple genes were excluded from the analysis. We included genes with at least one counts per million (cpm) in at least two samples. Count data were TMM (trimmed mean of M-values) normalized in R using edgeR. To assess differential expression a nested interaction model (~time+treatment:time) was fitted separately for diencephalon and telencephalon in edgeR (see edgeR manual section 3.3.2). A tagwise dispersion estimate was used after computing common and trended dispersions. Finally, to call differential expression between treatment groups, a ‘glm’ approach was used. We FDR-adjusted the p-values from all contrasts at once. A FDR cutoff < 0.1 was used to call for differentially expressed genes.

### Hierarchical clustering analysis

An agglomerative clustering was done separately on DEG_x_ from each brain region. A hierarchical dendogram was generated using hclust function in R (R version 3.2.2), whereas “ward.D” objective criterion was used to merge the pair of cluster at each step. Trees were cut at height 25 to obtain clusters. Each cluster’s fold change values at each time point were plotted as profile plots using ggplot2 in R.

### Defining differentially accessible peaks (DAPs)

H3K27Ac peaks and their differences between experimental and control groups were calculated at 30 minutes and 120 minutes in each brain region using HOMER’s getDifferentialPeaks functionality. For each time point and brain region, two sets of results were calculated: one treating the experimental group as background and the other treating the control group as background. An H3K27ac peak was termed to be “differentially accessible” if it had a fold change of larger than 2 in either set of results, and if it had a p-value less than 10^−4^. Differential peak sets were then annotated using a custom R script to search for all transcripts with transcript start or end sites within 20 kb on all Ensembl-annotated splice variants built using biomaRt.

### Associating DAPs with genes

A chromatin domain was defined for each gene in the Ensembl build (v.1.75) of the stickleback genome. First, for each transcript corresponding to a gene, a window was defined that began 20 kb upstream of the transcription start site and ended 20 kb downstream of the transcription end site. A 20 kb window was chosen based on the estimated intergene interval in the stickleback genome. Next, this window was truncated so that it did not intersect with any transcript of any other gene. The union of these windows for all transcripts of a gene constituted that gene’s domain. All peaks that had any overlap with the domain of a gene were considered as potential regulators of that gene’s expression.

### Transcriptional regulatory network (TRN) analysis

ASTRIX uses gene expression data to identify regulatory interactions between transcription factors and their target genes. A previous study validated ASTRIX-generated TF-target associations using data from ModENCODE, REDfly and DROID databases [27]. The predicted targets of TFs were defined as those genes that share very high mutual information (P < 10^−6^) with a TF, and can be predicted quantitatively with high accuracy (Root Mean Square Deviation (RMSD) < 0.33 i.e prediction error less than 1/3rd of each gene expression profile’s standard deviation. The list of putative TFs in the stickleback genome was obtained from the Animal Transcription Factor Database. Given TFs and targets sets, ASTRIX infers a genome-scale TRN model capable of making quantitative predictions about the expression levels of genes given the expression values of the transcription factors. The ASTRIX algorithm was previously used to infer a TRN model for the honeybee brain that showed remarkably high accuracy in predicting behavior-specific gene expression changes. ASTRIX identified transcription factors that are central actors in regulating aggression, maturation and foraging behaviors in the honey bee brain [27]. Transcription factors that are predicted to regulate a cluster (from the hierarchical clustering analysis) were determined according to whether they had a significant number of targets in a cluster as assessed by a Bonferroni FDR-corrected hypergeometric test. TFs with at least 3 targets were used (S12 Table) and a FDR cutoff of < 0.05 was used to call for significant associations.

### Functional analysis

We derived GO assignments, using protein family annotations from the database PANTHER [63]. Stickleback protein sequences were blasted against all genomes in the database (PANTHER 9.0 ∼85 genomes). This procedure assigns proteins to PANTHER families on the basis of structural information as well as phylogenetic information. Genes were then annotated using GO information derived from the ∼82 sequenced genomes in the PANTHER database.

GO analysis were performed in R using TopGo v.2.16.0 and Fisher's exact test. A p-value cut off of <0.01 was used to select for significantly enriched functional terms wherever possible. For visualization we found dissimilarity among GO terms using zebrafish as closest organism and “Wang” algorithm in GOSemSim package [64]. We then plotted their similarity using the non-metric isoMDS function in MASS. We used the individual terms and the genes inside each term to manually annotate names for clusters appearing in MDS plots. This study has been submitted to GEO. The RNA-Seq data and ChIP-Seq data are accessible with this GEO ID: GSE96673.

## Supporting information

S1 Fig TRN highlighting top hubs in the network and region-specific DEGs. The TRN contains 352 TFs, which regulate 1155 genes through a total of 3683 interactions. The top 20 TFs (“hubs”) with the highest number of targets (over 30 each) are highlighted in pink. Target genes that are differentially expressed in Diencephalon or Telencephalon (CFDR < 0.1) are shown as blue or green nodes respectively. Genes in the TRN that were differentially expressed in both the regions are shown in orange.
(DOCX)

S2 Fig Baseline accessibility of diencephalon DEGx at 30 and 120 minutes. (DOCX)

S1 Table Differentially expressed genes between control and experimental identified using pairwise contrasts at each time point within each brain region with separate tabs for each time point (30, 60, 120) within a brain region (D, T). (XLSX)

S2 Table Functional gene ontology enrichment of genes in S1 Table, with separate tabs for biological processes and molecular functions. GO terms were further summarized according to their biological significance in the ‘comments’ column.
(XLSX)

S3 Table Shared genes across brain regions, with separate tabs for each time point. Discordant genes at 120 min are in red.
(XLSX)

S4 Table Differentially expressed genes whose expression profile changed over time in response to a territorial challenge (DEG_x_), and their associated cluster with separate tabs for each brain region. (XLSX)

S5 Table Functional enrichment of DEG_x_ (biological processes) with separate tabs for each brain region. GO terms were further summarized according to their biological significance in the ‘comments’ column.
(XLSX)

S6 Table TRN TFs enrichments in clusters. Numbers indicate p-values.
(XLSX)

S7 Table H3K27Ac peaks–Separate tabs include HOMER assigned peaks for each replicate compared to its corresponding input sample. (XLSX)

S8 Table Chromatin accessibility–Baseline and DAPs. There are separate tabs for each individual replicate compared to corresponding input, DAPs at 30 and 120 minutes, and for baseline peaks at 30 and 120 minutes.
(XLSX)

S9 Table Functional enrichment of DAPs with separate columns for DAPDEG_x_ and genes near peaks that were not accessible at baseline but became accessible in response to a territorial challenge (!AccDAPDEG_x_). There are separate tabs for biological processes and molecular function.
(XLSX)

S10 Table Integrating TFs with DEG_x_ and chromatin accessibility. These TFs are in the TRN and are enriched in the DAPDEG_x_ with accessibility indicated. Some of the TFs (in bold) were differentially expressed and in a cluster. The general expression pattern of their cluster is indicated. A subset of this table is in Table 1.
(DOCX)

S11 Table H3K27Ac ChIP-Seq samples read mapping and quality control information. (XLSX)

S12 Table Transcription factors and their targets as predicted by ASTRIX generated TRN. (XLSX)

S1 Data RNA-Seq quality control. This directory contains six subdirectories. The subdirectory “correlation” contains correlation heatmaps among all samples in diencephalon and telencephalon respectively. The subdirectory “mapping_stat” contains read mapping information on genomic features for each sample. The subdirectory “MDS” shows three dimensional MDS plots of the samples. The subdirectory “ReadDuplication” contains read duplication distributions for each sample. The subdirectory “ReadQuality” contains reads quality information for each sample plotted as both boxplots and heatmaps. The subdirectory “RPKMSaturation” contains information about read depth saturation for each sample as assessed by RPKM resamplings. All transcripts were divided into four quantiles based on their expression and a relative difference of observed and real RPKM values are plotted for each sample.
(ZIP)

## References

[1] Burmeister SS, Jarvis ED, Fernald RD. Rapid behavioral and genomic responses to social opportunity. PLoS Biol. 2005;3:e363 doi: 10.1371/journal.pbio.0030363 16216088 10.1371/journal.pbio.0030363 PMC1255743

[2] McGraw LA, Clark AG, Wolfner MF. Post-mating gene expression profiles of female *Drosophila melanogaster* in response to time and to four male accessory gland proteins. Genetics. 2008;179: 1395–1408. doi: 10.1534/genetics.108.086934 18562649 10.1534/genetics.108.086934 PMC2475742

[3] Mack PD, Kapelnikov A, Heifetz Y, Bender M. Mating-responsive genes in reproductive tissues of female *Drosophila melanogaster*. Proc Natl Acad Sci USA. 2006;103(27):10358–63. doi: 10.1073/pnas.0604046103 16798875 10.1073/pnas.0604046103 PMC1502462

[4] Lawniczak MKN, Begun DJ. A genome-wide analysis of courting and mating responses in *Drosophila melanogaster* females. Genome. 2004;47(5):900–10. doi: 10.1139/g04-050 15499404 10.1139/g04-050

[5] Cummings ME, Larkins-Ford J, Reilly CRL, Wong RY, Ramsey M, Hofmann HA. Sexual and social stimuli elicit rapid and contrasting genomic responses. Proc R Soc Lond B Biol Sci. 2008;275:393–402. 10.1098/rspb.2007.1454 PMC2212751 18055387

[6] Carney GE. A rapid genome-wide response to *Drosophila melanogaster* social interactions. BMC Genomics. 2007;8:288 doi: 10.1186/1471-2164-8-288. 17714588 10.1186/1471-2164-8-288 PMC1999498

[7] Fraser BA, Janowitz I, Thairu M, Travis J, Hughes KA. 2014 Phenotypic and genomic plasticity of alternative male reproductive tactics in sailfin mollies Proc R Soc Lond B Biol Sci. 281: 20132310. 10.1098/rspb.2013.2310 PMC3953829 24573842

[8] Sanogo YO, Hankison S, Band M, Obregon A, Bell AM. Brain transcriptomic response of threespine sticklebacks to cues of a predator. Brain Behav Evol. 2011;77(4):270–85. doi: 10.1159/000328221 21677424 10.1159/000328221 PMC3182040

[9] Lavergne SG, McGowan PO, Krebs CJ, Boonstra R. Impact of high predation risk on genome-wide hippocampal gene expression in snowshoe hares. Oecologia. 2014;176(3):613–24. doi: 10.1007/s00442-014-3053-0 25234370 10.1007/s00442-014-3053-0

[10] Jansen M, Vergauwen L, Vandenbrouck T, Knapen D, Dom N, Spanier KI, et al Gene expression profiling of three different stressors in the water flea *Daphnia magna*. Ecotoxicology. 2013;22(5):900–14. doi: 10.1007/s10646-013-1072-y 23564370 10.1007/s10646-013-1072-y

[11] Alaux C, Sinha S, Hasadsri L, Hunt GJ, Guzman-Novoa E, DeGrandi-Hoffman G, et al Honey bee aggression supports a link between gene regulation and behavioral evolution. Proc Natl Acad Sci U S A. 2009;106(36):15400–5. doi: 10.1073/pnas.0907043106 19706434 10.1073/pnas.0907043106 PMC2730357

[12] Rittschof CC, Robinson GE. Manipulation of colony environment modulates honey bee aggression and brain gene expression. Genes Brain and Behav. 2013;12(8):802–11. doi: 10.1111/gbb.12087 24034579 10.1111/gbb.12087 PMC3863782

[13] Sanogo YO, Band M.A., Blatti C., Sinha S. & Bell A.M. Transcriptional regulation of brain gene expression in response to a territorial intrusion. Proc R Soc Lond B Biol Sci 2012;279:4929–38. 10.1098/rspb.2012.2087 PMC3497248 23097509

[14] Aubin-Horth N, Renn SC. Genomic reaction norms: using integrative biology to understand molecular mechanisms of phenotypic plasticity. Mol Ecol. 2009;18(18):3763–80. doi: 10.1111/j.1365-294X.2009.04313.x 19732339 10.1111/j.1365-294X.2009.04313.x

[15] Bell AM, Aubin-Horth N. What whole genome expression data can tell us about the ecology and evolution of personality in animals. Philos Trans R Soc Lond B Biol Sci. 2010; 365:4001–4012. doi: 10.1098/rstb.2010.0185 21078652 10.1098/rstb.2010.0185 PMC2992745

[16] Arbeitman MN, Furlong EEM, Imam F, Johnson E, Null BH, Baker BS, et al Gene expression during the life cycle of *Drosophila melanogaster*. Science. 2002;297:2270–5. doi: 10.1126/science.1072152 12351791 10.1126/science.1072152

[17] Bar-Joseph Z, Gitter A, Simon I. Studying and modelling dynamic biological processes using time-series gene expression data. Nat Rev Genet. 2012;13:552–64. doi: 10.1038/nrg3244 22805708 10.1038/nrg3244

[18] Amit I, Garber M, Chevrier N, Leite AP, Donner Y, Eisenhaure T, et al Unbiased reconstruction of a mammalian transcriptional network mediating pathogen responses. Science. 2009;326:257–63. doi: 10.1126/science.1179050 19729616 10.1126/science.1179050 PMC2879337

[19] Huang Y, Zaas AK, Rao A, Dobigeon N, Woolf PJ, Veldman T, et al Temporal dynamics of host molecular responses differentiate symptomatic and asymptomatic influenza A infection. PLoS Genetics. 2011;7:e1002234 doi: 10.1371/journal.pgen.1002234 21901105 10.1371/journal.pgen.1002234 PMC3161909

[20] Pelkwijk JJ, Tinbergen N. Eine reizbiologische Analyse einiger Verhaltensweisen von *Gasterosteus aculeatus* L. Zeitschrift für Tierpsychologie. 1937;1:193–200. doi: 10.1111/j.1439-0310.1937.tb01422.x

[21] Peeke HVS. Habituation of conspecific aggression in the three-spined stickleback (*Gasterosteus aculeatus L*.). Behaviour. 1969;35:137–56. doi: 10.1163/156853970X00178

[22] Peeke HVS, Veno A. Stimulus specificity of habituated aggression in the stickleback (*Gasterosteus aculeatus*). Behavioral Biology1973. p. 427–32. 10.1016/s0091-6773(73)80083-5 4735876

[23] O’Connell LA, Hofmann HA. Evolution of a vertebrate social decision-making network. Science. 2012;336(6085):1154–7. doi: 10.1126/science.1218889 22654056 10.1126/science.1218889

[24] Rittschof CC, Bukhari SA, Sloofman LG, Troy JM, Caetano-Anolles D, Cash-Ahmed A, et al Neuromolecular responses to social challenge: common mechanisms across mouse, stickleback fish, and honey bee. Proc Natl Acad Sci U S A. 2014;111(50):17929–34. doi: 10.1073/pnas.1420369111 25453090 10.1073/pnas.1420369111 PMC4273386

[25] Sato Y, Hashiguchi Y, Nishida M. Evolution of multiple phosphodiesterase isoforms in stickleback involved in cAMP signal transduction pathway. BMC Syst Biol. 2009;3:23 doi: 10.1186/1752-0509-3-23 19232106 10.1186/1752-0509-3-23 PMC2653465

[26] Hu Z, Zhuang L, Guan X, Meng J, Dufau ML. Steroidogenic factor-1 is an essential transcriptional activator for gonad-specific expression of promoter I of the rat prolactin receptor gene. J Biol Chem. 1997;272:14263–71. 9162060 10.1074/jbc.272.22.14263

[27] Chandrasekaran S, Ament SA, Eddy JA, Rodriguez-Zas SL, Schatz BR, Price ND, et al Behavior-specific changes in transcriptional modules lead to distinct and predictable neurogenomic states. Proc Natl Acad Sci U S A. 2011;108:18020–5. doi: 10.1073/pnas.1114093108 21960440 10.1073/pnas.1114093108 PMC3207651

[28] Furukawa T, Morrow EM, Li T, Davis FC, Cepko CL. Retinopathy and attenuated circadian entrainment in Crx-deficient mice. Nat Genet. 1999;23(4):466–70. doi: 10.1038/70591 10581037 10.1038/70591

[29] Hon G, Wang W, Ren B. Discovery and annotation of functional chromatin signatures in the human genome. PLoS Comput Biol. 2009;5(11):e1000566 doi: 10.1371/journal.pcbi.1000566 19918365 10.1371/journal.pcbi.1000566 PMC2775352

[30] Maze I, Feng J, Wilkinson MB, Sun H, Shen L, Nestler EJ. Cocaine dynamically regulates heterochromatin and repetitive element unsilencing in nucleus accumbens. Proc Natl Acad Sci U S A. 2011;108:3035–40. doi: 10.1073/pnas.1015483108 21300862 10.1073/pnas.1015483108 PMC3041122

[31] Yang Y, Yamada T, Hill KK, Hemberg M, Reddy NC, Cho HY, et al Chromatin remodeling inactivates activity genes and regulates neural coding. Science. 2016;353. 10.1126/science.aad4225 PMC4993111 27418512

[32] Sweatt JD. The emerging field of neuroepigenetics. Neuron. 2013;80:624–32. doi: 10.1016/j.neuron.2013.10.023 24183015 10.1016/j.neuron.2013.10.023 PMC3878295

[33] Halder R, Hennion M, Vidal RO, Shomroni O, Rahman RU, Rajput A, et al DNA methylation changes in plasticity genes accompany the formation and maintenance of memory. Nat Neurosci. 2016;19(1):102–10. doi: 10.1038/nn.4194 26656643 10.1038/nn.4194

[34] Hirano Y, Ihara K, Masuda T, Yamamoto T, Iwata I, Takahashi A, et al Shifting transcriptional machinery is required for long-term memory maintenance and modification in *Drosophila* mushroom bodies. Nat Commun. 2016;7:13471 doi: 10.1038/ncomms13471 27841260 10.1038/ncomms13471 PMC5114576

[35] Saul MC, Seward CH, Troy JM, Zhang H, Sloofman LG, Lu X, Weisner P. A., Caetano-Anolles D., Sun H., Zhao S. D., Chandrasekaran S., Sinha S., Stubbs L. Transcriptional regulatory dynamics drive coordinated metabolic and neural response to social challenge in mice. Genome Res. 2017 doi: 10.1101/gr.214221.116 28356321 10.1101/gr.214221.116 PMC5453329

[36] Wingfield JC, Hegner RE, Dufty, Alfred M., Ball GF. The challenge hypothesis: Theoretical implications for patterns of testosterone secretion, mating systems, and breeding strategies. Am Nat. 1990;136:829–46. doi: 10.1086/285134

[37] Bell AM, Backström T, Huntingford FA, Pottinger TG, Winberg S. Variable neuroendocrine responses to ecologically-relevant challenges in sticklebacks. Physiol Behav. 2007;91:15–25. doi: 10.1016/j.physbeh.2007.01.012 17321556 10.1016/j.physbeh.2007.01.012

[38] Shpigler HY, Saul M. C., Murdoch E. E., Cash-Ahmed A. C., Seward C. H., Sloofman L., Chandrasekaran S., Sinha S., Stubbs L. J., Robinson G. E. Behavioral, transcriptomic and epigenetic responses to social challenge in honey bees. Genes, Brain Behav. 2017. 10.1111/gbb.12379 28328153

[39] Blaeser F, Sanders MJ, Truong N, Ko S, Wu LJ, Wozniak DF, et al Long-term memory deficits in Pavlovian fear conditioning in Ca2+/calmodulin kinase kinase alpha-deficient mice. Mol Cell Biol. 2006;26(23):9105–15. doi: 10.1128/MCB.01452-06 17015467 10.1128/MCB.01452-06 PMC1636841

[40] Chen Z, Stockwell J, Cayabyab FS. Adenosine A1 receptor-mediated endocytosis of AMPA receptors contributes to impairments in long-term potentiation (LTP) in the middle-aged rat hippocampus. Neurochem Res. 2016;41(5):1085–97. doi: 10.1007/s11064-015-1799-3 26700433 10.1007/s11064-015-1799-3

[41] Filiano AJ, Xu Y, Tustison NJ, Marsh RL, Baker W, Smirnov I, et al Unexpected role of interferon-γ in regulating neuronal connectivity and social behaviour. Nature. 2016;535(7612):425–9. doi: 10.1038/nature18626 27409813 10.1038/nature18626 PMC4961620

[42] Derecki NC, Cardani AN, Yang CH, Quinnies KM, Crihfield A, Lynch KR, et al Regulation of learning and memory by meningeal immunity: a key role for IL-4. J Exp Med. 2010;207(5):1067–80. doi: 10.1084/jem.20091419 20439540 10.1084/jem.20091419 PMC2867291

[43] Stamps Judy A, Krishnan VV. How territorial animals compete for divisible space: A learning‐based model with unequal competitors. Am Nat. 2001;157:154–69. doi: 10.1086/318634 18707269 10.1086/318634

[44] Hollis KL. The role of learning in the aggressive and reproductive behavior of blue gouramis, *Trichogaster trichopterus*. Environ Biol Fishes. 1999;54:355–69. doi: 10.1023/A:1007529628117

[45] Bronstein JL. Conditional outcomes in mutualistic interactions. Trends Ecol Evol. 1994;9:214–7. doi: 10.1016/0169-5347(94)90246-1 21236825 10.1016/0169-5347(94)90246-1

[46] Jenkins JR, Rowland WJ. Pavlovian conditioning of agonistic behavior in male threespine stickleback (*Gasterosteus aculeatus*). J Comp Psychol. 1996;110:396–401. 8956509 10.1037/0735-7036.110.4.396

[47] Losey GS, Sevenster P. Can three-spined sticklebacks learn when to display? Rewarded displays. Anim Behav. 1995;49:137–50. doi: 10.1016/0003-3472(95)80161-8

[48] Huntingford FA, Turner AK. Conflict in the animal world Animal Conflict. Dordrecht: Springer Netherlands; 1987 p. 3–12.

[49] Hsu Y, Earley RL, Wolf LL. Modulation of aggressive behaviour by fighting experience: mechanisms and contest outcomes. Biol Rev Camb Philos Soc. 2006;81:33–74. doi: 10.1017/S146479310500686X 16460581 10.1017/S146479310500686X

[50] Temeles EJ. The role of neighbours in territorial systems: when are they 'dear enemies'? Anim Behav. 1994;47:339–50. doi: 10.1006/anbe.1994.1047

[51] Bronstein PM. Socially mediated learning in male *Betta splendens*. J Comp Psych. 1986;100:279–84. 3769447

[52] Bronstein PM. Aggression, by John Klama. London: Longman Scientific, 1988, 169 pp. Aggressive Behavior. 1988;14:463–4. doi: 10.1002/1098-2337(1988)14:6<::AID-AB2480140607>3.0.CO;2-T

[53] Li-Byarlay H, Rittschof CC, Massey JH, Pittendrigh BR, Robinson GE. Socially responsive effects of brain oxidative metabolism on aggression. Proc Natl Acad Sci U S A. 2014;111:12533–7. doi: 10.1073/pnas.1412306111 25092297 10.1073/pnas.1412306111 PMC4151721

[54] Rittschof Cc, Grozinger CM, Robinson GE. The energetic basis of behavior: Bridging behavioral ecology and neuroscience. Curr Opin Behav Sci. 2015;6:19–27.

[55] Chandrasekaran S, Rittschof CC, Djukovic D, Gu H, Raftery D, Price ND, et al Aggression is associated with aerobic glycolysis in the honey bee brain. Genes Brain Behav. 2015;14(2):158–66. doi: 10.1111/gbb.12201 25640316 10.1111/gbb.12201 PMC4449359

[56] Kawai M, Rosen CJ. PPARγ: a circadian transcription factor in adipogenesis and osteogenesis. Nat Rev Endocrinol. 2010;6:629–36. doi: 10.1038/nrendo.2010.155 20820194 10.1038/nrendo.2010.155 PMC3132113

[57] Ryan KK, Li B, Grayson BE, Matter EK, Woods SC, Seeley RJ. A role for central nervous system PPAR-γ in the regulation of energy balance. Nat Med. 2011;17:623–6. doi: 10.1038/nm.2349 21532595 10.1038/nm.2349 PMC3089657

[58] Sarruf DA, Yu F, Nguyen HT, Williams DL, Printz RL, Niswender KD, et al Expression of peroxisome proliferator-activated receptor-gamma in key neuronal subsets regulating glucose metabolism and energy homeostasis. Endocrinology. 2009;150:707–12. doi: 10.1210/en.2008-0899 18845632 10.1210/en.2008-0899 PMC2646542

[59] Goldschmidt T, Bakker TC. Determinants of reproductive success of male sticklebacks in the field and in the laboratory. Netherlands Journal of Zoology. 1990;40:664–87.

[60] Iersel JJAv. Some aspects of territorial behaviour of the male three-spined stickleback. Archs neerl Zool. 1958;13, Suppl. 1:381–400.

[61] Langmead B, Salzberg SL. Fast gapped-read alignment with Bowtie 2. Nat Methods. 2012;9:357–9. doi: 10.1038/nmeth.1923 22388286 10.1038/nmeth.1923 PMC3322381

[62] Heinz S, Benner C, Spann N, Bertolino E, Lin YC, Laslo P, et al Simple combinations of lineage-determining transcription factors prime cis-regulatory elements required for macrophage and B cell identities. Mol Cell. 2010;38:576–89. doi: 10.1016/j.molcel.2010.05.004 20513432 10.1016/j.molcel.2010.05.004 PMC2898526

[63] Thomas PD, Campbell MJ, Kejariwal A, Mi H, Karlak B, Daverman R, et al PANTHER: a library of protein families and subfamilies indexed by function. Genome Res. 2003;13: 2129–2141. doi: 10.1101/gr.772403 12952881 10.1101/gr.772403 PMC403709

[64] Yu G, Li F, Qin Y, Bo X, Wu Y, Wang S. GOSemSim: an R package for measuring semantic similarity among GO terms and gene products. Bioinformatics. 2010;26(7):976–8. doi: 10.1093/bioinformatics/btq064 20179076 10.1093/bioinformatics/btq064

[65] Guzman SJ, Gerevich Z, Guzman SJ, Gerevich Z. P2Y receptors in synaptic transmission and plasticity: Therapeutic potential in cognitive dysfunction. Neural Plasticity. 2016;2016:1–12. doi: 10.1155/2016/1207393 27069691 10.1155/2016/1207393 PMC4812485

[66] Haynes SE, Hollopeter G, Yang G, Kurpius D, Dailey ME, Gan W-B, et al The P2Y12 receptor regulates microglial activation by extracellular nucleotides. Nat Neurosci. 2006;9:1512–9. doi: 10.1038/nn1805 17115040 10.1038/nn1805

