# Supplementary material for: Temporal dynamics of neurogenomic plasticity in response to social interactions in male threespined sticklebacks
Source: PLoS Genet. 2017 Jul 13;13(7):e1006840. doi: 10.1371/journal.pgen.1006840 (PMC5509087; doi:10.1371/journal.pgen.1006840)
Supplement: S1 Fig — The TRN contains 352 TFs, which regulate 1155 genes through a total of 3683 interactions. The top 20 TFs (“hubs”) with the highest number of targets (over 30 each) are highlighted in pink. Target genes that are differentially expressed in Diencephalon or Telencephalon (CFDR < 0.1) are shown as blue or green nodes respectively. Genes in the TRN that were differentially expressed in both the regions are shown in orange. (DOCX) [file pgen.1006840.s001.docx]

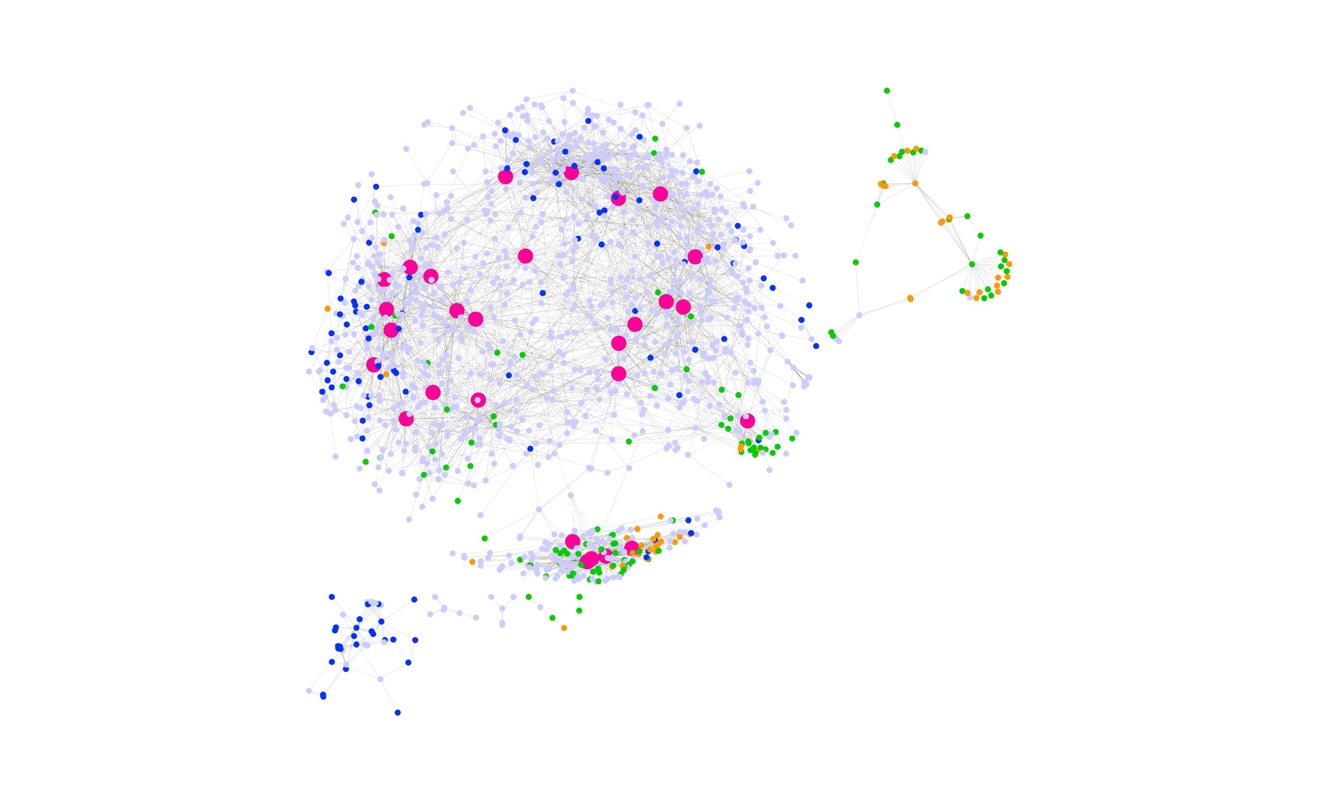


S1 Figure. TRN highlighting top hubs in the network and region-specific DEGs. The TRN contains 352 TFs, which regulate 1155 genes through a total of 3683 interactions. The top 20 TFs (“hubs”) with the highest number of targets (over 30 each) are highlighted in pink. Target genes that are differentially expressed in Diencephalon or Telencephalon (CFDR < 0.1) are shown as blue or green nodes respectively. Genes in the TRN that were differentially expressed in both the regions are shown in orange.
