# Supplementary figures and images for: Temporal dynamics of neurogenomic plasticity in response to social interactions in male threespined sticklebacks (part 1 of 2)
Source: PLoS Genet. 2017 Jul 13;13(7):e1006840. doi: 10.1371/journal.pgen.1006840 (PMC5509087; doi:10.1371/journal.pgen.1006840)

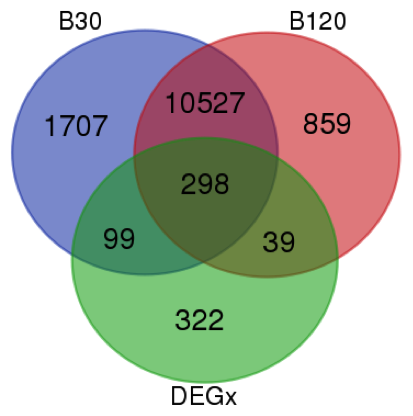


S2 Figure. Baseline accessibility of diencephalon DEGx at 30 and 120 minutes.

Supplement: S2 Fig — (DOCX) [file pgen.1006840.s002.docx]

## Mapped Counts All Samples

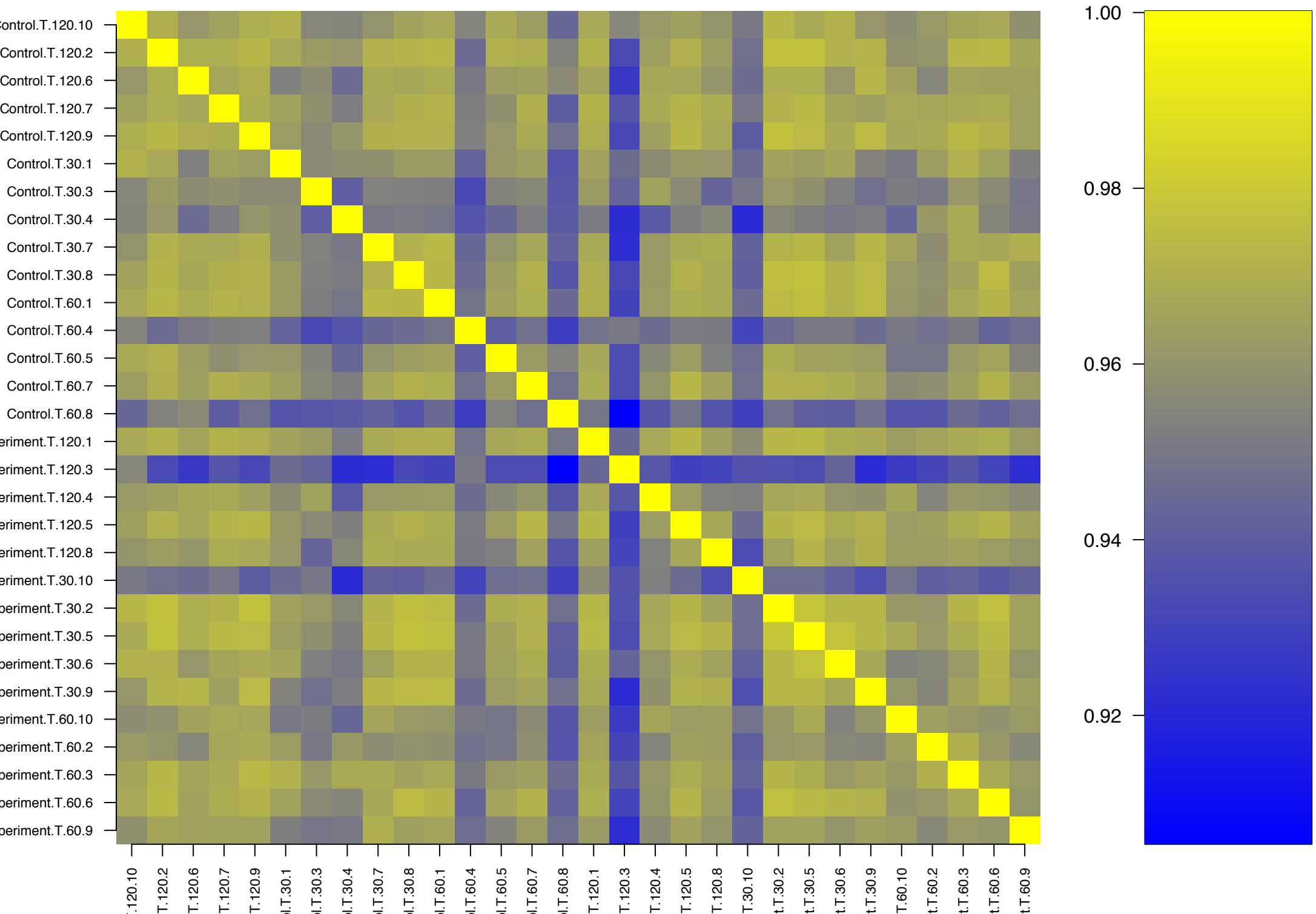

Supplement: S1 Data — This directory contains six subdirectories. The subdirectory “correlation” contains correlation heatmaps among all samples in diencephalon and telencephalon respectively. The subdirectory “mapping_stat” contains read mapping information on genomic features for each sample. The subdirectory “MDS” shows three dimensional MDS plots of the samples. The subdirectory “ReadDuplication” contains read duplication distributions for each sample. The subdirectory “ReadQuality” contains reads quality information for each sample plotted as both boxplots and heatmaps. The subdirectory “RPKMSaturation” contains information about read depth saturation for each sample as assessed by RPKM resamplings. All transcripts were divided into four quantiles based on their expression and a relative difference of observed and real RPKM values are plotted for each sample. (ZIP) [file pgen.1006840.s015.zip › RNASeq/correlation/T.all.samples.correlation.pdf]

Diencephalon samples 3D MDS plots


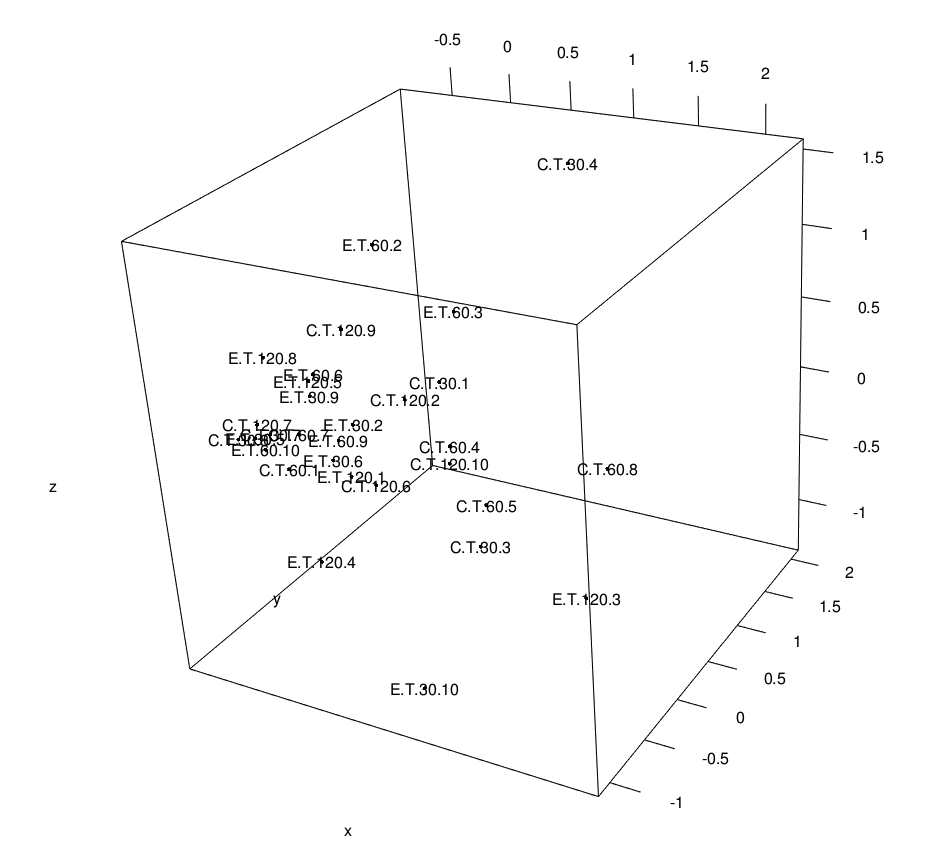


Telencephalon samples 3D MDS plots


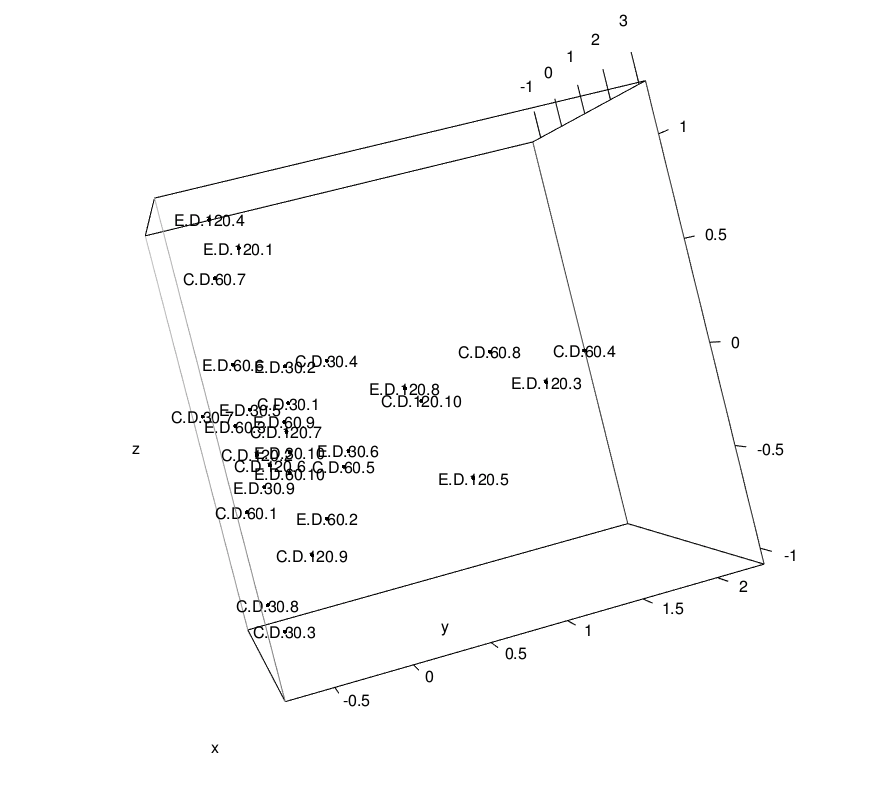

Supplement: S1 Data — This directory contains six subdirectories. The subdirectory “correlation” contains correlation heatmaps among all samples in diencephalon and telencephalon respectively. The subdirectory “mapping_stat” contains read mapping information on genomic features for each sample. The subdirectory “MDS” shows three dimensional MDS plots of the samples. The subdirectory “ReadDuplication” contains read duplication distributions for each sample. The subdirectory “ReadQuality” contains reads quality information for each sample plotted as both boxplots and heatmaps. The subdirectory “RPKMSaturation” contains information about read depth saturation for each sample as assessed by RPKM resamplings. All transcripts were divided into four quantiles based on their expression and a relative difference of observed and real RPKM values are plotted for each sample. (ZIP) [file pgen.1006840.s015.zip › RNASeq/MDS/Samples 3D MDS plots.docx]

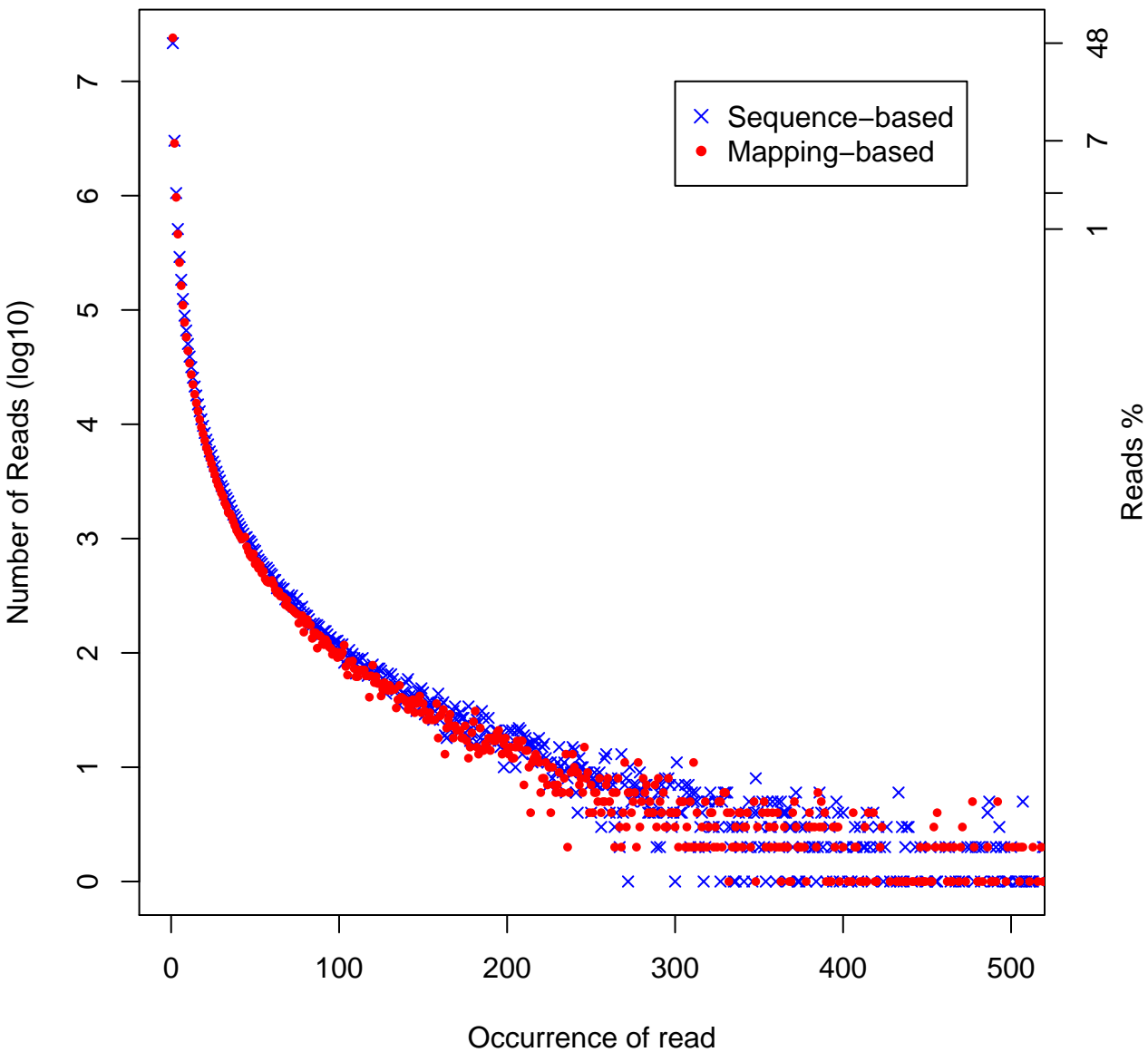

Supplement: S1 Data — This directory contains six subdirectories. The subdirectory “correlation” contains correlation heatmaps among all samples in diencephalon and telencephalon respectively. The subdirectory “mapping_stat” contains read mapping information on genomic features for each sample. The subdirectory “MDS” shows three dimensional MDS plots of the samples. The subdirectory “ReadDuplication” contains read duplication distributions for each sample. The subdirectory “ReadQuality” contains reads quality information for each sample plotted as both boxplots and heatmaps. The subdirectory “RPKMSaturation” contains information about read depth saturation for each sample as assessed by RPKM resamplings. All transcripts were divided into four quantiles based on their expression and a relative difference of observed and real RPKM values are plotted for each sample. (ZIP) [file pgen.1006840.s015.zip › RNASeq/ReadDuplication/10D_CGCTCATT-ATAGAGGC_L00M_R1_001.DupRate_plot.pdf]

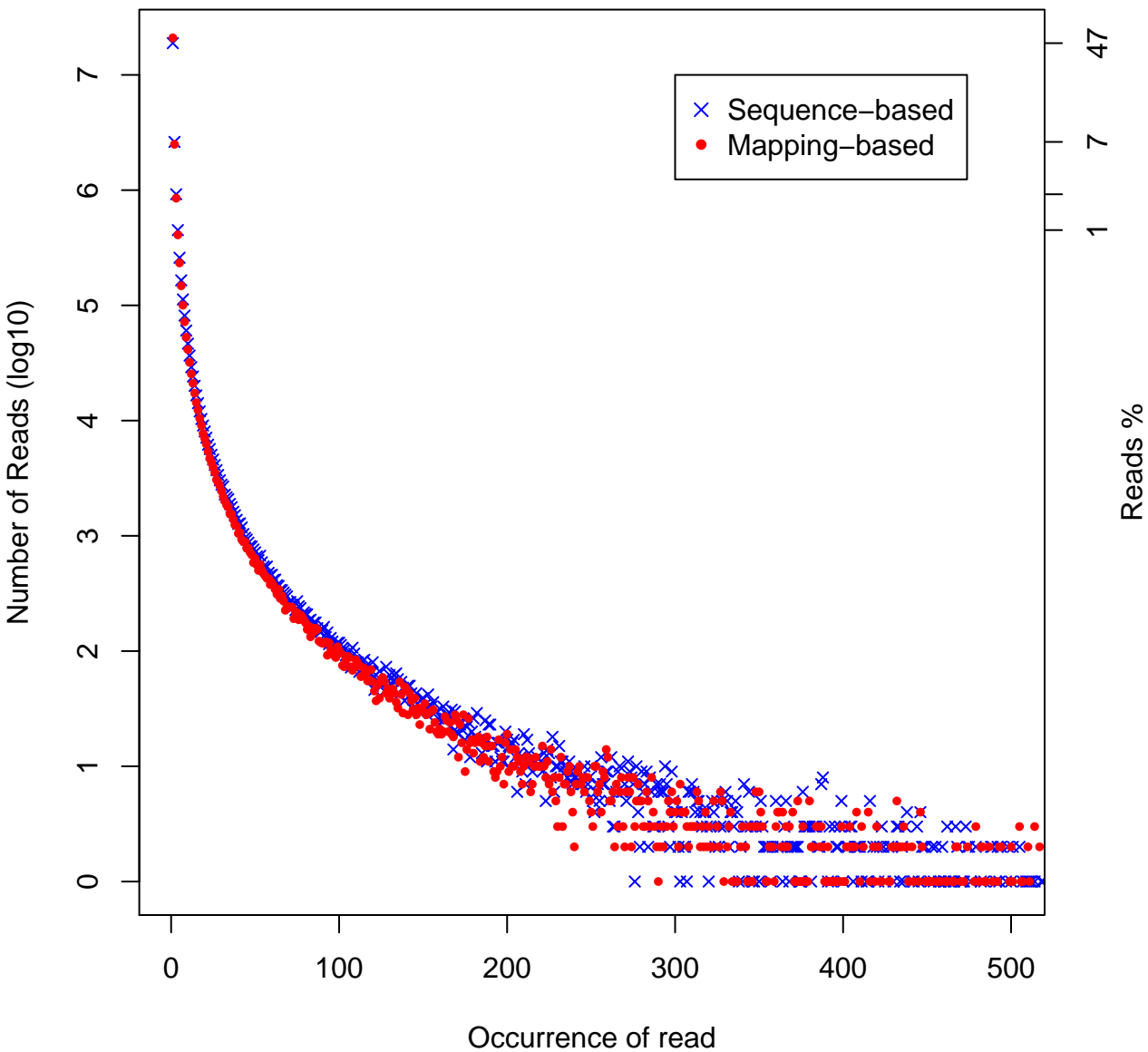

Supplement: S1 Data — This directory contains six subdirectories. The subdirectory “correlation” contains correlation heatmaps among all samples in diencephalon and telencephalon respectively. The subdirectory “mapping_stat” contains read mapping information on genomic features for each sample. The subdirectory “MDS” shows three dimensional MDS plots of the samples. The subdirectory “ReadDuplication” contains read duplication distributions for each sample. The subdirectory “ReadQuality” contains reads quality information for each sample plotted as both boxplots and heatmaps. The subdirectory “RPKMSaturation” contains information about read depth saturation for each sample as assessed by RPKM resamplings. All transcripts were divided into four quantiles based on their expression and a relative difference of observed and real RPKM values are plotted for each sample. (ZIP) [file pgen.1006840.s015.zip › RNASeq/ReadDuplication/10T_CGCTCATT-TATAGCCT_L00M_R1_001.DupRate_plot.pdf]

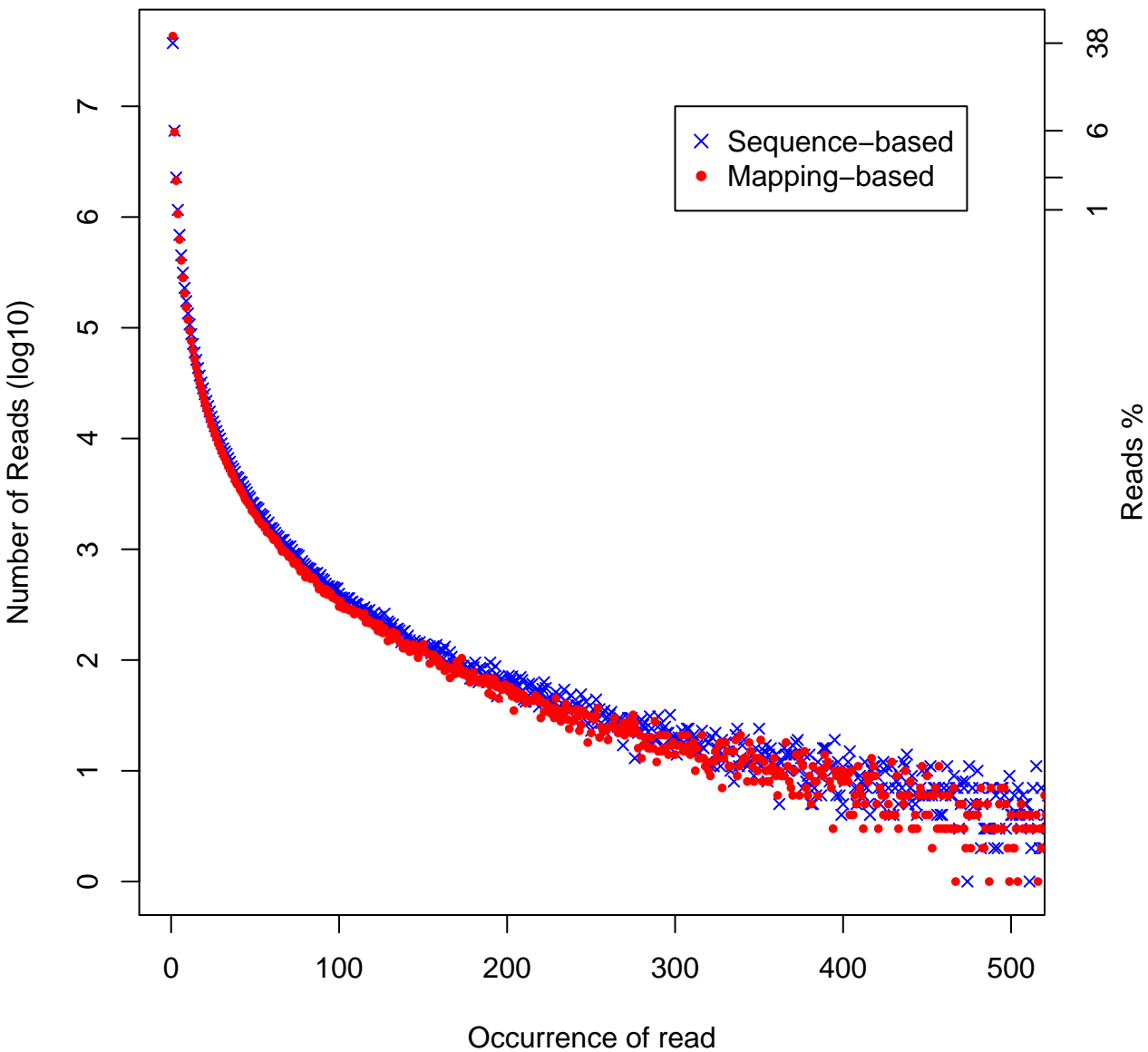

Supplement: S1 Data — This directory contains six subdirectories. The subdirectory “correlation” contains correlation heatmaps among all samples in diencephalon and telencephalon respectively. The subdirectory “mapping_stat” contains read mapping information on genomic features for each sample. The subdirectory “MDS” shows three dimensional MDS plots of the samples. The subdirectory “ReadDuplication” contains read duplication distributions for each sample. The subdirectory “ReadQuality” contains reads quality information for each sample plotted as both boxplots and heatmaps. The subdirectory “RPKMSaturation” contains information about read depth saturation for each sample as assessed by RPKM resamplings. All transcripts were divided into four quantiles based on their expression and a relative difference of observed and real RPKM values are plotted for each sample. (ZIP) [file pgen.1006840.s015.zip › RNASeq/ReadDuplication/11D_CGCTCATT-GGCTCTGA_L00M_R1_001.DupRate_plot.pdf]

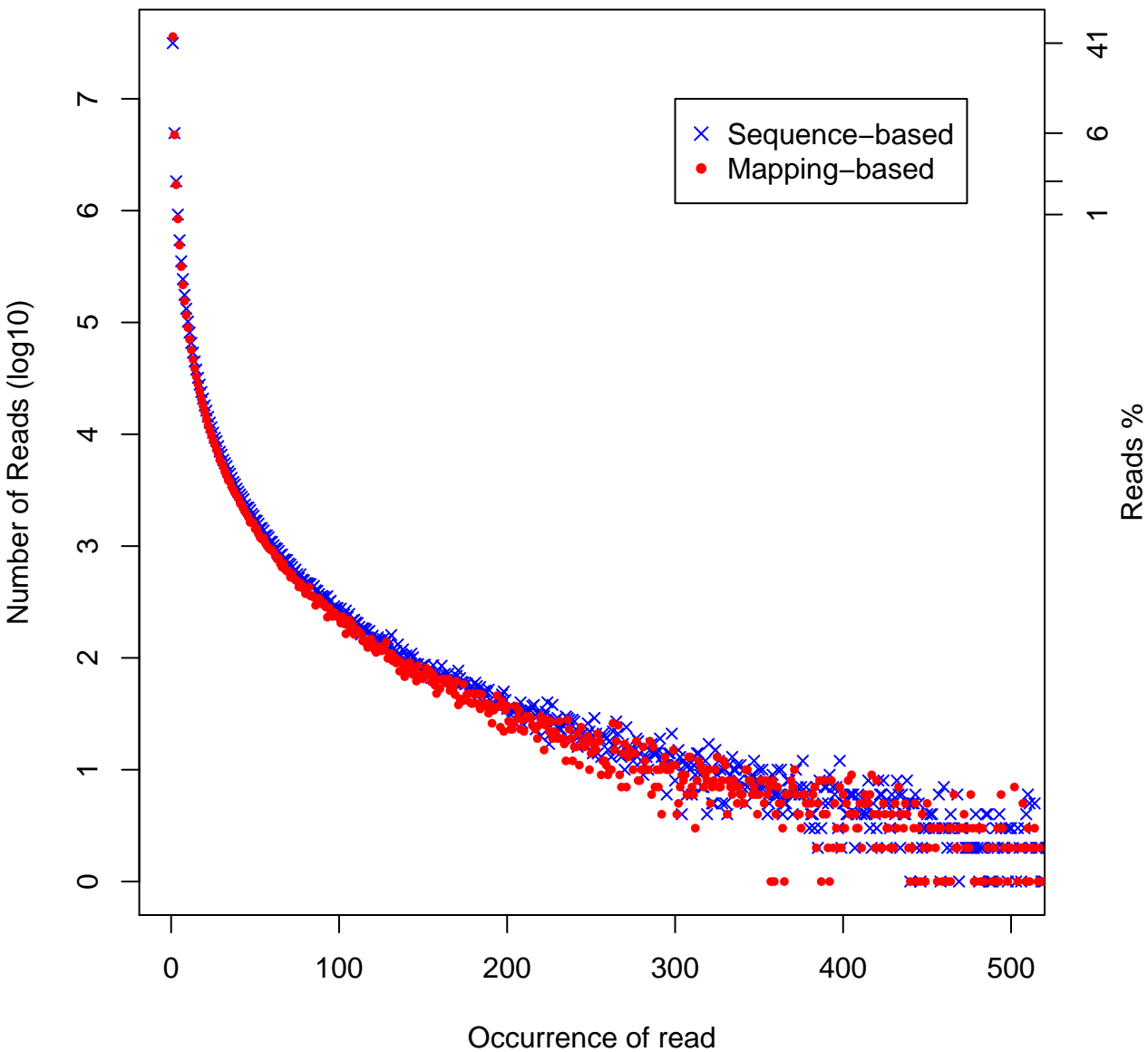

Supplement: S1 Data — This directory contains six subdirectories. The subdirectory “correlation” contains correlation heatmaps among all samples in diencephalon and telencephalon respectively. The subdirectory “mapping_stat” contains read mapping information on genomic features for each sample. The subdirectory “MDS” shows three dimensional MDS plots of the samples. The subdirectory “ReadDuplication” contains read duplication distributions for each sample. The subdirectory “ReadQuality” contains reads quality information for each sample plotted as both boxplots and heatmaps. The subdirectory “RPKMSaturation” contains information about read depth saturation for each sample as assessed by RPKM resamplings. All transcripts were divided into four quantiles based on their expression and a relative difference of observed and real RPKM values are plotted for each sample. (ZIP) [file pgen.1006840.s015.zip › RNASeq/ReadDuplication/11T_CGCTCATT-CCTATCCT_L00M_R1_001.DupRate_plot.pdf]

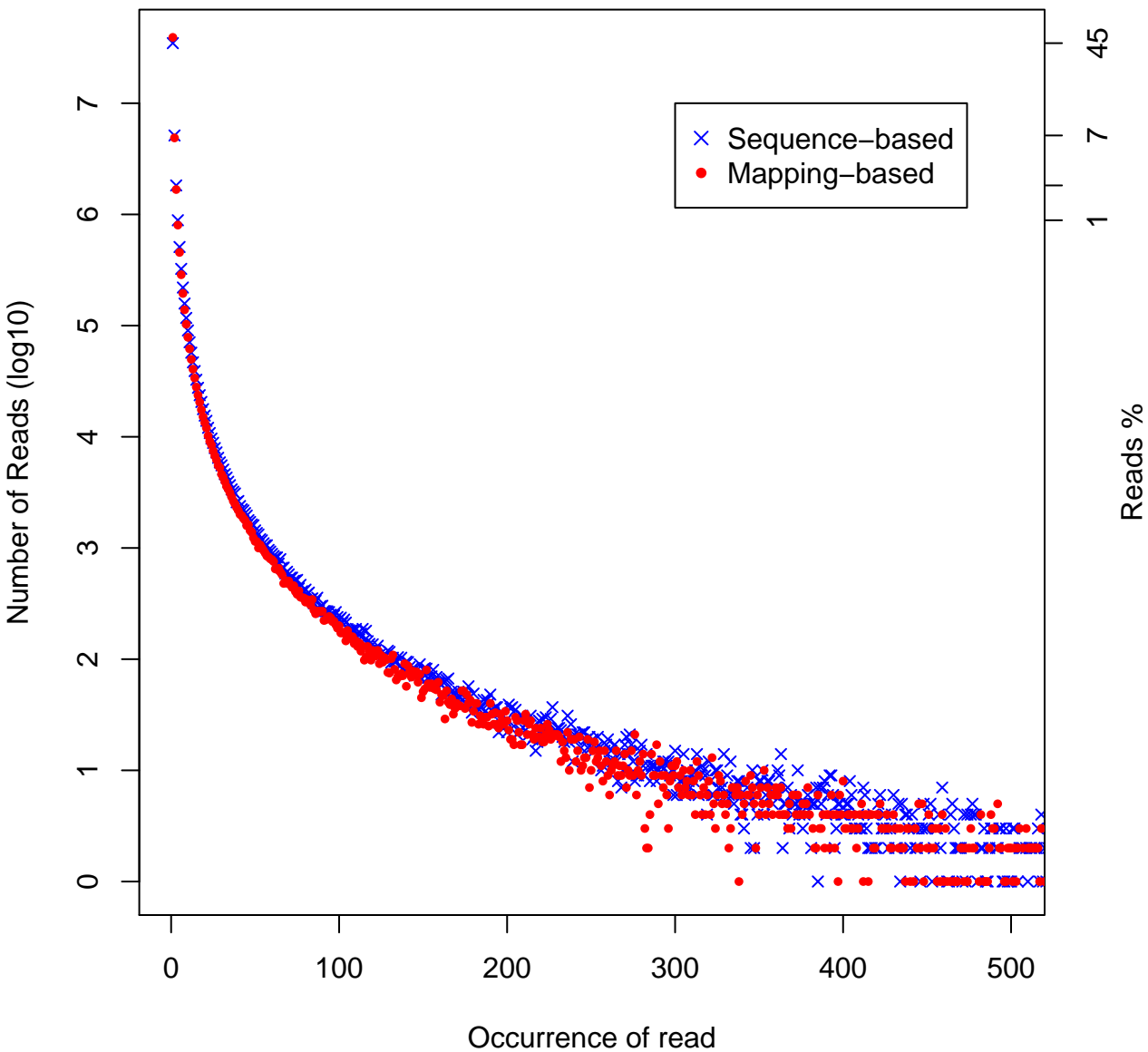

Supplement: S1 Data — This directory contains six subdirectories. The subdirectory “correlation” contains correlation heatmaps among all samples in diencephalon and telencephalon respectively. The subdirectory “mapping_stat” contains read mapping information on genomic features for each sample. The subdirectory “MDS” shows three dimensional MDS plots of the samples. The subdirectory “ReadDuplication” contains read duplication distributions for each sample. The subdirectory “ReadQuality” contains reads quality information for each sample plotted as both boxplots and heatmaps. The subdirectory “RPKMSaturation” contains information about read depth saturation for each sample as assessed by RPKM resamplings. All transcripts were divided into four quantiles based on their expression and a relative difference of observed and real RPKM values are plotted for each sample. (ZIP) [file pgen.1006840.s015.zip › RNASeq/ReadDuplication/12D_CGCTCATT-TAATCTTA_L00M_R1_001.DupRate_plot.pdf]

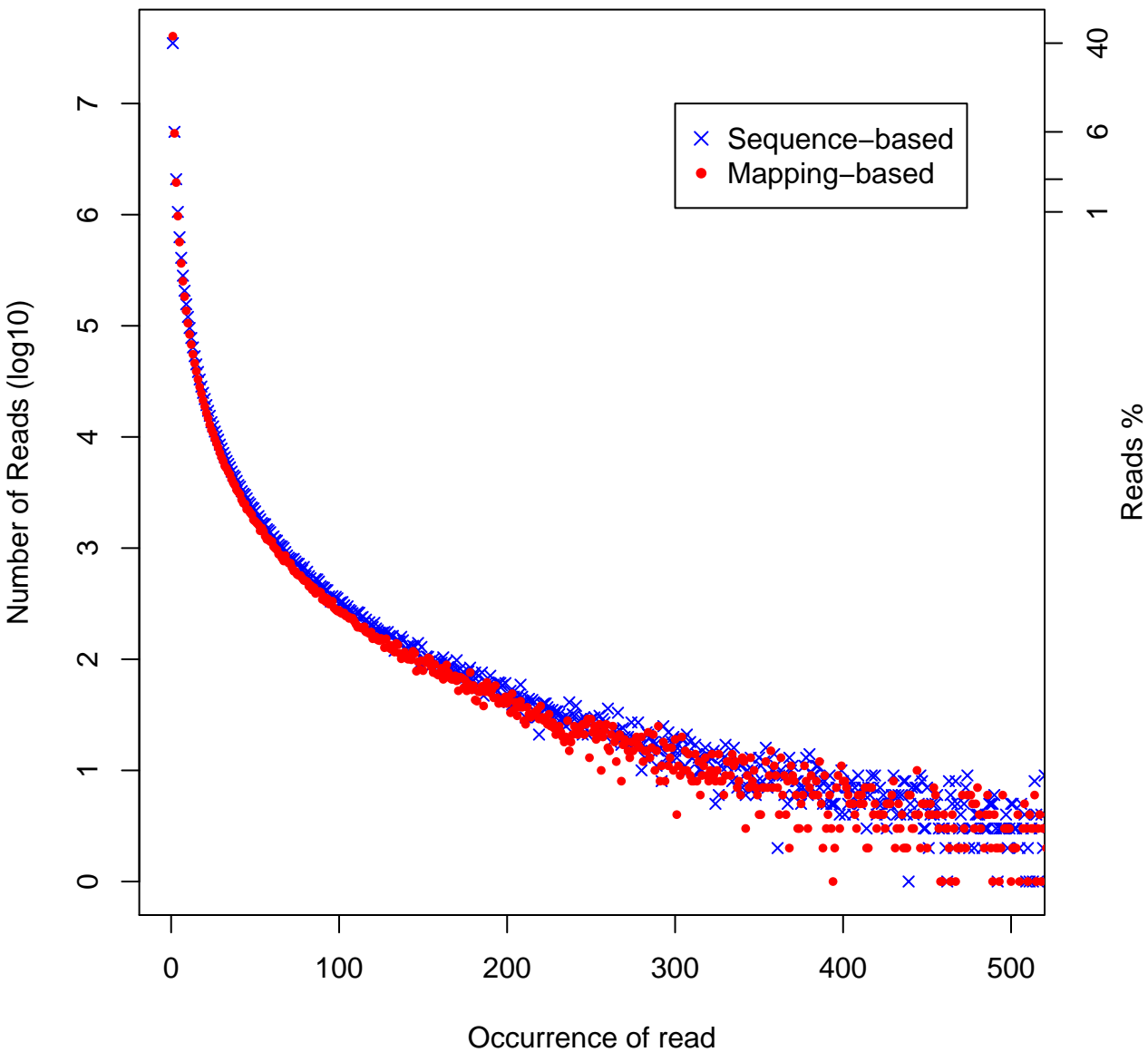

Supplement: S1 Data — This directory contains six subdirectories. The subdirectory “correlation” contains correlation heatmaps among all samples in diencephalon and telencephalon respectively. The subdirectory “mapping_stat” contains read mapping information on genomic features for each sample. The subdirectory “MDS” shows three dimensional MDS plots of the samples. The subdirectory “ReadDuplication” contains read duplication distributions for each sample. The subdirectory “ReadQuality” contains reads quality information for each sample plotted as both boxplots and heatmaps. The subdirectory “RPKMSaturation” contains information about read depth saturation for each sample as assessed by RPKM resamplings. All transcripts were divided into four quantiles based on their expression and a relative difference of observed and real RPKM values are plotted for each sample. (ZIP) [file pgen.1006840.s015.zip › RNASeq/ReadDuplication/12T_CGCTCATT-AGGCGAAG_L00M_R1_001.DupRate_plot.pdf]

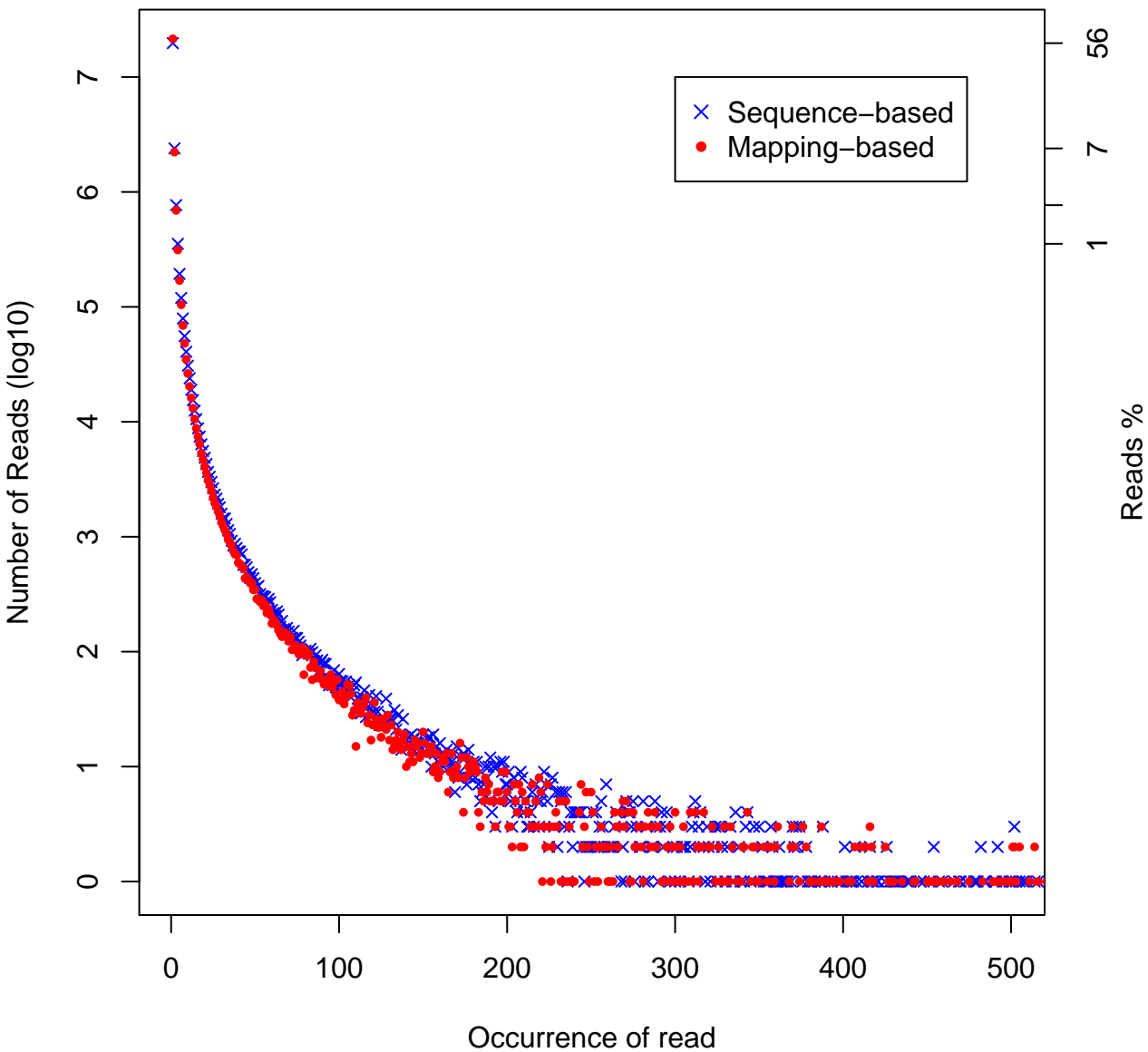

Supplement: S1 Data — This directory contains six subdirectories. The subdirectory “correlation” contains correlation heatmaps among all samples in diencephalon and telencephalon respectively. The subdirectory “mapping_stat” contains read mapping information on genomic features for each sample. The subdirectory “MDS” shows three dimensional MDS plots of the samples. The subdirectory “ReadDuplication” contains read duplication distributions for each sample. The subdirectory “ReadQuality” contains reads quality information for each sample plotted as both boxplots and heatmaps. The subdirectory “RPKMSaturation” contains information about read depth saturation for each sample as assessed by RPKM resamplings. All transcripts were divided into four quantiles based on their expression and a relative difference of observed and real RPKM values are plotted for each sample. (ZIP) [file pgen.1006840.s015.zip › RNASeq/ReadDuplication/13D_CGCTCATT-GTACTGAC_L00M_R1_001.DupRate_plot.pdf]

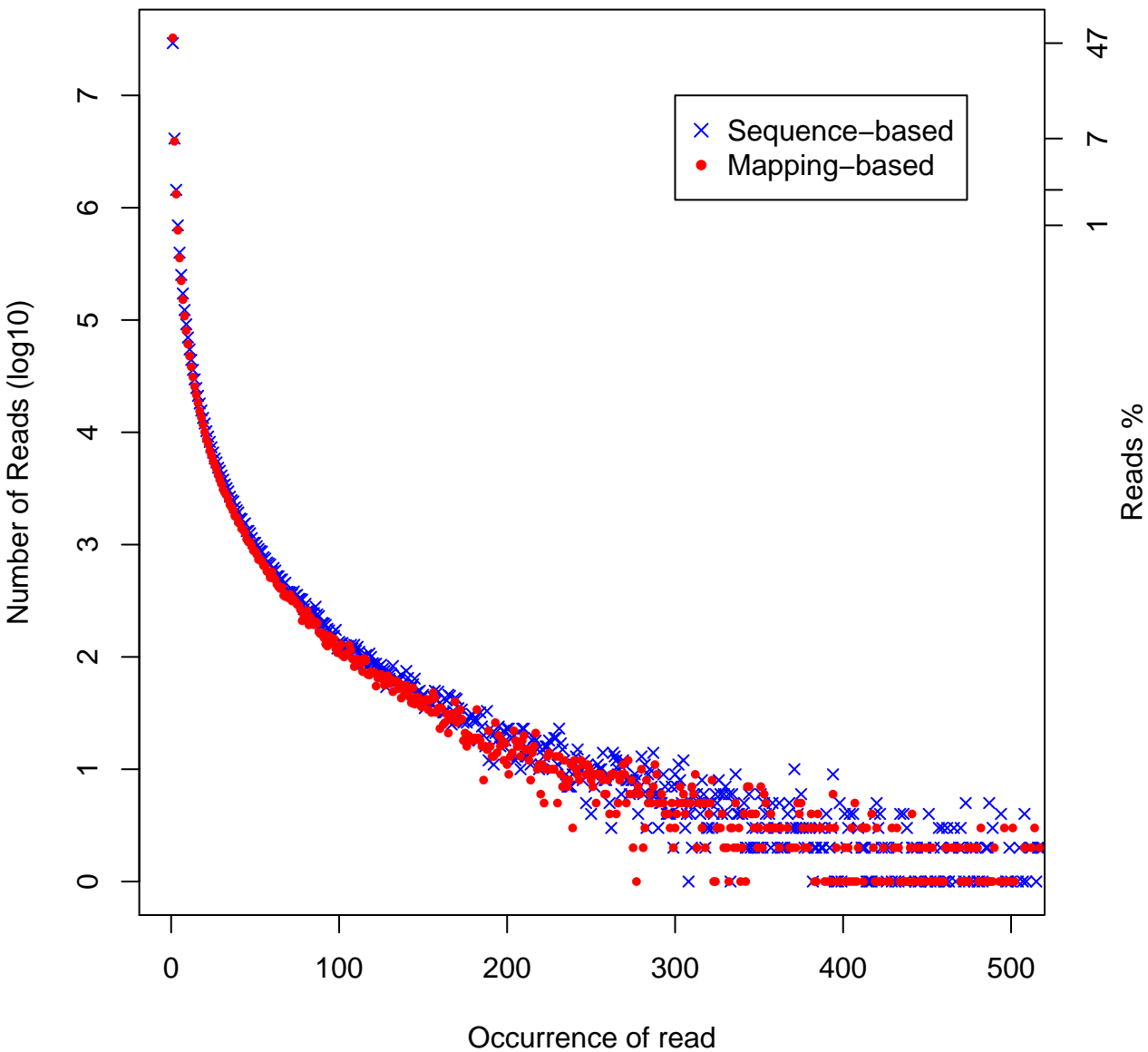

Supplement: S1 Data — This directory contains six subdirectories. The subdirectory “correlation” contains correlation heatmaps among all samples in diencephalon and telencephalon respectively. The subdirectory “mapping_stat” contains read mapping information on genomic features for each sample. The subdirectory “MDS” shows three dimensional MDS plots of the samples. The subdirectory “ReadDuplication” contains read duplication distributions for each sample. The subdirectory “ReadQuality” contains reads quality information for each sample plotted as both boxplots and heatmaps. The subdirectory “RPKMSaturation” contains information about read depth saturation for each sample as assessed by RPKM resamplings. All transcripts were divided into four quantiles based on their expression and a relative difference of observed and real RPKM values are plotted for each sample. (ZIP) [file pgen.1006840.s015.zip › RNASeq/ReadDuplication/13T_CGCTCATT-CAGGACGT_L00M_R1_001.DupRate_plot.pdf]

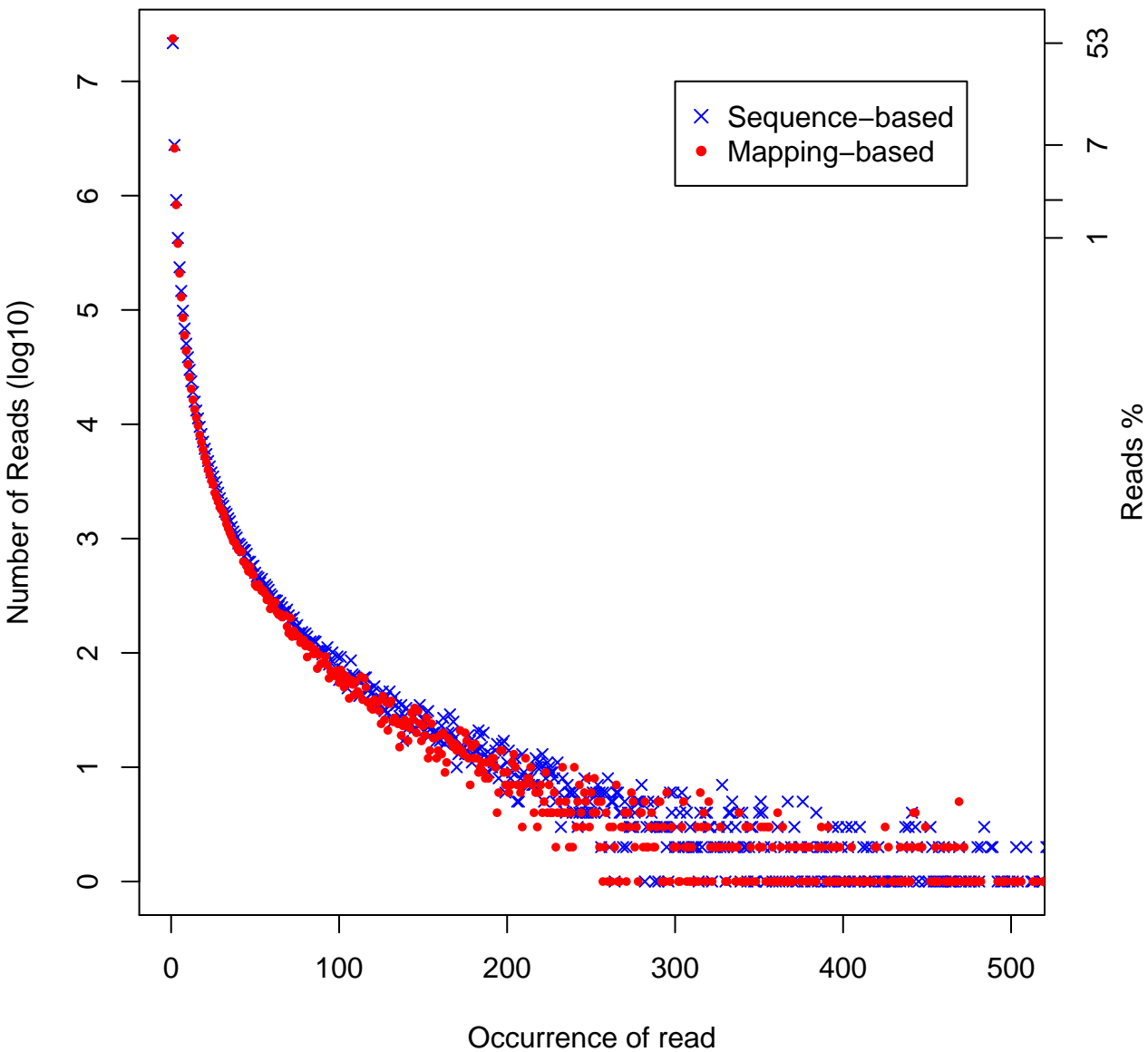

Supplement: S1 Data — This directory contains six subdirectories. The subdirectory “correlation” contains correlation heatmaps among all samples in diencephalon and telencephalon respectively. The subdirectory “mapping_stat” contains read mapping information on genomic features for each sample. The subdirectory “MDS” shows three dimensional MDS plots of the samples. The subdirectory “ReadDuplication” contains read duplication distributions for each sample. The subdirectory “ReadQuality” contains reads quality information for each sample plotted as both boxplots and heatmaps. The subdirectory “RPKMSaturation” contains information about read depth saturation for each sample as assessed by RPKM resamplings. All transcripts were divided into four quantiles based on their expression and a relative difference of observed and real RPKM values are plotted for each sample. (ZIP) [file pgen.1006840.s015.zip › RNASeq/ReadDuplication/15D_GAGATTCC-ATAGAGGC_L00M_R1_001.DupRate_plot.pdf]

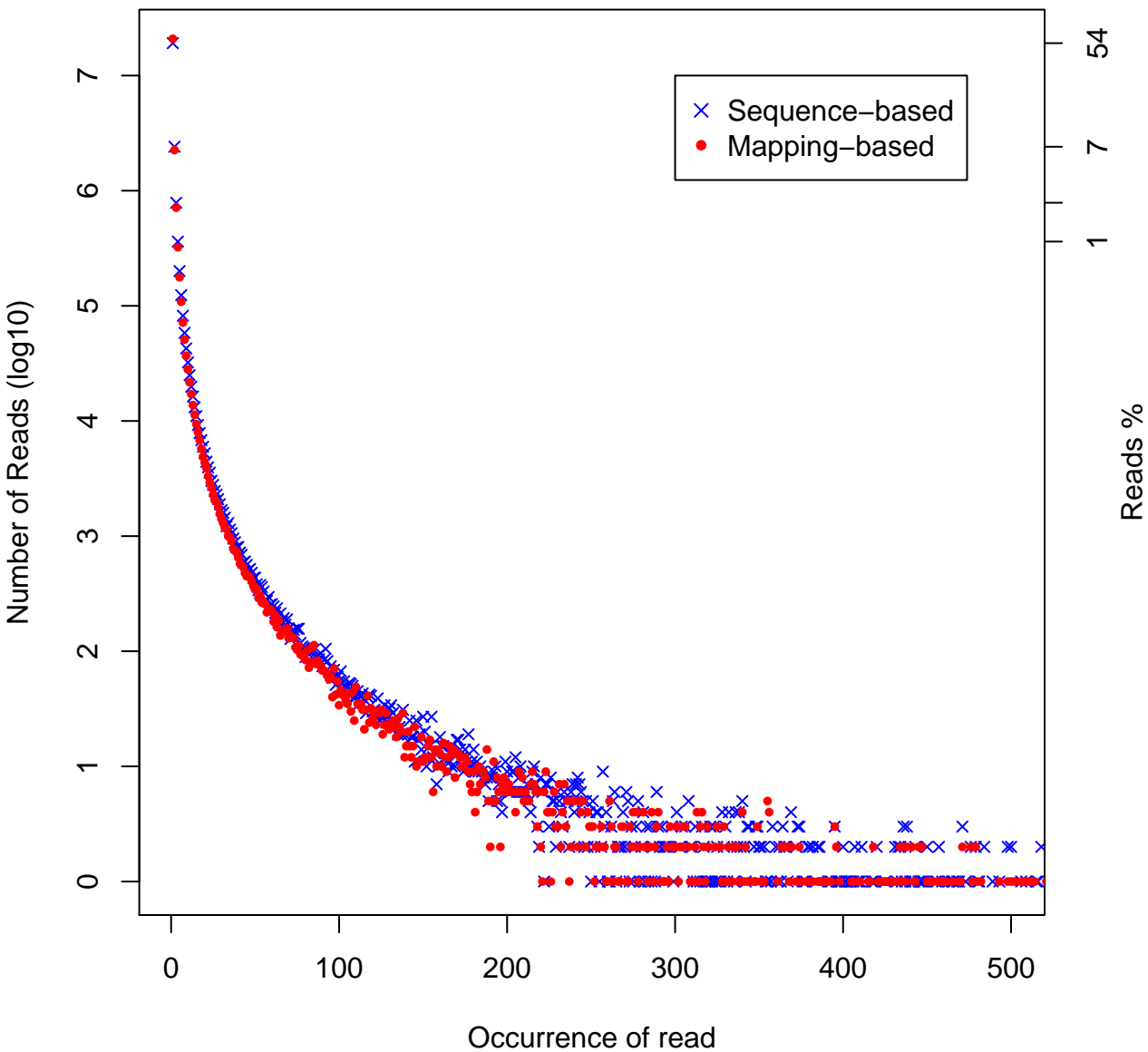

Supplement: S1 Data — This directory contains six subdirectories. The subdirectory “correlation” contains correlation heatmaps among all samples in diencephalon and telencephalon respectively. The subdirectory “mapping_stat” contains read mapping information on genomic features for each sample. The subdirectory “MDS” shows three dimensional MDS plots of the samples. The subdirectory “ReadDuplication” contains read duplication distributions for each sample. The subdirectory “ReadQuality” contains reads quality information for each sample plotted as both boxplots and heatmaps. The subdirectory “RPKMSaturation” contains information about read depth saturation for each sample as assessed by RPKM resamplings. All transcripts were divided into four quantiles based on their expression and a relative difference of observed and real RPKM values are plotted for each sample. (ZIP) [file pgen.1006840.s015.zip › RNASeq/ReadDuplication/15T_GAGATTCC-TATAGCCT_L00M_R1_001.DupRate_plot.pdf]

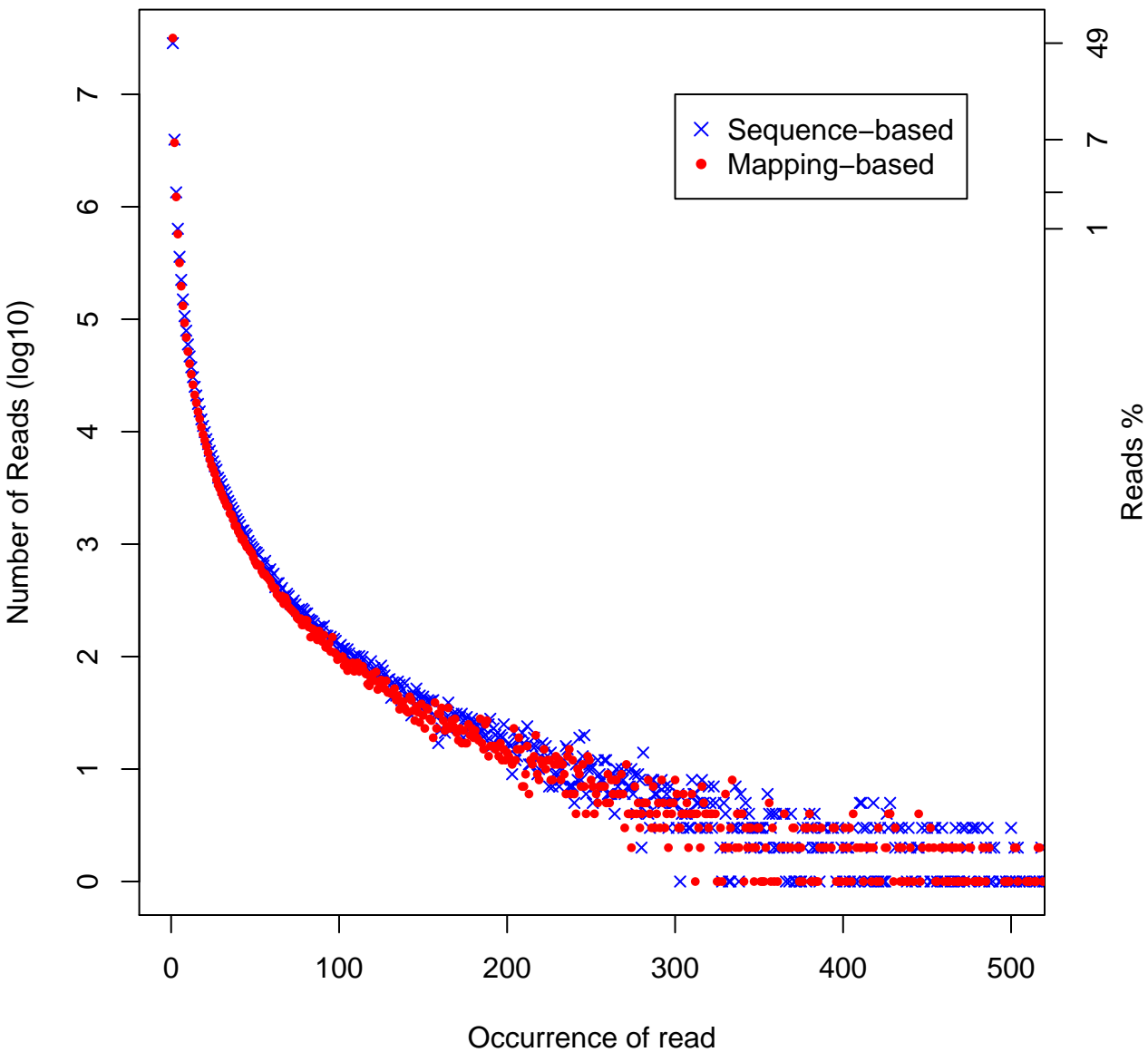

Supplement: S1 Data — This directory contains six subdirectories. The subdirectory “correlation” contains correlation heatmaps among all samples in diencephalon and telencephalon respectively. The subdirectory “mapping_stat” contains read mapping information on genomic features for each sample. The subdirectory “MDS” shows three dimensional MDS plots of the samples. The subdirectory “ReadDuplication” contains read duplication distributions for each sample. The subdirectory “ReadQuality” contains reads quality information for each sample plotted as both boxplots and heatmaps. The subdirectory “RPKMSaturation” contains information about read depth saturation for each sample as assessed by RPKM resamplings. All transcripts were divided into four quantiles based on their expression and a relative difference of observed and real RPKM values are plotted for each sample. (ZIP) [file pgen.1006840.s015.zip › RNASeq/ReadDuplication/17D_GAGATTCC-GGCTCTGA_L00M_R1_001.DupRate_plot.pdf]

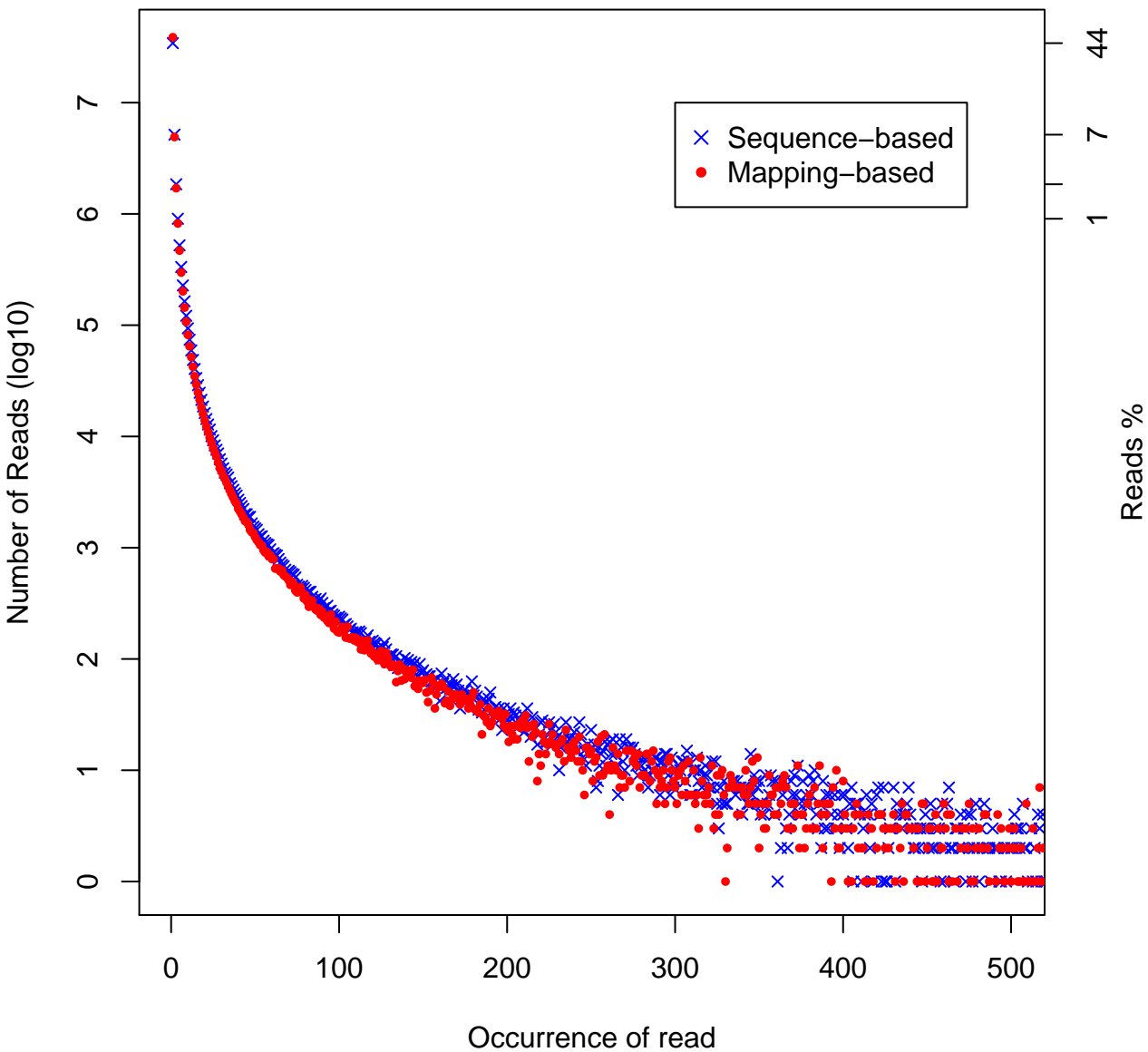

Supplement: S1 Data — This directory contains six subdirectories. The subdirectory “correlation” contains correlation heatmaps among all samples in diencephalon and telencephalon respectively. The subdirectory “mapping_stat” contains read mapping information on genomic features for each sample. The subdirectory “MDS” shows three dimensional MDS plots of the samples. The subdirectory “ReadDuplication” contains read duplication distributions for each sample. The subdirectory “ReadQuality” contains reads quality information for each sample plotted as both boxplots and heatmaps. The subdirectory “RPKMSaturation” contains information about read depth saturation for each sample as assessed by RPKM resamplings. All transcripts were divided into four quantiles based on their expression and a relative difference of observed and real RPKM values are plotted for each sample. (ZIP) [file pgen.1006840.s015.zip › RNASeq/ReadDuplication/17T_GAGATTCC-CCTATCCT_L00M_R1_001.DupRate_plot.pdf]

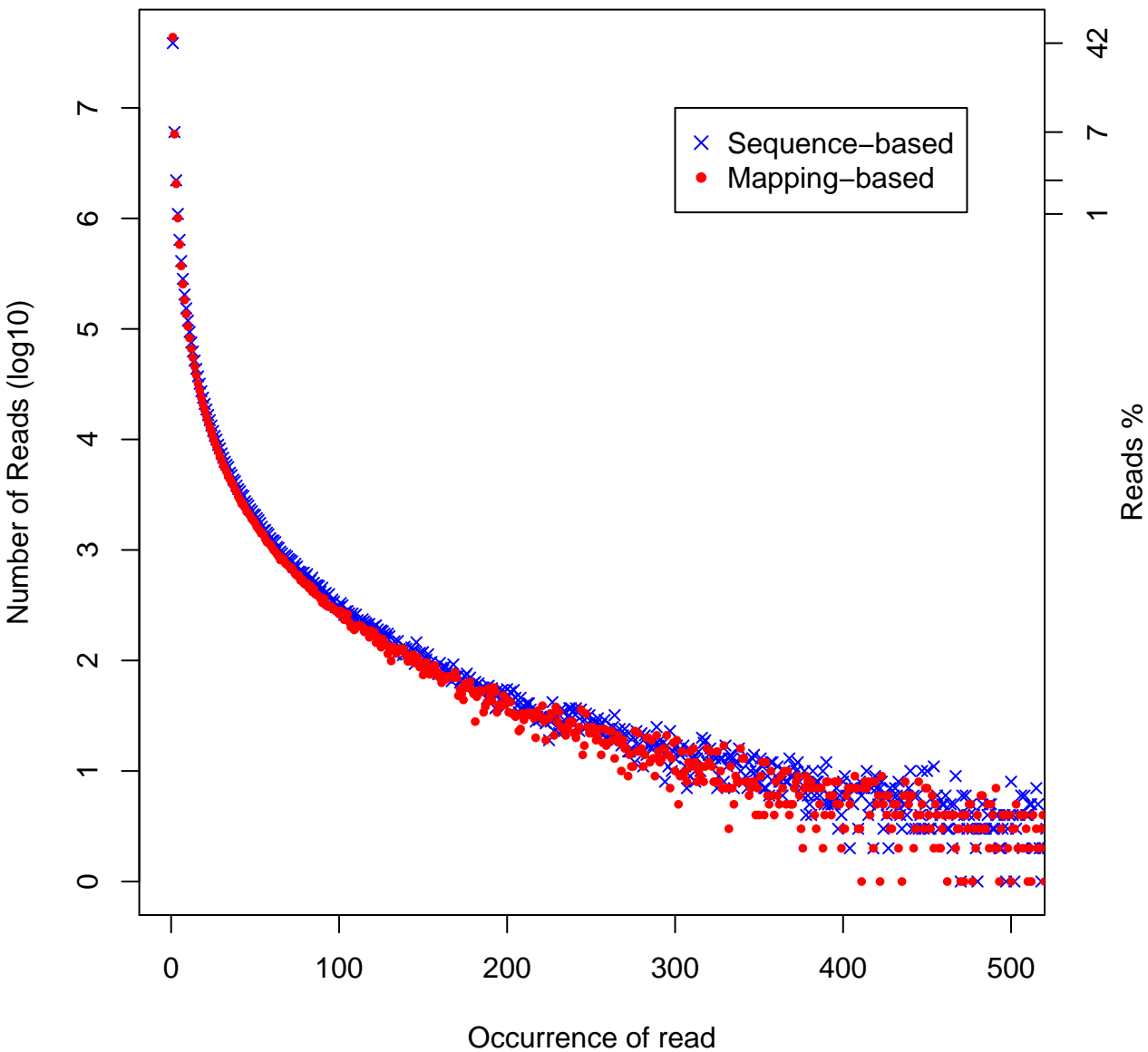

Supplement: S1 Data — This directory contains six subdirectories. The subdirectory “correlation” contains correlation heatmaps among all samples in diencephalon and telencephalon respectively. The subdirectory “mapping_stat” contains read mapping information on genomic features for each sample. The subdirectory “MDS” shows three dimensional MDS plots of the samples. The subdirectory “ReadDuplication” contains read duplication distributions for each sample. The subdirectory “ReadQuality” contains reads quality information for each sample plotted as both boxplots and heatmaps. The subdirectory “RPKMSaturation” contains information about read depth saturation for each sample as assessed by RPKM resamplings. All transcripts were divided into four quantiles based on their expression and a relative difference of observed and real RPKM values are plotted for each sample. (ZIP) [file pgen.1006840.s015.zip › RNASeq/ReadDuplication/18D_GAGATTCC-TAATCTTA_L00M_R1_001.DupRate_plot.pdf]

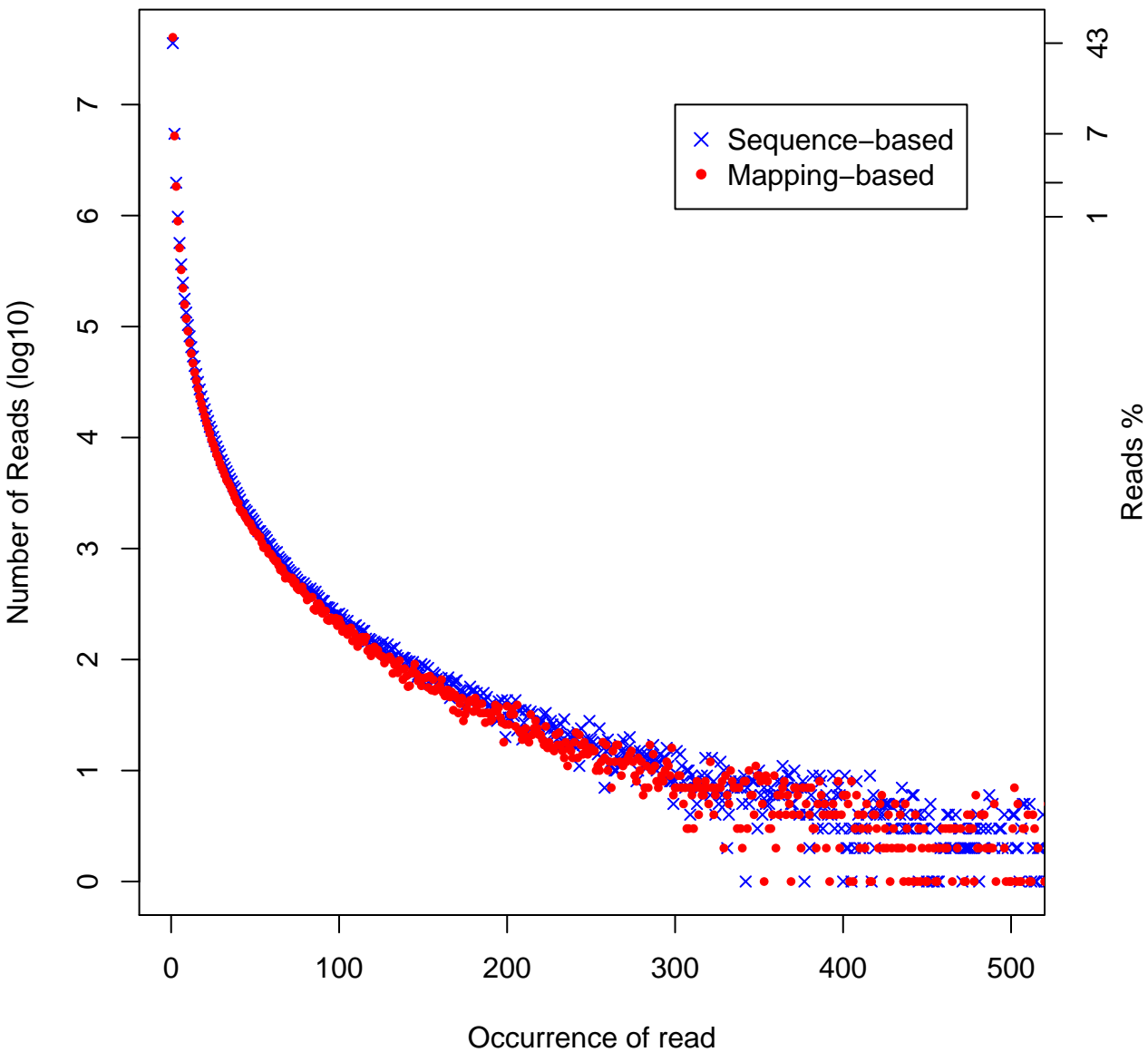

Supplement: S1 Data — This directory contains six subdirectories. The subdirectory “correlation” contains correlation heatmaps among all samples in diencephalon and telencephalon respectively. The subdirectory “mapping_stat” contains read mapping information on genomic features for each sample. The subdirectory “MDS” shows three dimensional MDS plots of the samples. The subdirectory “ReadDuplication” contains read duplication distributions for each sample. The subdirectory “ReadQuality” contains reads quality information for each sample plotted as both boxplots and heatmaps. The subdirectory “RPKMSaturation” contains information about read depth saturation for each sample as assessed by RPKM resamplings. All transcripts were divided into four quantiles based on their expression and a relative difference of observed and real RPKM values are plotted for each sample. (ZIP) [file pgen.1006840.s015.zip › RNASeq/ReadDuplication/18T_GAGATTCC-AGGCGAAG_L00M_R1_001.DupRate_plot.pdf]

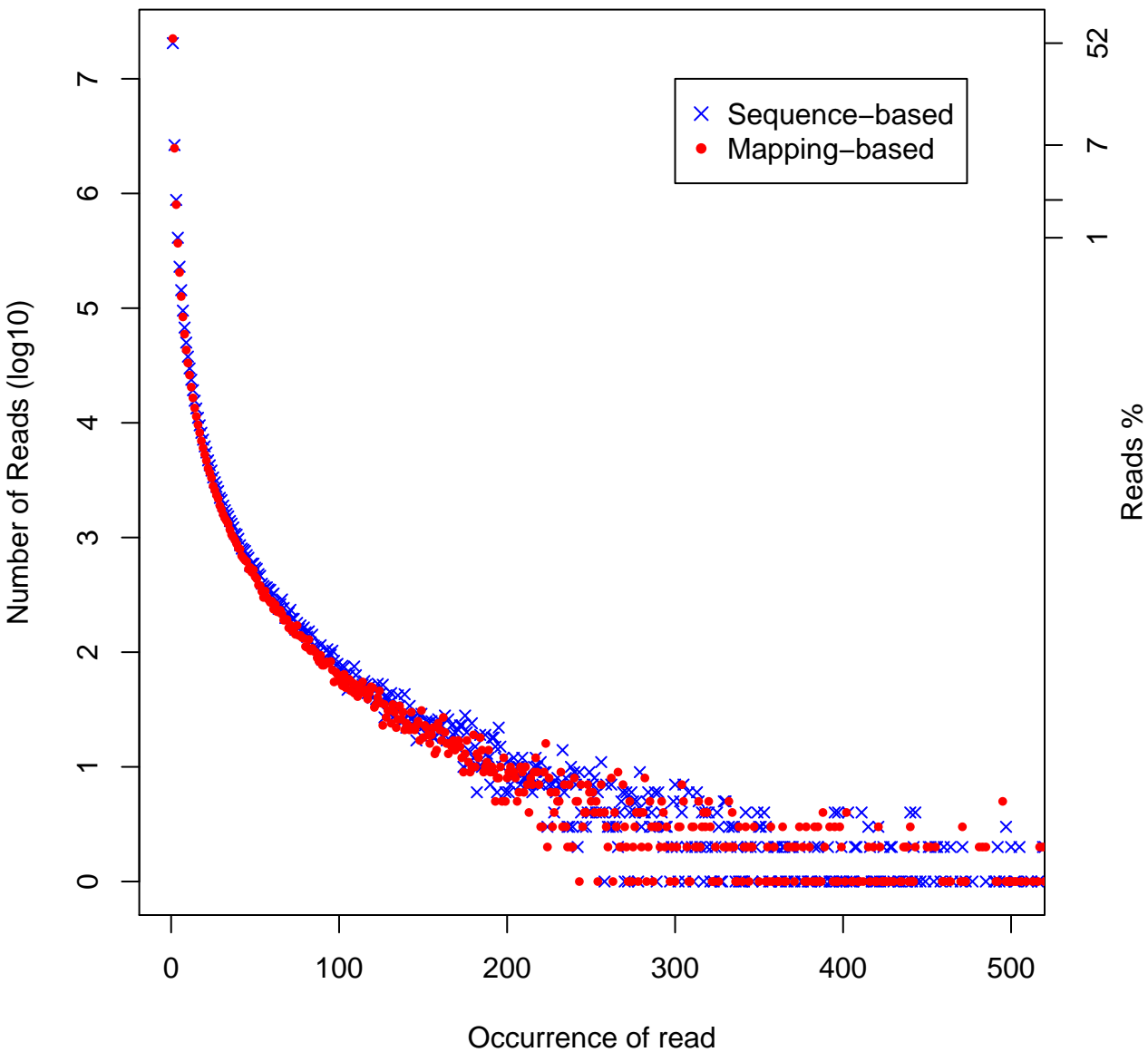

Supplement: S1 Data — This directory contains six subdirectories. The subdirectory “correlation” contains correlation heatmaps among all samples in diencephalon and telencephalon respectively. The subdirectory “mapping_stat” contains read mapping information on genomic features for each sample. The subdirectory “MDS” shows three dimensional MDS plots of the samples. The subdirectory “ReadDuplication” contains read duplication distributions for each sample. The subdirectory “ReadQuality” contains reads quality information for each sample plotted as both boxplots and heatmaps. The subdirectory “RPKMSaturation” contains information about read depth saturation for each sample as assessed by RPKM resamplings. All transcripts were divided into four quantiles based on their expression and a relative difference of observed and real RPKM values are plotted for each sample. (ZIP) [file pgen.1006840.s015.zip › RNASeq/ReadDuplication/19D_GAGATTCC-GTACTGAC_L00M_R1_001.DupRate_plot.pdf]

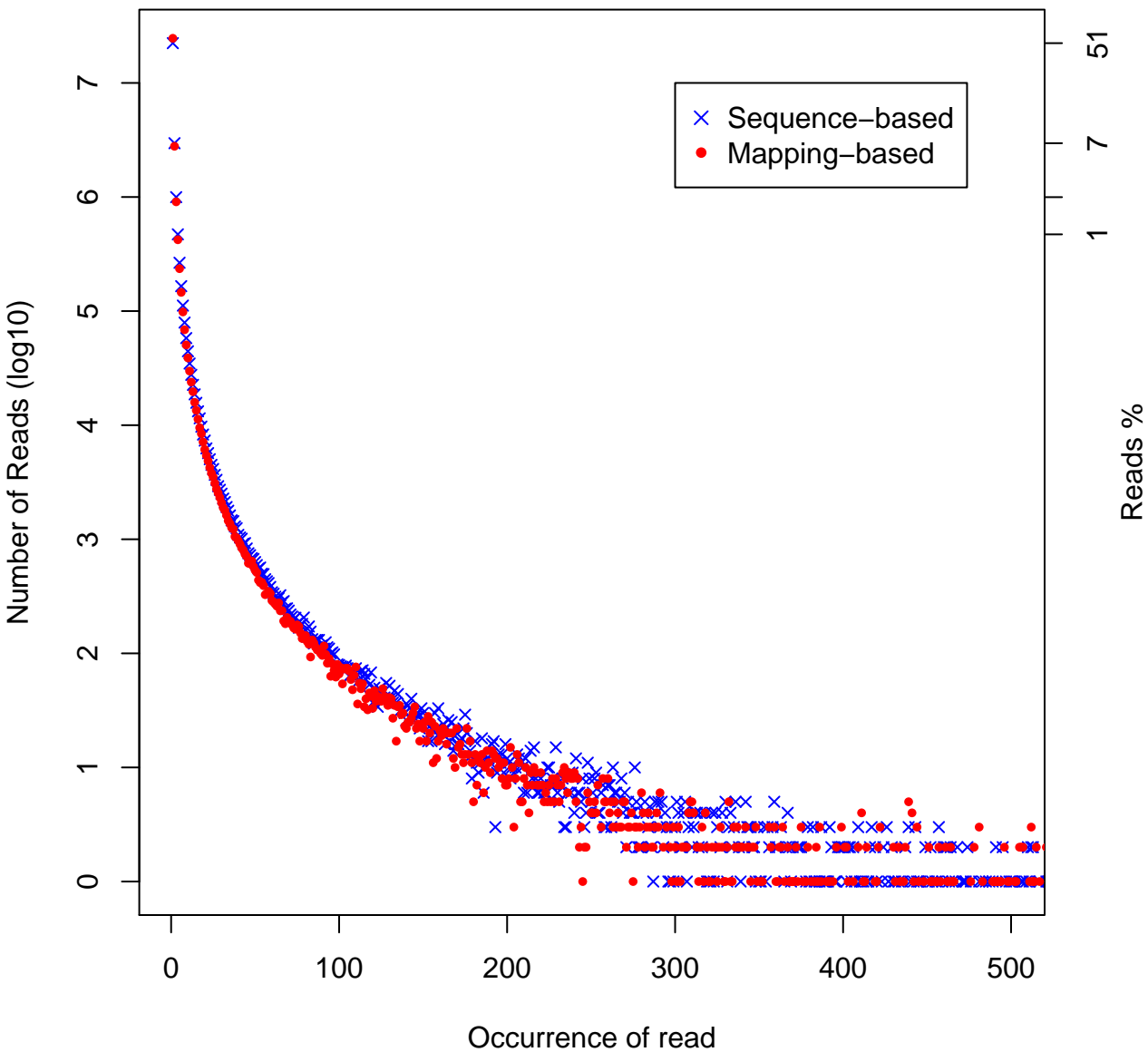

Supplement: S1 Data — This directory contains six subdirectories. The subdirectory “correlation” contains correlation heatmaps among all samples in diencephalon and telencephalon respectively. The subdirectory “mapping_stat” contains read mapping information on genomic features for each sample. The subdirectory “MDS” shows three dimensional MDS plots of the samples. The subdirectory “ReadDuplication” contains read duplication distributions for each sample. The subdirectory “ReadQuality” contains reads quality information for each sample plotted as both boxplots and heatmaps. The subdirectory “RPKMSaturation” contains information about read depth saturation for each sample as assessed by RPKM resamplings. All transcripts were divided into four quantiles based on their expression and a relative difference of observed and real RPKM values are plotted for each sample. (ZIP) [file pgen.1006840.s015.zip › RNASeq/ReadDuplication/19T_GAGATTCC-CAGGACGT_L00M_R1_001.DupRate_plot.pdf]

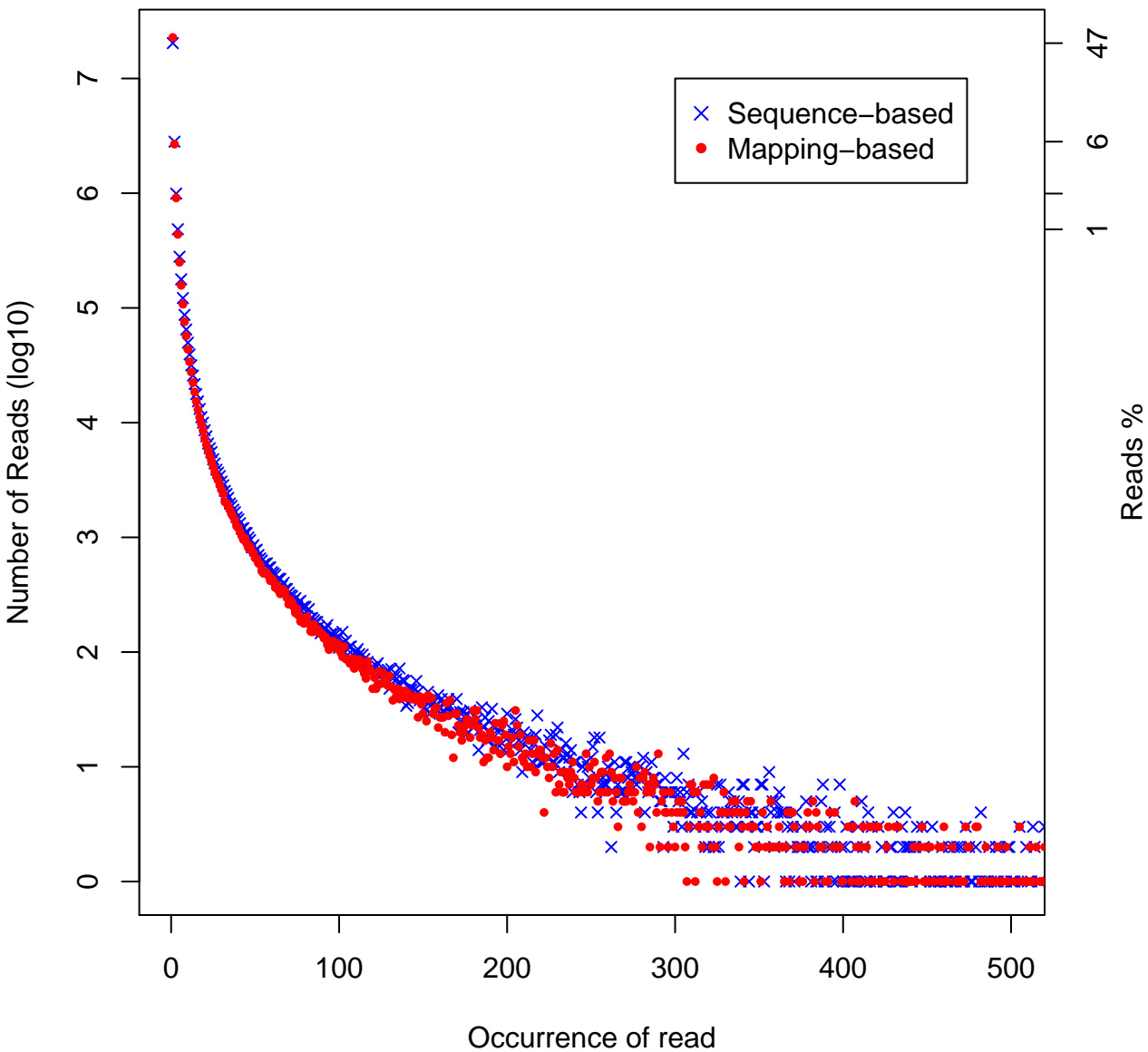

Supplement: S1 Data — This directory contains six subdirectories. The subdirectory “correlation” contains correlation heatmaps among all samples in diencephalon and telencephalon respectively. The subdirectory “mapping_stat” contains read mapping information on genomic features for each sample. The subdirectory “MDS” shows three dimensional MDS plots of the samples. The subdirectory “ReadDuplication” contains read duplication distributions for each sample. The subdirectory “ReadQuality” contains reads quality information for each sample plotted as both boxplots and heatmaps. The subdirectory “RPKMSaturation” contains information about read depth saturation for each sample as assessed by RPKM resamplings. All transcripts were divided into four quantiles based on their expression and a relative difference of observed and real RPKM values are plotted for each sample. (ZIP) [file pgen.1006840.s015.zip › RNASeq/ReadDuplication/1D_ATTACTCG-ATAGAGGC_L00M_R1_001.DupRate_plot.pdf]

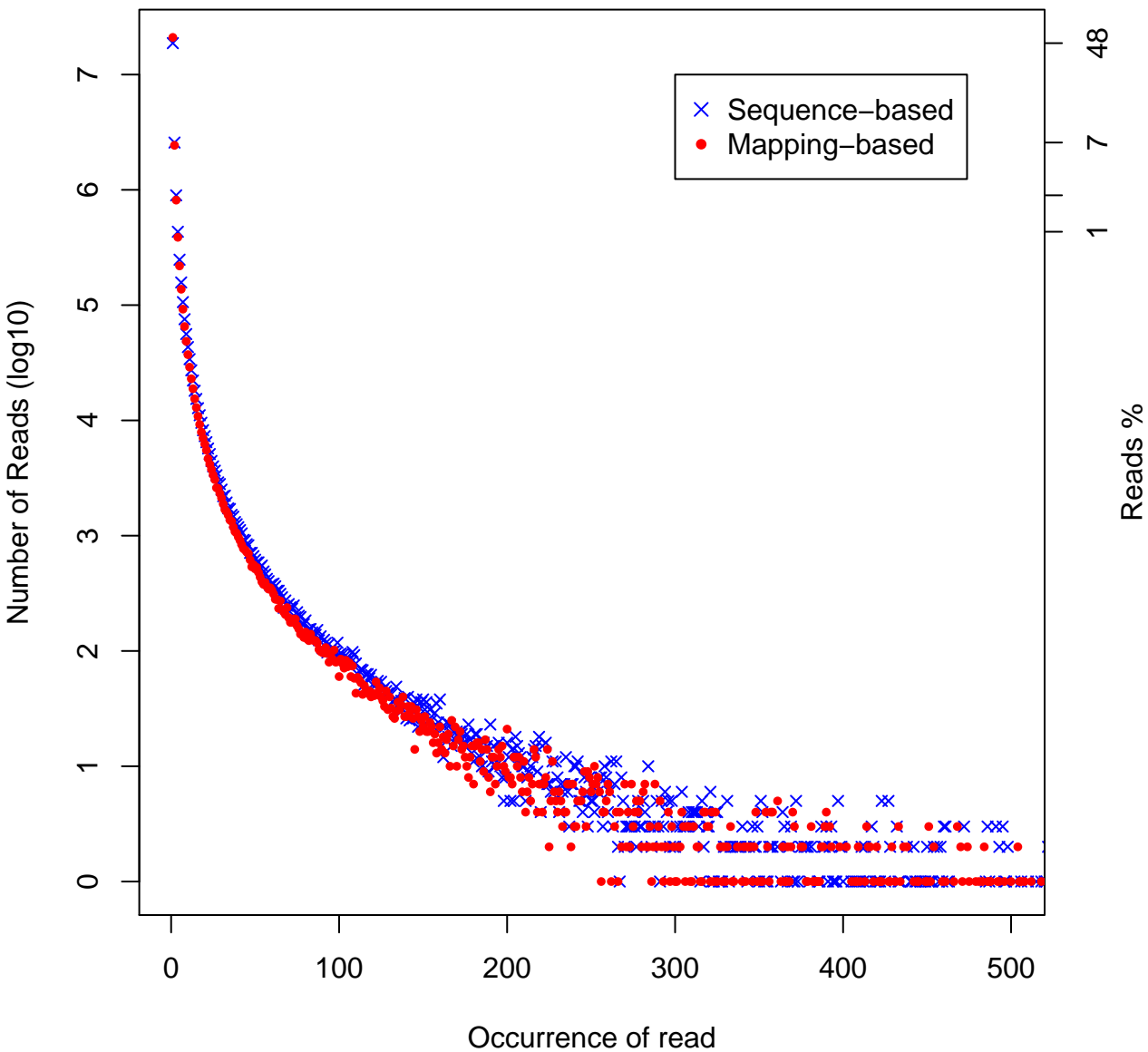

Supplement: S1 Data — This directory contains six subdirectories. The subdirectory “correlation” contains correlation heatmaps among all samples in diencephalon and telencephalon respectively. The subdirectory “mapping_stat” contains read mapping information on genomic features for each sample. The subdirectory “MDS” shows three dimensional MDS plots of the samples. The subdirectory “ReadDuplication” contains read duplication distributions for each sample. The subdirectory “ReadQuality” contains reads quality information for each sample plotted as both boxplots and heatmaps. The subdirectory “RPKMSaturation” contains information about read depth saturation for each sample as assessed by RPKM resamplings. All transcripts were divided into four quantiles based on their expression and a relative difference of observed and real RPKM values are plotted for each sample. (ZIP) [file pgen.1006840.s015.zip › RNASeq/ReadDuplication/1T_ATTACTCG-TATAGCCT_L00M_R1_001.DupRate_plot.pdf]

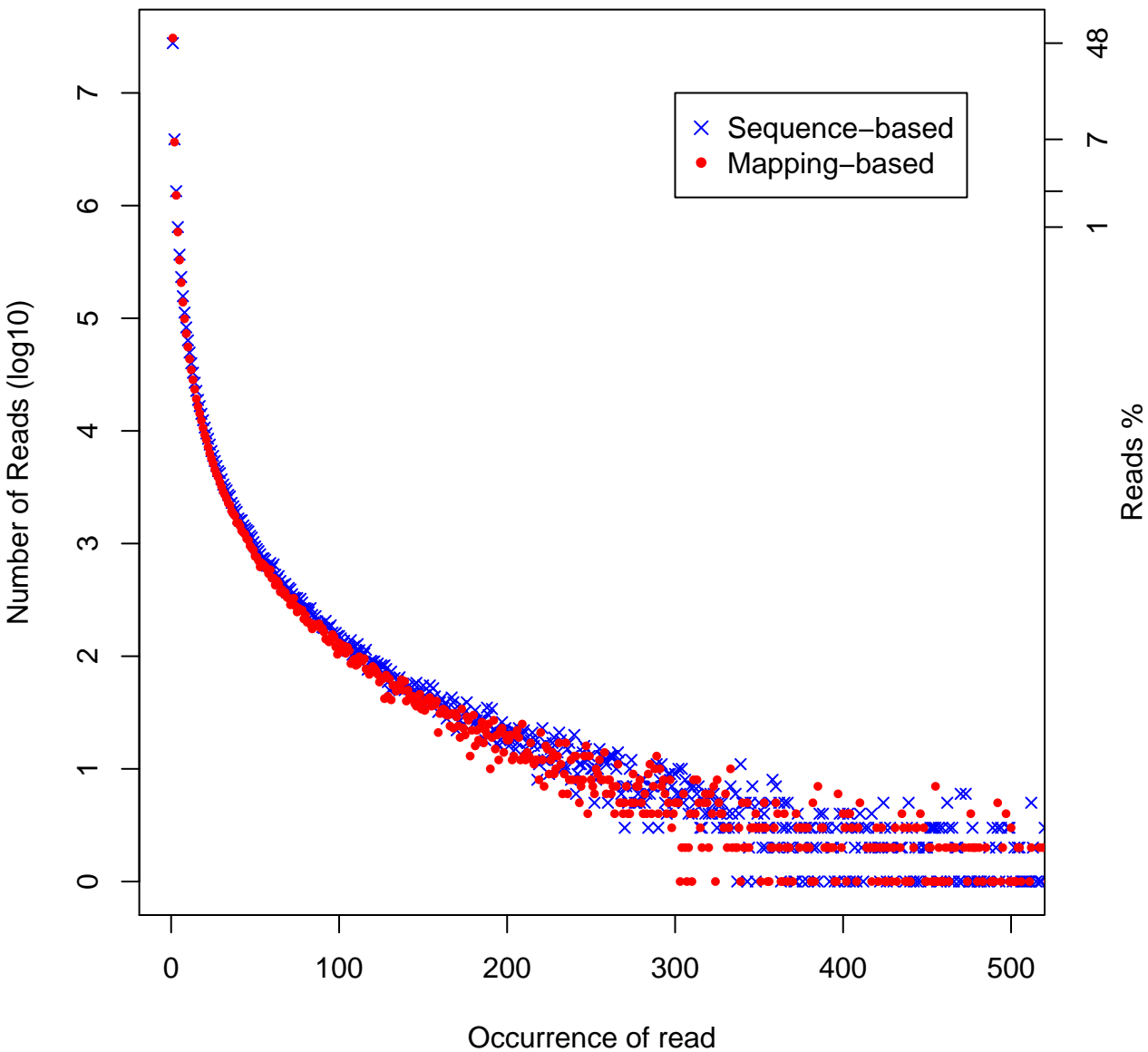

Supplement: S1 Data — This directory contains six subdirectories. The subdirectory “correlation” contains correlation heatmaps among all samples in diencephalon and telencephalon respectively. The subdirectory “mapping_stat” contains read mapping information on genomic features for each sample. The subdirectory “MDS” shows three dimensional MDS plots of the samples. The subdirectory “ReadDuplication” contains read duplication distributions for each sample. The subdirectory “ReadQuality” contains reads quality information for each sample plotted as both boxplots and heatmaps. The subdirectory “RPKMSaturation” contains information about read depth saturation for each sample as assessed by RPKM resamplings. All transcripts were divided into four quantiles based on their expression and a relative difference of observed and real RPKM values are plotted for each sample. (ZIP) [file pgen.1006840.s015.zip › RNASeq/ReadDuplication/20D_ATTCAGAA-ATAGAGGC_L00M_R1_001.DupRate_plot.pdf]

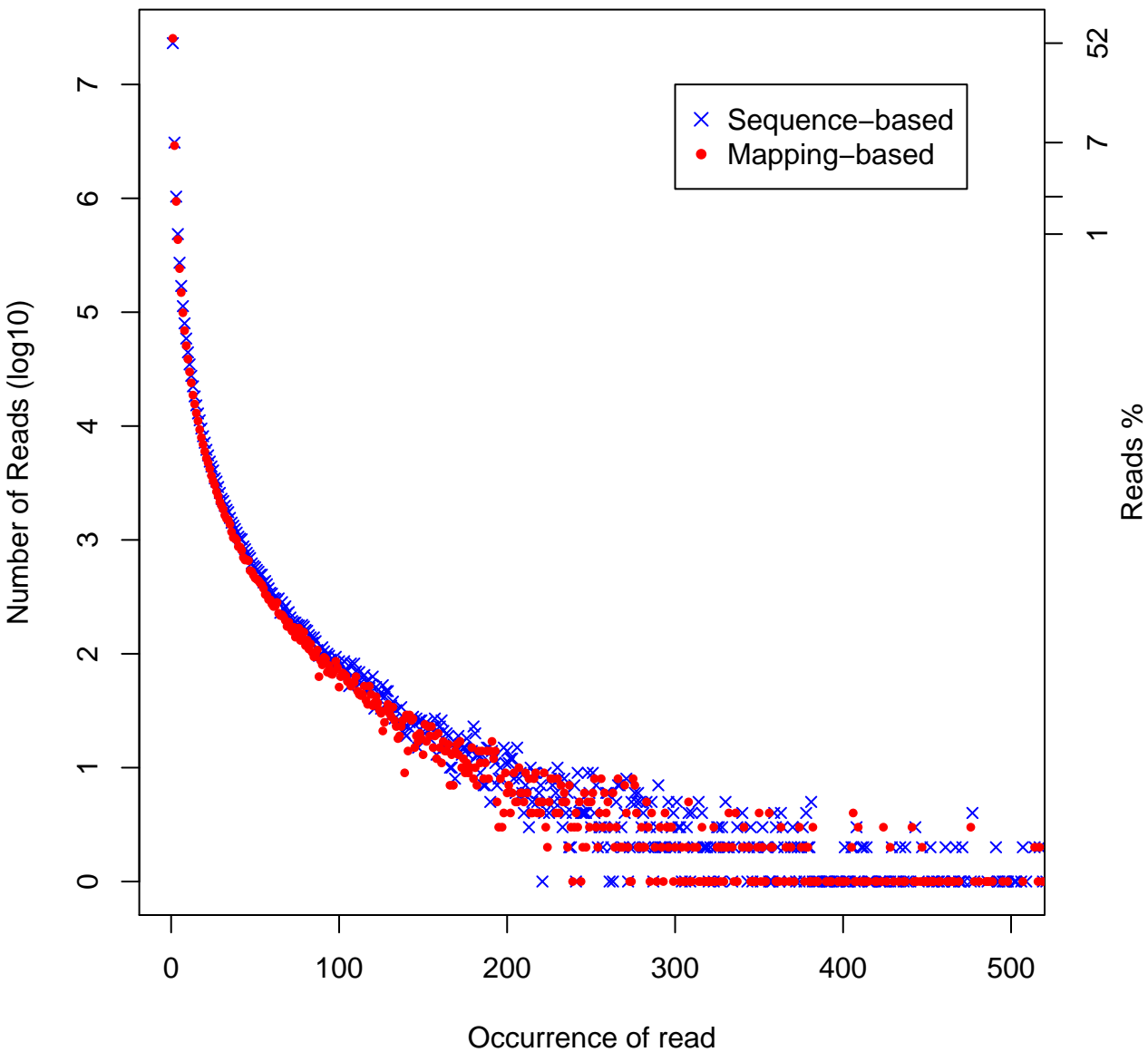

Supplement: S1 Data — This directory contains six subdirectories. The subdirectory “correlation” contains correlation heatmaps among all samples in diencephalon and telencephalon respectively. The subdirectory “mapping_stat” contains read mapping information on genomic features for each sample. The subdirectory “MDS” shows three dimensional MDS plots of the samples. The subdirectory “ReadDuplication” contains read duplication distributions for each sample. The subdirectory “ReadQuality” contains reads quality information for each sample plotted as both boxplots and heatmaps. The subdirectory “RPKMSaturation” contains information about read depth saturation for each sample as assessed by RPKM resamplings. All transcripts were divided into four quantiles based on their expression and a relative difference of observed and real RPKM values are plotted for each sample. (ZIP) [file pgen.1006840.s015.zip › RNASeq/ReadDuplication/20T_ATTCAGAA-TATAGCCT_L00M_R1_001.DupRate_plot.pdf]

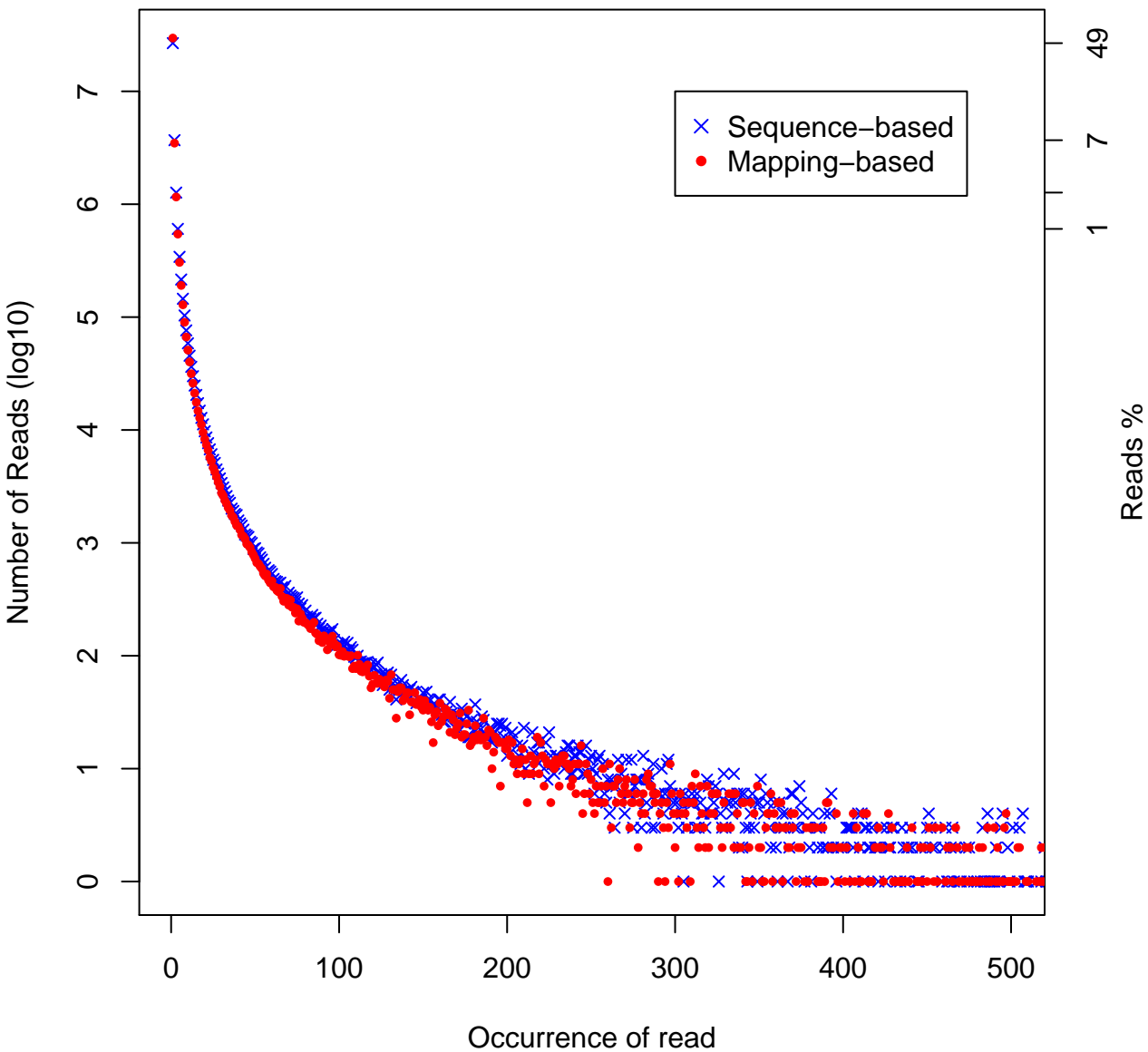

Supplement: S1 Data — This directory contains six subdirectories. The subdirectory “correlation” contains correlation heatmaps among all samples in diencephalon and telencephalon respectively. The subdirectory “mapping_stat” contains read mapping information on genomic features for each sample. The subdirectory “MDS” shows three dimensional MDS plots of the samples. The subdirectory “ReadDuplication” contains read duplication distributions for each sample. The subdirectory “ReadQuality” contains reads quality information for each sample plotted as both boxplots and heatmaps. The subdirectory “RPKMSaturation” contains information about read depth saturation for each sample as assessed by RPKM resamplings. All transcripts were divided into four quantiles based on their expression and a relative difference of observed and real RPKM values are plotted for each sample. (ZIP) [file pgen.1006840.s015.zip › RNASeq/ReadDuplication/21D_ATTCAGAA-GGCTCTGA_L00M_R1_001.DupRate_plot.pdf]

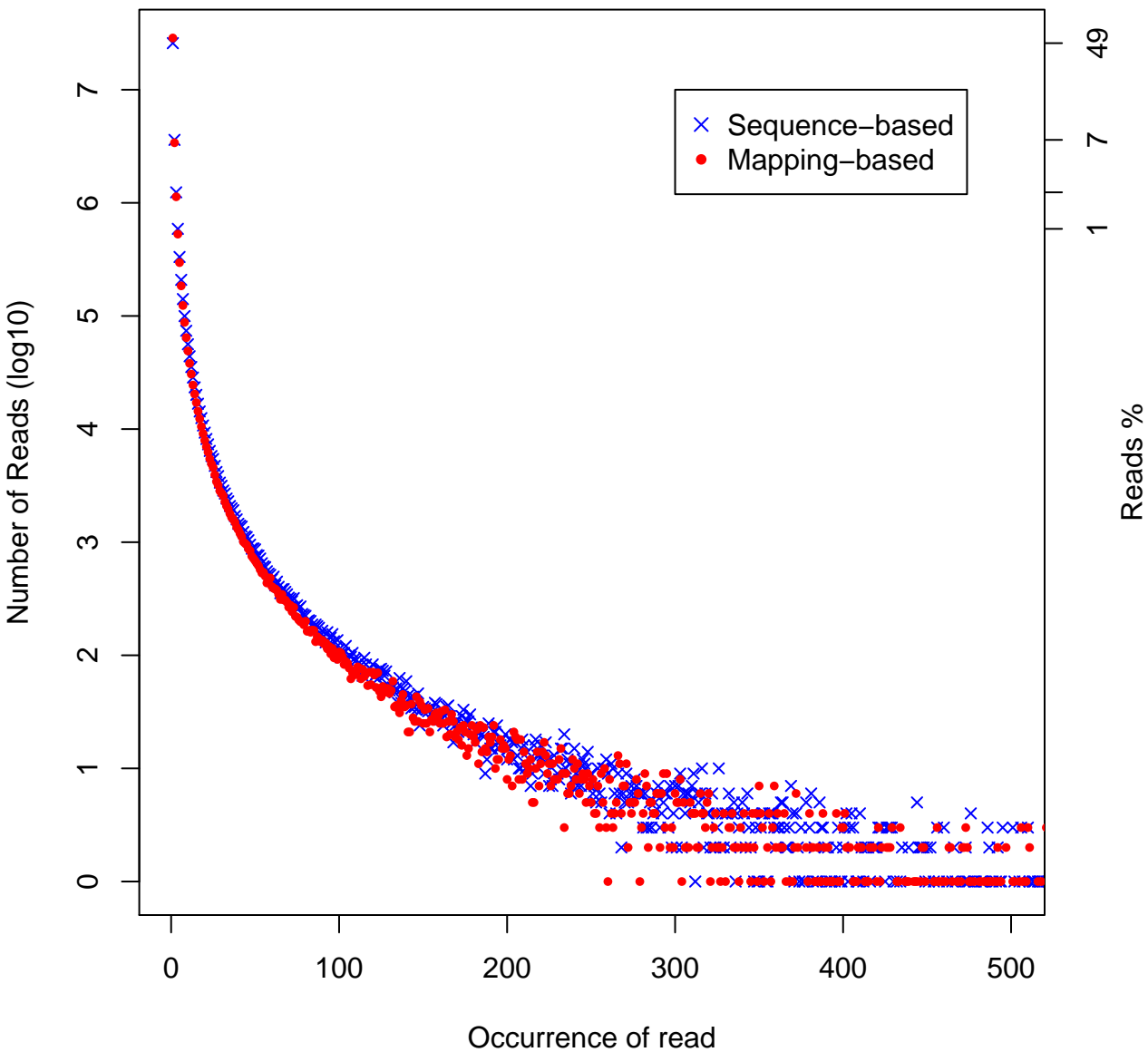

Supplement: S1 Data — This directory contains six subdirectories. The subdirectory “correlation” contains correlation heatmaps among all samples in diencephalon and telencephalon respectively. The subdirectory “mapping_stat” contains read mapping information on genomic features for each sample. The subdirectory “MDS” shows three dimensional MDS plots of the samples. The subdirectory “ReadDuplication” contains read duplication distributions for each sample. The subdirectory “ReadQuality” contains reads quality information for each sample plotted as both boxplots and heatmaps. The subdirectory “RPKMSaturation” contains information about read depth saturation for each sample as assessed by RPKM resamplings. All transcripts were divided into four quantiles based on their expression and a relative difference of observed and real RPKM values are plotted for each sample. (ZIP) [file pgen.1006840.s015.zip › RNASeq/ReadDuplication/21T_ATTCAGAA-CCTATCCT_L00M_R1_001.DupRate_plot.pdf]

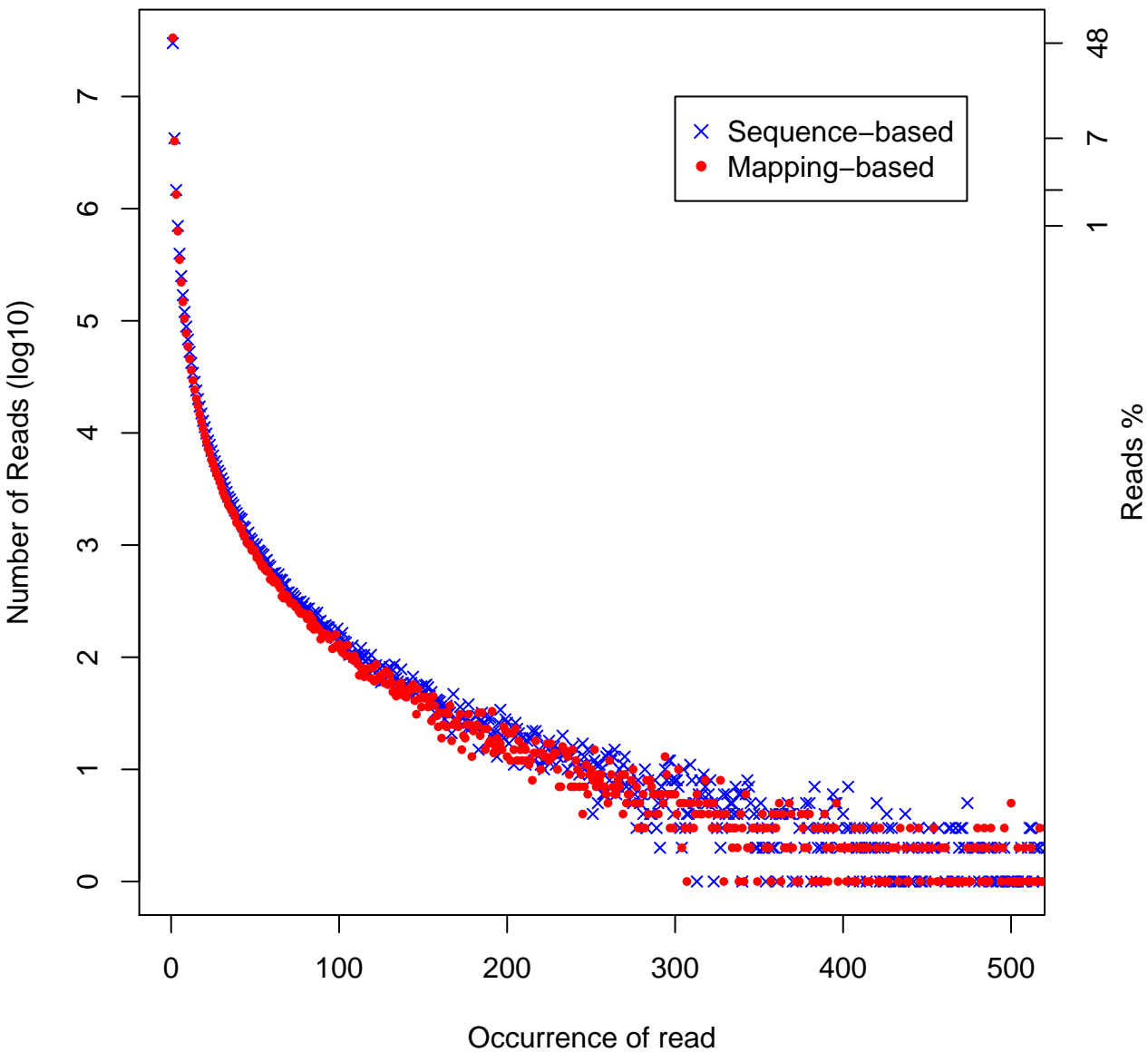

Supplement: S1 Data — This directory contains six subdirectories. The subdirectory “correlation” contains correlation heatmaps among all samples in diencephalon and telencephalon respectively. The subdirectory “mapping_stat” contains read mapping information on genomic features for each sample. The subdirectory “MDS” shows three dimensional MDS plots of the samples. The subdirectory “ReadDuplication” contains read duplication distributions for each sample. The subdirectory “ReadQuality” contains reads quality information for each sample plotted as both boxplots and heatmaps. The subdirectory “RPKMSaturation” contains information about read depth saturation for each sample as assessed by RPKM resamplings. All transcripts were divided into four quantiles based on their expression and a relative difference of observed and real RPKM values are plotted for each sample. (ZIP) [file pgen.1006840.s015.zip › RNASeq/ReadDuplication/23D_ATTCAGAA-TAATCTTA_L00M_R1_001.DupRate_plot.pdf]

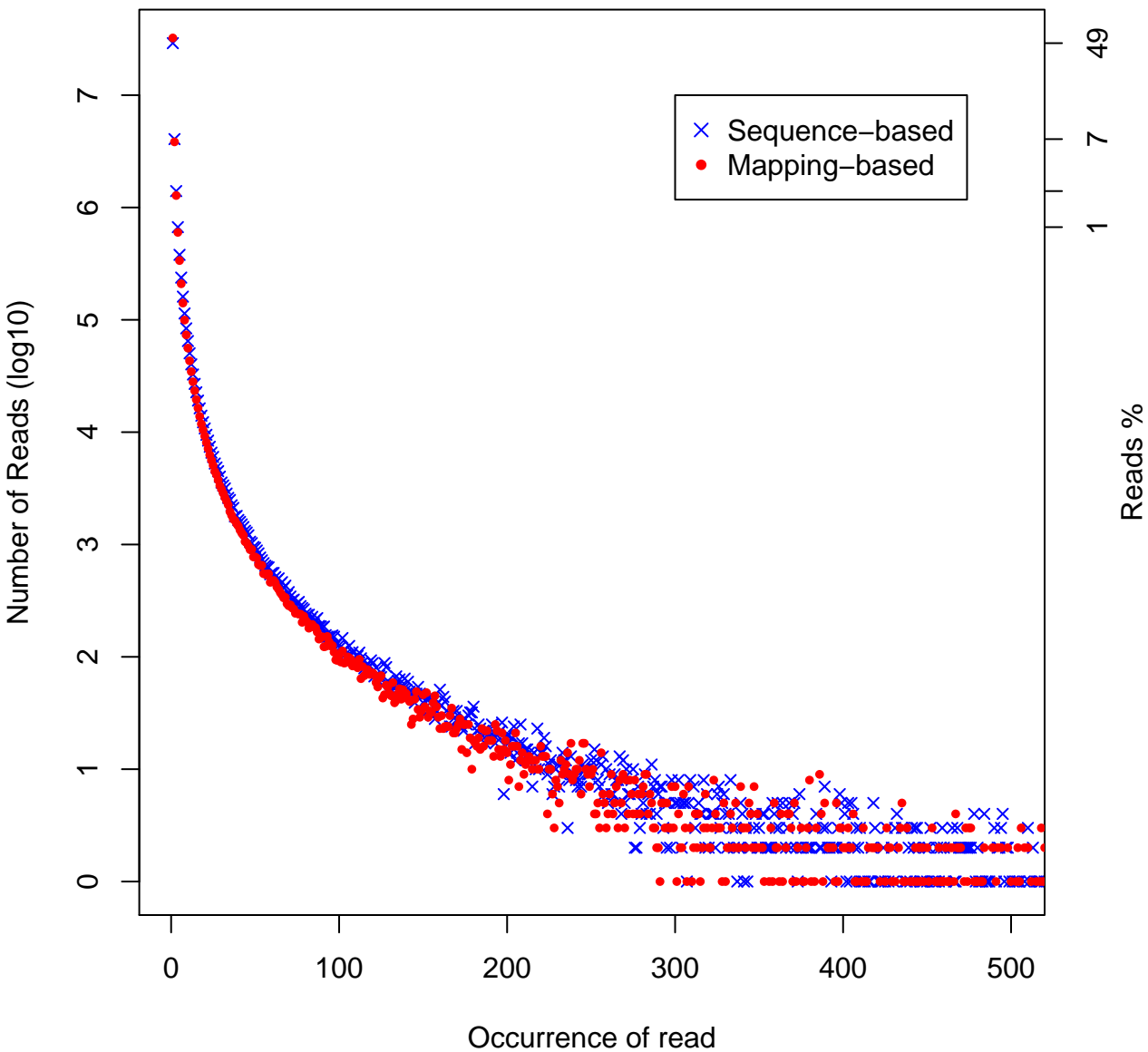

Supplement: S1 Data — This directory contains six subdirectories. The subdirectory “correlation” contains correlation heatmaps among all samples in diencephalon and telencephalon respectively. The subdirectory “mapping_stat” contains read mapping information on genomic features for each sample. The subdirectory “MDS” shows three dimensional MDS plots of the samples. The subdirectory “ReadDuplication” contains read duplication distributions for each sample. The subdirectory “ReadQuality” contains reads quality information for each sample plotted as both boxplots and heatmaps. The subdirectory “RPKMSaturation” contains information about read depth saturation for each sample as assessed by RPKM resamplings. All transcripts were divided into four quantiles based on their expression and a relative difference of observed and real RPKM values are plotted for each sample. (ZIP) [file pgen.1006840.s015.zip › RNASeq/ReadDuplication/23T_ATTCAGAA-AGGCGAAG_L00M_R1_001.DupRate_plot.pdf]

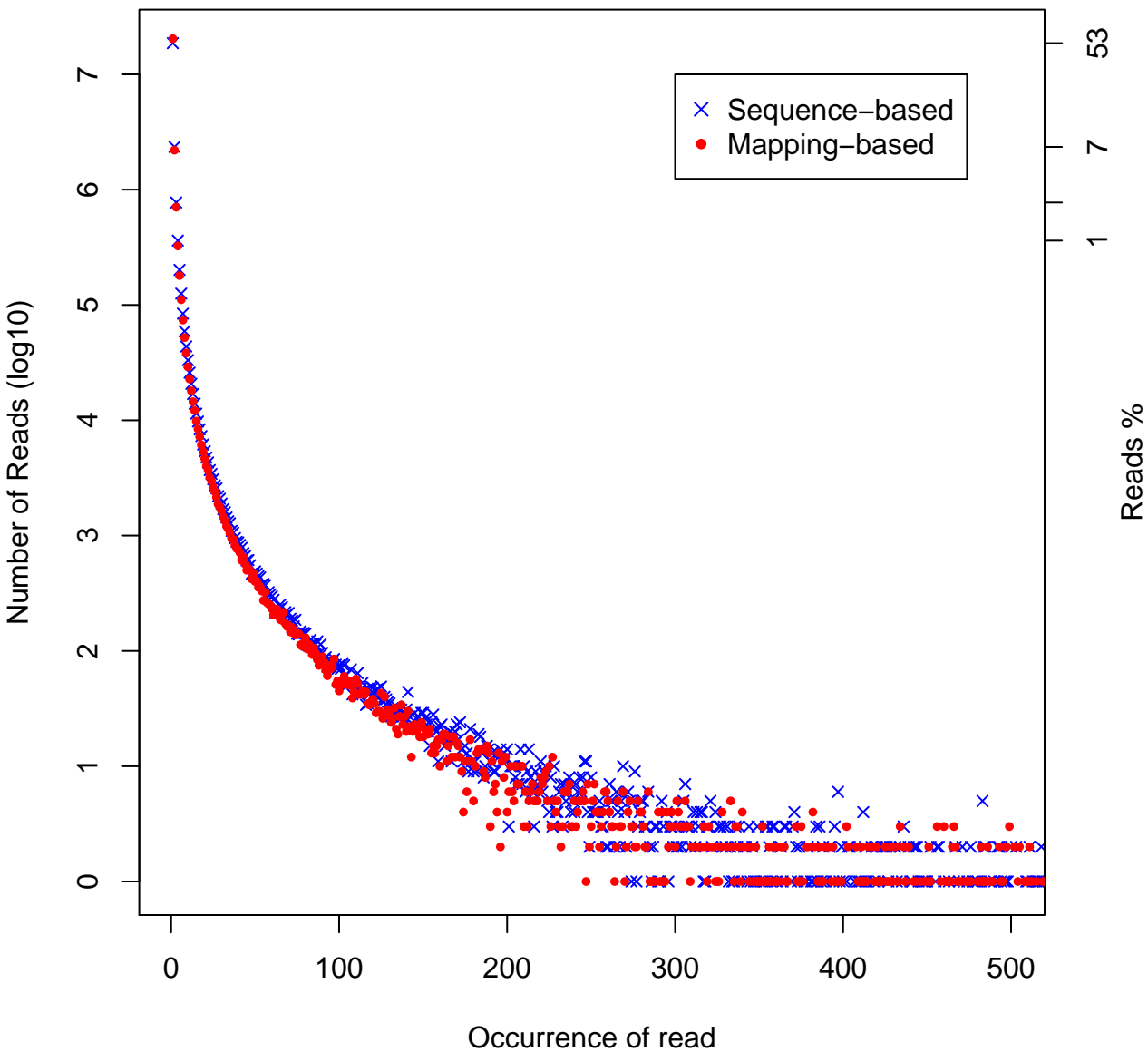

Supplement: S1 Data — This directory contains six subdirectories. The subdirectory “correlation” contains correlation heatmaps among all samples in diencephalon and telencephalon respectively. The subdirectory “mapping_stat” contains read mapping information on genomic features for each sample. The subdirectory “MDS” shows three dimensional MDS plots of the samples. The subdirectory “ReadDuplication” contains read duplication distributions for each sample. The subdirectory “ReadQuality” contains reads quality information for each sample plotted as both boxplots and heatmaps. The subdirectory “RPKMSaturation” contains information about read depth saturation for each sample as assessed by RPKM resamplings. All transcripts were divided into four quantiles based on their expression and a relative difference of observed and real RPKM values are plotted for each sample. (ZIP) [file pgen.1006840.s015.zip › RNASeq/ReadDuplication/25D_ATTCAGAA-GTACTGAC_L00M_R1_001.DupRate_plot.pdf]

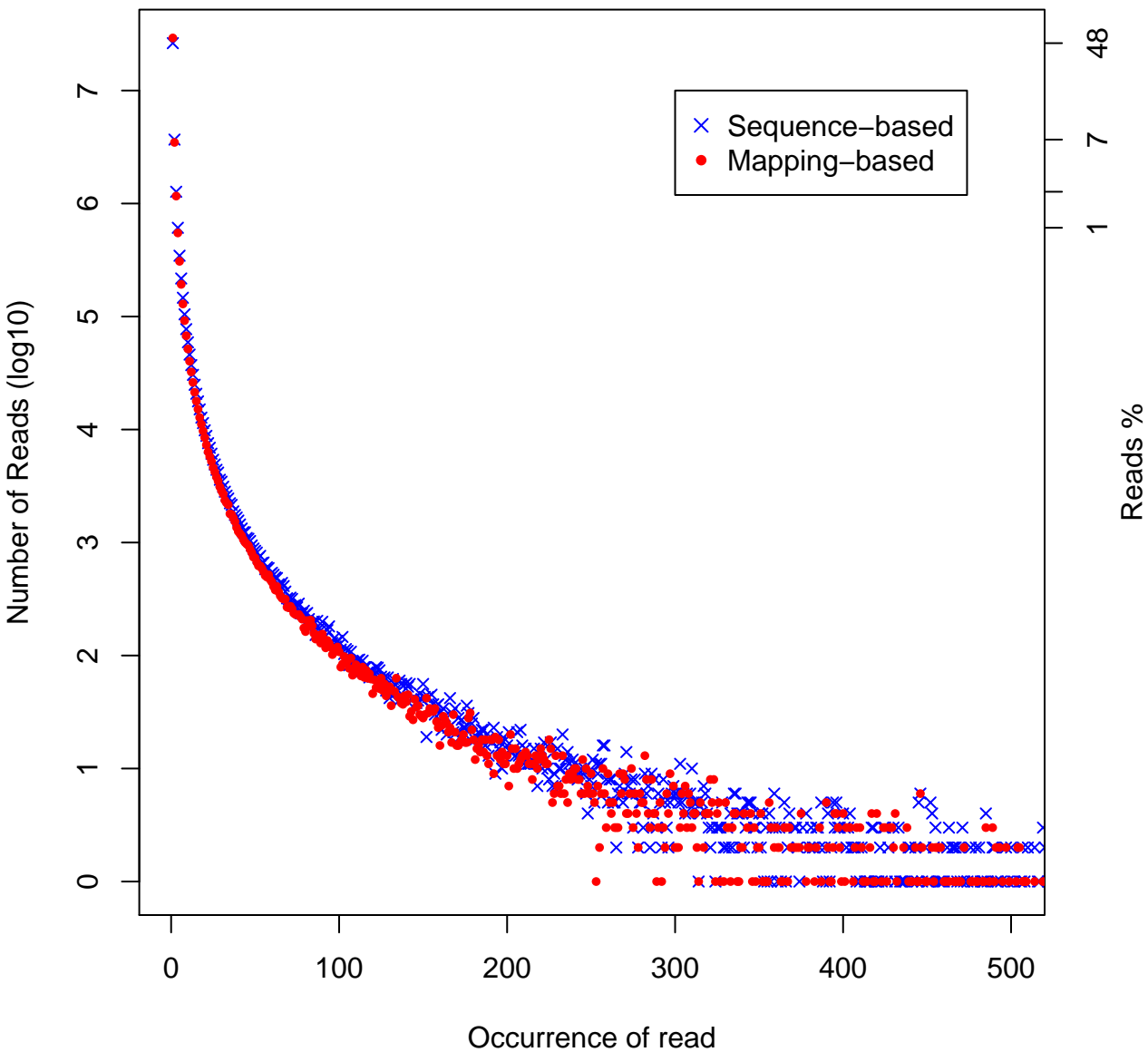

Supplement: S1 Data — This directory contains six subdirectories. The subdirectory “correlation” contains correlation heatmaps among all samples in diencephalon and telencephalon respectively. The subdirectory “mapping_stat” contains read mapping information on genomic features for each sample. The subdirectory “MDS” shows three dimensional MDS plots of the samples. The subdirectory “ReadDuplication” contains read duplication distributions for each sample. The subdirectory “ReadQuality” contains reads quality information for each sample plotted as both boxplots and heatmaps. The subdirectory “RPKMSaturation” contains information about read depth saturation for each sample as assessed by RPKM resamplings. All transcripts were divided into four quantiles based on their expression and a relative difference of observed and real RPKM values are plotted for each sample. (ZIP) [file pgen.1006840.s015.zip › RNASeq/ReadDuplication/25T_ATTCAGAA-CAGGACGT_L00M_R1_001.DupRate_plot.pdf]

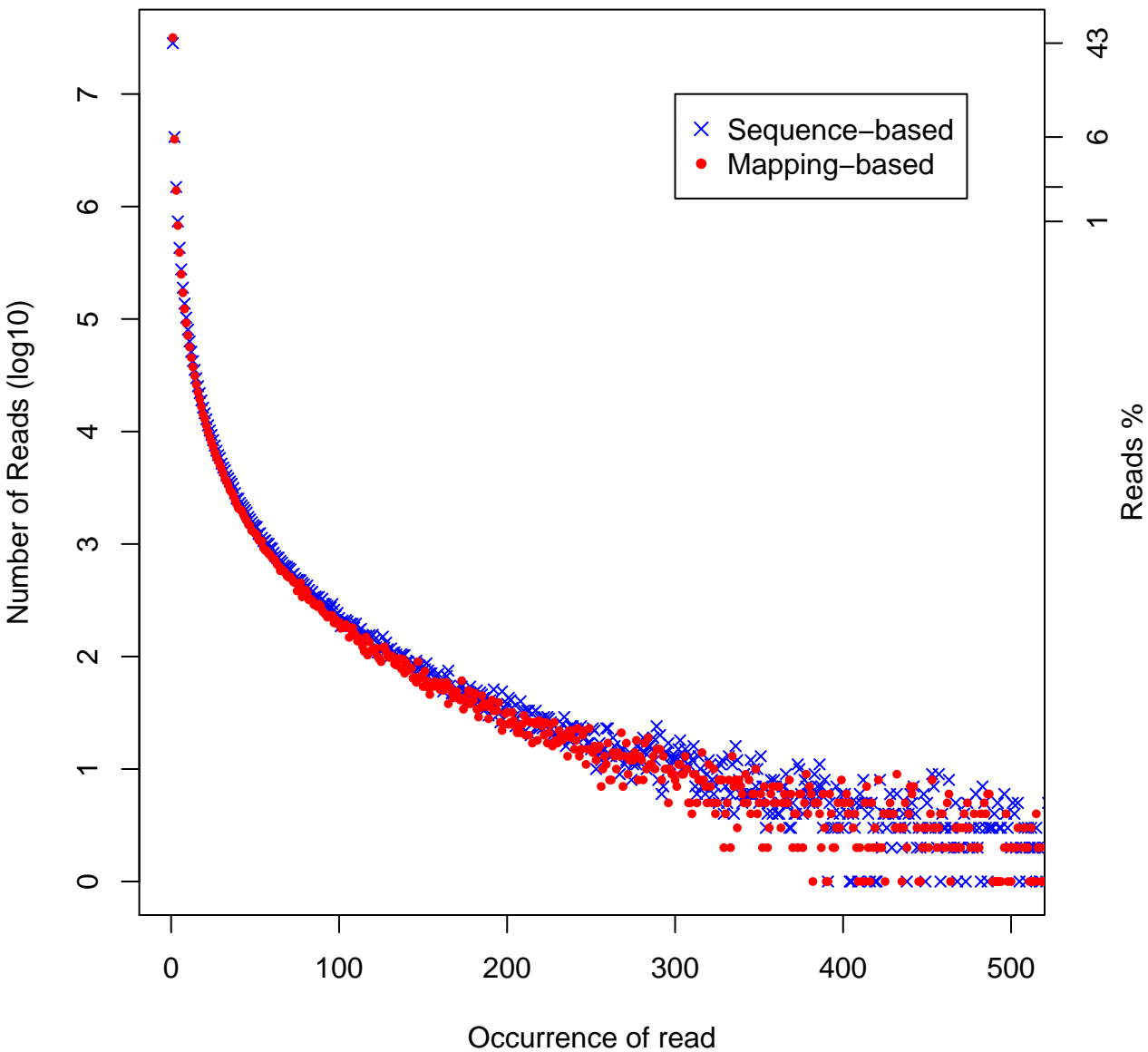

Supplement: S1 Data — This directory contains six subdirectories. The subdirectory “correlation” contains correlation heatmaps among all samples in diencephalon and telencephalon respectively. The subdirectory “mapping_stat” contains read mapping information on genomic features for each sample. The subdirectory “MDS” shows three dimensional MDS plots of the samples. The subdirectory “ReadDuplication” contains read duplication distributions for each sample. The subdirectory “ReadQuality” contains reads quality information for each sample plotted as both boxplots and heatmaps. The subdirectory “RPKMSaturation” contains information about read depth saturation for each sample as assessed by RPKM resamplings. All transcripts were divided into four quantiles based on their expression and a relative difference of observed and real RPKM values are plotted for each sample. (ZIP) [file pgen.1006840.s015.zip › RNASeq/ReadDuplication/27D_GAATTCGT-ATAGAGGC_L00M_R1_001.DupRate_plot.pdf]

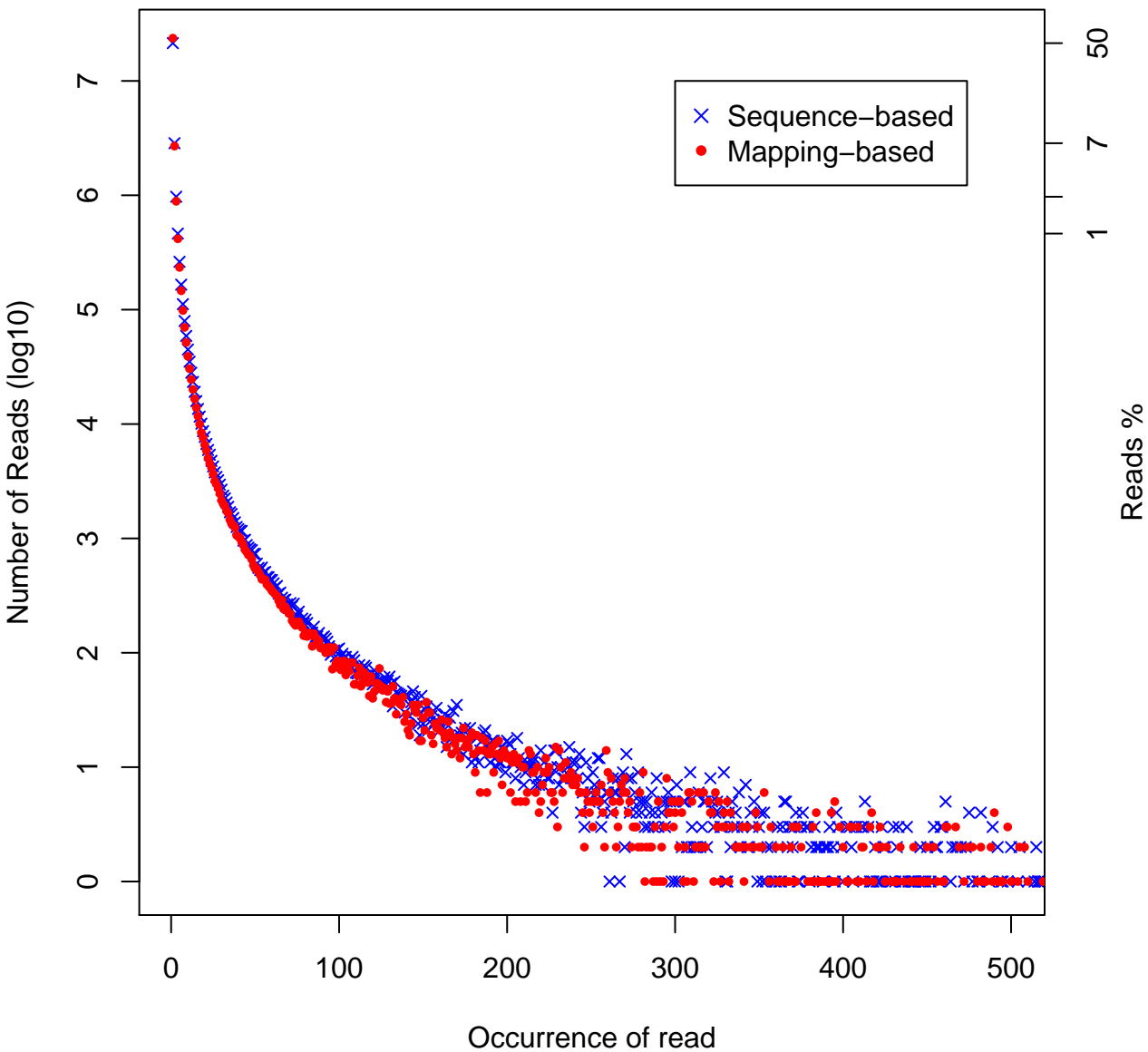

Supplement: S1 Data — This directory contains six subdirectories. The subdirectory “correlation” contains correlation heatmaps among all samples in diencephalon and telencephalon respectively. The subdirectory “mapping_stat” contains read mapping information on genomic features for each sample. The subdirectory “MDS” shows three dimensional MDS plots of the samples. The subdirectory “ReadDuplication” contains read duplication distributions for each sample. The subdirectory “ReadQuality” contains reads quality information for each sample plotted as both boxplots and heatmaps. The subdirectory “RPKMSaturation” contains information about read depth saturation for each sample as assessed by RPKM resamplings. All transcripts were divided into four quantiles based on their expression and a relative difference of observed and real RPKM values are plotted for each sample. (ZIP) [file pgen.1006840.s015.zip › RNASeq/ReadDuplication/27T_GAATTCGT-TATAGCCT_L00M_R1_001.DupRate_plot.pdf]

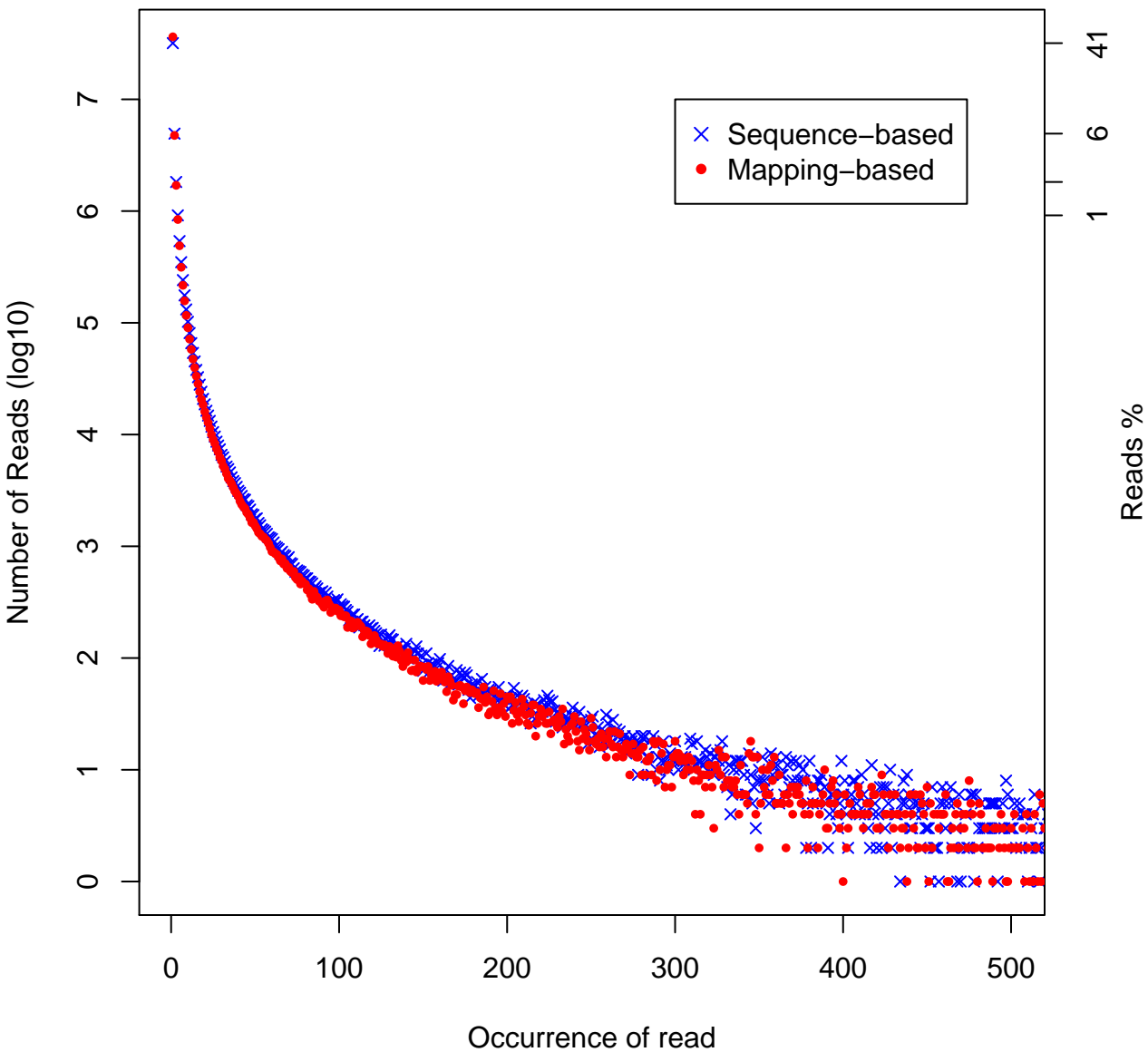

Supplement: S1 Data — This directory contains six subdirectories. The subdirectory “correlation” contains correlation heatmaps among all samples in diencephalon and telencephalon respectively. The subdirectory “mapping_stat” contains read mapping information on genomic features for each sample. The subdirectory “MDS” shows three dimensional MDS plots of the samples. The subdirectory “ReadDuplication” contains read duplication distributions for each sample. The subdirectory “ReadQuality” contains reads quality information for each sample plotted as both boxplots and heatmaps. The subdirectory “RPKMSaturation” contains information about read depth saturation for each sample as assessed by RPKM resamplings. All transcripts were divided into four quantiles based on their expression and a relative difference of observed and real RPKM values are plotted for each sample. (ZIP) [file pgen.1006840.s015.zip › RNASeq/ReadDuplication/28D_GAATTCGT-GGCTCTGA_L00M_R1_001.DupRate_plot.pdf]

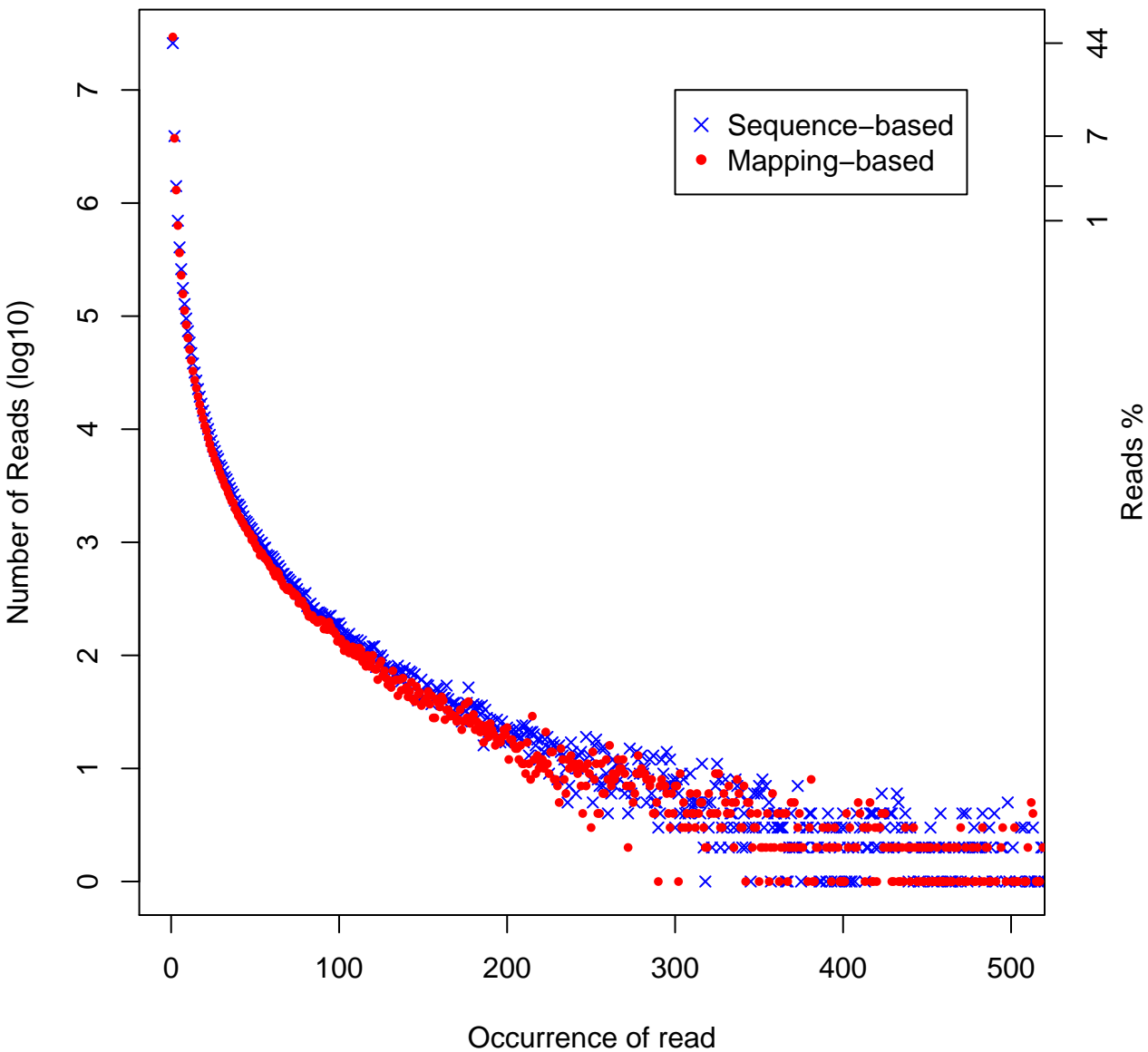

Supplement: S1 Data — This directory contains six subdirectories. The subdirectory “correlation” contains correlation heatmaps among all samples in diencephalon and telencephalon respectively. The subdirectory “mapping_stat” contains read mapping information on genomic features for each sample. The subdirectory “MDS” shows three dimensional MDS plots of the samples. The subdirectory “ReadDuplication” contains read duplication distributions for each sample. The subdirectory “ReadQuality” contains reads quality information for each sample plotted as both boxplots and heatmaps. The subdirectory “RPKMSaturation” contains information about read depth saturation for each sample as assessed by RPKM resamplings. All transcripts were divided into four quantiles based on their expression and a relative difference of observed and real RPKM values are plotted for each sample. (ZIP) [file pgen.1006840.s015.zip › RNASeq/ReadDuplication/28T_GAATTCGT-CCTATCCT_L00M_R1_001.DupRate_plot.pdf]

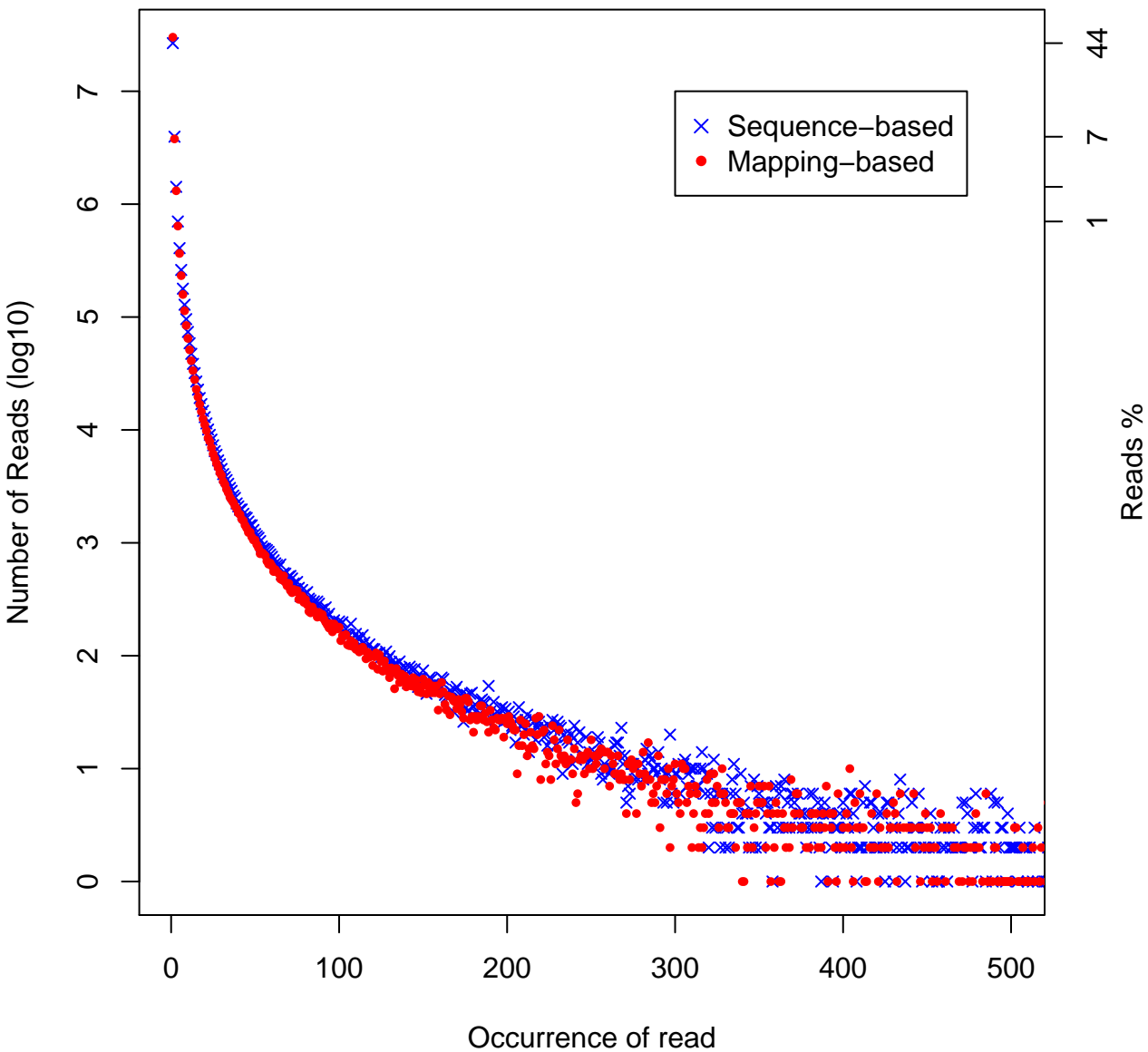

Supplement: S1 Data — This directory contains six subdirectories. The subdirectory “correlation” contains correlation heatmaps among all samples in diencephalon and telencephalon respectively. The subdirectory “mapping_stat” contains read mapping information on genomic features for each sample. The subdirectory “MDS” shows three dimensional MDS plots of the samples. The subdirectory “ReadDuplication” contains read duplication distributions for each sample. The subdirectory “ReadQuality” contains reads quality information for each sample plotted as both boxplots and heatmaps. The subdirectory “RPKMSaturation” contains information about read depth saturation for each sample as assessed by RPKM resamplings. All transcripts were divided into four quantiles based on their expression and a relative difference of observed and real RPKM values are plotted for each sample. (ZIP) [file pgen.1006840.s015.zip › RNASeq/ReadDuplication/29D_GAATTCGT-TAATCTTA_L00M_R1_001.DupRate_plot.pdf]

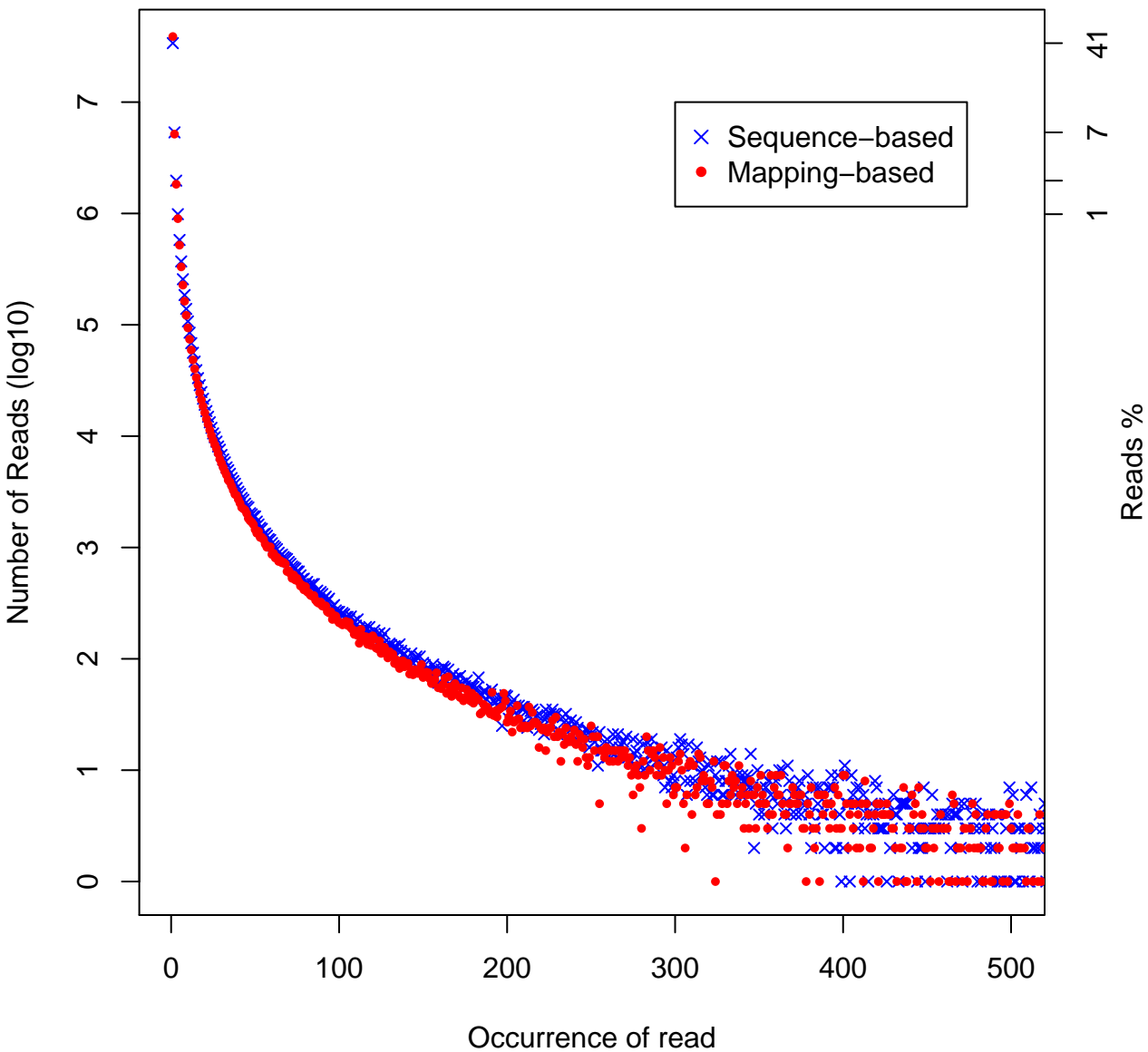

Supplement: S1 Data — This directory contains six subdirectories. The subdirectory “correlation” contains correlation heatmaps among all samples in diencephalon and telencephalon respectively. The subdirectory “mapping_stat” contains read mapping information on genomic features for each sample. The subdirectory “MDS” shows three dimensional MDS plots of the samples. The subdirectory “ReadDuplication” contains read duplication distributions for each sample. The subdirectory “ReadQuality” contains reads quality information for each sample plotted as both boxplots and heatmaps. The subdirectory “RPKMSaturation” contains information about read depth saturation for each sample as assessed by RPKM resamplings. All transcripts were divided into four quantiles based on their expression and a relative difference of observed and real RPKM values are plotted for each sample. (ZIP) [file pgen.1006840.s015.zip › RNASeq/ReadDuplication/29T_GAATTCGT-AGGCGAAG_L00M_R1_001.DupRate_plot.pdf]

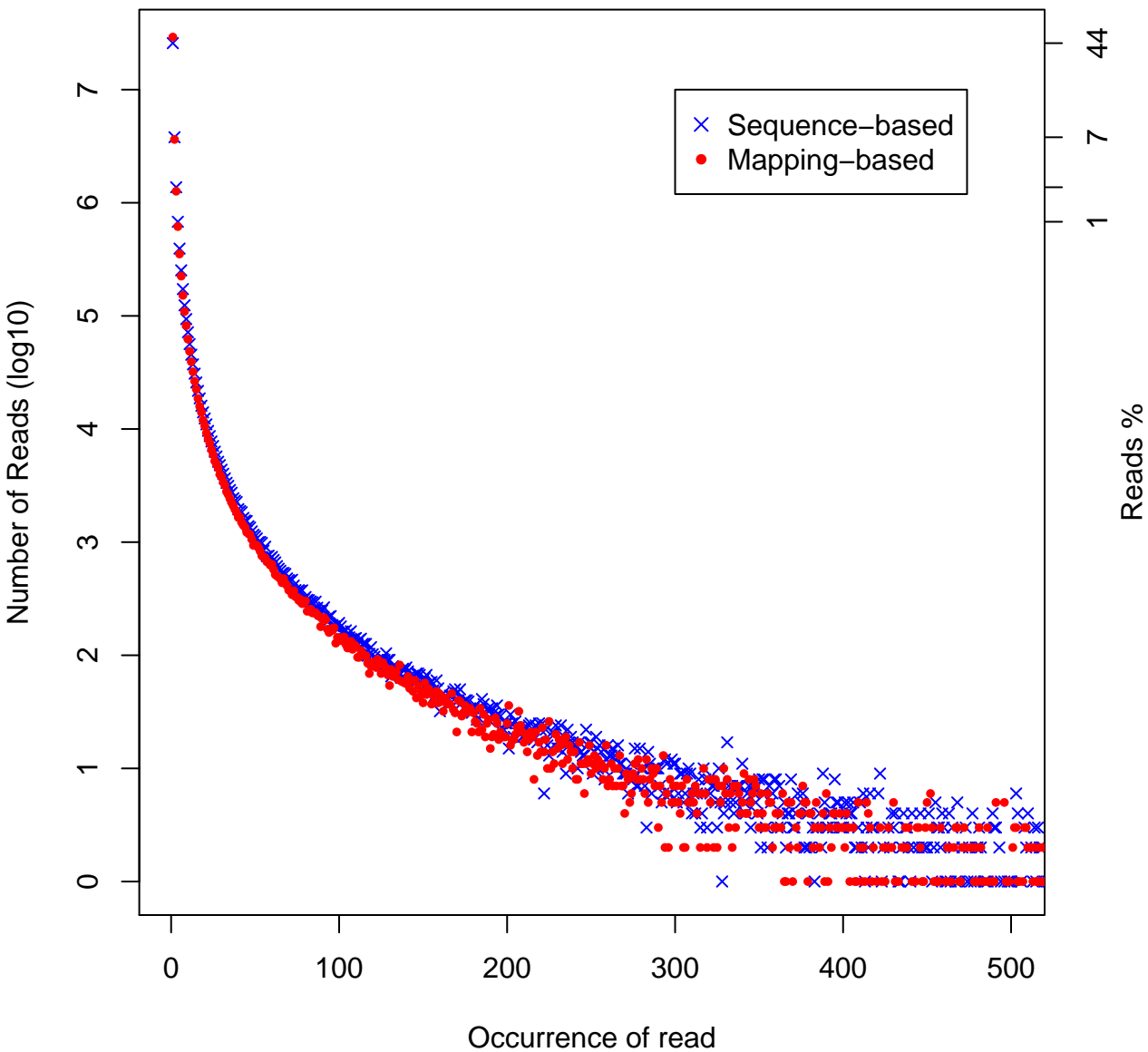

Supplement: S1 Data — This directory contains six subdirectories. The subdirectory “correlation” contains correlation heatmaps among all samples in diencephalon and telencephalon respectively. The subdirectory “mapping_stat” contains read mapping information on genomic features for each sample. The subdirectory “MDS” shows three dimensional MDS plots of the samples. The subdirectory “ReadDuplication” contains read duplication distributions for each sample. The subdirectory “ReadQuality” contains reads quality information for each sample plotted as both boxplots and heatmaps. The subdirectory “RPKMSaturation” contains information about read depth saturation for each sample as assessed by RPKM resamplings. All transcripts were divided into four quantiles based on their expression and a relative difference of observed and real RPKM values are plotted for each sample. (ZIP) [file pgen.1006840.s015.zip › RNASeq/ReadDuplication/2D_ATTACTCG-GGCTCTGA_L00M_R1_001.DupRate_plot.pdf]

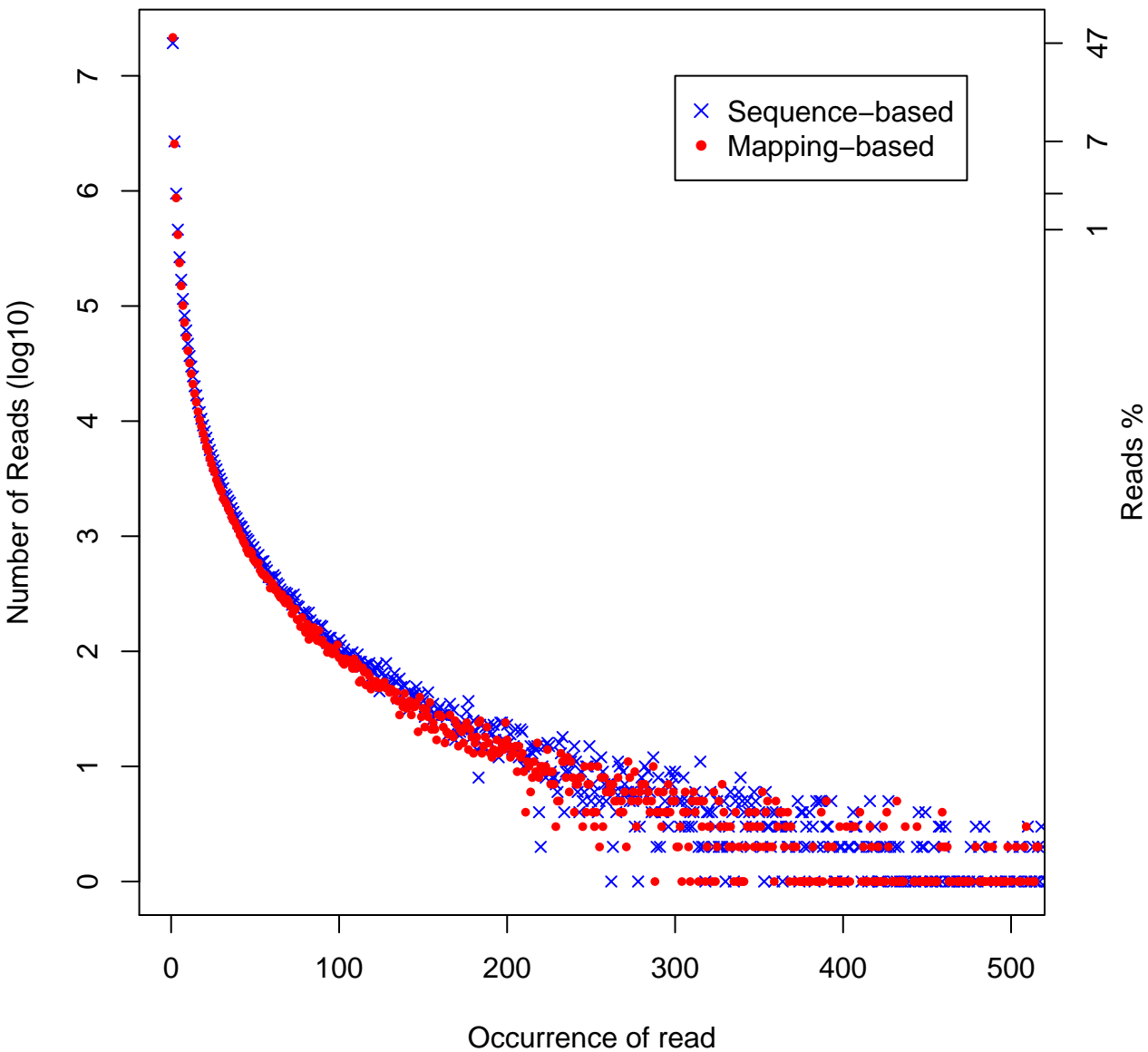

Supplement: S1 Data — This directory contains six subdirectories. The subdirectory “correlation” contains correlation heatmaps among all samples in diencephalon and telencephalon respectively. The subdirectory “mapping_stat” contains read mapping information on genomic features for each sample. The subdirectory “MDS” shows three dimensional MDS plots of the samples. The subdirectory “ReadDuplication” contains read duplication distributions for each sample. The subdirectory “ReadQuality” contains reads quality information for each sample plotted as both boxplots and heatmaps. The subdirectory “RPKMSaturation” contains information about read depth saturation for each sample as assessed by RPKM resamplings. All transcripts were divided into four quantiles based on their expression and a relative difference of observed and real RPKM values are plotted for each sample. (ZIP) [file pgen.1006840.s015.zip › RNASeq/ReadDuplication/2T_ATTACTCG-CCTATCCT_L00M_R1_001.DupRate_plot.pdf]

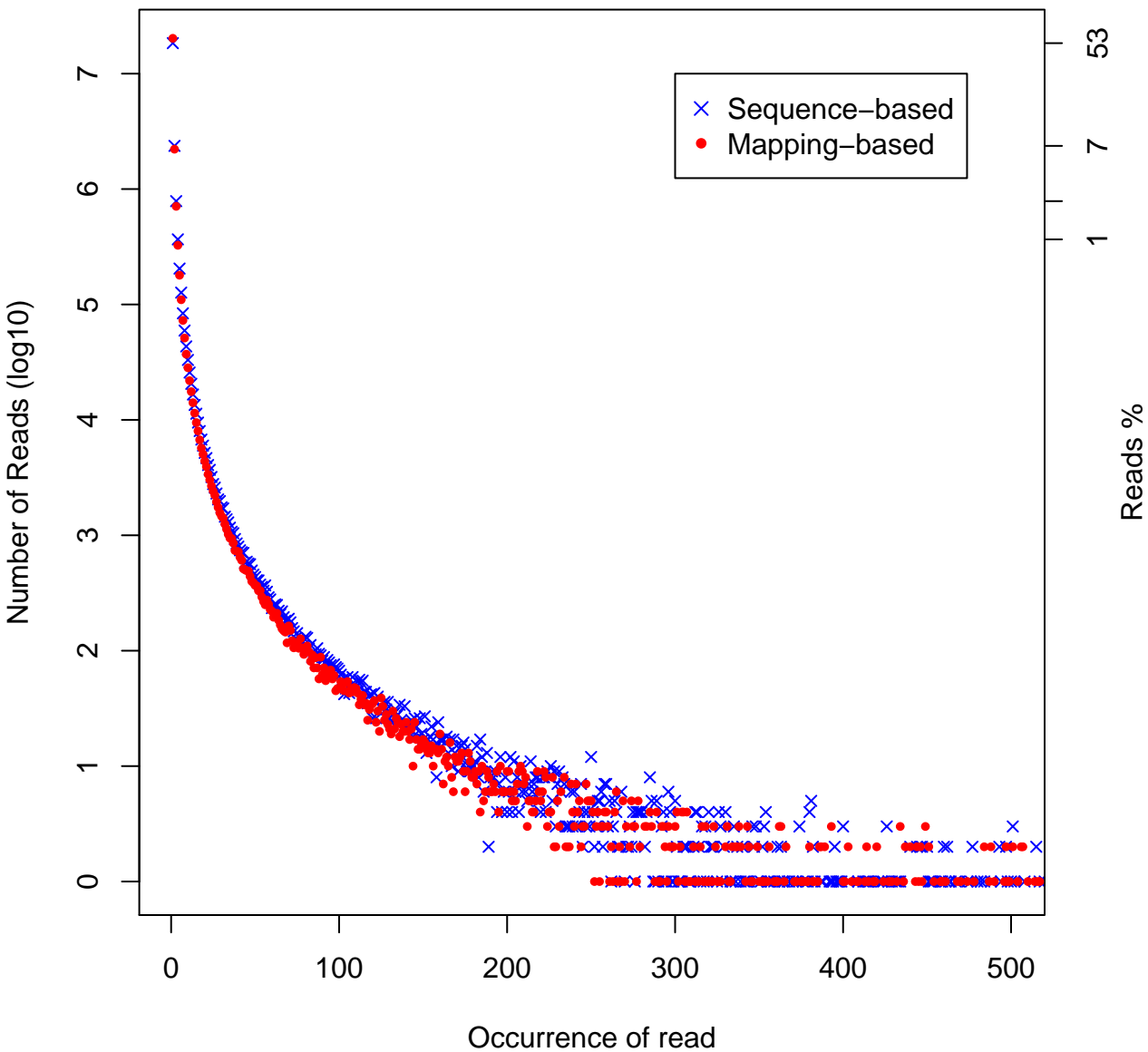

Supplement: S1 Data — This directory contains six subdirectories. The subdirectory “correlation” contains correlation heatmaps among all samples in diencephalon and telencephalon respectively. The subdirectory “mapping_stat” contains read mapping information on genomic features for each sample. The subdirectory “MDS” shows three dimensional MDS plots of the samples. The subdirectory “ReadDuplication” contains read duplication distributions for each sample. The subdirectory “ReadQuality” contains reads quality information for each sample plotted as both boxplots and heatmaps. The subdirectory “RPKMSaturation” contains information about read depth saturation for each sample as assessed by RPKM resamplings. All transcripts were divided into four quantiles based on their expression and a relative difference of observed and real RPKM values are plotted for each sample. (ZIP) [file pgen.1006840.s015.zip › RNASeq/ReadDuplication/30D_GAATTCGT-GTACTGAC_L00M_R1_001.DupRate_plot.pdf]

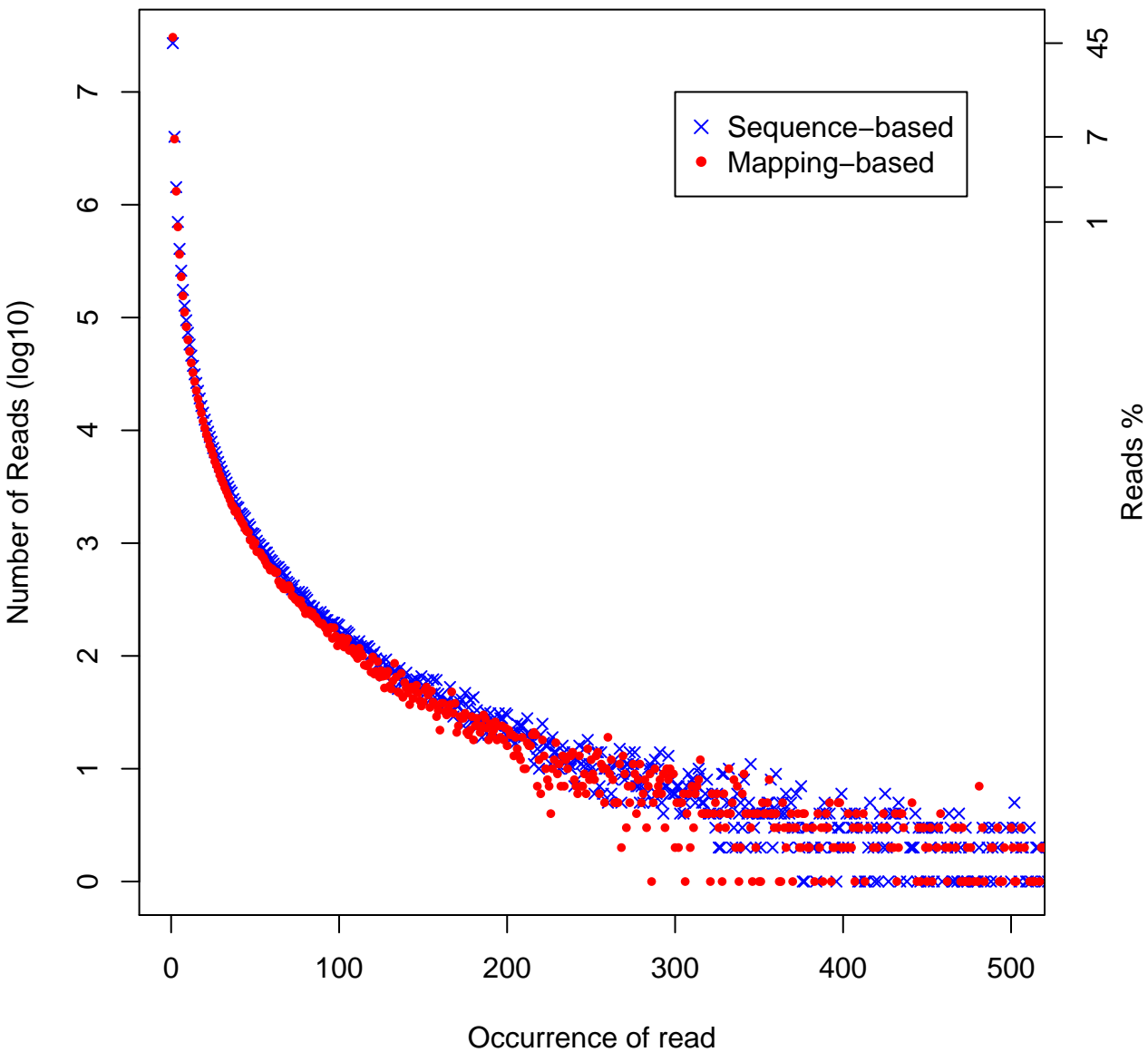

Supplement: S1 Data — This directory contains six subdirectories. The subdirectory “correlation” contains correlation heatmaps among all samples in diencephalon and telencephalon respectively. The subdirectory “mapping_stat” contains read mapping information on genomic features for each sample. The subdirectory “MDS” shows three dimensional MDS plots of the samples. The subdirectory “ReadDuplication” contains read duplication distributions for each sample. The subdirectory “ReadQuality” contains reads quality information for each sample plotted as both boxplots and heatmaps. The subdirectory “RPKMSaturation” contains information about read depth saturation for each sample as assessed by RPKM resamplings. All transcripts were divided into four quantiles based on their expression and a relative difference of observed and real RPKM values are plotted for each sample. (ZIP) [file pgen.1006840.s015.zip › RNASeq/ReadDuplication/30T_GAATTCGT-CAGGACGT_L00M_R1_001.DupRate_plot.pdf]

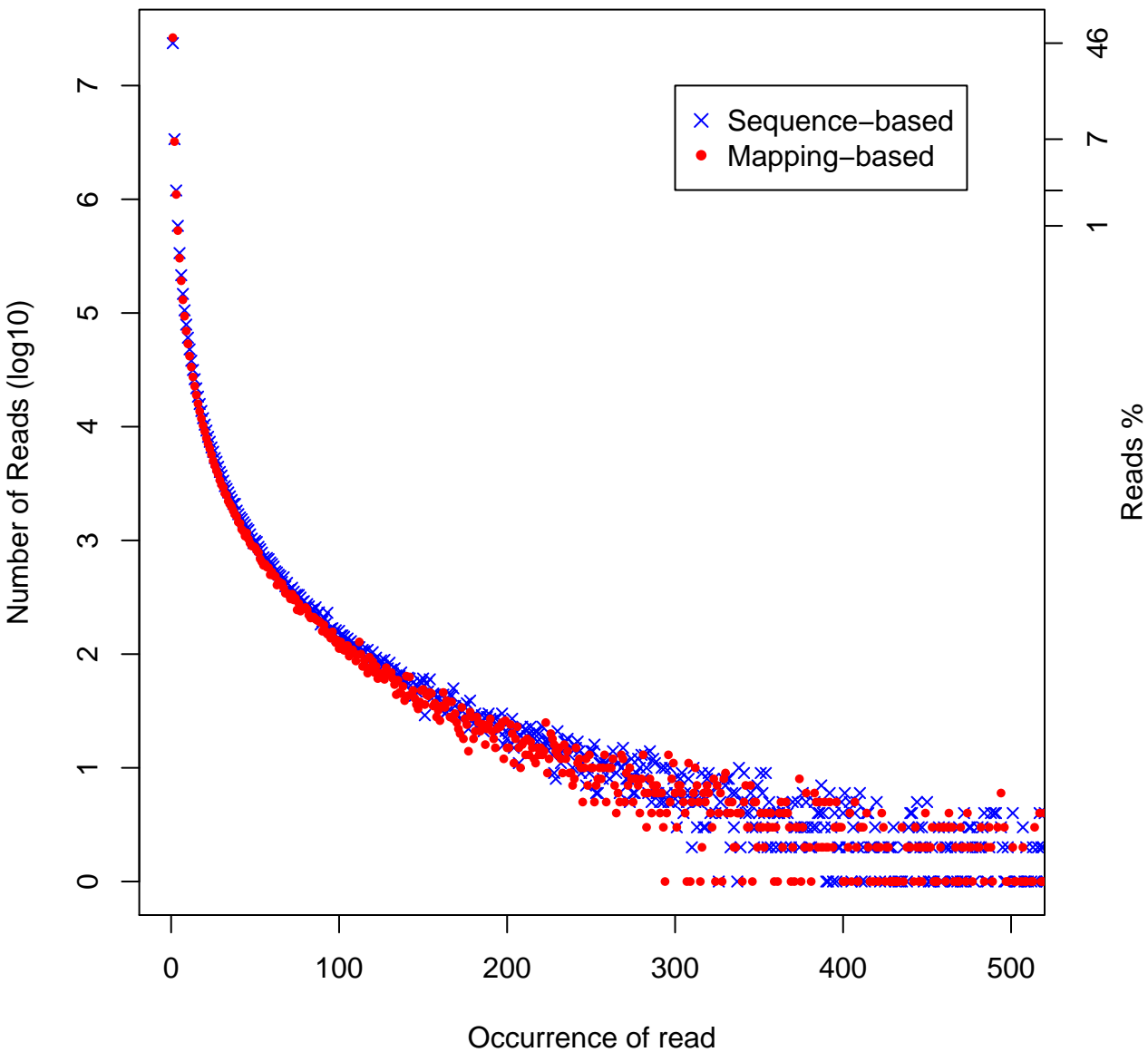

Supplement: S1 Data — This directory contains six subdirectories. The subdirectory “correlation” contains correlation heatmaps among all samples in diencephalon and telencephalon respectively. The subdirectory “mapping_stat” contains read mapping information on genomic features for each sample. The subdirectory “MDS” shows three dimensional MDS plots of the samples. The subdirectory “ReadDuplication” contains read duplication distributions for each sample. The subdirectory “ReadQuality” contains reads quality information for each sample plotted as both boxplots and heatmaps. The subdirectory “RPKMSaturation” contains information about read depth saturation for each sample as assessed by RPKM resamplings. All transcripts were divided into four quantiles based on their expression and a relative difference of observed and real RPKM values are plotted for each sample. (ZIP) [file pgen.1006840.s015.zip › RNASeq/ReadDuplication/32D_CTGAAGCT-ATAGAGGC_L00M_R1_001.DupRate_plot.pdf]

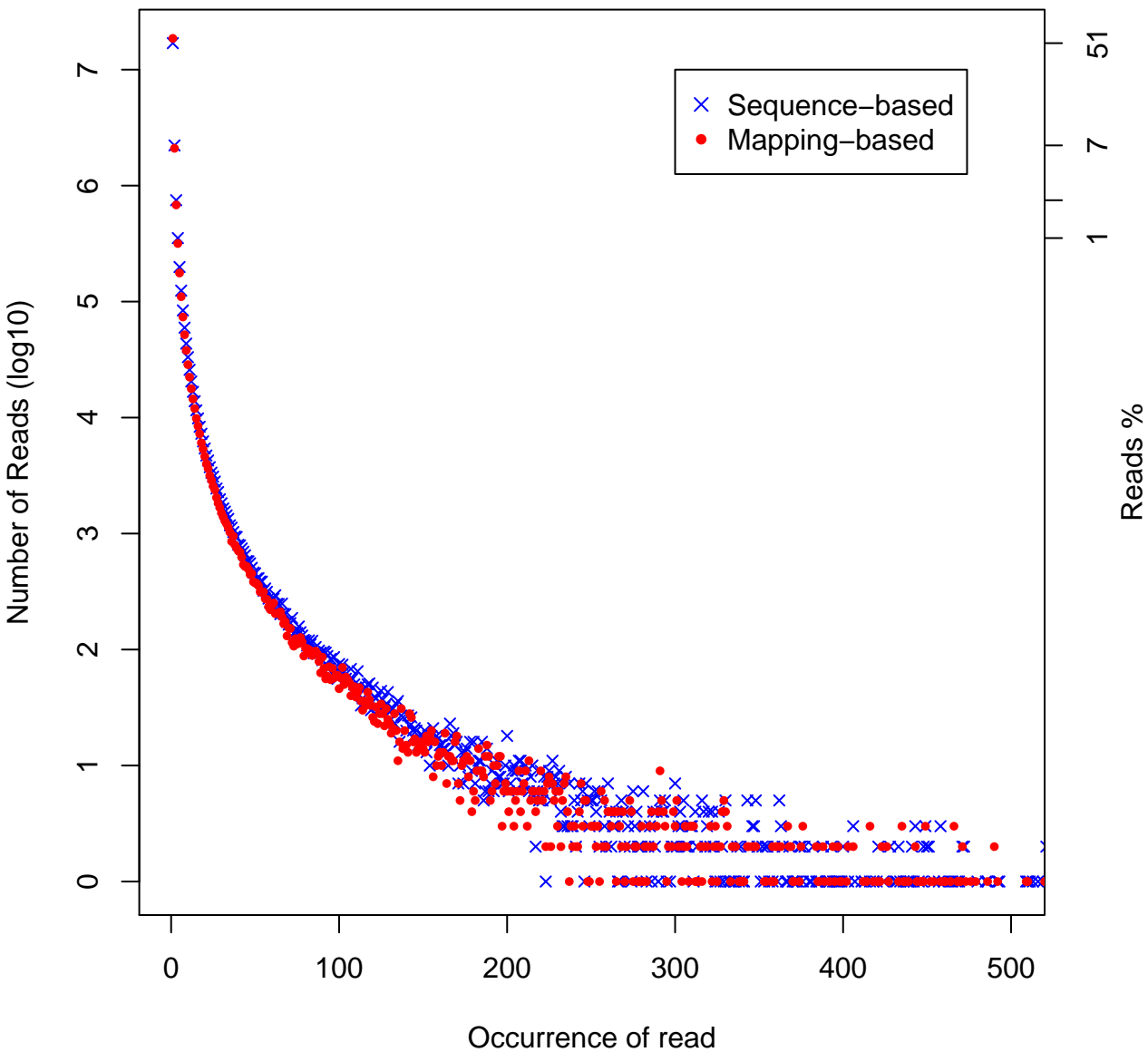

Supplement: S1 Data — This directory contains six subdirectories. The subdirectory “correlation” contains correlation heatmaps among all samples in diencephalon and telencephalon respectively. The subdirectory “mapping_stat” contains read mapping information on genomic features for each sample. The subdirectory “MDS” shows three dimensional MDS plots of the samples. The subdirectory “ReadDuplication” contains read duplication distributions for each sample. The subdirectory “ReadQuality” contains reads quality information for each sample plotted as both boxplots and heatmaps. The subdirectory “RPKMSaturation” contains information about read depth saturation for each sample as assessed by RPKM resamplings. All transcripts were divided into four quantiles based on their expression and a relative difference of observed and real RPKM values are plotted for each sample. (ZIP) [file pgen.1006840.s015.zip › RNASeq/ReadDuplication/32T_CTGAAGCT-TATAGCCT_L00M_R1_001.DupRate_plot.pdf]

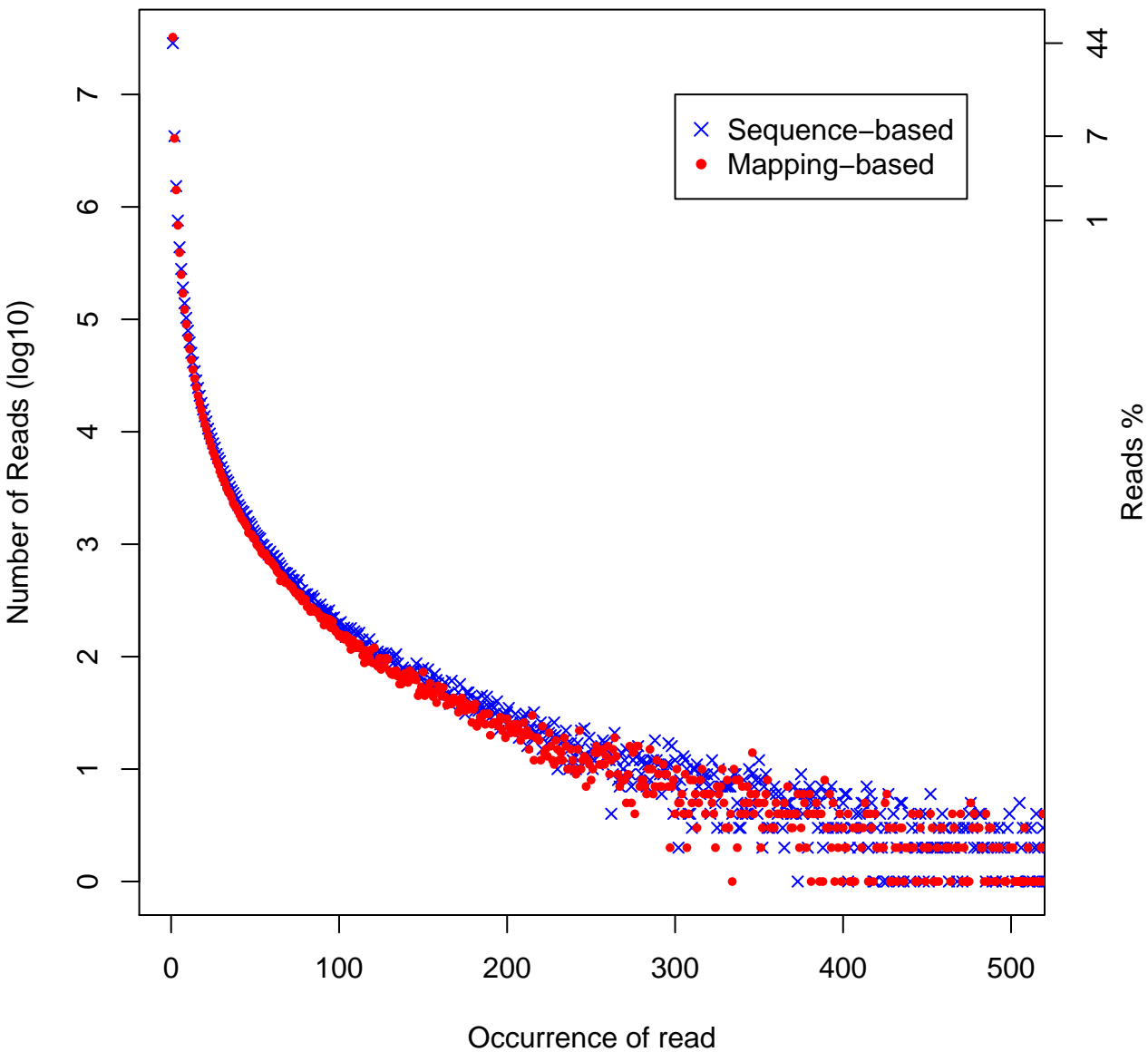

Supplement: S1 Data — This directory contains six subdirectories. The subdirectory “correlation” contains correlation heatmaps among all samples in diencephalon and telencephalon respectively. The subdirectory “mapping_stat” contains read mapping information on genomic features for each sample. The subdirectory “MDS” shows three dimensional MDS plots of the samples. The subdirectory “ReadDuplication” contains read duplication distributions for each sample. The subdirectory “ReadQuality” contains reads quality information for each sample plotted as both boxplots and heatmaps. The subdirectory “RPKMSaturation” contains information about read depth saturation for each sample as assessed by RPKM resamplings. All transcripts were divided into four quantiles based on their expression and a relative difference of observed and real RPKM values are plotted for each sample. (ZIP) [file pgen.1006840.s015.zip › RNASeq/ReadDuplication/33D_CTGAAGCT-GGCTCTGA_L00M_R1_001.DupRate_plot.pdf]

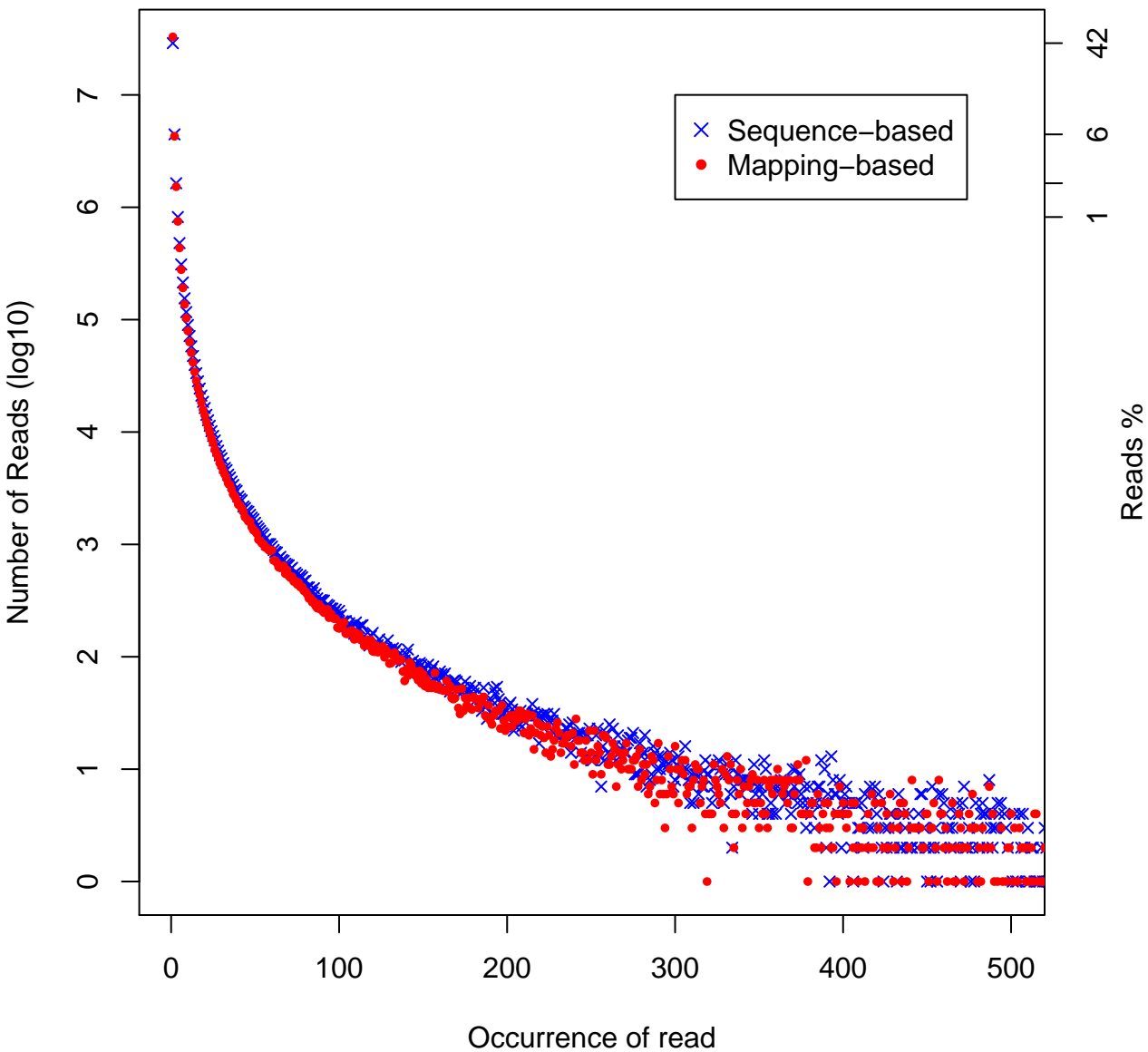

Supplement: S1 Data — This directory contains six subdirectories. The subdirectory “correlation” contains correlation heatmaps among all samples in diencephalon and telencephalon respectively. The subdirectory “mapping_stat” contains read mapping information on genomic features for each sample. The subdirectory “MDS” shows three dimensional MDS plots of the samples. The subdirectory “ReadDuplication” contains read duplication distributions for each sample. The subdirectory “ReadQuality” contains reads quality information for each sample plotted as both boxplots and heatmaps. The subdirectory “RPKMSaturation” contains information about read depth saturation for each sample as assessed by RPKM resamplings. All transcripts were divided into four quantiles based on their expression and a relative difference of observed and real RPKM values are plotted for each sample. (ZIP) [file pgen.1006840.s015.zip › RNASeq/ReadDuplication/33T_CTGAAGCT-CCTATCCT_L00M_R1_001.DupRate_plot.pdf]

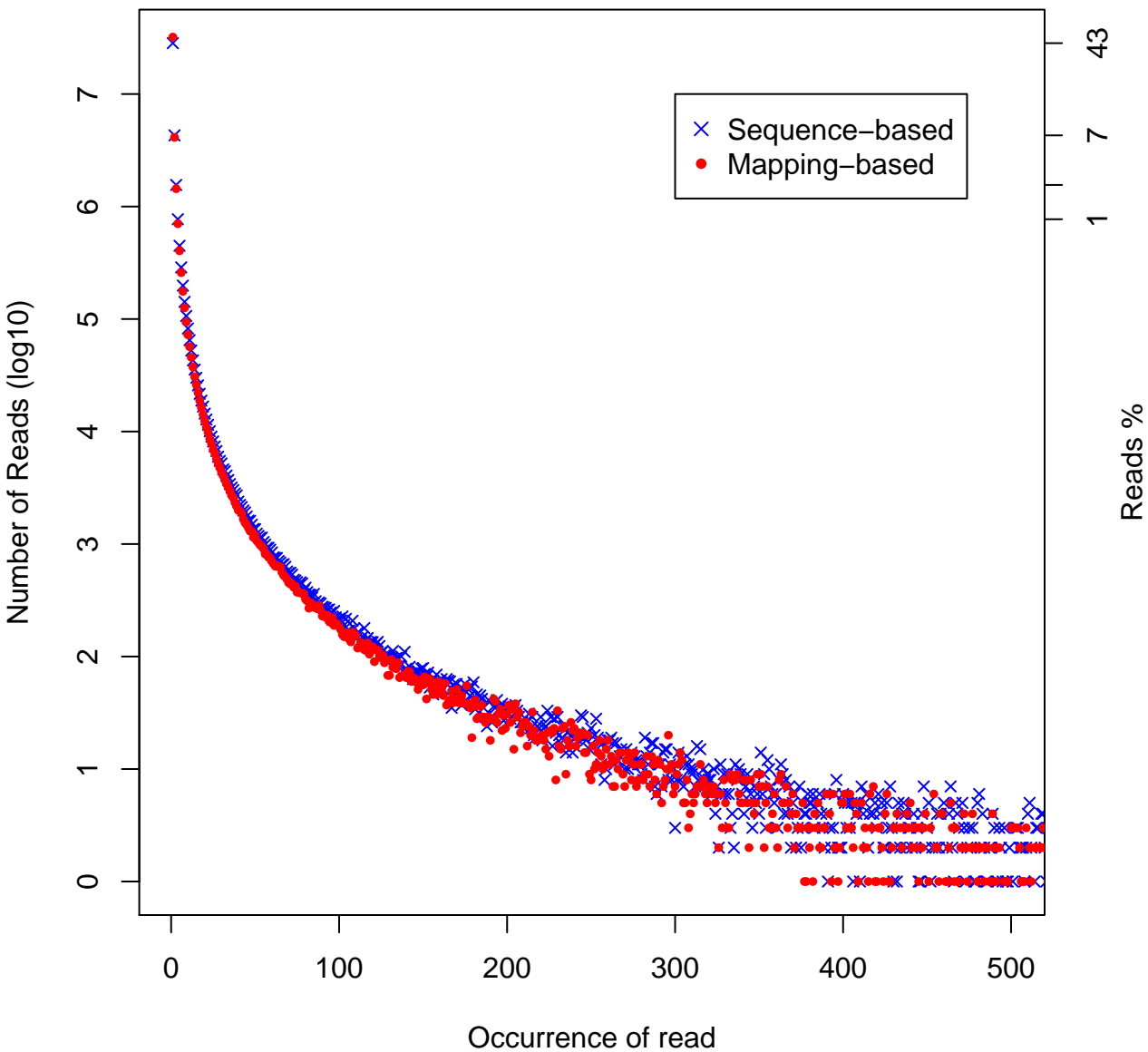

Supplement: S1 Data — This directory contains six subdirectories. The subdirectory “correlation” contains correlation heatmaps among all samples in diencephalon and telencephalon respectively. The subdirectory “mapping_stat” contains read mapping information on genomic features for each sample. The subdirectory “MDS” shows three dimensional MDS plots of the samples. The subdirectory “ReadDuplication” contains read duplication distributions for each sample. The subdirectory “ReadQuality” contains reads quality information for each sample plotted as both boxplots and heatmaps. The subdirectory “RPKMSaturation” contains information about read depth saturation for each sample as assessed by RPKM resamplings. All transcripts were divided into four quantiles based on their expression and a relative difference of observed and real RPKM values are plotted for each sample. (ZIP) [file pgen.1006840.s015.zip › RNASeq/ReadDuplication/34D_CTGAAGCT-TAATCTTA_L00M_R1_001.DupRate_plot.pdf]

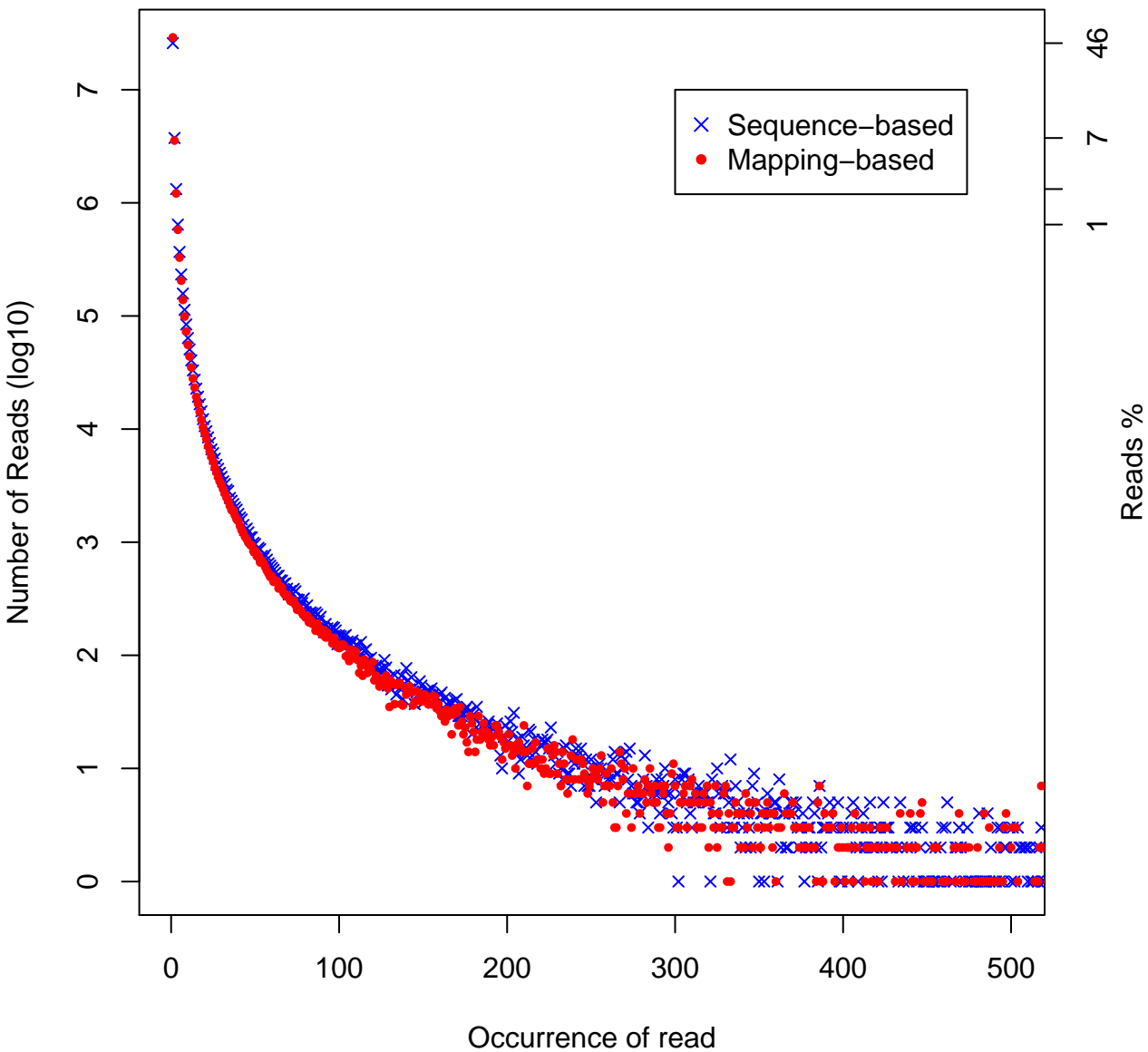

Supplement: S1 Data — This directory contains six subdirectories. The subdirectory “correlation” contains correlation heatmaps among all samples in diencephalon and telencephalon respectively. The subdirectory “mapping_stat” contains read mapping information on genomic features for each sample. The subdirectory “MDS” shows three dimensional MDS plots of the samples. The subdirectory “ReadDuplication” contains read duplication distributions for each sample. The subdirectory “ReadQuality” contains reads quality information for each sample plotted as both boxplots and heatmaps. The subdirectory “RPKMSaturation” contains information about read depth saturation for each sample as assessed by RPKM resamplings. All transcripts were divided into four quantiles based on their expression and a relative difference of observed and real RPKM values are plotted for each sample. (ZIP) [file pgen.1006840.s015.zip › RNASeq/ReadDuplication/34T_CTGAAGCT-AGGCGAAG_L00M_R1_001.DupRate_plot.pdf]

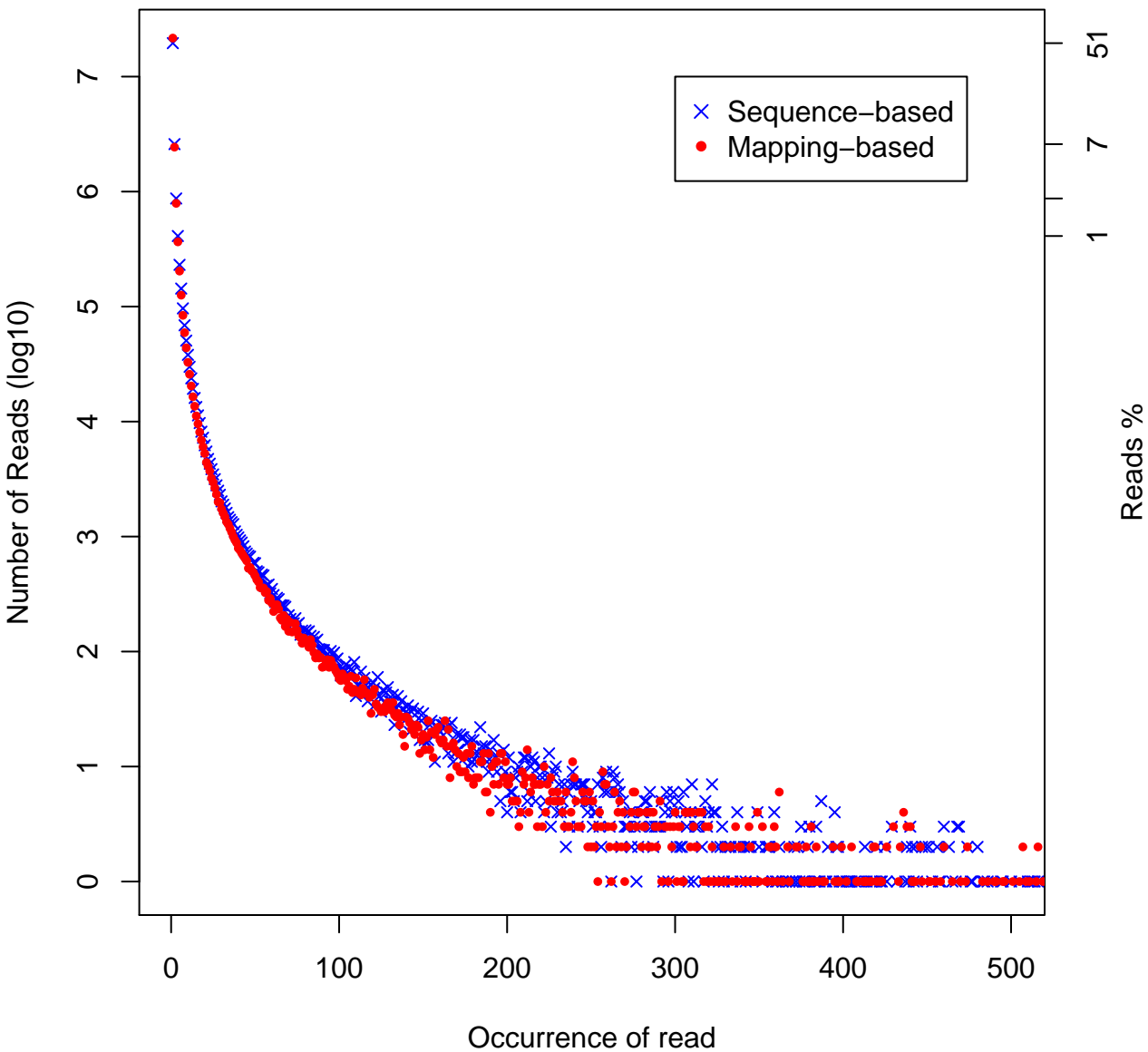

Supplement: S1 Data — This directory contains six subdirectories. The subdirectory “correlation” contains correlation heatmaps among all samples in diencephalon and telencephalon respectively. The subdirectory “mapping_stat” contains read mapping information on genomic features for each sample. The subdirectory “MDS” shows three dimensional MDS plots of the samples. The subdirectory “ReadDuplication” contains read duplication distributions for each sample. The subdirectory “ReadQuality” contains reads quality information for each sample plotted as both boxplots and heatmaps. The subdirectory “RPKMSaturation” contains information about read depth saturation for each sample as assessed by RPKM resamplings. All transcripts were divided into four quantiles based on their expression and a relative difference of observed and real RPKM values are plotted for each sample. (ZIP) [file pgen.1006840.s015.zip › RNASeq/ReadDuplication/35D_CTGAAGCT-GTACTGAC_L00M_R1_001.DupRate_plot.pdf]

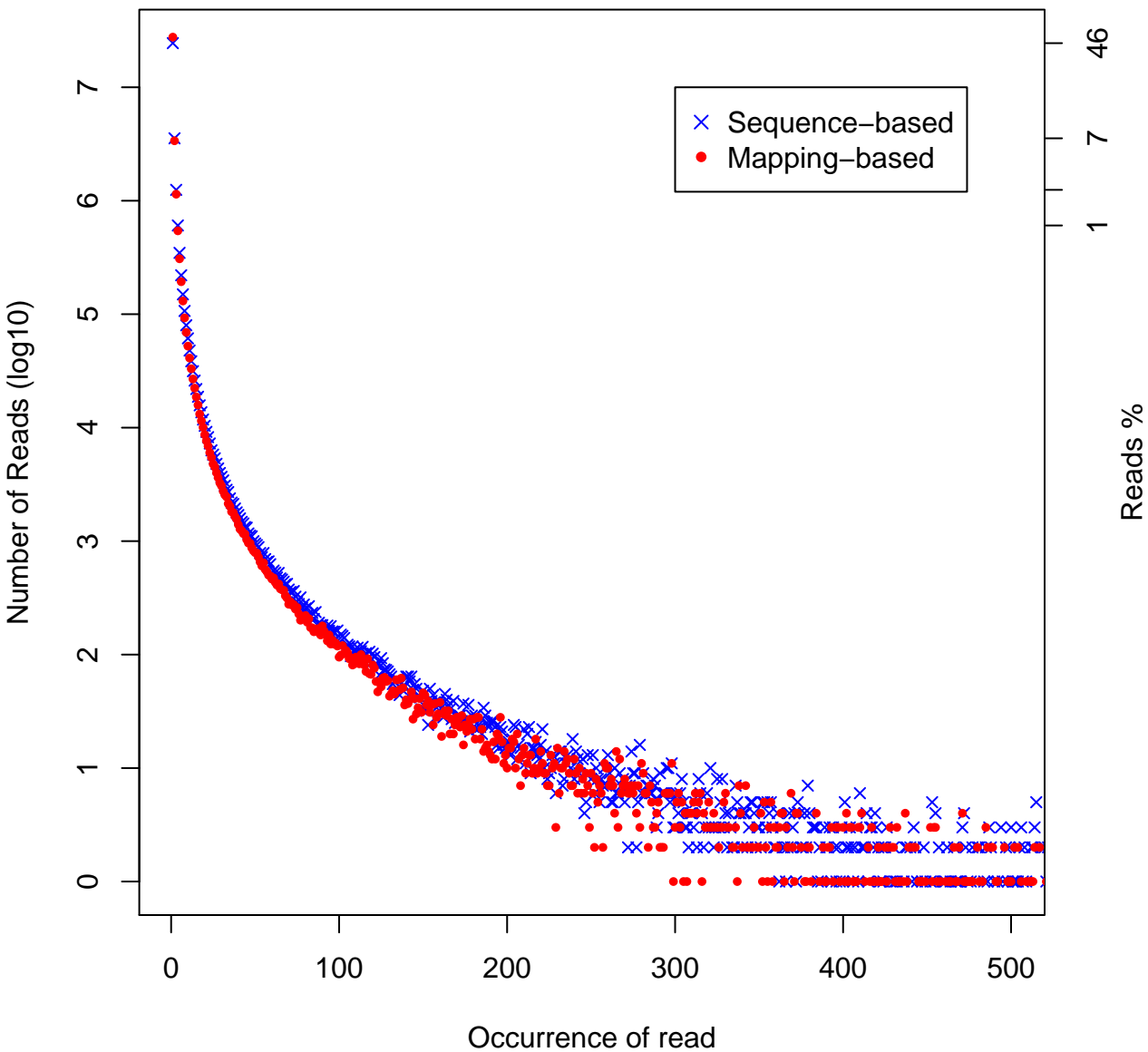

Supplement: S1 Data — This directory contains six subdirectories. The subdirectory “correlation” contains correlation heatmaps among all samples in diencephalon and telencephalon respectively. The subdirectory “mapping_stat” contains read mapping information on genomic features for each sample. The subdirectory “MDS” shows three dimensional MDS plots of the samples. The subdirectory “ReadDuplication” contains read duplication distributions for each sample. The subdirectory “ReadQuality” contains reads quality information for each sample plotted as both boxplots and heatmaps. The subdirectory “RPKMSaturation” contains information about read depth saturation for each sample as assessed by RPKM resamplings. All transcripts were divided into four quantiles based on their expression and a relative difference of observed and real RPKM values are plotted for each sample. (ZIP) [file pgen.1006840.s015.zip › RNASeq/ReadDuplication/35T_CTGAAGCT-CAGGACGT_L00M_R1_001.DupRate_plot.pdf]

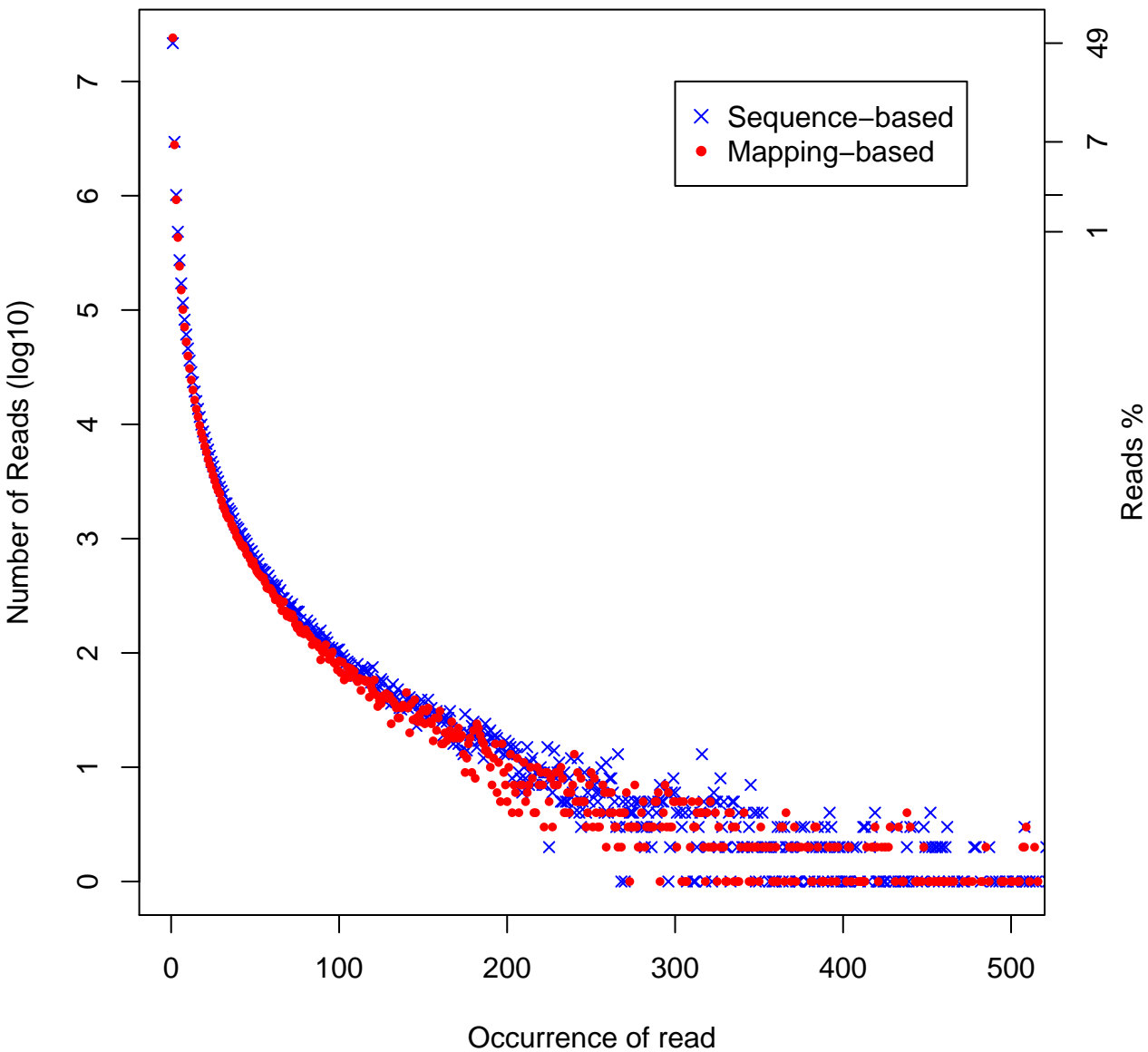

Supplement: S1 Data — This directory contains six subdirectories. The subdirectory “correlation” contains correlation heatmaps among all samples in diencephalon and telencephalon respectively. The subdirectory “mapping_stat” contains read mapping information on genomic features for each sample. The subdirectory “MDS” shows three dimensional MDS plots of the samples. The subdirectory “ReadDuplication” contains read duplication distributions for each sample. The subdirectory “ReadQuality” contains reads quality information for each sample plotted as both boxplots and heatmaps. The subdirectory “RPKMSaturation” contains information about read depth saturation for each sample as assessed by RPKM resamplings. All transcripts were divided into four quantiles based on their expression and a relative difference of observed and real RPKM values are plotted for each sample. (ZIP) [file pgen.1006840.s015.zip › RNASeq/ReadDuplication/36D_TAATGCGC-ATAGAGGC_L00M_R1_001.DupRate_plot.pdf]

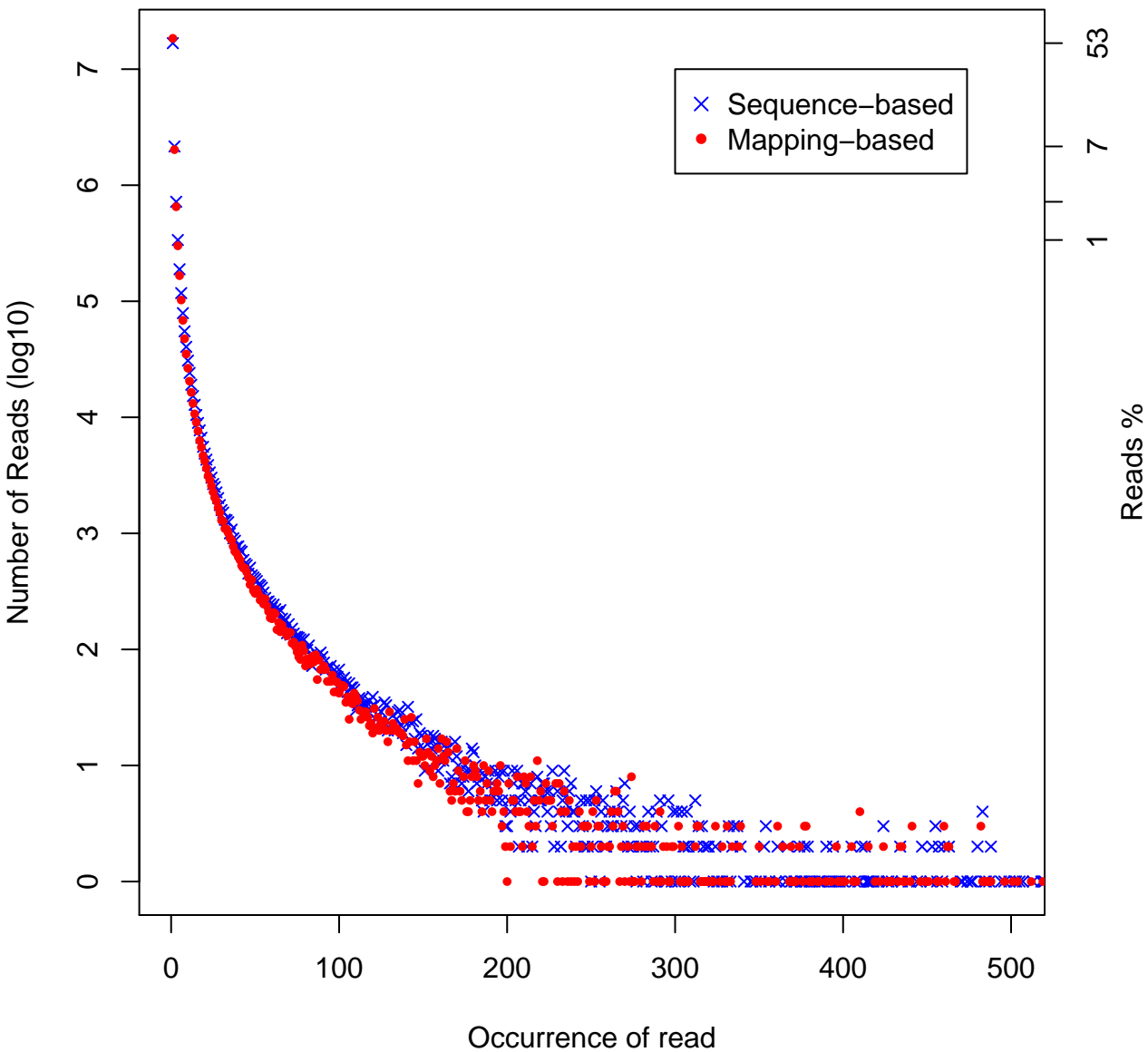

Supplement: S1 Data — This directory contains six subdirectories. The subdirectory “correlation” contains correlation heatmaps among all samples in diencephalon and telencephalon respectively. The subdirectory “mapping_stat” contains read mapping information on genomic features for each sample. The subdirectory “MDS” shows three dimensional MDS plots of the samples. The subdirectory “ReadDuplication” contains read duplication distributions for each sample. The subdirectory “ReadQuality” contains reads quality information for each sample plotted as both boxplots and heatmaps. The subdirectory “RPKMSaturation” contains information about read depth saturation for each sample as assessed by RPKM resamplings. All transcripts were divided into four quantiles based on their expression and a relative difference of observed and real RPKM values are plotted for each sample. (ZIP) [file pgen.1006840.s015.zip › RNASeq/ReadDuplication/36T_TAATGCGC-TATAGCCT_L00M_R1_001.DupRate_plot.pdf]

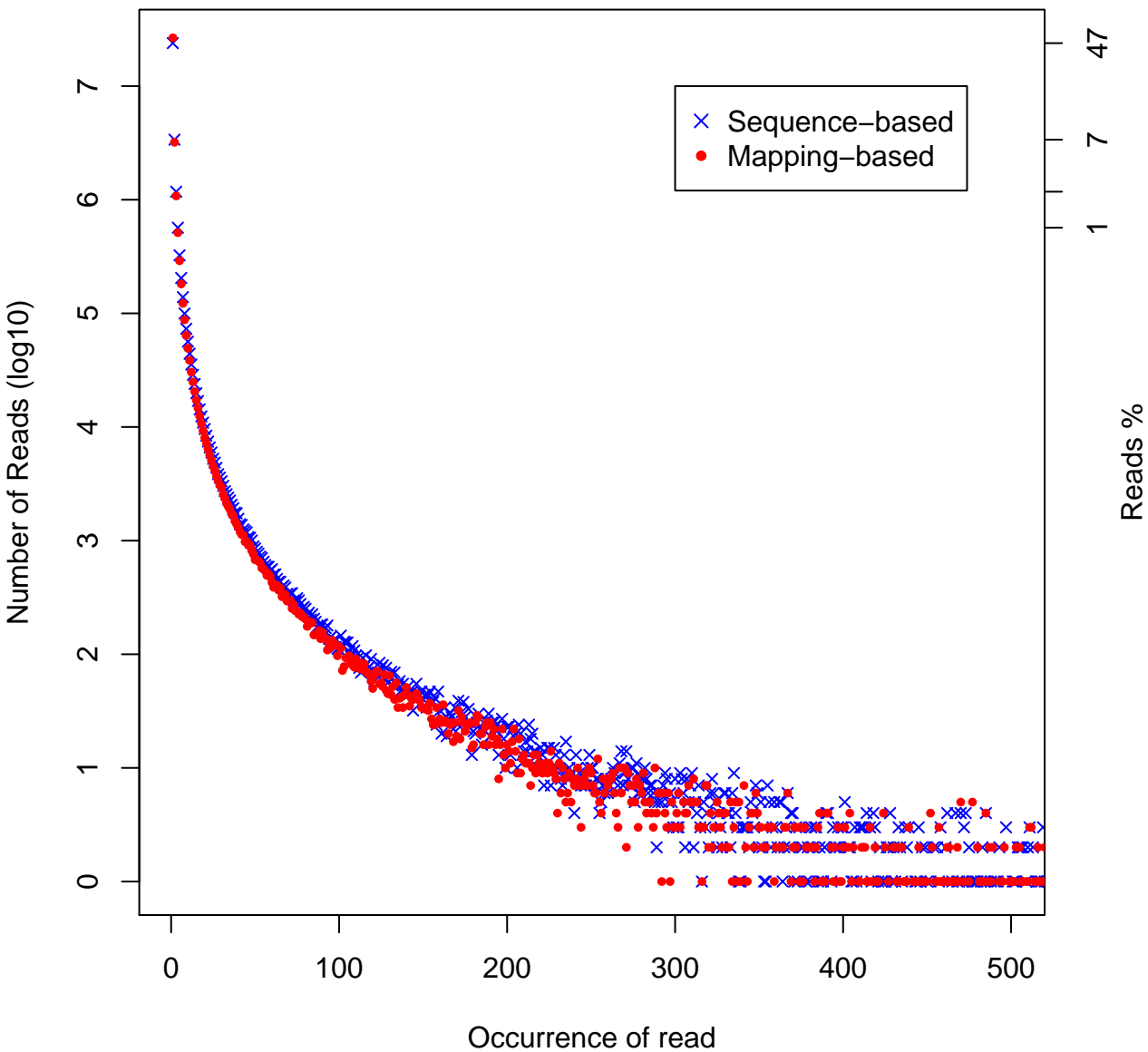

Supplement: S1 Data — This directory contains six subdirectories. The subdirectory “correlation” contains correlation heatmaps among all samples in diencephalon and telencephalon respectively. The subdirectory “mapping_stat” contains read mapping information on genomic features for each sample. The subdirectory “MDS” shows three dimensional MDS plots of the samples. The subdirectory “ReadDuplication” contains read duplication distributions for each sample. The subdirectory “ReadQuality” contains reads quality information for each sample plotted as both boxplots and heatmaps. The subdirectory “RPKMSaturation” contains information about read depth saturation for each sample as assessed by RPKM resamplings. All transcripts were divided into four quantiles based on their expression and a relative difference of observed and real RPKM values are plotted for each sample. (ZIP) [file pgen.1006840.s015.zip › RNASeq/ReadDuplication/38D_TAATGCGC-GGCTCTGA_L00M_R1_001.DupRate_plot.pdf]

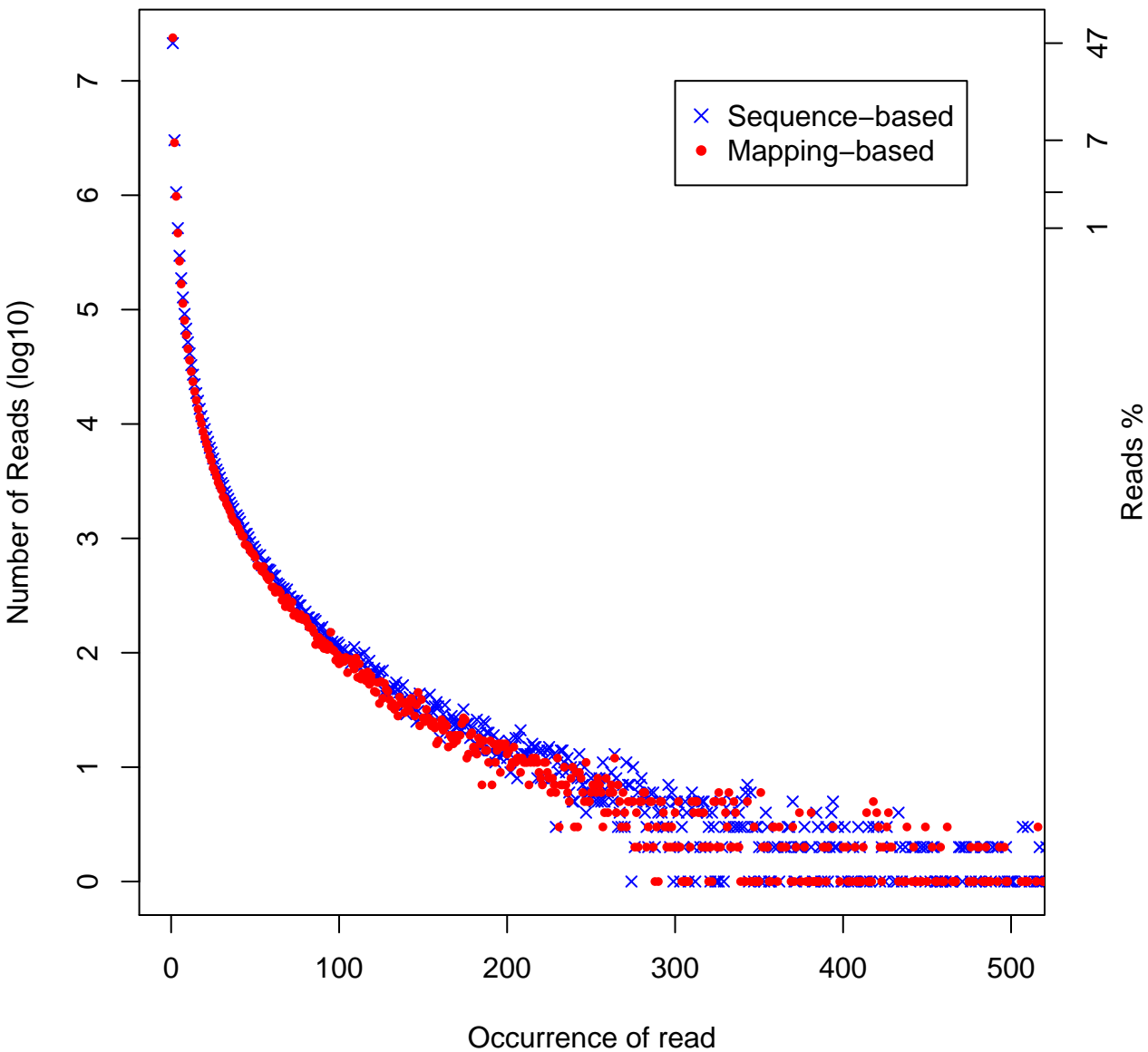

Supplement: S1 Data — This directory contains six subdirectories. The subdirectory “correlation” contains correlation heatmaps among all samples in diencephalon and telencephalon respectively. The subdirectory “mapping_stat” contains read mapping information on genomic features for each sample. The subdirectory “MDS” shows three dimensional MDS plots of the samples. The subdirectory “ReadDuplication” contains read duplication distributions for each sample. The subdirectory “ReadQuality” contains reads quality information for each sample plotted as both boxplots and heatmaps. The subdirectory “RPKMSaturation” contains information about read depth saturation for each sample as assessed by RPKM resamplings. All transcripts were divided into four quantiles based on their expression and a relative difference of observed and real RPKM values are plotted for each sample. (ZIP) [file pgen.1006840.s015.zip › RNASeq/ReadDuplication/38T_TAATGCGC-CCTATCCT_L00M_R1_001.DupRate_plot.pdf]

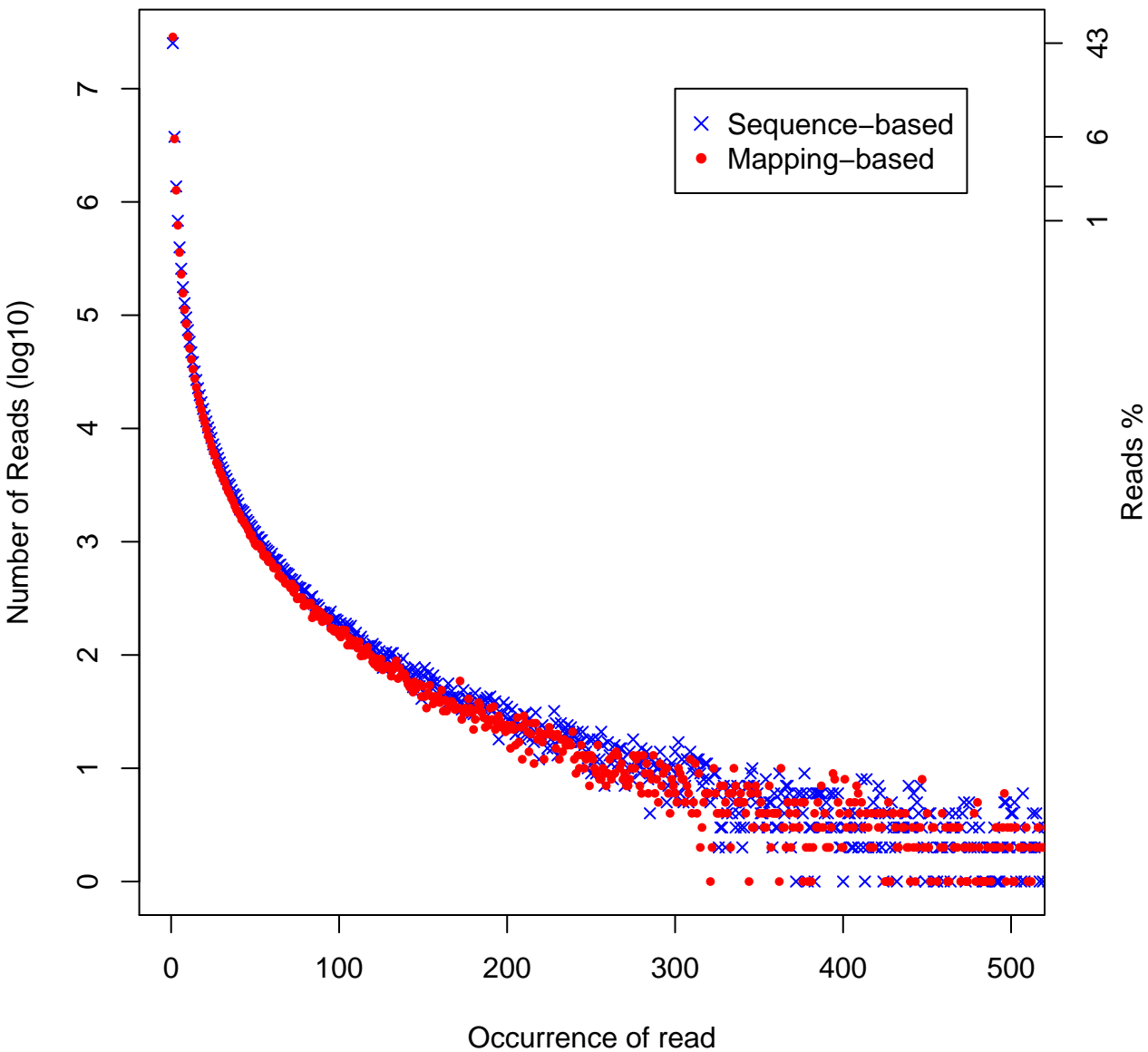

Supplement: S1 Data — This directory contains six subdirectories. The subdirectory “correlation” contains correlation heatmaps among all samples in diencephalon and telencephalon respectively. The subdirectory “mapping_stat” contains read mapping information on genomic features for each sample. The subdirectory “MDS” shows three dimensional MDS plots of the samples. The subdirectory “ReadDuplication” contains read duplication distributions for each sample. The subdirectory “ReadQuality” contains reads quality information for each sample plotted as both boxplots and heatmaps. The subdirectory “RPKMSaturation” contains information about read depth saturation for each sample as assessed by RPKM resamplings. All transcripts were divided into four quantiles based on their expression and a relative difference of observed and real RPKM values are plotted for each sample. (ZIP) [file pgen.1006840.s015.zip › RNASeq/ReadDuplication/3D_ATTACTCG-TAATCTTA_L00M_R1_001.DupRate_plot.pdf]

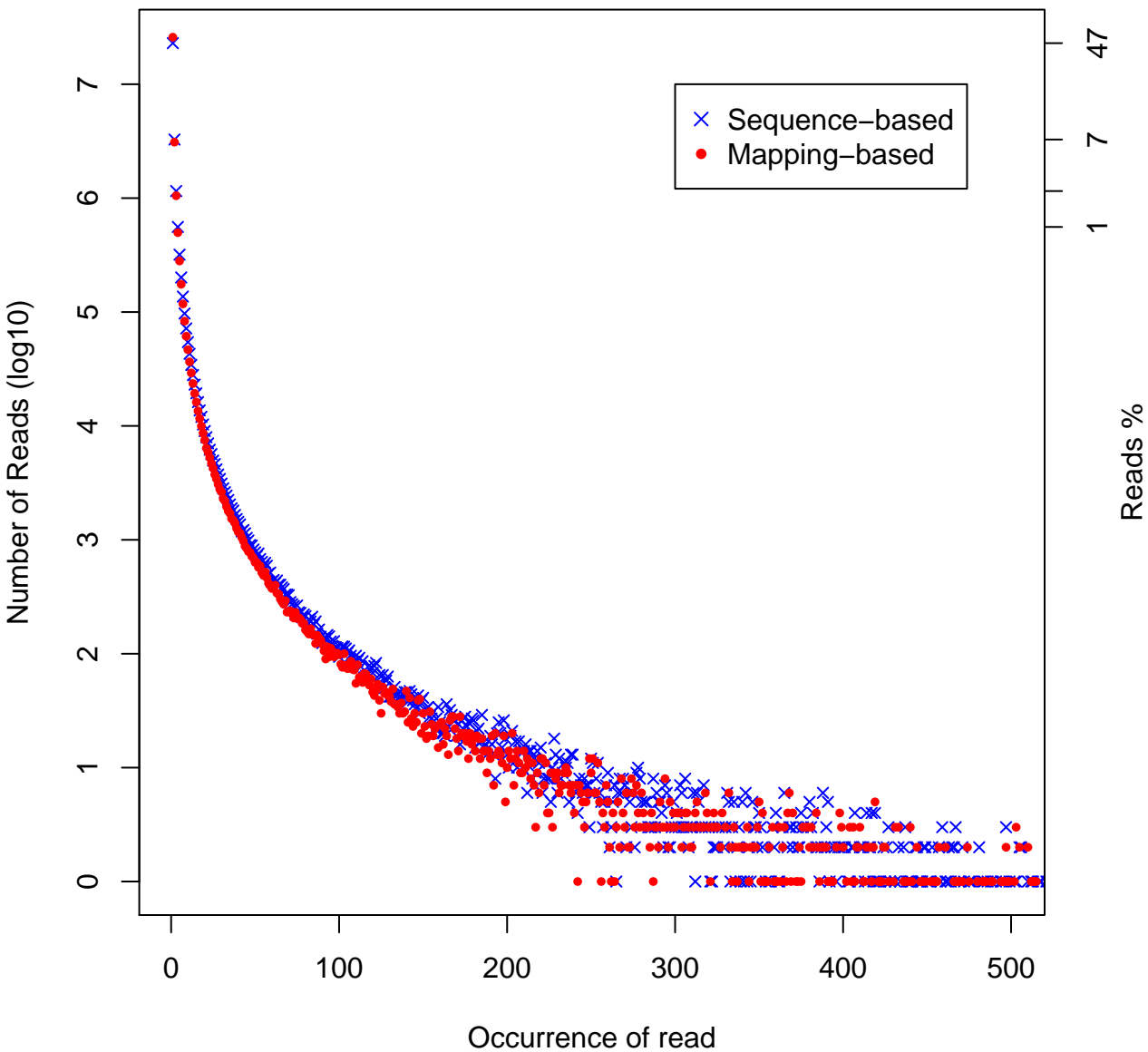

Supplement: S1 Data — This directory contains six subdirectories. The subdirectory “correlation” contains correlation heatmaps among all samples in diencephalon and telencephalon respectively. The subdirectory “mapping_stat” contains read mapping information on genomic features for each sample. The subdirectory “MDS” shows three dimensional MDS plots of the samples. The subdirectory “ReadDuplication” contains read duplication distributions for each sample. The subdirectory “ReadQuality” contains reads quality information for each sample plotted as both boxplots and heatmaps. The subdirectory “RPKMSaturation” contains information about read depth saturation for each sample as assessed by RPKM resamplings. All transcripts were divided into four quantiles based on their expression and a relative difference of observed and real RPKM values are plotted for each sample. (ZIP) [file pgen.1006840.s015.zip › RNASeq/ReadDuplication/3T_ATTACTCG-AGGCGAAG_L00M_R1_001.DupRate_plot.pdf]

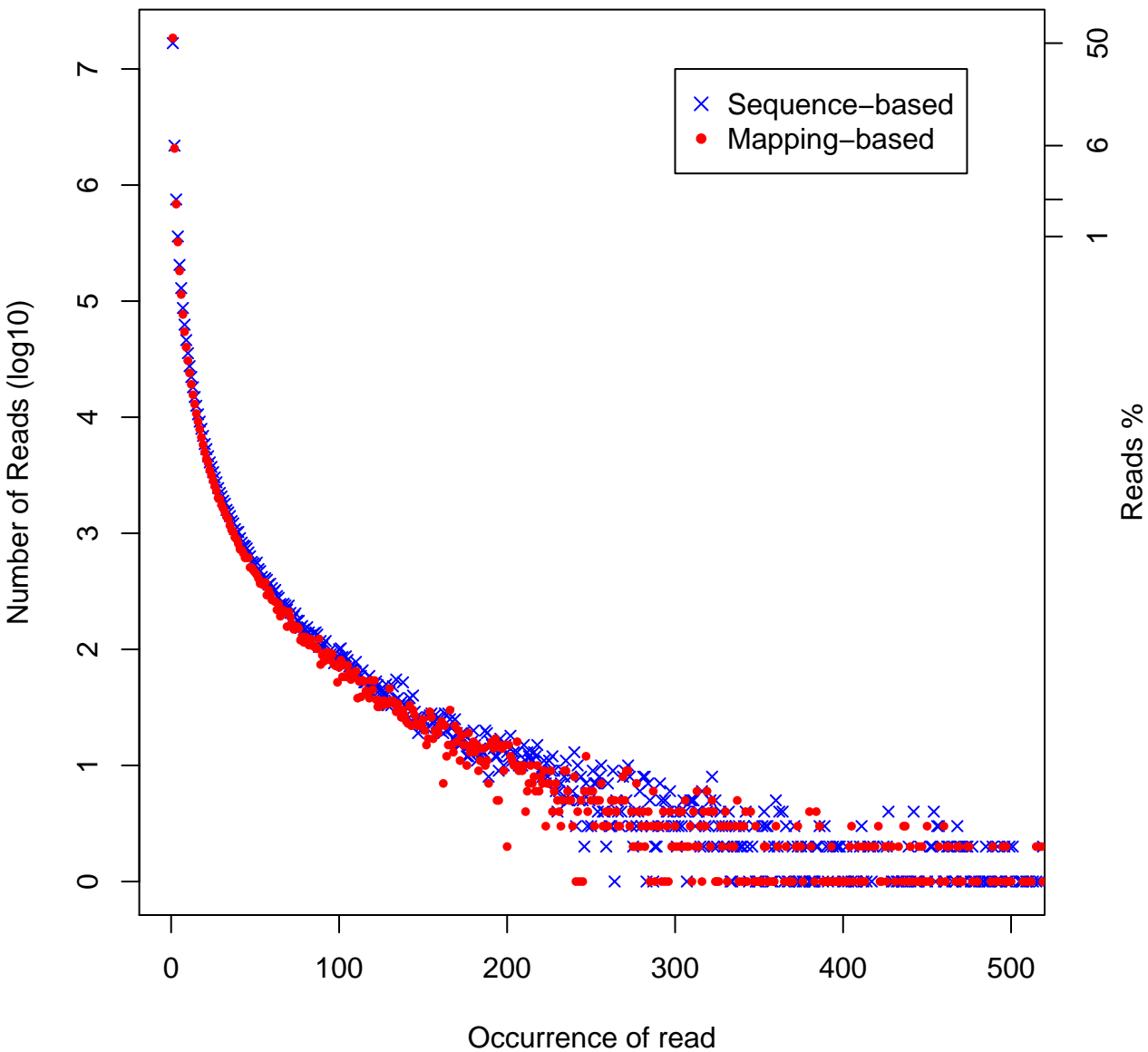

Supplement: S1 Data — This directory contains six subdirectories. The subdirectory “correlation” contains correlation heatmaps among all samples in diencephalon and telencephalon respectively. The subdirectory “mapping_stat” contains read mapping information on genomic features for each sample. The subdirectory “MDS” shows three dimensional MDS plots of the samples. The subdirectory “ReadDuplication” contains read duplication distributions for each sample. The subdirectory “ReadQuality” contains reads quality information for each sample plotted as both boxplots and heatmaps. The subdirectory “RPKMSaturation” contains information about read depth saturation for each sample as assessed by RPKM resamplings. All transcripts were divided into four quantiles based on their expression and a relative difference of observed and real RPKM values are plotted for each sample. (ZIP) [file pgen.1006840.s015.zip › RNASeq/ReadDuplication/4D_ATTACTCG-GTACTGAC_L00M_R1_001.DupRate_plot.pdf]

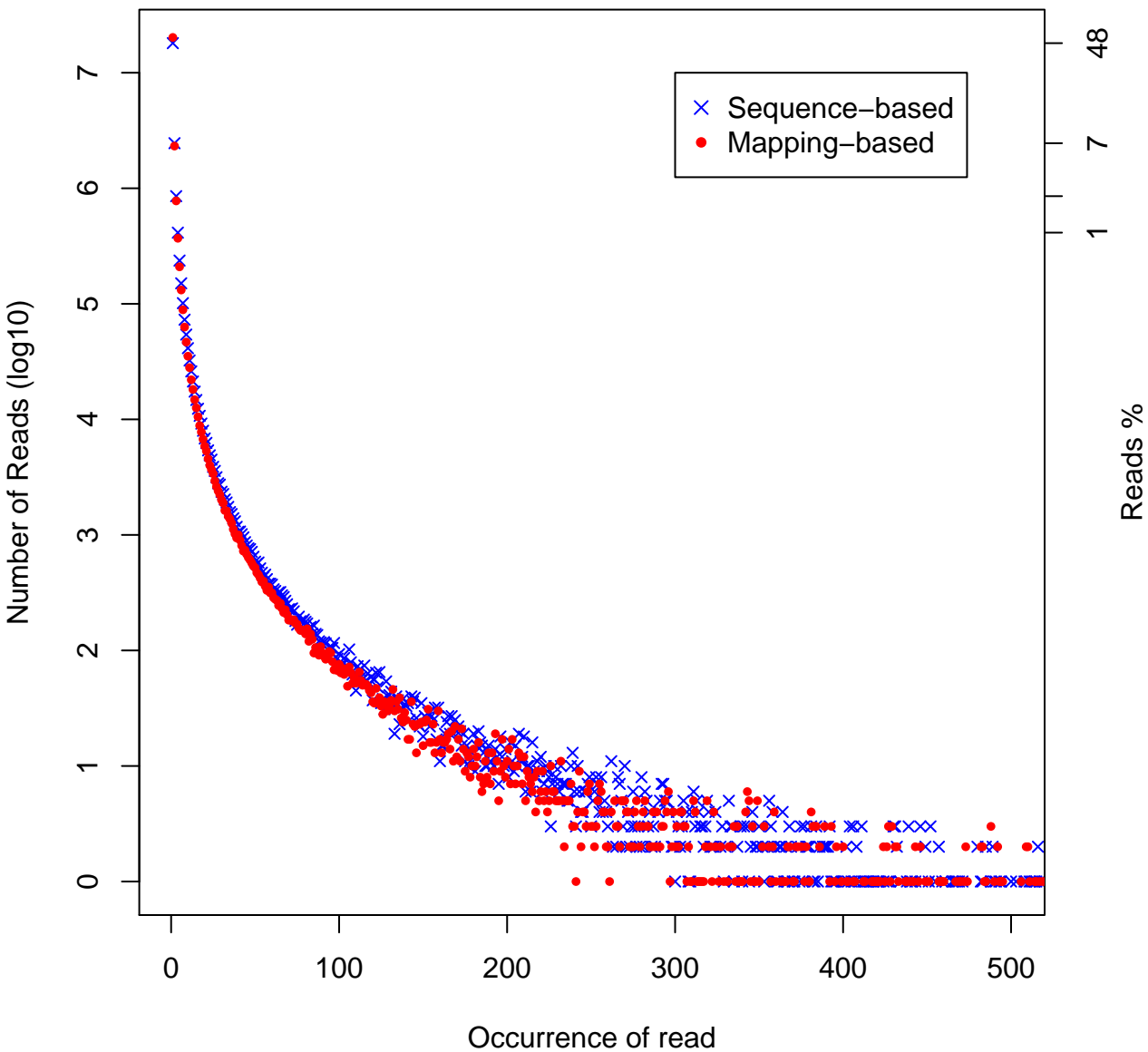

Supplement: S1 Data — This directory contains six subdirectories. The subdirectory “correlation” contains correlation heatmaps among all samples in diencephalon and telencephalon respectively. The subdirectory “mapping_stat” contains read mapping information on genomic features for each sample. The subdirectory “MDS” shows three dimensional MDS plots of the samples. The subdirectory “ReadDuplication” contains read duplication distributions for each sample. The subdirectory “ReadQuality” contains reads quality information for each sample plotted as both boxplots and heatmaps. The subdirectory “RPKMSaturation” contains information about read depth saturation for each sample as assessed by RPKM resamplings. All transcripts were divided into four quantiles based on their expression and a relative difference of observed and real RPKM values are plotted for each sample. (ZIP) [file pgen.1006840.s015.zip › RNASeq/ReadDuplication/4T_ATTACTCG-CAGGACGT_L00M_R1_001.DupRate_plot.pdf]

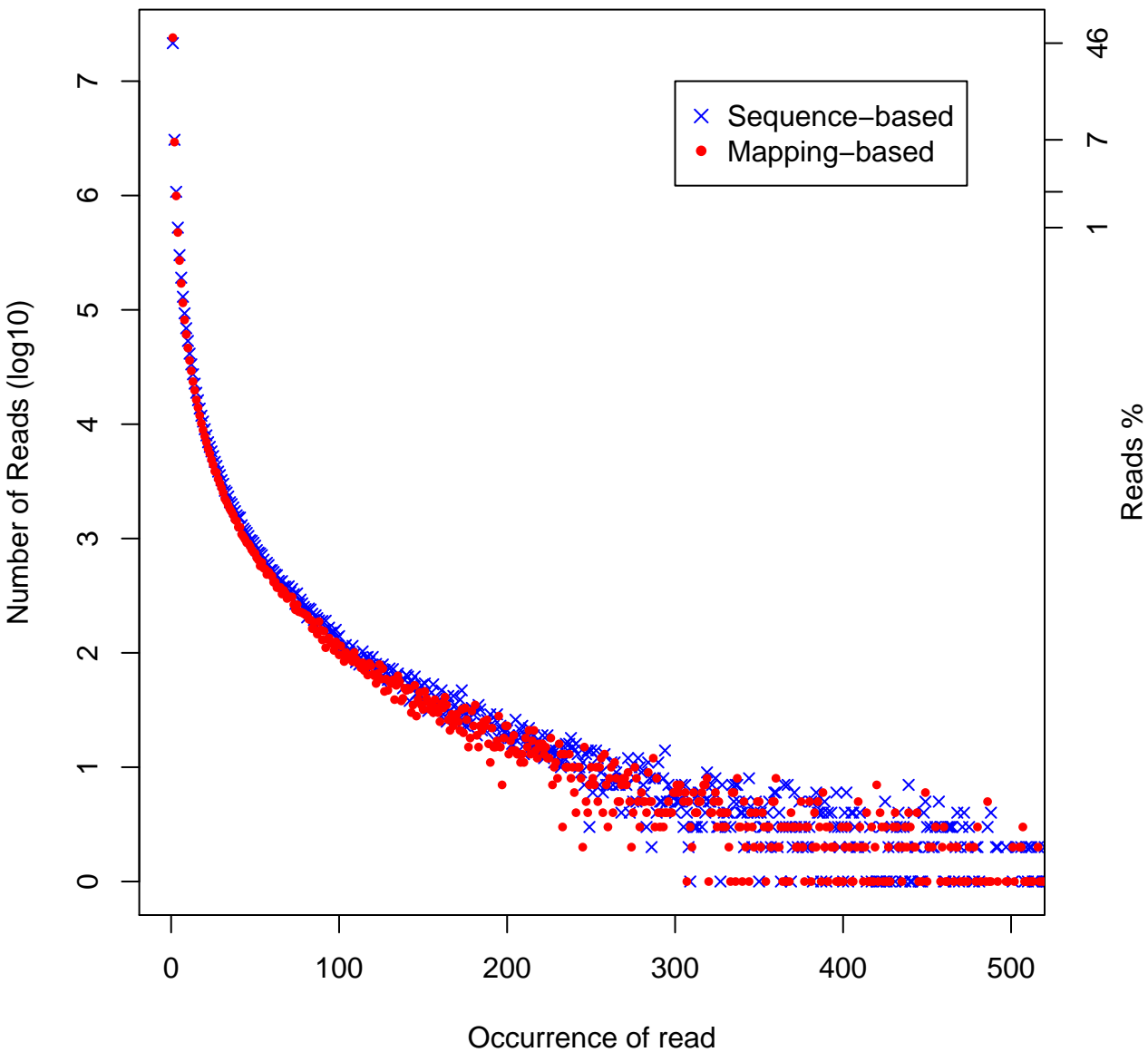

Supplement: S1 Data — This directory contains six subdirectories. The subdirectory “correlation” contains correlation heatmaps among all samples in diencephalon and telencephalon respectively. The subdirectory “mapping_stat” contains read mapping information on genomic features for each sample. The subdirectory “MDS” shows three dimensional MDS plots of the samples. The subdirectory “ReadDuplication” contains read duplication distributions for each sample. The subdirectory “ReadQuality” contains reads quality information for each sample plotted as both boxplots and heatmaps. The subdirectory “RPKMSaturation” contains information about read depth saturation for each sample as assessed by RPKM resamplings. All transcripts were divided into four quantiles based on their expression and a relative difference of observed and real RPKM values are plotted for each sample. (ZIP) [file pgen.1006840.s015.zip › RNASeq/ReadDuplication/5D_TCCGGAGA-ATAGAGGC_L00M_R1_001.DupRate_plot.pdf]

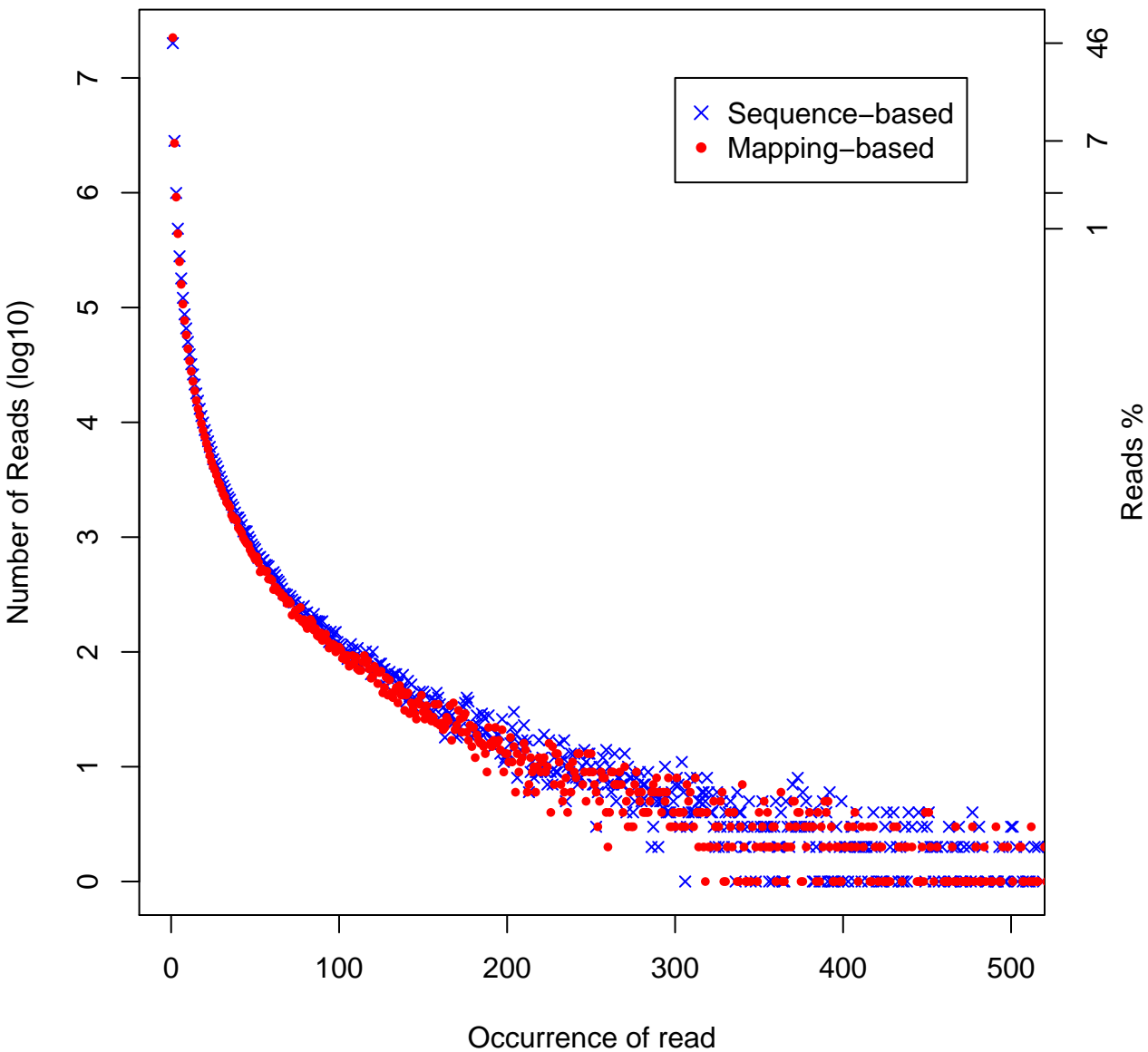

Supplement: S1 Data — This directory contains six subdirectories. The subdirectory “correlation” contains correlation heatmaps among all samples in diencephalon and telencephalon respectively. The subdirectory “mapping_stat” contains read mapping information on genomic features for each sample. The subdirectory “MDS” shows three dimensional MDS plots of the samples. The subdirectory “ReadDuplication” contains read duplication distributions for each sample. The subdirectory “ReadQuality” contains reads quality information for each sample plotted as both boxplots and heatmaps. The subdirectory “RPKMSaturation” contains information about read depth saturation for each sample as assessed by RPKM resamplings. All transcripts were divided into four quantiles based on their expression and a relative difference of observed and real RPKM values are plotted for each sample. (ZIP) [file pgen.1006840.s015.zip › RNASeq/ReadDuplication/5T_TCCGGAGA-TATAGCCT_L00M_R1_001.DupRate_plot.pdf]

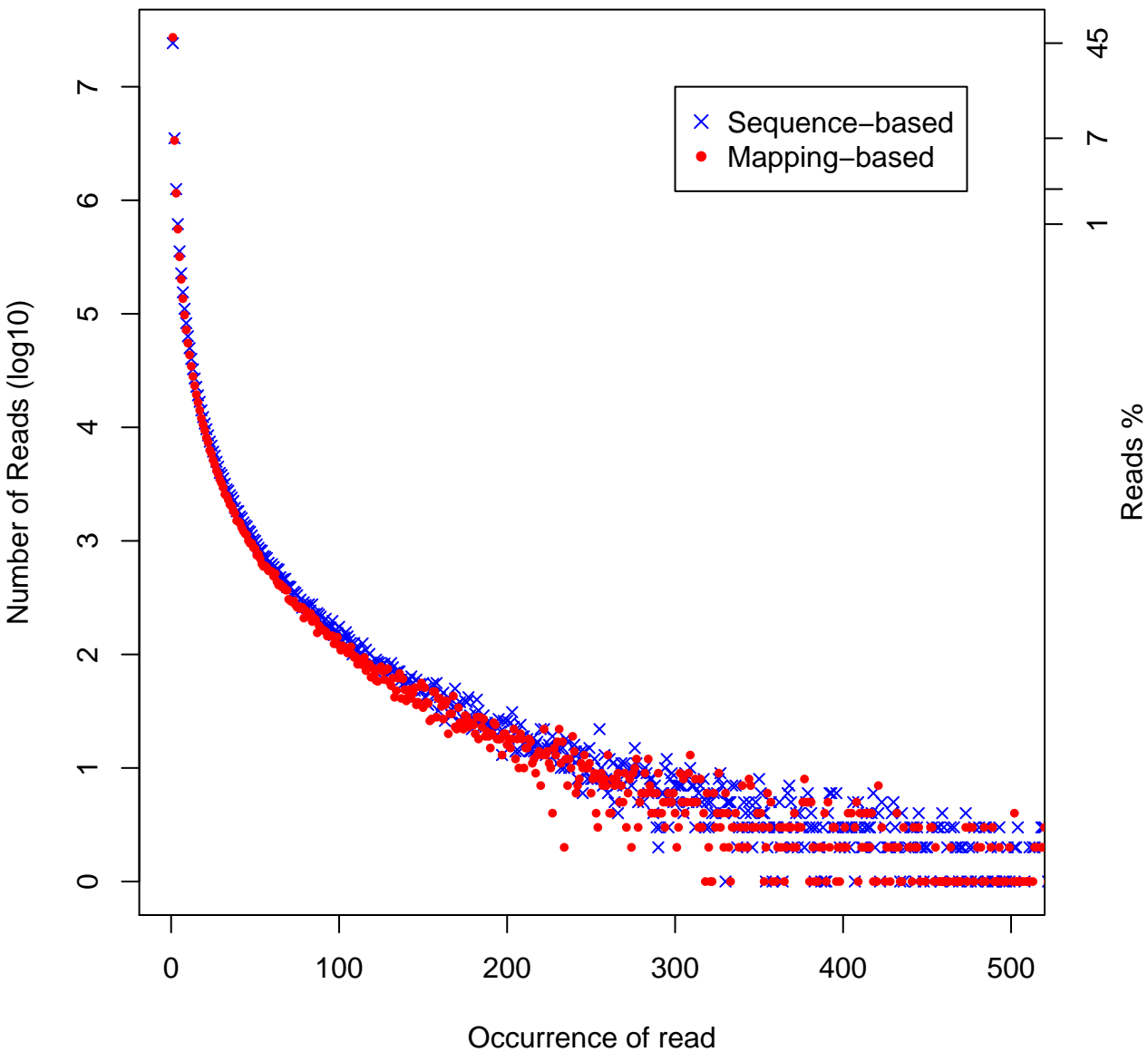

Supplement: S1 Data — This directory contains six subdirectories. The subdirectory “correlation” contains correlation heatmaps among all samples in diencephalon and telencephalon respectively. The subdirectory “mapping_stat” contains read mapping information on genomic features for each sample. The subdirectory “MDS” shows three dimensional MDS plots of the samples. The subdirectory “ReadDuplication” contains read duplication distributions for each sample. The subdirectory “ReadQuality” contains reads quality information for each sample plotted as both boxplots and heatmaps. The subdirectory “RPKMSaturation” contains information about read depth saturation for each sample as assessed by RPKM resamplings. All transcripts were divided into four quantiles based on their expression and a relative difference of observed and real RPKM values are plotted for each sample. (ZIP) [file pgen.1006840.s015.zip › RNASeq/ReadDuplication/7D_TCCGGAGA-GGCTCTGA_L00M_R1_001.DupRate_plot.pdf]

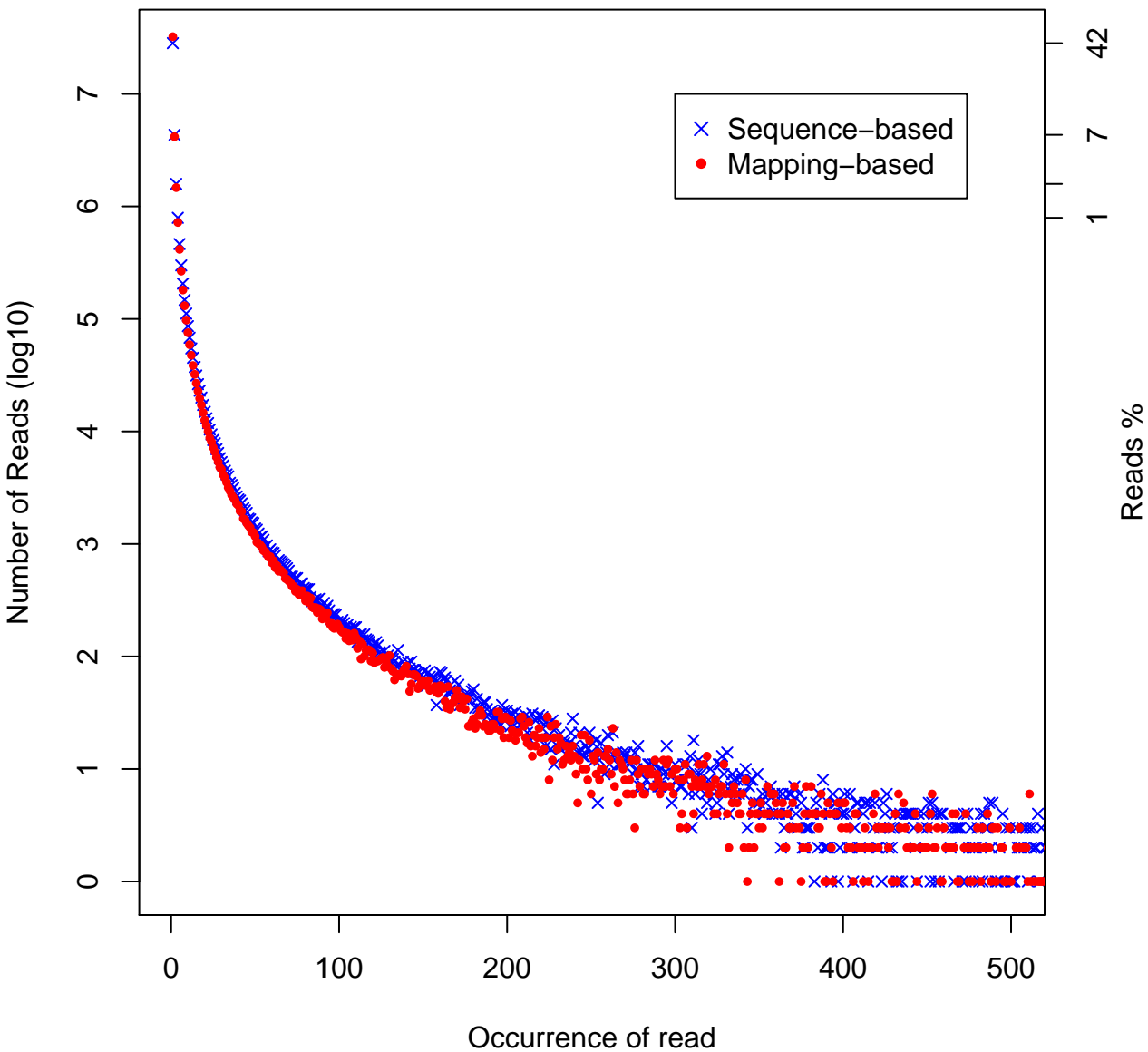

Supplement: S1 Data — This directory contains six subdirectories. The subdirectory “correlation” contains correlation heatmaps among all samples in diencephalon and telencephalon respectively. The subdirectory “mapping_stat” contains read mapping information on genomic features for each sample. The subdirectory “MDS” shows three dimensional MDS plots of the samples. The subdirectory “ReadDuplication” contains read duplication distributions for each sample. The subdirectory “ReadQuality” contains reads quality information for each sample plotted as both boxplots and heatmaps. The subdirectory “RPKMSaturation” contains information about read depth saturation for each sample as assessed by RPKM resamplings. All transcripts were divided into four quantiles based on their expression and a relative difference of observed and real RPKM values are plotted for each sample. (ZIP) [file pgen.1006840.s015.zip › RNASeq/ReadDuplication/7T_TCCGGAGA-CCTATCCT_L00M_R1_001.DupRate_plot.pdf]

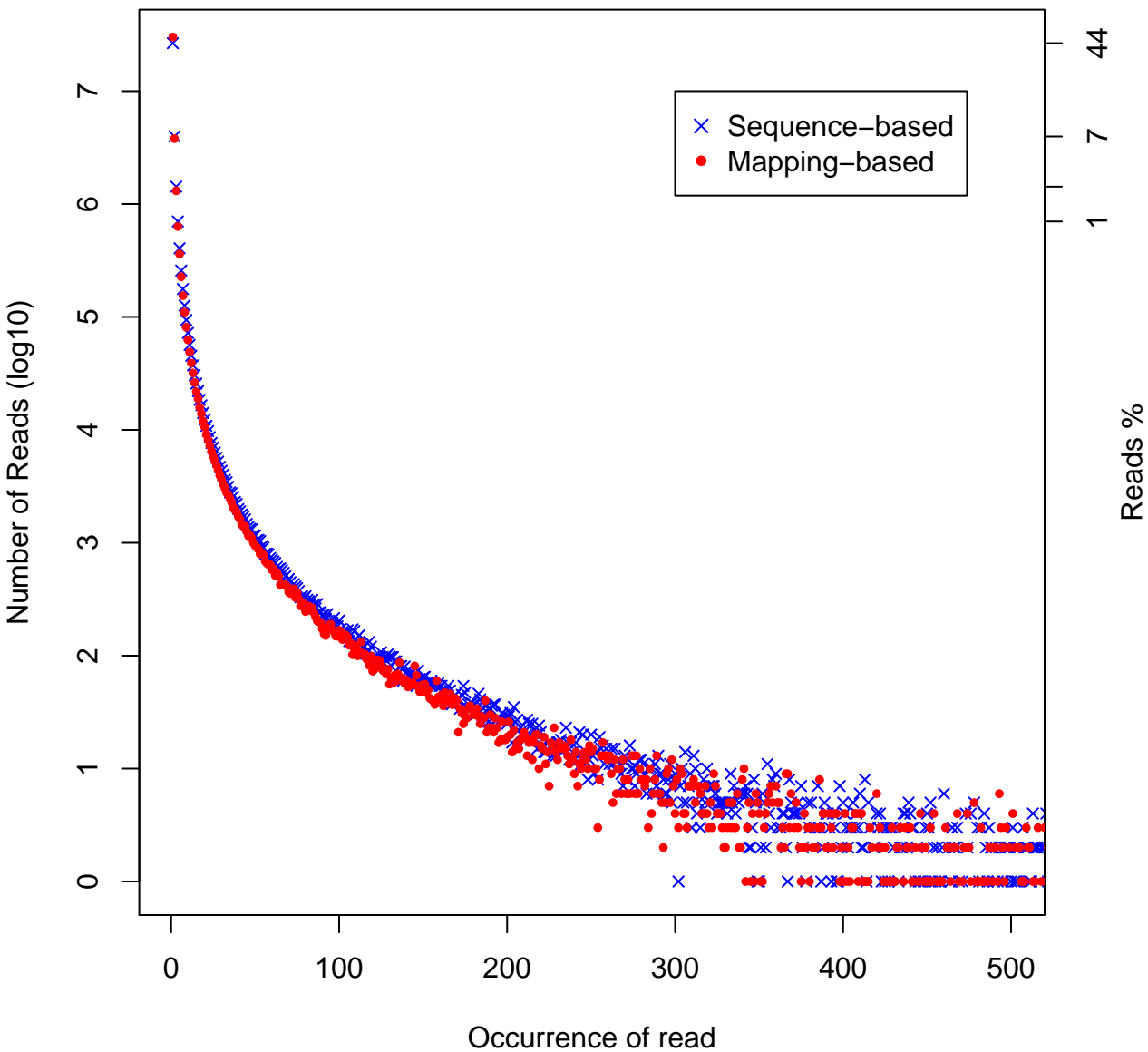

Supplement: S1 Data — This directory contains six subdirectories. The subdirectory “correlation” contains correlation heatmaps among all samples in diencephalon and telencephalon respectively. The subdirectory “mapping_stat” contains read mapping information on genomic features for each sample. The subdirectory “MDS” shows three dimensional MDS plots of the samples. The subdirectory “ReadDuplication” contains read duplication distributions for each sample. The subdirectory “ReadQuality” contains reads quality information for each sample plotted as both boxplots and heatmaps. The subdirectory “RPKMSaturation” contains information about read depth saturation for each sample as assessed by RPKM resamplings. All transcripts were divided into four quantiles based on their expression and a relative difference of observed and real RPKM values are plotted for each sample. (ZIP) [file pgen.1006840.s015.zip › RNASeq/ReadDuplication/8D_TCCGGAGA-TAATCTTA_L00M_R1_001.DupRate_plot.pdf]

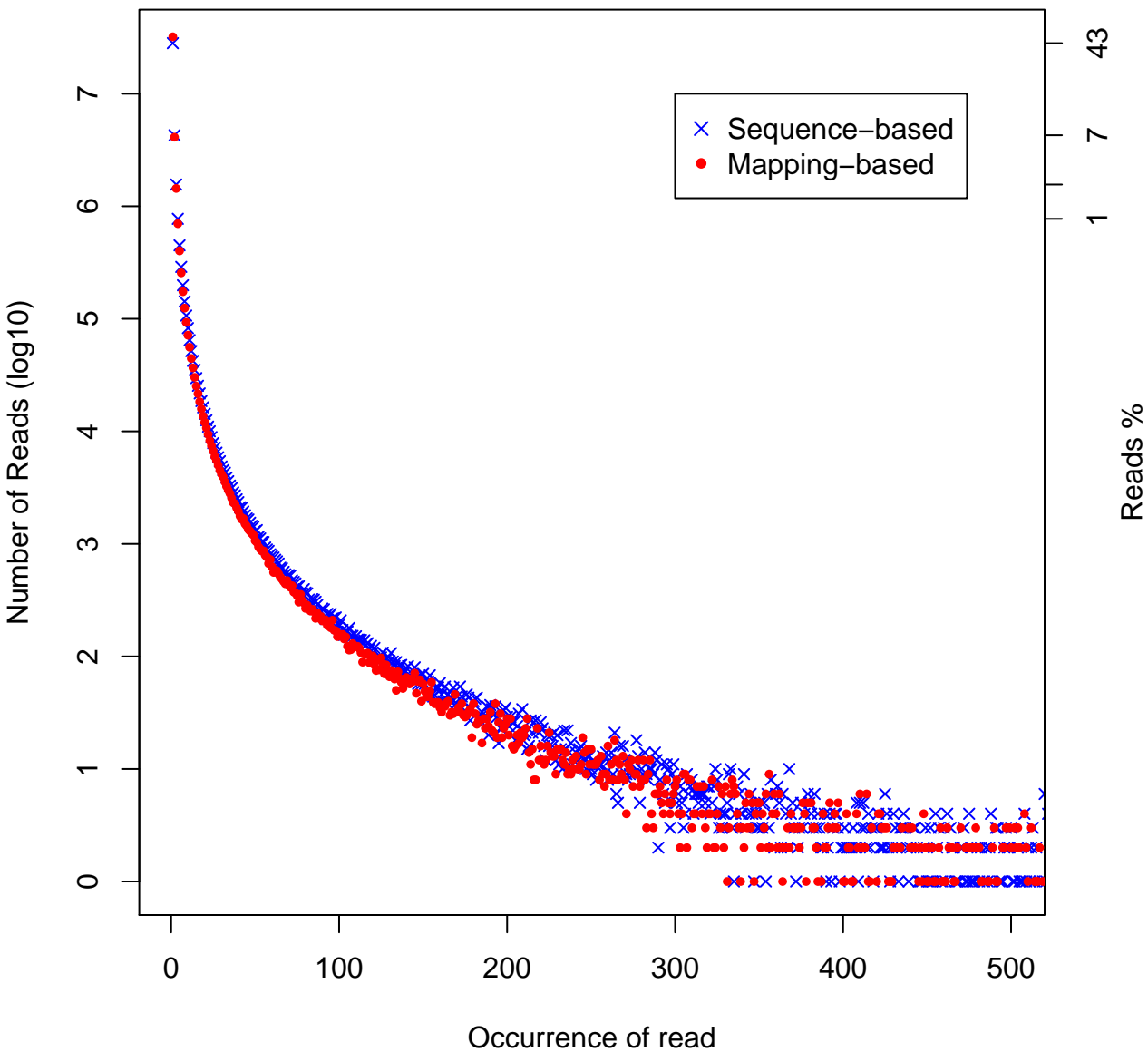

Supplement: S1 Data — This directory contains six subdirectories. The subdirectory “correlation” contains correlation heatmaps among all samples in diencephalon and telencephalon respectively. The subdirectory “mapping_stat” contains read mapping information on genomic features for each sample. The subdirectory “MDS” shows three dimensional MDS plots of the samples. The subdirectory “ReadDuplication” contains read duplication distributions for each sample. The subdirectory “ReadQuality” contains reads quality information for each sample plotted as both boxplots and heatmaps. The subdirectory “RPKMSaturation” contains information about read depth saturation for each sample as assessed by RPKM resamplings. All transcripts were divided into four quantiles based on their expression and a relative difference of observed and real RPKM values are plotted for each sample. (ZIP) [file pgen.1006840.s015.zip › RNASeq/ReadDuplication/8T_TCCGGAGA-AGGCGAAG_L00M_R1_001.DupRate_plot.pdf]

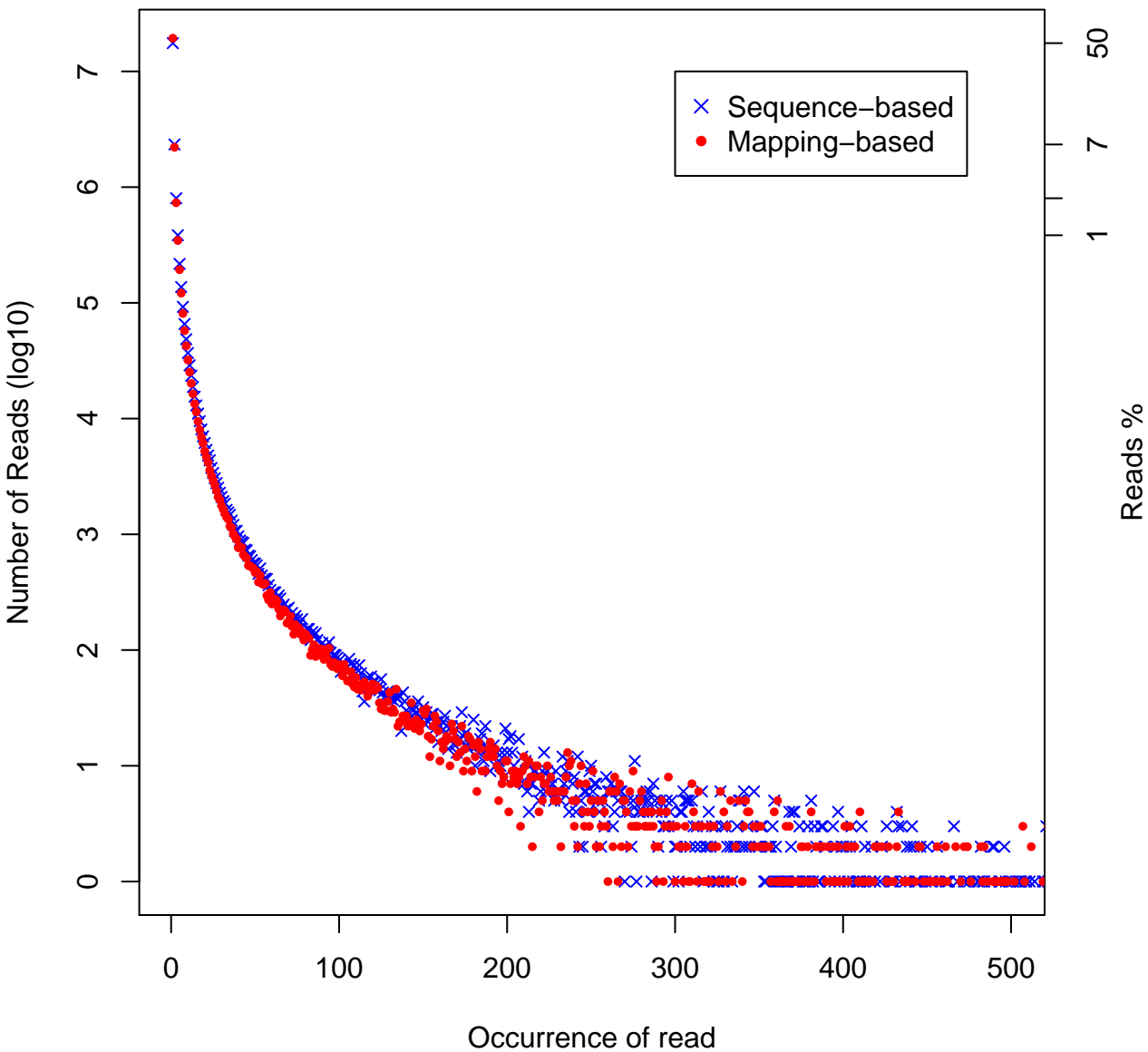

Supplement: S1 Data — This directory contains six subdirectories. The subdirectory “correlation” contains correlation heatmaps among all samples in diencephalon and telencephalon respectively. The subdirectory “mapping_stat” contains read mapping information on genomic features for each sample. The subdirectory “MDS” shows three dimensional MDS plots of the samples. The subdirectory “ReadDuplication” contains read duplication distributions for each sample. The subdirectory “ReadQuality” contains reads quality information for each sample plotted as both boxplots and heatmaps. The subdirectory “RPKMSaturation” contains information about read depth saturation for each sample as assessed by RPKM resamplings. All transcripts were divided into four quantiles based on their expression and a relative difference of observed and real RPKM values are plotted for each sample. (ZIP) [file pgen.1006840.s015.zip › RNASeq/ReadDuplication/9D_TCCGGAGA-GTACTGAC_L00M_R1_001.DupRate_plot.pdf]

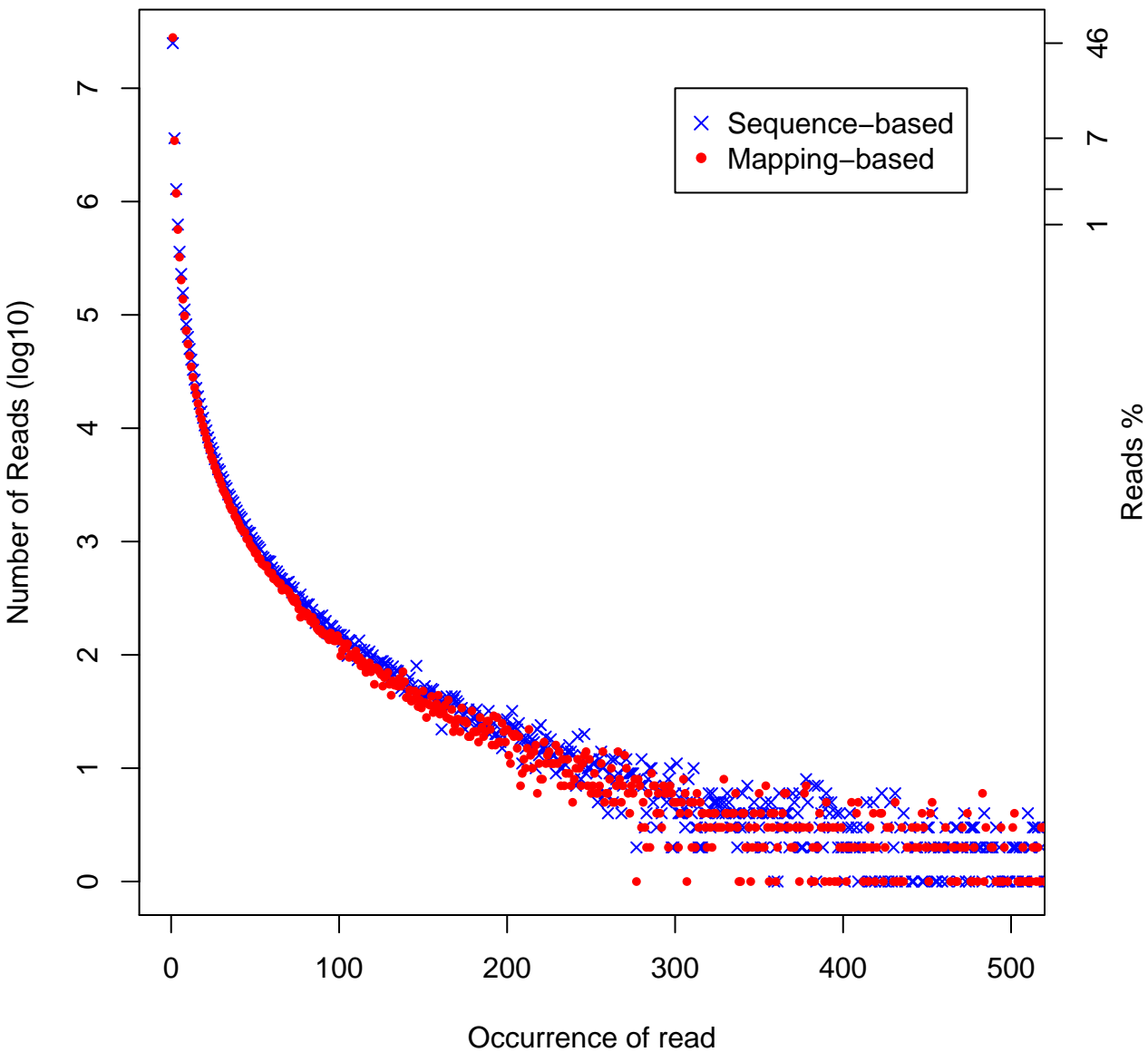

Supplement: S1 Data — This directory contains six subdirectories. The subdirectory “correlation” contains correlation heatmaps among all samples in diencephalon and telencephalon respectively. The subdirectory “mapping_stat” contains read mapping information on genomic features for each sample. The subdirectory “MDS” shows three dimensional MDS plots of the samples. The subdirectory “ReadDuplication” contains read duplication distributions for each sample. The subdirectory “ReadQuality” contains reads quality information for each sample plotted as both boxplots and heatmaps. The subdirectory “RPKMSaturation” contains information about read depth saturation for each sample as assessed by RPKM resamplings. All transcripts were divided into four quantiles based on their expression and a relative difference of observed and real RPKM values are plotted for each sample. (ZIP) [file pgen.1006840.s015.zip › RNASeq/ReadDuplication/9T_TCCGGAGA-CAGGACGT_L00M_R1_001.DupRate_plot.pdf]

Phred Quality Score

40  
35  
30

Position of Read(5'→3')

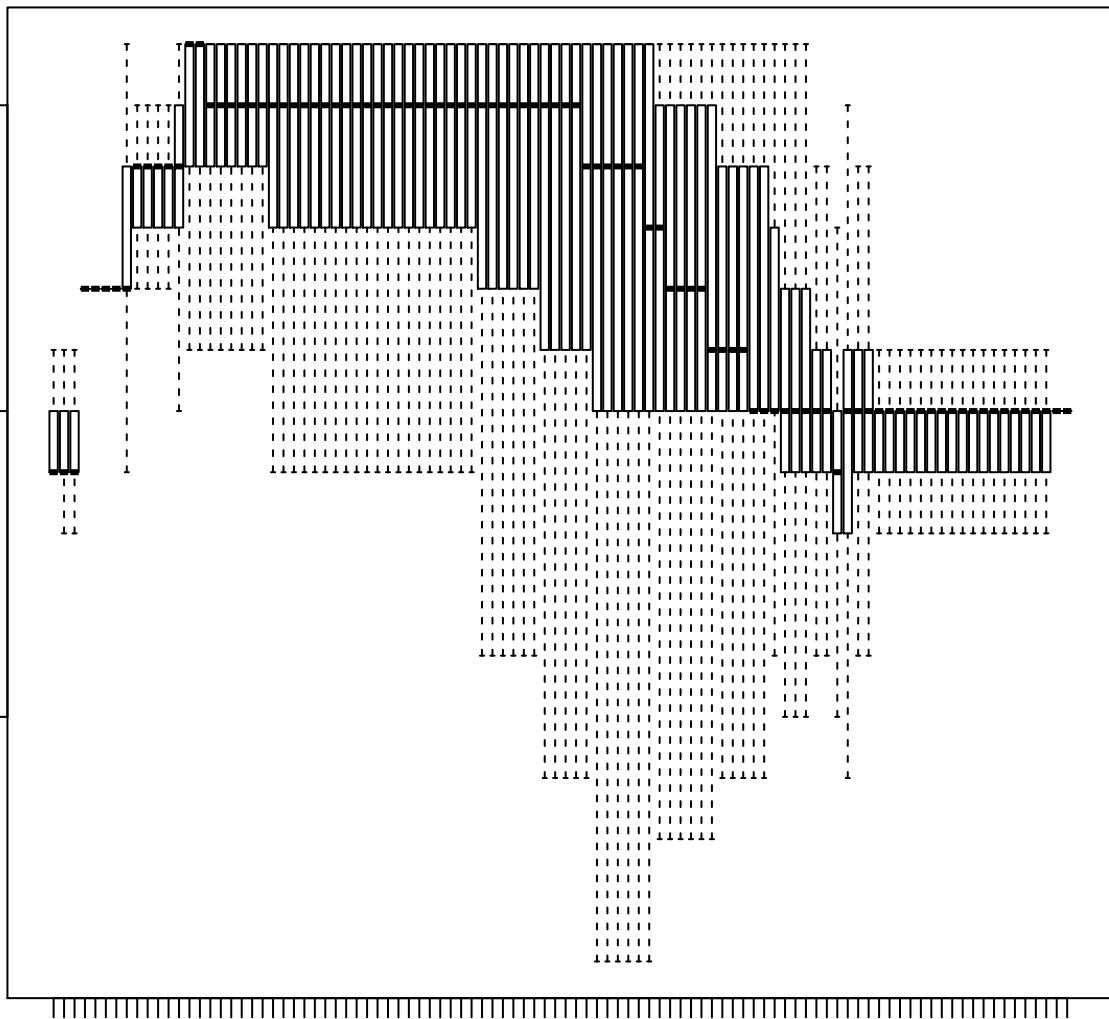

Supplement: S1 Data — This directory contains six subdirectories. The subdirectory “correlation” contains correlation heatmaps among all samples in diencephalon and telencephalon respectively. The subdirectory “mapping_stat” contains read mapping information on genomic features for each sample. The subdirectory “MDS” shows three dimensional MDS plots of the samples. The subdirectory “ReadDuplication” contains read duplication distributions for each sample. The subdirectory “ReadQuality” contains reads quality information for each sample plotted as both boxplots and heatmaps. The subdirectory “RPKMSaturation” contains information about read depth saturation for each sample as assessed by RPKM resamplings. All transcripts were divided into four quantiles based on their expression and a relative difference of observed and real RPKM values are plotted for each sample. (ZIP) [file pgen.1006840.s015.zip › RNASeq/ReadQuality/10D_CGCTCATT-ATAGAGGC_L00M_R1_001.readquality.qual.boxplot.pdf]

Phred Quality Score

40

35

30

Position of Read(5'→3')

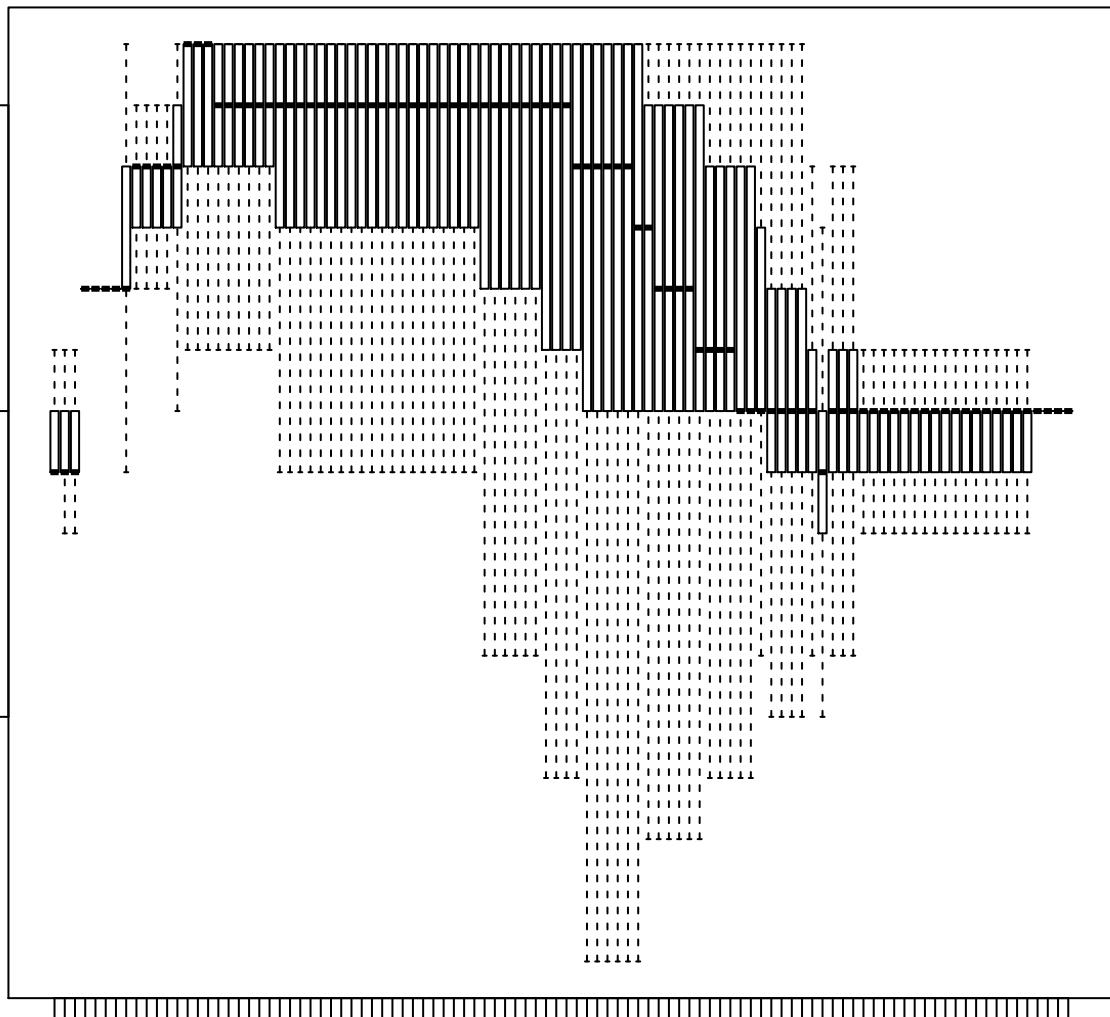

Supplement: S1 Data — This directory contains six subdirectories. The subdirectory “correlation” contains correlation heatmaps among all samples in diencephalon and telencephalon respectively. The subdirectory “mapping_stat” contains read mapping information on genomic features for each sample. The subdirectory “MDS” shows three dimensional MDS plots of the samples. The subdirectory “ReadDuplication” contains read duplication distributions for each sample. The subdirectory “ReadQuality” contains reads quality information for each sample plotted as both boxplots and heatmaps. The subdirectory “RPKMSaturation” contains information about read depth saturation for each sample as assessed by RPKM resamplings. All transcripts were divided into four quantiles based on their expression and a relative difference of observed and real RPKM values are plotted for each sample. (ZIP) [file pgen.1006840.s015.zip › RNASeq/ReadQuality/10T_CGCTCATT-TATAGCCT_L00M_R1_001.readquality.qual.boxplot.pdf]

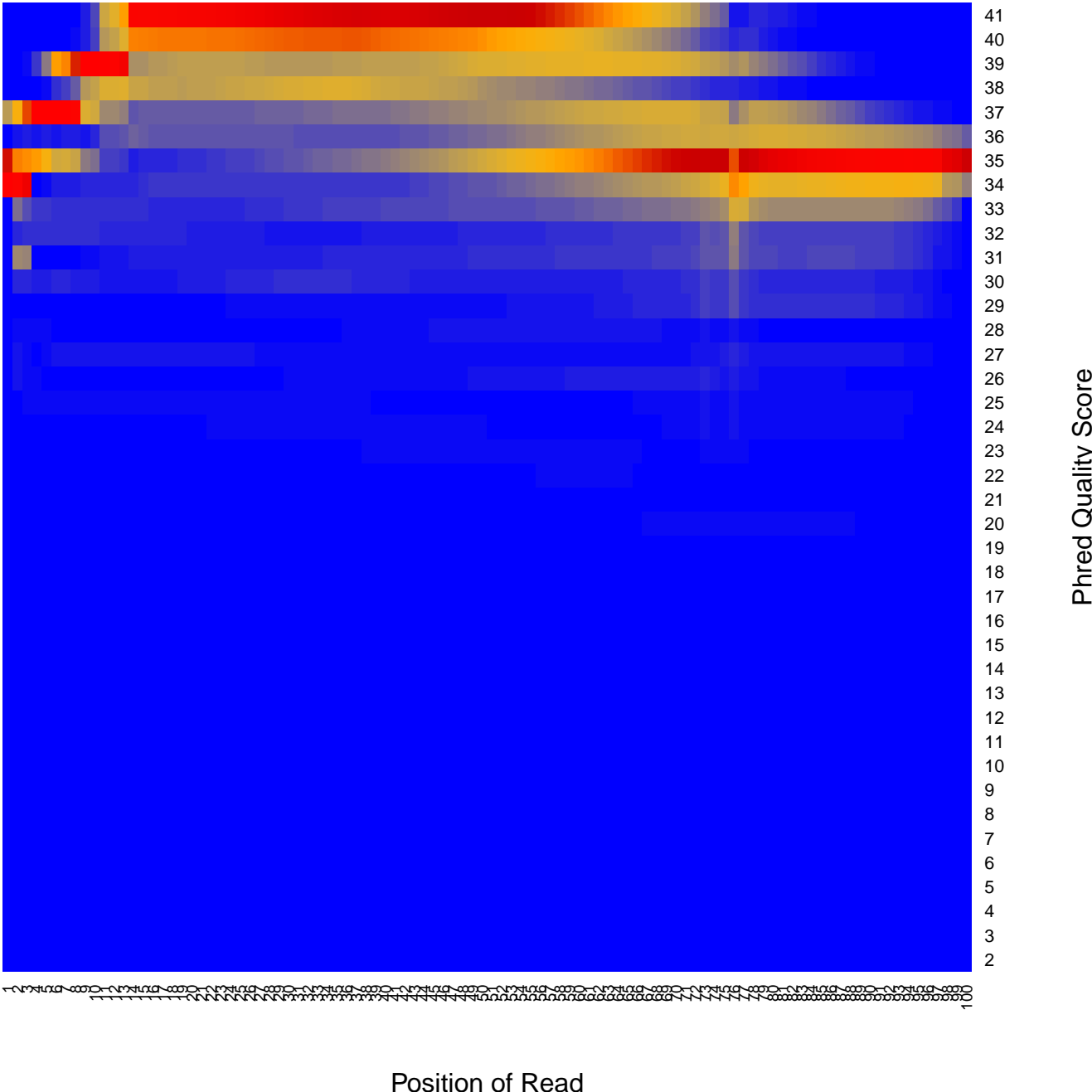

Supplement: S1 Data — This directory contains six subdirectories. The subdirectory “correlation” contains correlation heatmaps among all samples in diencephalon and telencephalon respectively. The subdirectory “mapping_stat” contains read mapping information on genomic features for each sample. The subdirectory “MDS” shows three dimensional MDS plots of the samples. The subdirectory “ReadDuplication” contains read duplication distributions for each sample. The subdirectory “ReadQuality” contains reads quality information for each sample plotted as both boxplots and heatmaps. The subdirectory “RPKMSaturation” contains information about read depth saturation for each sample as assessed by RPKM resamplings. All transcripts were divided into four quantiles based on their expression and a relative difference of observed and real RPKM values are plotted for each sample. (ZIP) [file pgen.1006840.s015.zip › RNASeq/ReadQuality/10T_CGCTCATT-TATAGCCT_L00M_R1_001.readquality.qual.heatmap.pdf]

Phred Quality Score

40  
35  
30

Position of Read(5'→3')

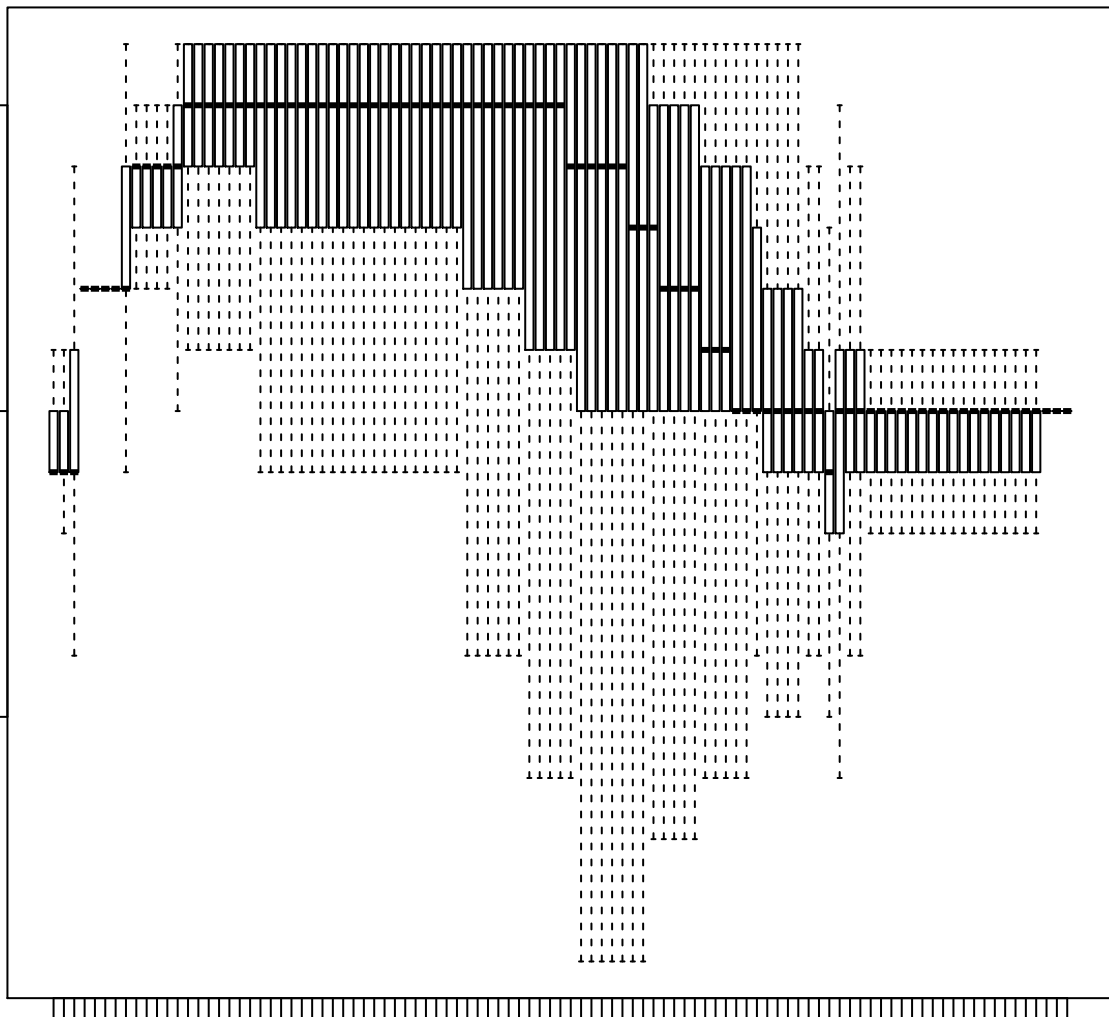

Supplement: S1 Data — This directory contains six subdirectories. The subdirectory “correlation” contains correlation heatmaps among all samples in diencephalon and telencephalon respectively. The subdirectory “mapping_stat” contains read mapping information on genomic features for each sample. The subdirectory “MDS” shows three dimensional MDS plots of the samples. The subdirectory “ReadDuplication” contains read duplication distributions for each sample. The subdirectory “ReadQuality” contains reads quality information for each sample plotted as both boxplots and heatmaps. The subdirectory “RPKMSaturation” contains information about read depth saturation for each sample as assessed by RPKM resamplings. All transcripts were divided into four quantiles based on their expression and a relative difference of observed and real RPKM values are plotted for each sample. (ZIP) [file pgen.1006840.s015.zip › RNASeq/ReadQuality/11D_CGCTCATT-GGCTCTGA_L00M_R1_001.readquality.qual.boxplot.pdf]

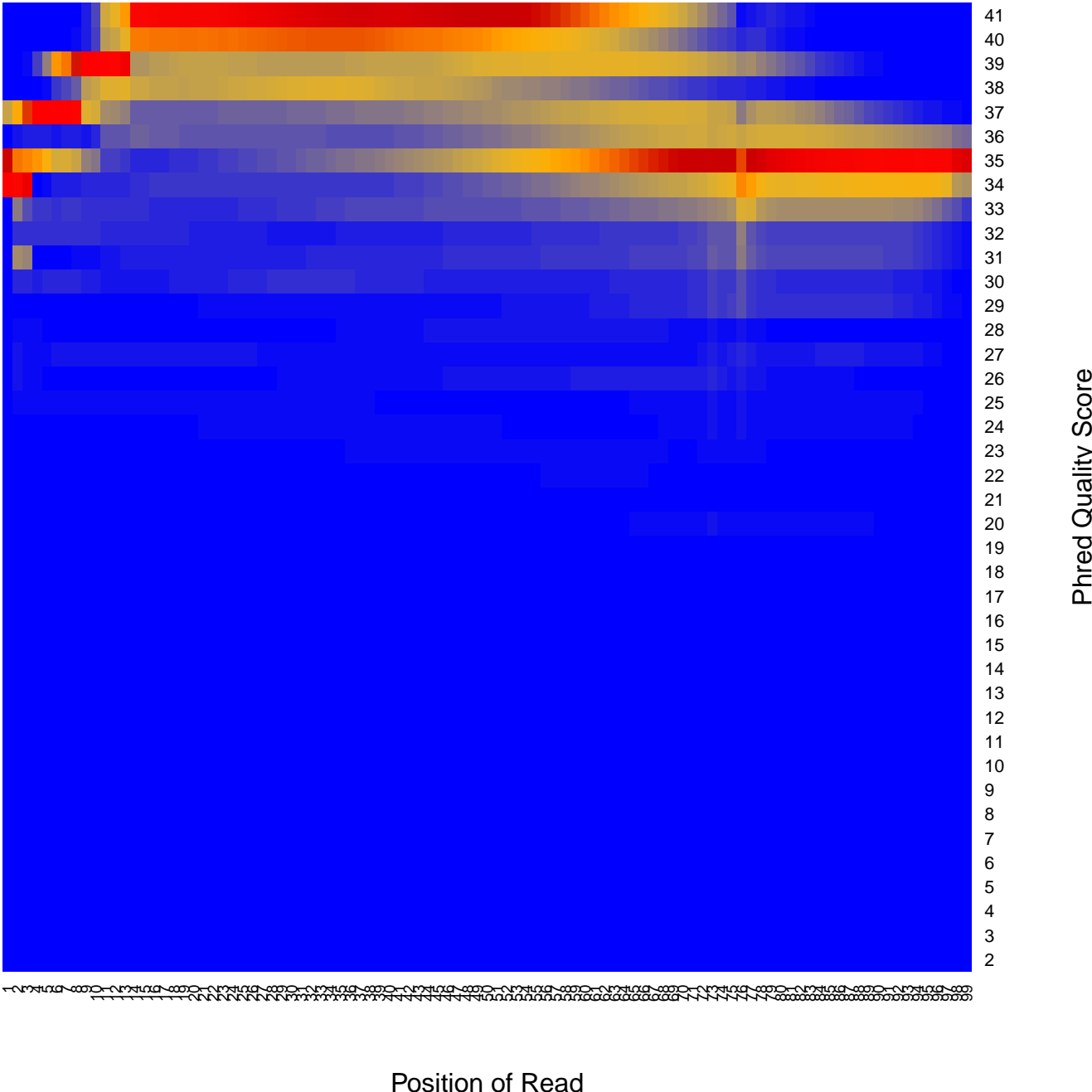

Supplement: S1 Data — This directory contains six subdirectories. The subdirectory “correlation” contains correlation heatmaps among all samples in diencephalon and telencephalon respectively. The subdirectory “mapping_stat” contains read mapping information on genomic features for each sample. The subdirectory “MDS” shows three dimensional MDS plots of the samples. The subdirectory “ReadDuplication” contains read duplication distributions for each sample. The subdirectory “ReadQuality” contains reads quality information for each sample plotted as both boxplots and heatmaps. The subdirectory “RPKMSaturation” contains information about read depth saturation for each sample as assessed by RPKM resamplings. All transcripts were divided into four quantiles based on their expression and a relative difference of observed and real RPKM values are plotted for each sample. (ZIP) [file pgen.1006840.s015.zip › RNASeq/ReadQuality/11D_CGCTCATT-GGCTCTGA_L00M_R1_001.readquality.qual.heatmap.pdf]

Phred Quality Score

30  
35  
40

Position of Read(5'→3')

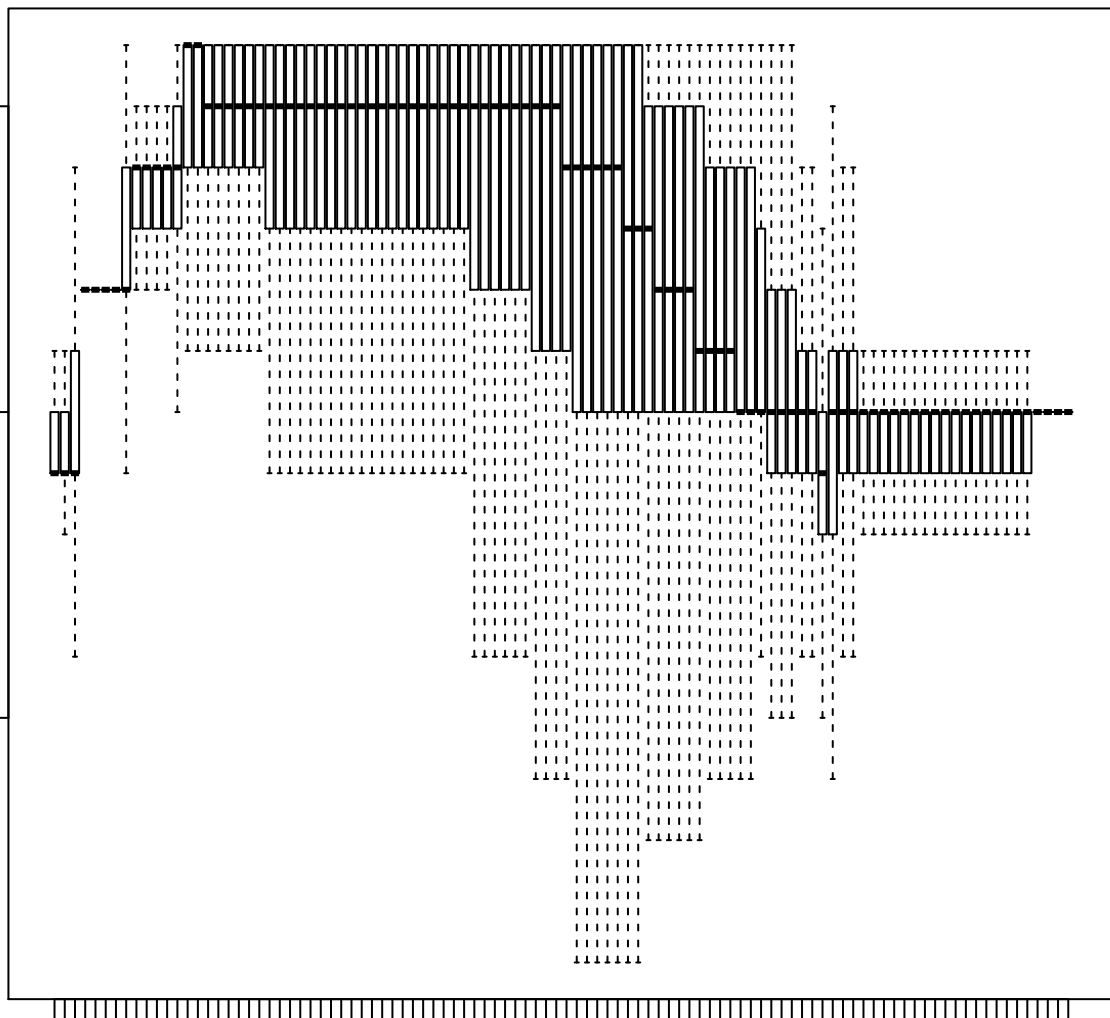

Supplement: S1 Data — This directory contains six subdirectories. The subdirectory “correlation” contains correlation heatmaps among all samples in diencephalon and telencephalon respectively. The subdirectory “mapping_stat” contains read mapping information on genomic features for each sample. The subdirectory “MDS” shows three dimensional MDS plots of the samples. The subdirectory “ReadDuplication” contains read duplication distributions for each sample. The subdirectory “ReadQuality” contains reads quality information for each sample plotted as both boxplots and heatmaps. The subdirectory “RPKMSaturation” contains information about read depth saturation for each sample as assessed by RPKM resamplings. All transcripts were divided into four quantiles based on their expression and a relative difference of observed and real RPKM values are plotted for each sample. (ZIP) [file pgen.1006840.s015.zip › RNASeq/ReadQuality/11T_CGCTCATT-CCTATCCT_L00M_R1_001.readquality.qual.boxplot.pdf]

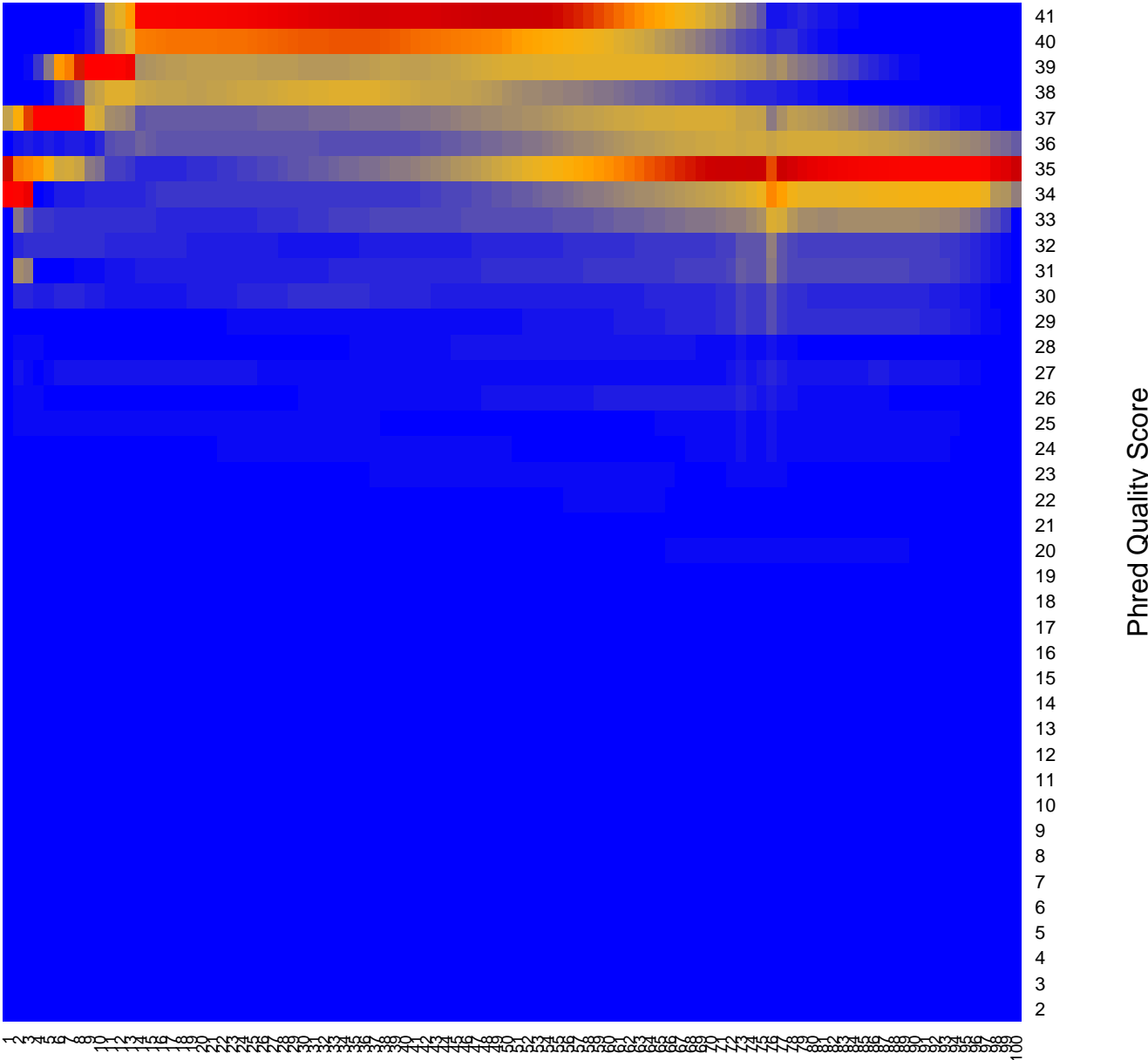

Position of Read

Supplement: S1 Data — This directory contains six subdirectories. The subdirectory “correlation” contains correlation heatmaps among all samples in diencephalon and telencephalon respectively. The subdirectory “mapping_stat” contains read mapping information on genomic features for each sample. The subdirectory “MDS” shows three dimensional MDS plots of the samples. The subdirectory “ReadDuplication” contains read duplication distributions for each sample. The subdirectory “ReadQuality” contains reads quality information for each sample plotted as both boxplots and heatmaps. The subdirectory “RPKMSaturation” contains information about read depth saturation for each sample as assessed by RPKM resamplings. All transcripts were divided into four quantiles based on their expression and a relative difference of observed and real RPKM values are plotted for each sample. (ZIP) [file pgen.1006840.s015.zip › RNASeq/ReadQuality/11T_CGCTCATT-CCTATCCT_L00M_R1_001.readquality.qual.heatmap.pdf]

Phred Quality Score

40  
35  
30

Position of Read(5'→3')

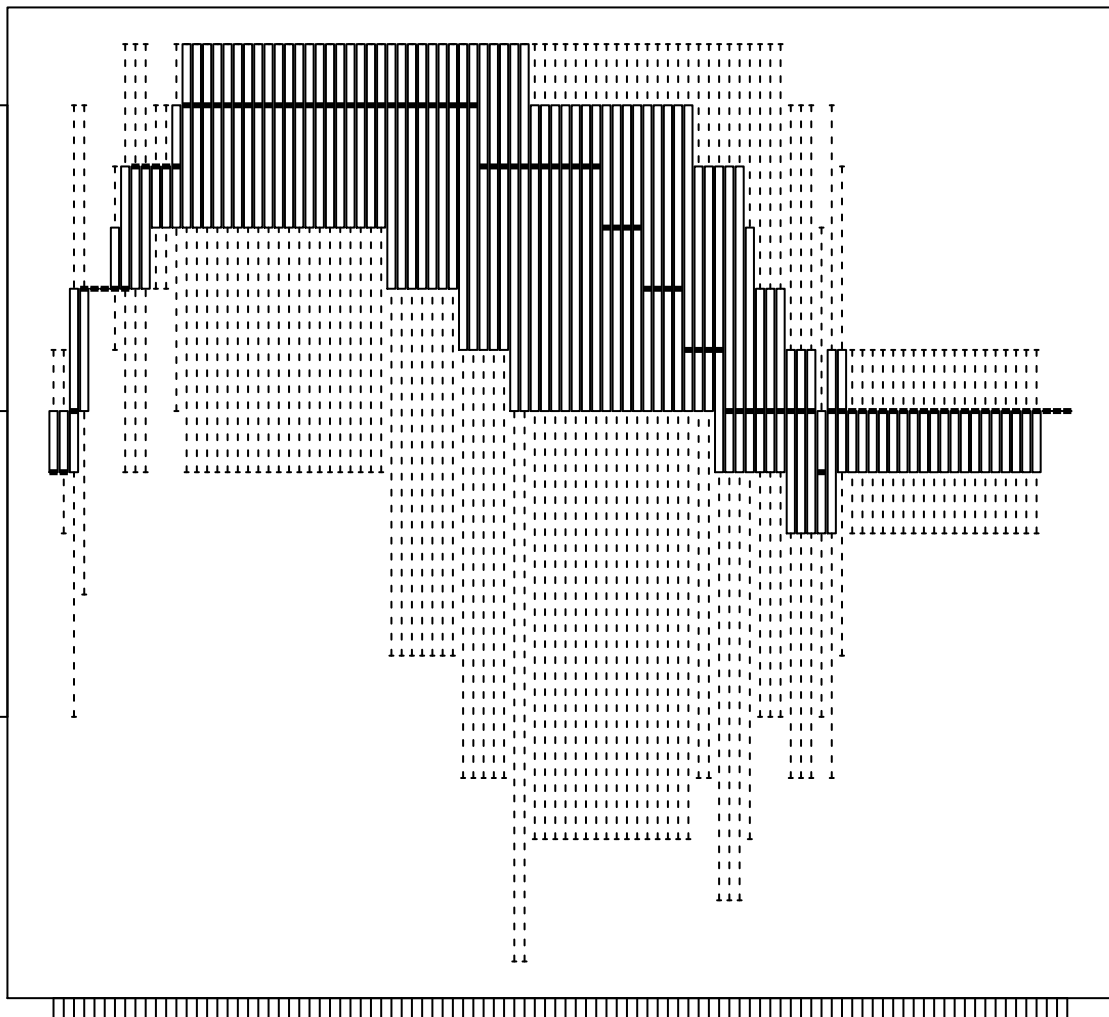

Supplement: S1 Data — This directory contains six subdirectories. The subdirectory “correlation” contains correlation heatmaps among all samples in diencephalon and telencephalon respectively. The subdirectory “mapping_stat” contains read mapping information on genomic features for each sample. The subdirectory “MDS” shows three dimensional MDS plots of the samples. The subdirectory “ReadDuplication” contains read duplication distributions for each sample. The subdirectory “ReadQuality” contains reads quality information for each sample plotted as both boxplots and heatmaps. The subdirectory “RPKMSaturation” contains information about read depth saturation for each sample as assessed by RPKM resamplings. All transcripts were divided into four quantiles based on their expression and a relative difference of observed and real RPKM values are plotted for each sample. (ZIP) [file pgen.1006840.s015.zip › RNASeq/ReadQuality/12D_CGCTCATT-TAATCTTA_L00M_R1_001.readquality.qual.boxplot.pdf]

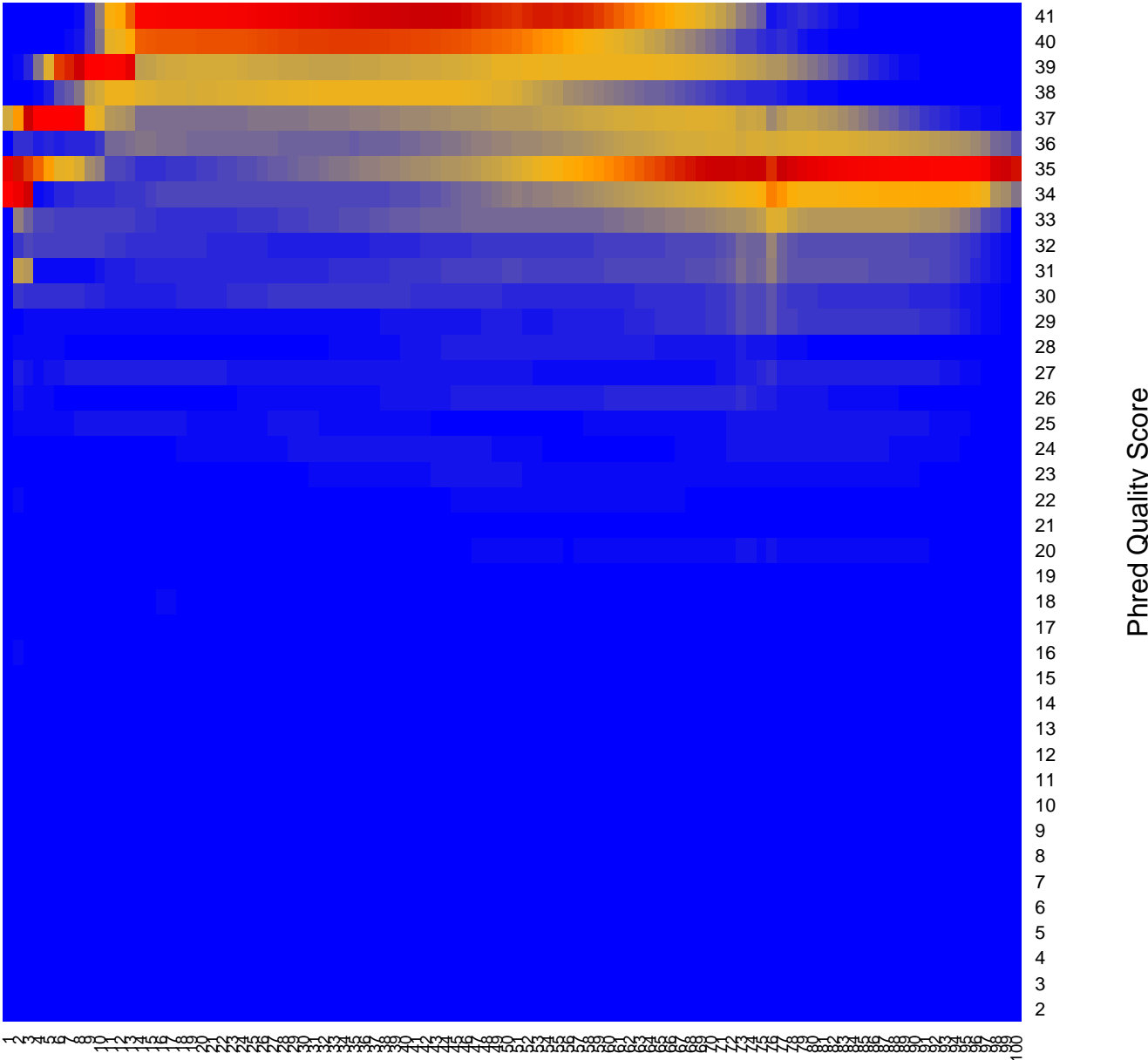

Position of Read

Supplement: S1 Data — This directory contains six subdirectories. The subdirectory “correlation” contains correlation heatmaps among all samples in diencephalon and telencephalon respectively. The subdirectory “mapping_stat” contains read mapping information on genomic features for each sample. The subdirectory “MDS” shows three dimensional MDS plots of the samples. The subdirectory “ReadDuplication” contains read duplication distributions for each sample. The subdirectory “ReadQuality” contains reads quality information for each sample plotted as both boxplots and heatmaps. The subdirectory “RPKMSaturation” contains information about read depth saturation for each sample as assessed by RPKM resamplings. All transcripts were divided into four quantiles based on their expression and a relative difference of observed and real RPKM values are plotted for each sample. (ZIP) [file pgen.1006840.s015.zip › RNASeq/ReadQuality/12D_CGCTCATT-TAATCTTA_L00M_R1_001.readquality.qual.heatmap.pdf]

Phred Quality Score

30  
35  
40

Position of Read(5'→3')

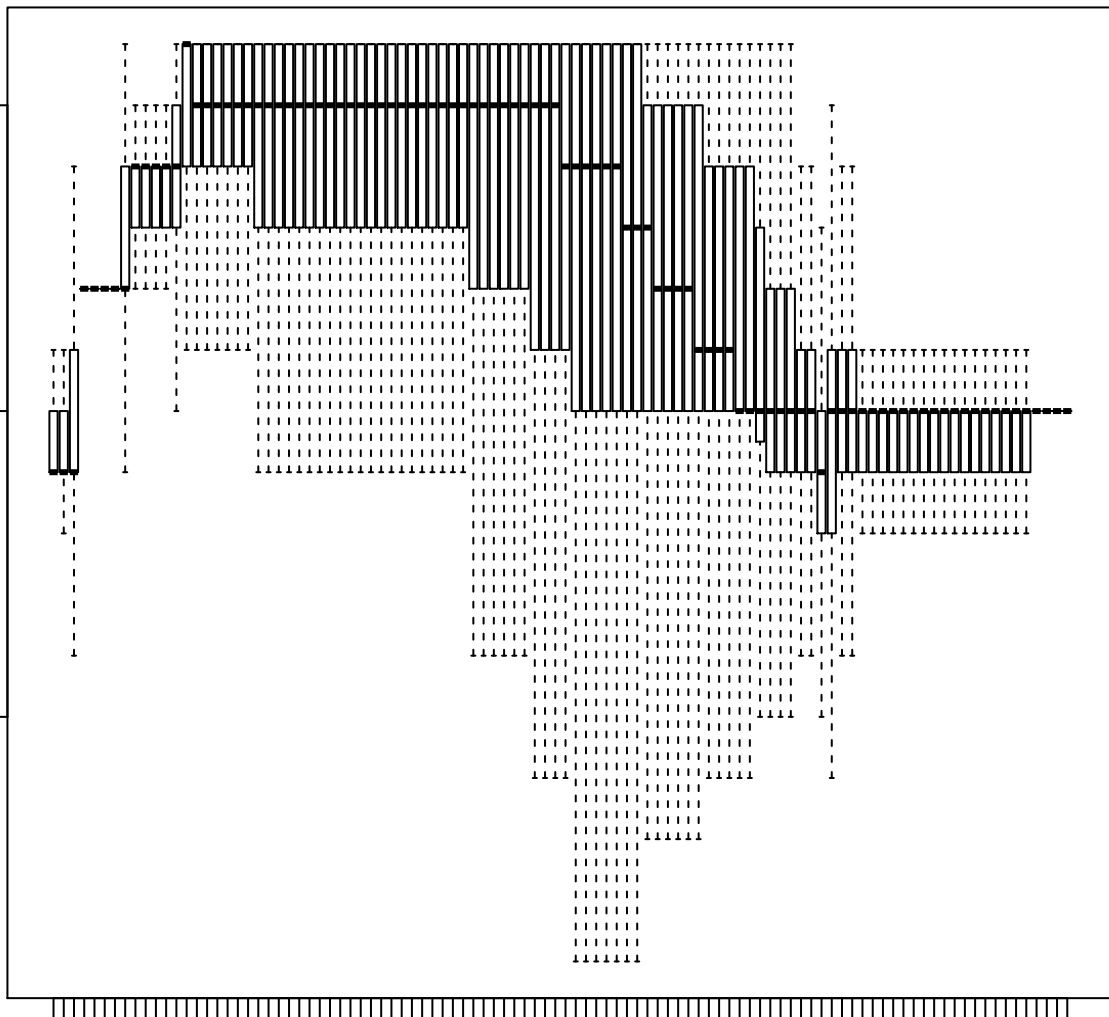

Supplement: S1 Data — This directory contains six subdirectories. The subdirectory “correlation” contains correlation heatmaps among all samples in diencephalon and telencephalon respectively. The subdirectory “mapping_stat” contains read mapping information on genomic features for each sample. The subdirectory “MDS” shows three dimensional MDS plots of the samples. The subdirectory “ReadDuplication” contains read duplication distributions for each sample. The subdirectory “ReadQuality” contains reads quality information for each sample plotted as both boxplots and heatmaps. The subdirectory “RPKMSaturation” contains information about read depth saturation for each sample as assessed by RPKM resamplings. All transcripts were divided into four quantiles based on their expression and a relative difference of observed and real RPKM values are plotted for each sample. (ZIP) [file pgen.1006840.s015.zip › RNASeq/ReadQuality/12T_CGCTCATT-AGGCGAAG_L00M_R1_001.readquality.qual.boxplot.pdf]

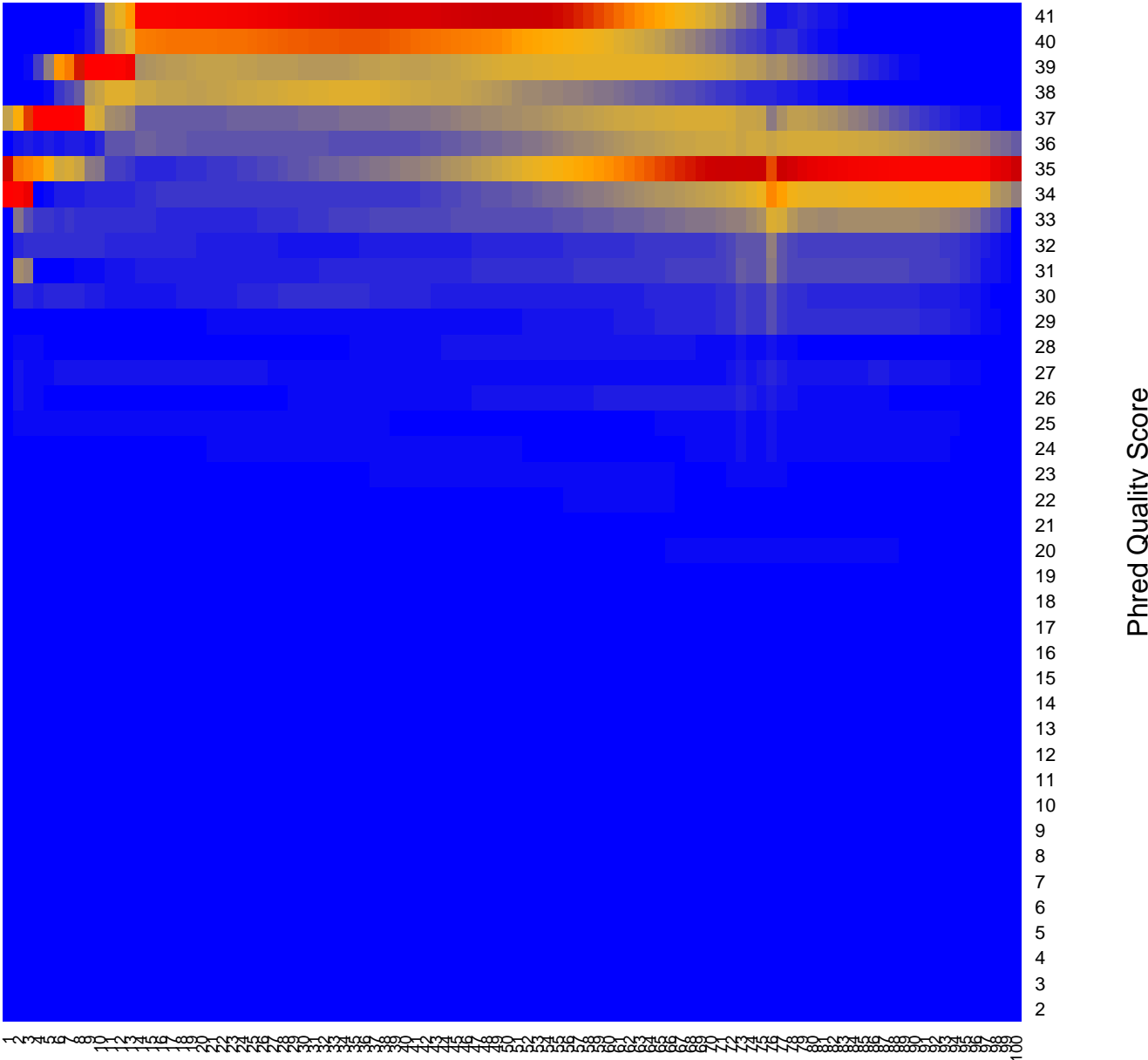

Supplement: S1 Data — This directory contains six subdirectories. The subdirectory “correlation” contains correlation heatmaps among all samples in diencephalon and telencephalon respectively. The subdirectory “mapping_stat” contains read mapping information on genomic features for each sample. The subdirectory “MDS” shows three dimensional MDS plots of the samples. The subdirectory “ReadDuplication” contains read duplication distributions for each sample. The subdirectory “ReadQuality” contains reads quality information for each sample plotted as both boxplots and heatmaps. The subdirectory “RPKMSaturation” contains information about read depth saturation for each sample as assessed by RPKM resamplings. All transcripts were divided into four quantiles based on their expression and a relative difference of observed and real RPKM values are plotted for each sample. (ZIP) [file pgen.1006840.s015.zip › RNASeq/ReadQuality/12T_CGCTCATT-AGGCGAAG_L00M_R1_001.readquality.qual.heatmap.pdf]

Phred Quality Score

30  
35  
40

Position of Read(5'→3')

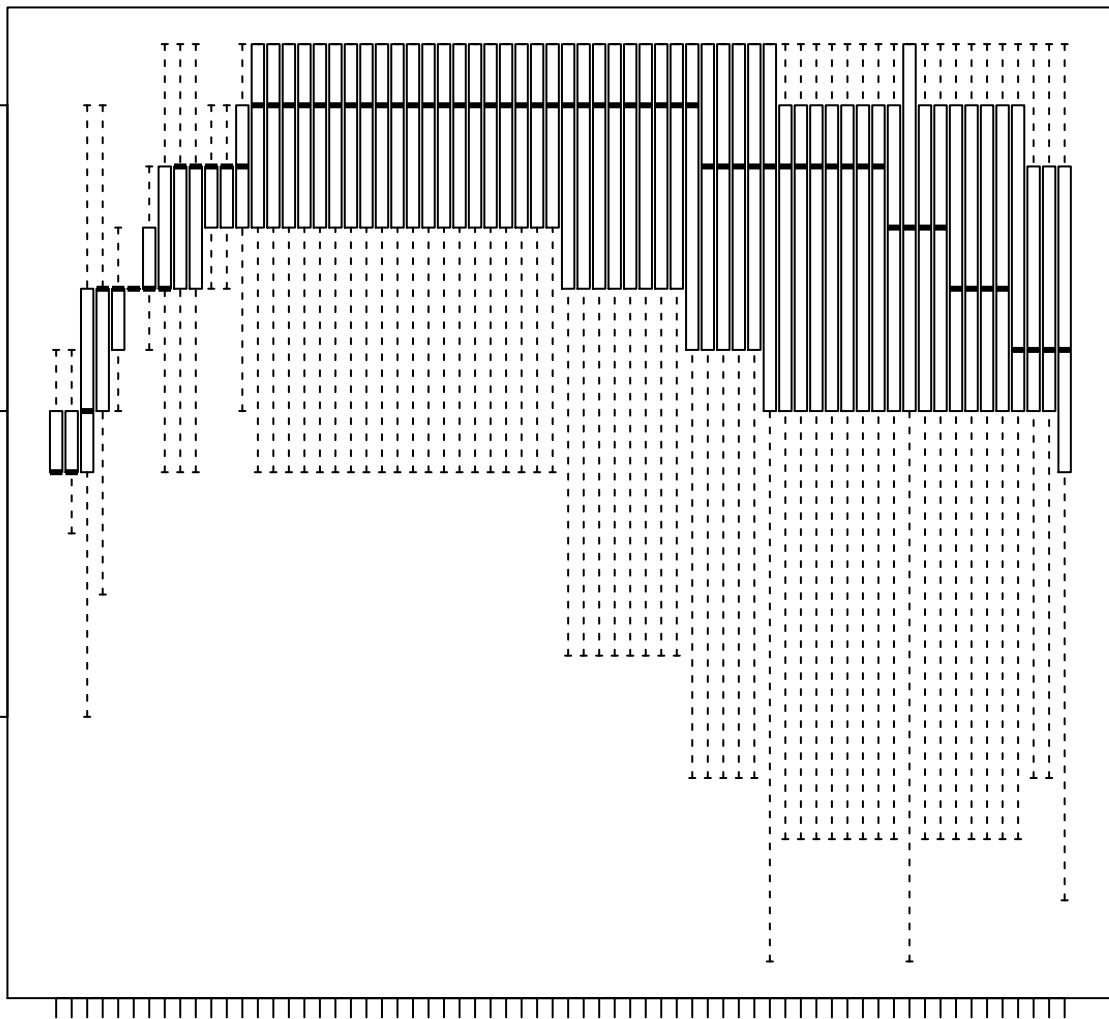

Supplement: S1 Data — This directory contains six subdirectories. The subdirectory “correlation” contains correlation heatmaps among all samples in diencephalon and telencephalon respectively. The subdirectory “mapping_stat” contains read mapping information on genomic features for each sample. The subdirectory “MDS” shows three dimensional MDS plots of the samples. The subdirectory “ReadDuplication” contains read duplication distributions for each sample. The subdirectory “ReadQuality” contains reads quality information for each sample plotted as both boxplots and heatmaps. The subdirectory “RPKMSaturation” contains information about read depth saturation for each sample as assessed by RPKM resamplings. All transcripts were divided into four quantiles based on their expression and a relative difference of observed and real RPKM values are plotted for each sample. (ZIP) [file pgen.1006840.s015.zip › RNASeq/ReadQuality/13D_CGCTCATT-GTACTGAC_L00M_R1_001.readquality.qual.boxplot.pdf]

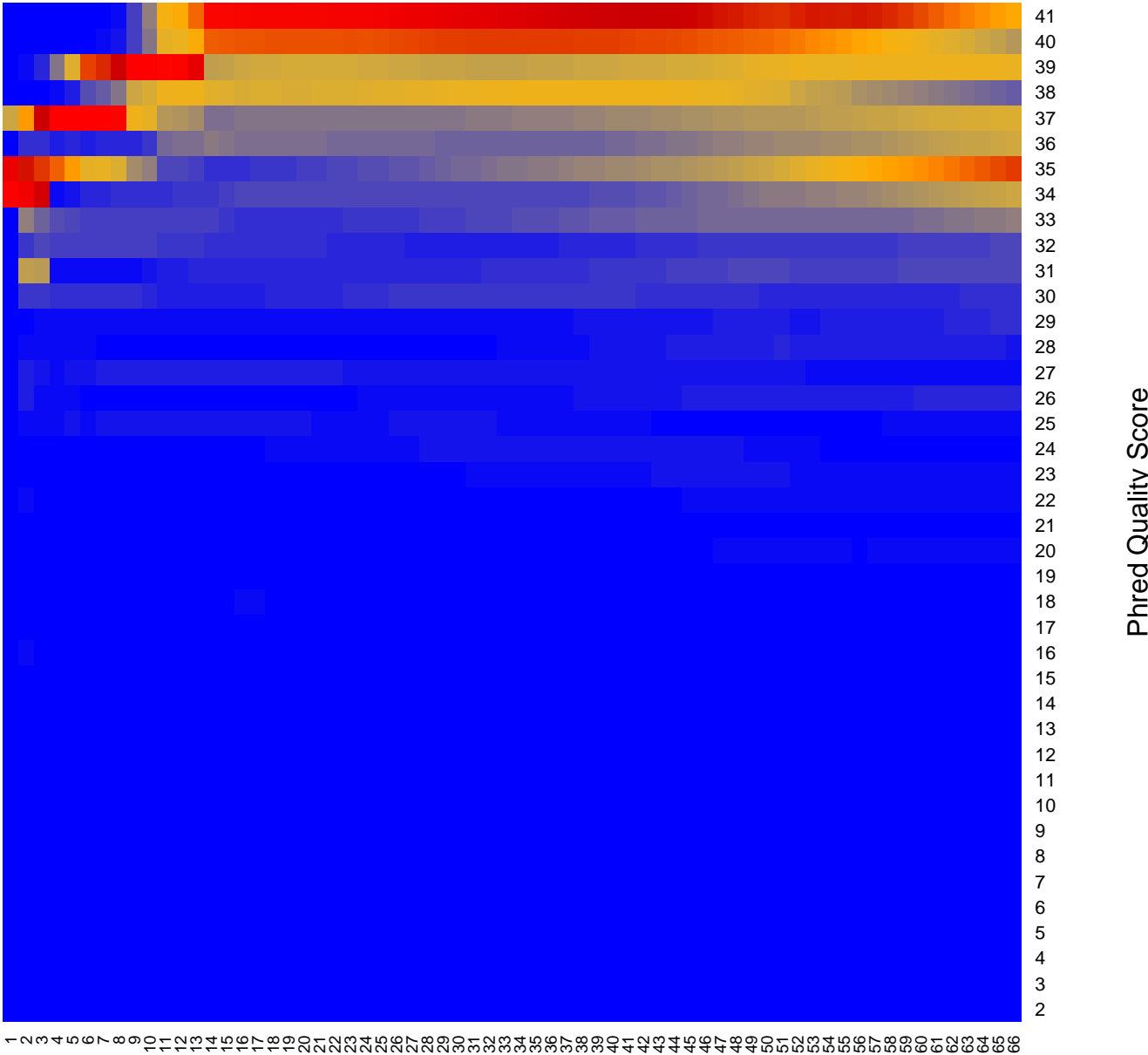

Position of Read

Supplement: S1 Data — This directory contains six subdirectories. The subdirectory “correlation” contains correlation heatmaps among all samples in diencephalon and telencephalon respectively. The subdirectory “mapping_stat” contains read mapping information on genomic features for each sample. The subdirectory “MDS” shows three dimensional MDS plots of the samples. The subdirectory “ReadDuplication” contains read duplication distributions for each sample. The subdirectory “ReadQuality” contains reads quality information for each sample plotted as both boxplots and heatmaps. The subdirectory “RPKMSaturation” contains information about read depth saturation for each sample as assessed by RPKM resamplings. All transcripts were divided into four quantiles based on their expression and a relative difference of observed and real RPKM values are plotted for each sample. (ZIP) [file pgen.1006840.s015.zip › RNASeq/ReadQuality/13D_CGCTCATT-GTACTGAC_L00M_R1_001.readquality.qual.heatmap.pdf]

Phred Quality Score

40

35

30

Position of Read(5'→3')

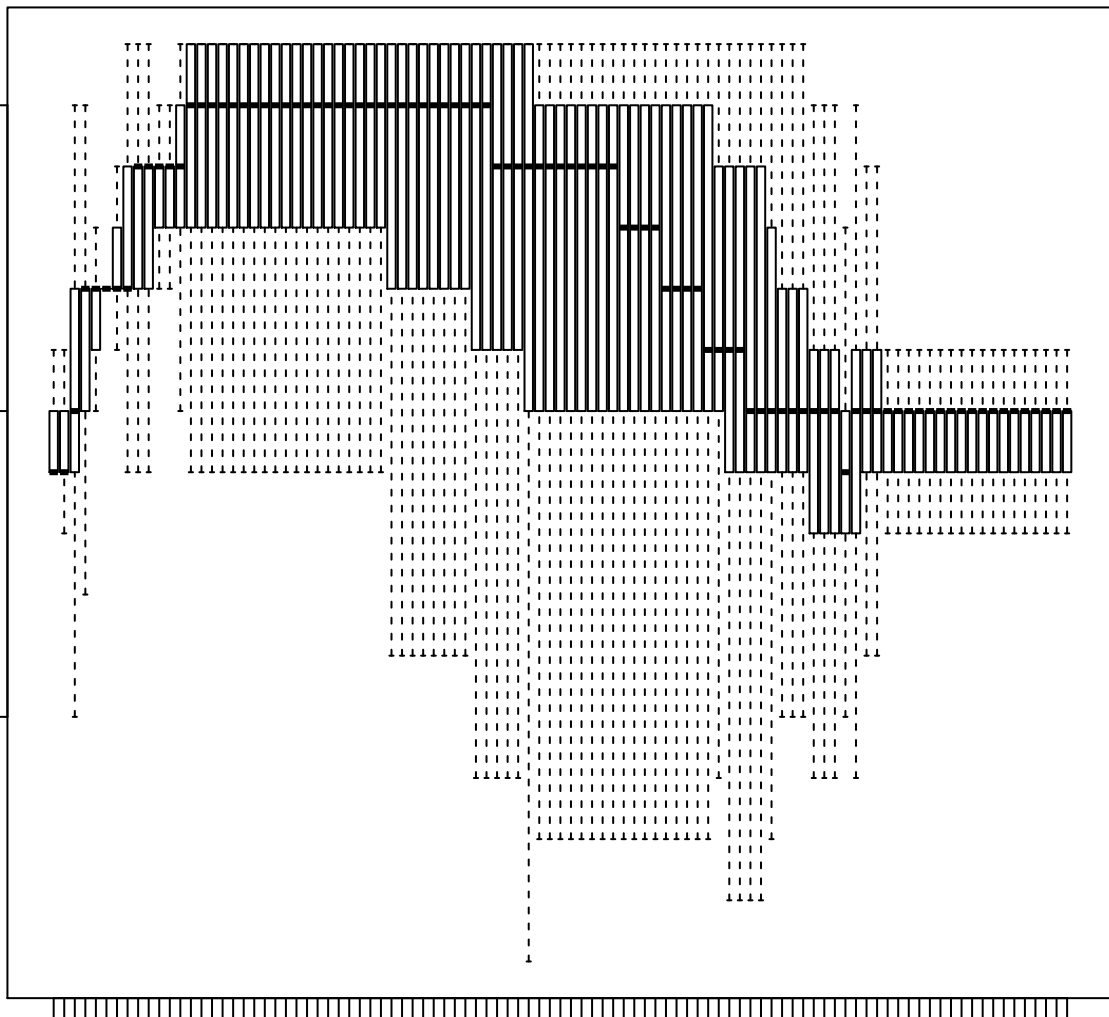

Supplement: S1 Data — This directory contains six subdirectories. The subdirectory “correlation” contains correlation heatmaps among all samples in diencephalon and telencephalon respectively. The subdirectory “mapping_stat” contains read mapping information on genomic features for each sample. The subdirectory “MDS” shows three dimensional MDS plots of the samples. The subdirectory “ReadDuplication” contains read duplication distributions for each sample. The subdirectory “ReadQuality” contains reads quality information for each sample plotted as both boxplots and heatmaps. The subdirectory “RPKMSaturation” contains information about read depth saturation for each sample as assessed by RPKM resamplings. All transcripts were divided into four quantiles based on their expression and a relative difference of observed and real RPKM values are plotted for each sample. (ZIP) [file pgen.1006840.s015.zip › RNASeq/ReadQuality/13T_CGCTCATT-CAGGACGT_L00M_R1_001.readquality.qual.boxplot.pdf]

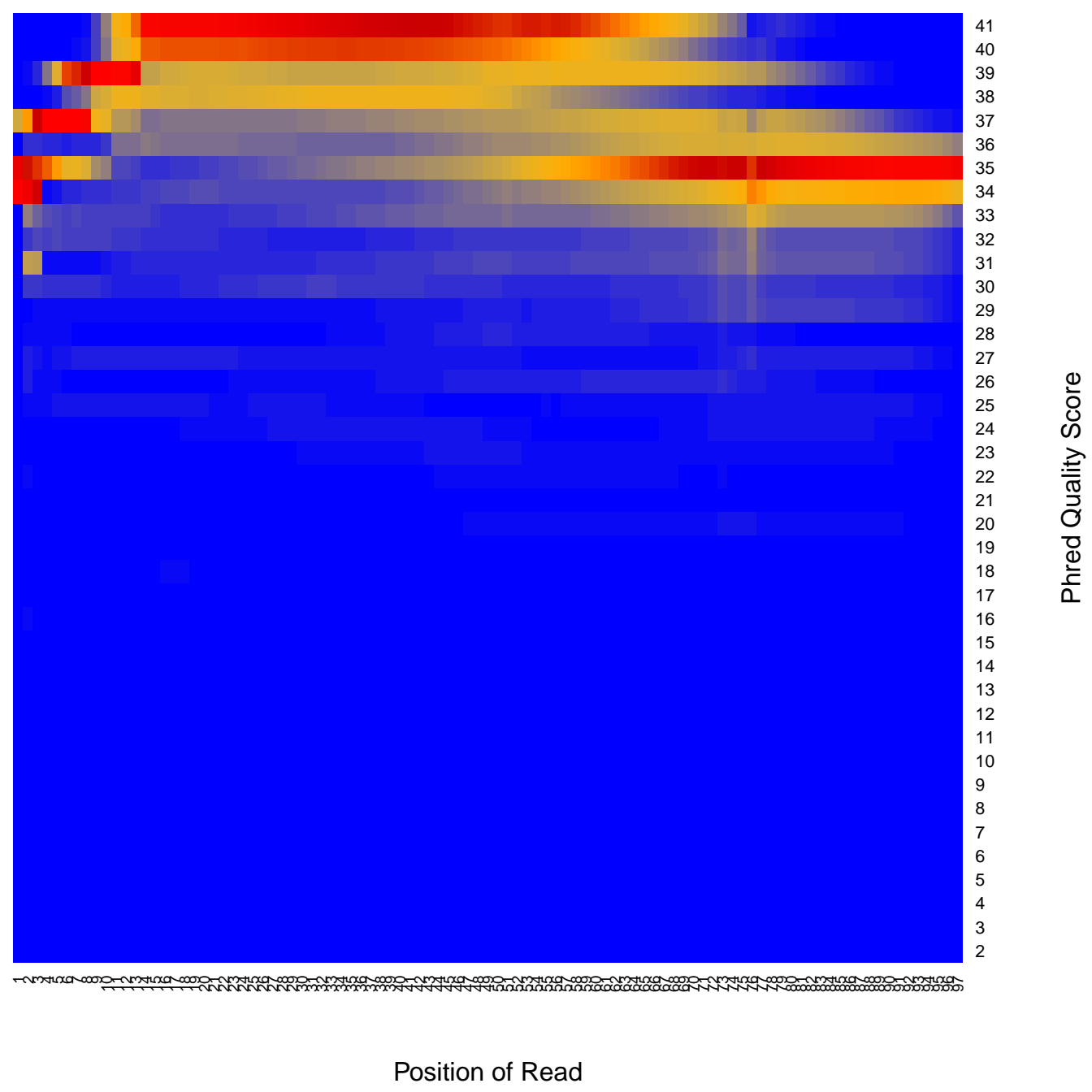

Supplement: S1 Data — This directory contains six subdirectories. The subdirectory “correlation” contains correlation heatmaps among all samples in diencephalon and telencephalon respectively. The subdirectory “mapping_stat” contains read mapping information on genomic features for each sample. The subdirectory “MDS” shows three dimensional MDS plots of the samples. The subdirectory “ReadDuplication” contains read duplication distributions for each sample. The subdirectory “ReadQuality” contains reads quality information for each sample plotted as both boxplots and heatmaps. The subdirectory “RPKMSaturation” contains information about read depth saturation for each sample as assessed by RPKM resamplings. All transcripts were divided into four quantiles based on their expression and a relative difference of observed and real RPKM values are plotted for each sample. (ZIP) [file pgen.1006840.s015.zip › RNASeq/ReadQuality/13T_CGCTCATT-CAGGACGT_L00M_R1_001.readquality.qual.heatmap.pdf]

Phred Quality Score

30  
35  
40

Position of Read(5'→3')

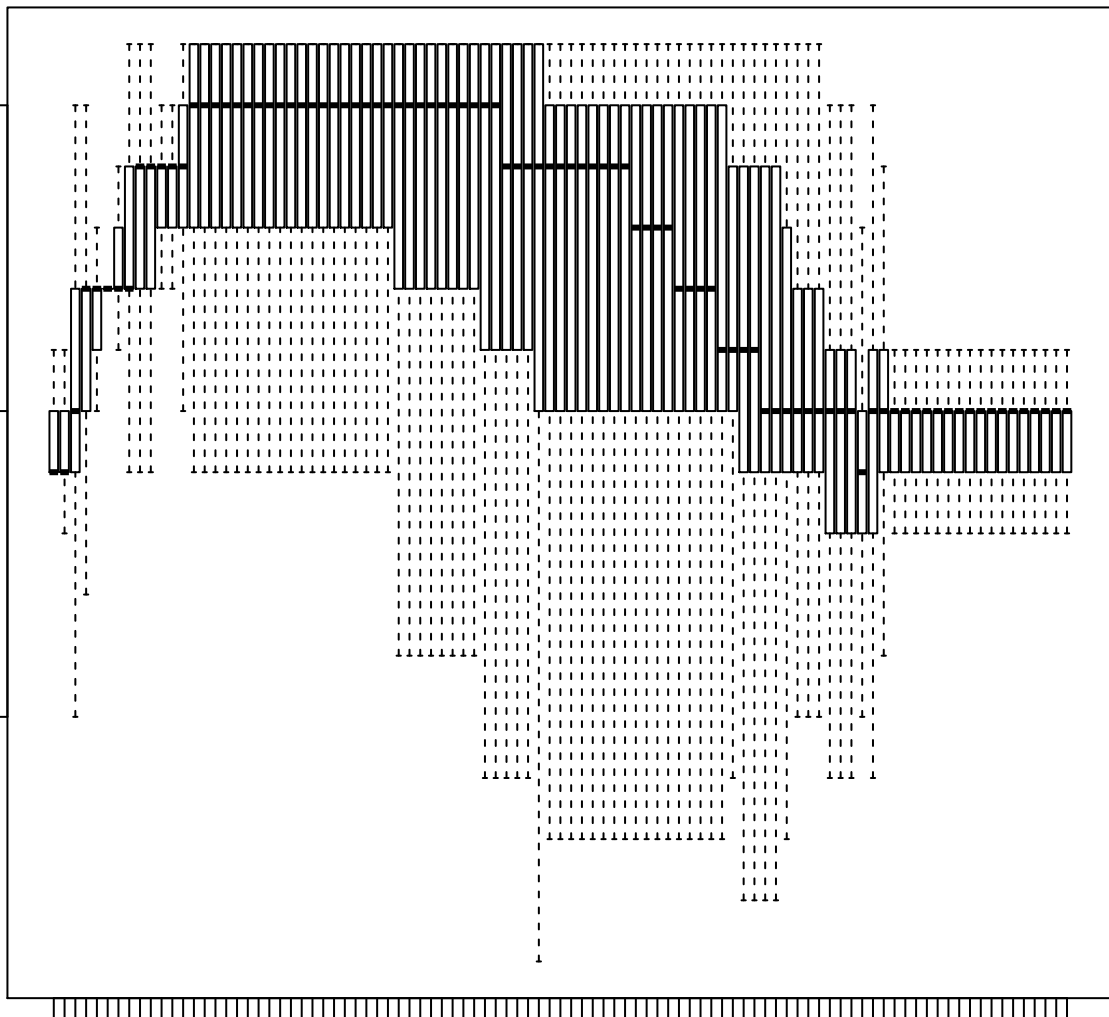

Supplement: S1 Data — This directory contains six subdirectories. The subdirectory “correlation” contains correlation heatmaps among all samples in diencephalon and telencephalon respectively. The subdirectory “mapping_stat” contains read mapping information on genomic features for each sample. The subdirectory “MDS” shows three dimensional MDS plots of the samples. The subdirectory “ReadDuplication” contains read duplication distributions for each sample. The subdirectory “ReadQuality” contains reads quality information for each sample plotted as both boxplots and heatmaps. The subdirectory “RPKMSaturation” contains information about read depth saturation for each sample as assessed by RPKM resamplings. All transcripts were divided into four quantiles based on their expression and a relative difference of observed and real RPKM values are plotted for each sample. (ZIP) [file pgen.1006840.s015.zip › RNASeq/ReadQuality/15D_GAGATTCC-ATAGAGGC_L00M_R1_001.readquality.qual.boxplot.pdf]

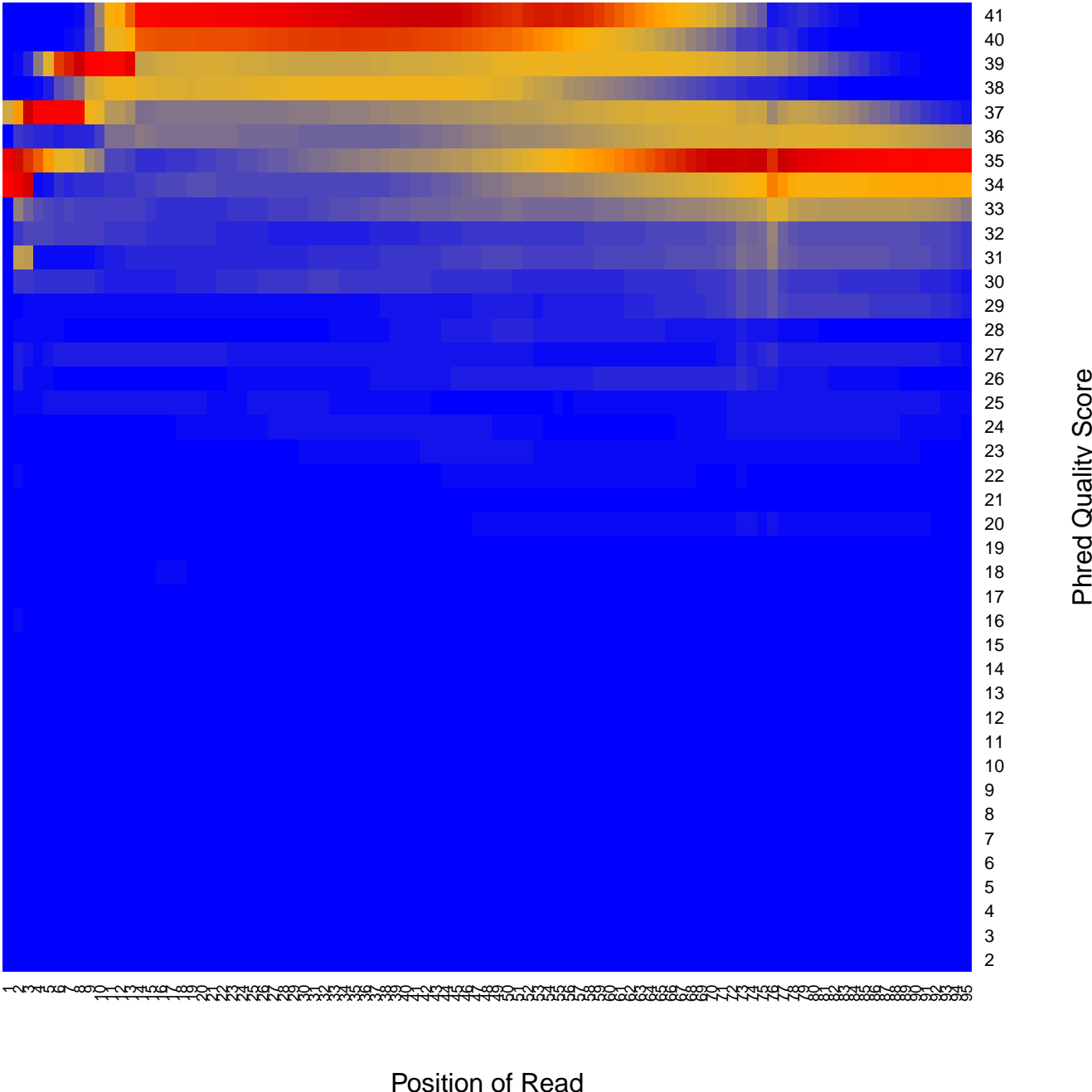

Supplement: S1 Data — This directory contains six subdirectories. The subdirectory “correlation” contains correlation heatmaps among all samples in diencephalon and telencephalon respectively. The subdirectory “mapping_stat” contains read mapping information on genomic features for each sample. The subdirectory “MDS” shows three dimensional MDS plots of the samples. The subdirectory “ReadDuplication” contains read duplication distributions for each sample. The subdirectory “ReadQuality” contains reads quality information for each sample plotted as both boxplots and heatmaps. The subdirectory “RPKMSaturation” contains information about read depth saturation for each sample as assessed by RPKM resamplings. All transcripts were divided into four quantiles based on their expression and a relative difference of observed and real RPKM values are plotted for each sample. (ZIP) [file pgen.1006840.s015.zip › RNASeq/ReadQuality/15D_GAGATTCC-ATAGAGGC_L00M_R1_001.readquality.qual.heatmap.pdf]

Phred Quality Score

30  
35  
40

Position of Read(5'→3')

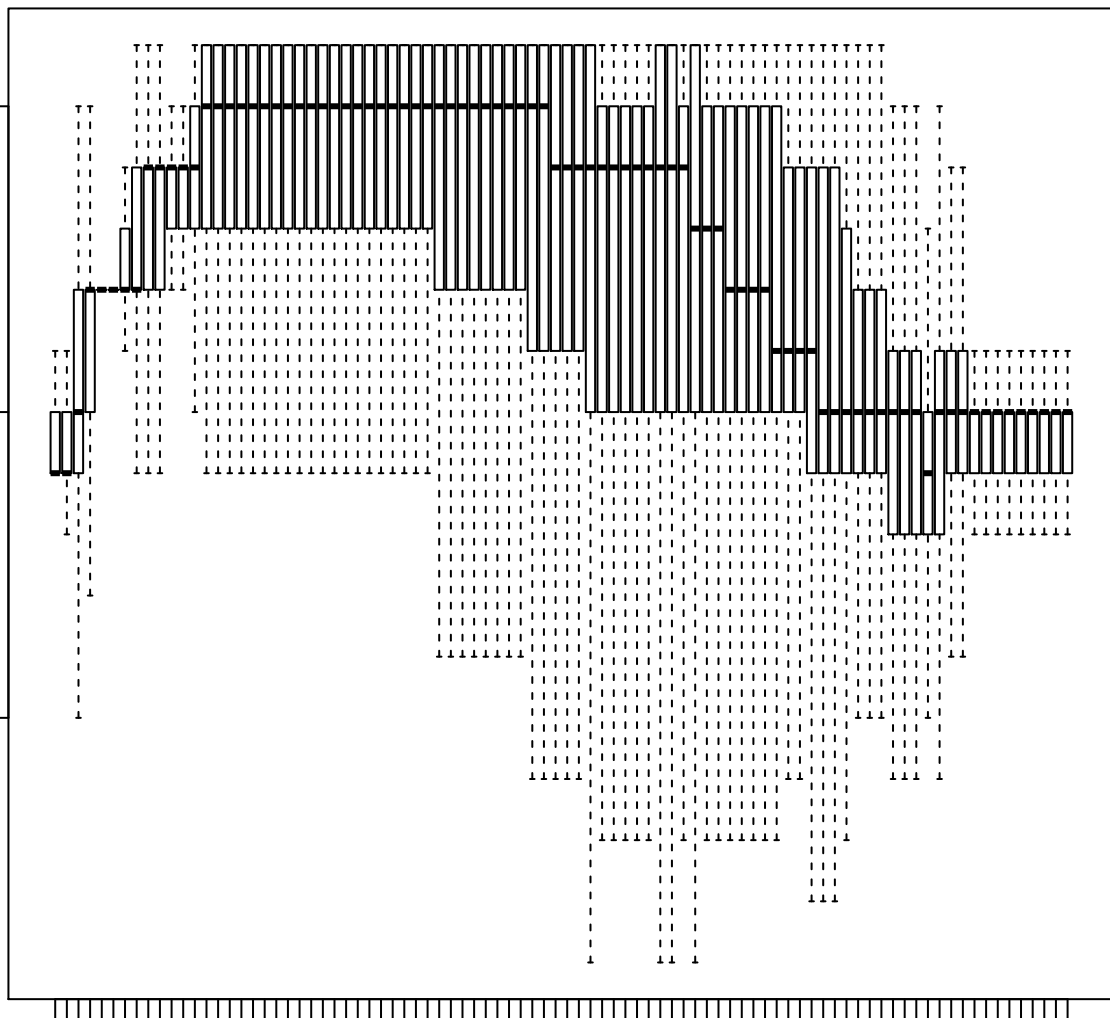

Supplement: S1 Data — This directory contains six subdirectories. The subdirectory “correlation” contains correlation heatmaps among all samples in diencephalon and telencephalon respectively. The subdirectory “mapping_stat” contains read mapping information on genomic features for each sample. The subdirectory “MDS” shows three dimensional MDS plots of the samples. The subdirectory “ReadDuplication” contains read duplication distributions for each sample. The subdirectory “ReadQuality” contains reads quality information for each sample plotted as both boxplots and heatmaps. The subdirectory “RPKMSaturation” contains information about read depth saturation for each sample as assessed by RPKM resamplings. All transcripts were divided into four quantiles based on their expression and a relative difference of observed and real RPKM values are plotted for each sample. (ZIP) [file pgen.1006840.s015.zip › RNASeq/ReadQuality/15T_GAGATTCC-TATAGCCT_L00M_R1_001.readquality.qual.boxplot.pdf]

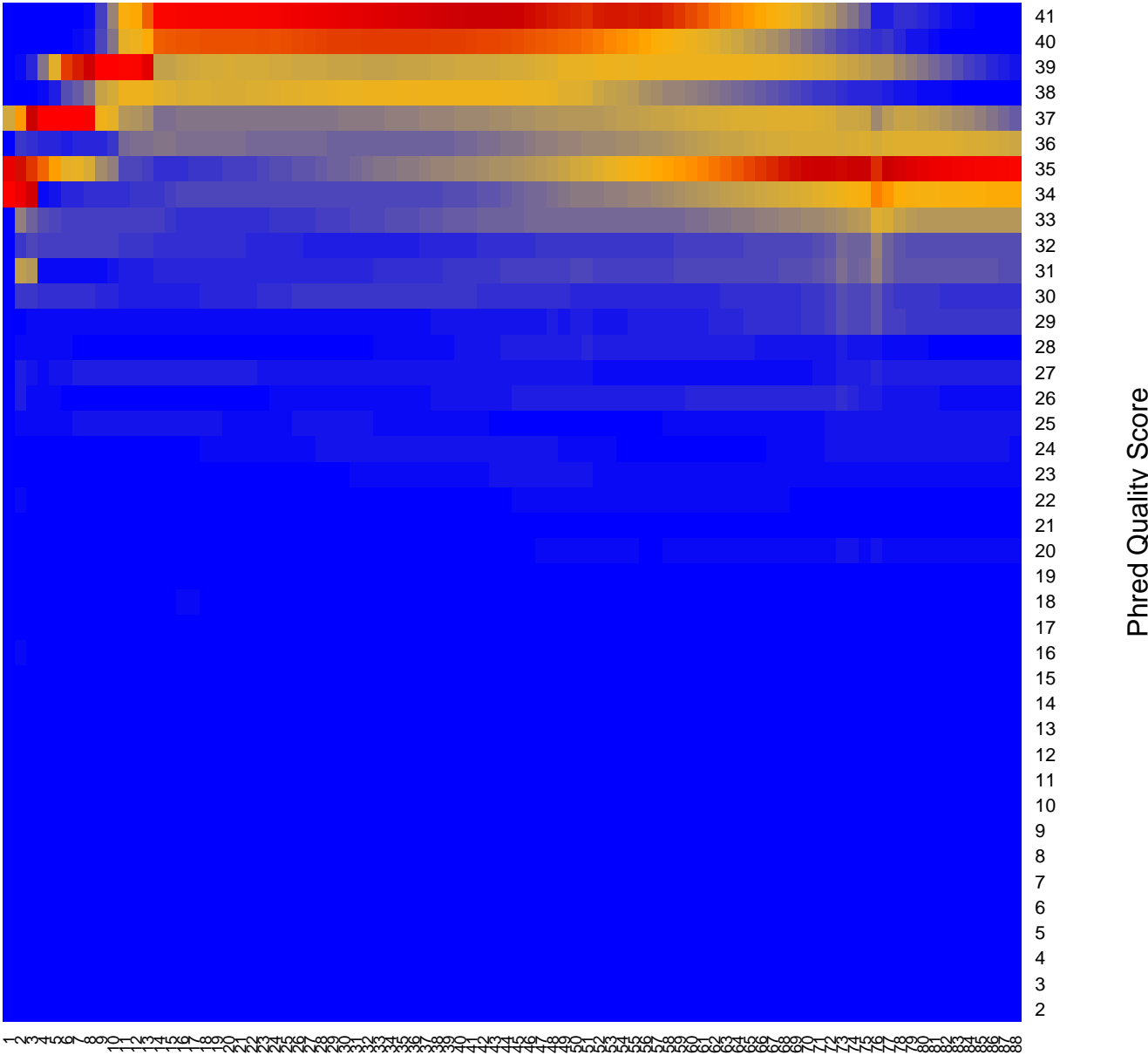

Position of Read

Supplement: S1 Data — This directory contains six subdirectories. The subdirectory “correlation” contains correlation heatmaps among all samples in diencephalon and telencephalon respectively. The subdirectory “mapping_stat” contains read mapping information on genomic features for each sample. The subdirectory “MDS” shows three dimensional MDS plots of the samples. The subdirectory “ReadDuplication” contains read duplication distributions for each sample. The subdirectory “ReadQuality” contains reads quality information for each sample plotted as both boxplots and heatmaps. The subdirectory “RPKMSaturation” contains information about read depth saturation for each sample as assessed by RPKM resamplings. All transcripts were divided into four quantiles based on their expression and a relative difference of observed and real RPKM values are plotted for each sample. (ZIP) [file pgen.1006840.s015.zip › RNASeq/ReadQuality/15T_GAGATTCC-TATAGCCT_L00M_R1_001.readquality.qual.heatmap.pdf]

Phred Quality Score

30  
35  
40

Position of Read(5'→3')

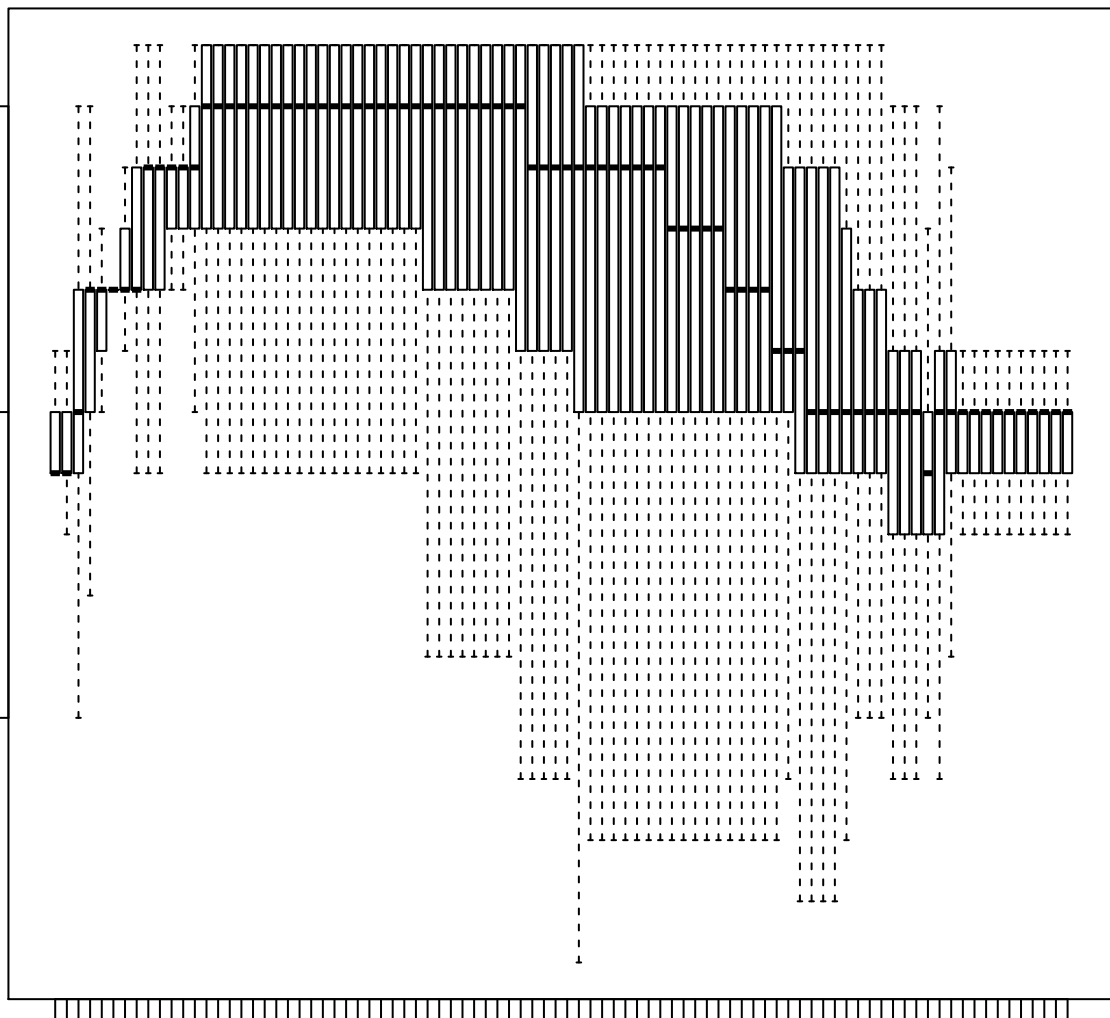

Supplement: S1 Data — This directory contains six subdirectories. The subdirectory “correlation” contains correlation heatmaps among all samples in diencephalon and telencephalon respectively. The subdirectory “mapping_stat” contains read mapping information on genomic features for each sample. The subdirectory “MDS” shows three dimensional MDS plots of the samples. The subdirectory “ReadDuplication” contains read duplication distributions for each sample. The subdirectory “ReadQuality” contains reads quality information for each sample plotted as both boxplots and heatmaps. The subdirectory “RPKMSaturation” contains information about read depth saturation for each sample as assessed by RPKM resamplings. All transcripts were divided into four quantiles based on their expression and a relative difference of observed and real RPKM values are plotted for each sample. (ZIP) [file pgen.1006840.s015.zip › RNASeq/ReadQuality/17D_GAGATTCC-GGCTCTGA_L00M_R1_001.readquality.qual.boxplot.pdf]

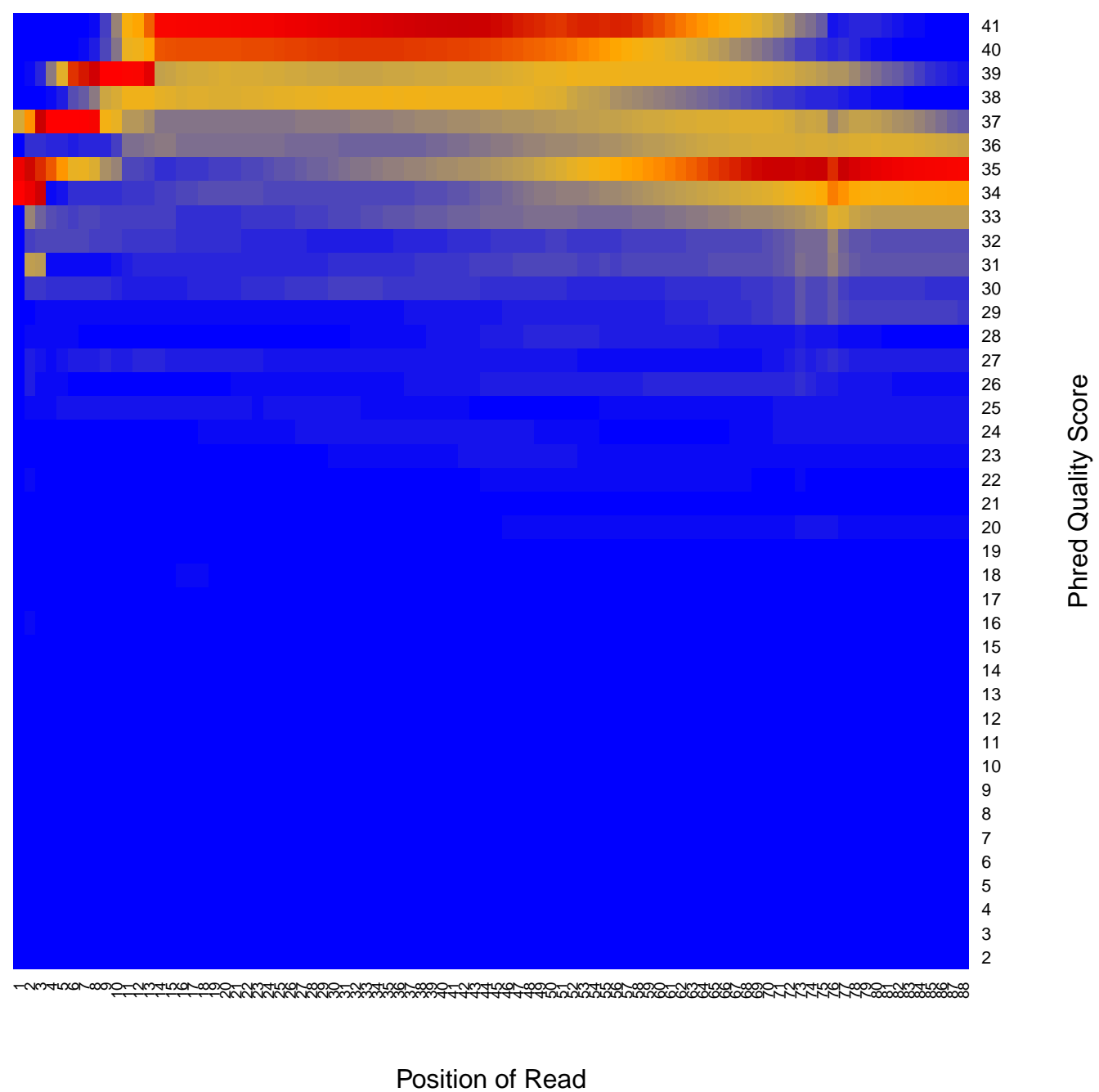

Supplement: S1 Data — This directory contains six subdirectories. The subdirectory “correlation” contains correlation heatmaps among all samples in diencephalon and telencephalon respectively. The subdirectory “mapping_stat” contains read mapping information on genomic features for each sample. The subdirectory “MDS” shows three dimensional MDS plots of the samples. The subdirectory “ReadDuplication” contains read duplication distributions for each sample. The subdirectory “ReadQuality” contains reads quality information for each sample plotted as both boxplots and heatmaps. The subdirectory “RPKMSaturation” contains information about read depth saturation for each sample as assessed by RPKM resamplings. All transcripts were divided into four quantiles based on their expression and a relative difference of observed and real RPKM values are plotted for each sample. (ZIP) [file pgen.1006840.s015.zip › RNASeq/ReadQuality/17D_GAGATTCC-GGCTCTGA_L00M_R1_001.readquality.qual.heatmap.pdf]

Phred Quality Score

40  
35  
30

Position of Read(5'→3')

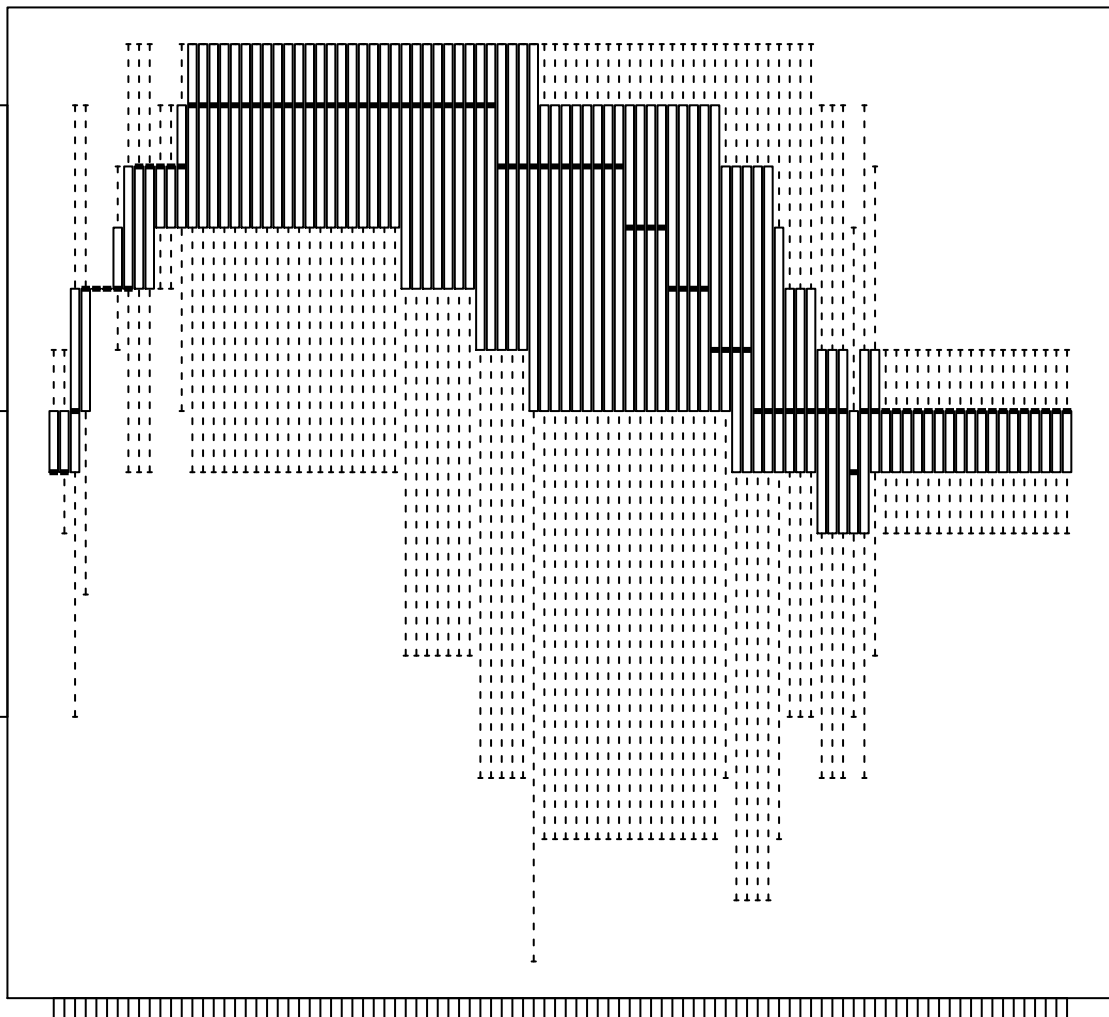

Supplement: S1 Data — This directory contains six subdirectories. The subdirectory “correlation” contains correlation heatmaps among all samples in diencephalon and telencephalon respectively. The subdirectory “mapping_stat” contains read mapping information on genomic features for each sample. The subdirectory “MDS” shows three dimensional MDS plots of the samples. The subdirectory “ReadDuplication” contains read duplication distributions for each sample. The subdirectory “ReadQuality” contains reads quality information for each sample plotted as both boxplots and heatmaps. The subdirectory “RPKMSaturation” contains information about read depth saturation for each sample as assessed by RPKM resamplings. All transcripts were divided into four quantiles based on their expression and a relative difference of observed and real RPKM values are plotted for each sample. (ZIP) [file pgen.1006840.s015.zip › RNASeq/ReadQuality/17T_GAGATTCC-CCTATCCT_L00M_R1_001.readquality.qual.boxplot.pdf]

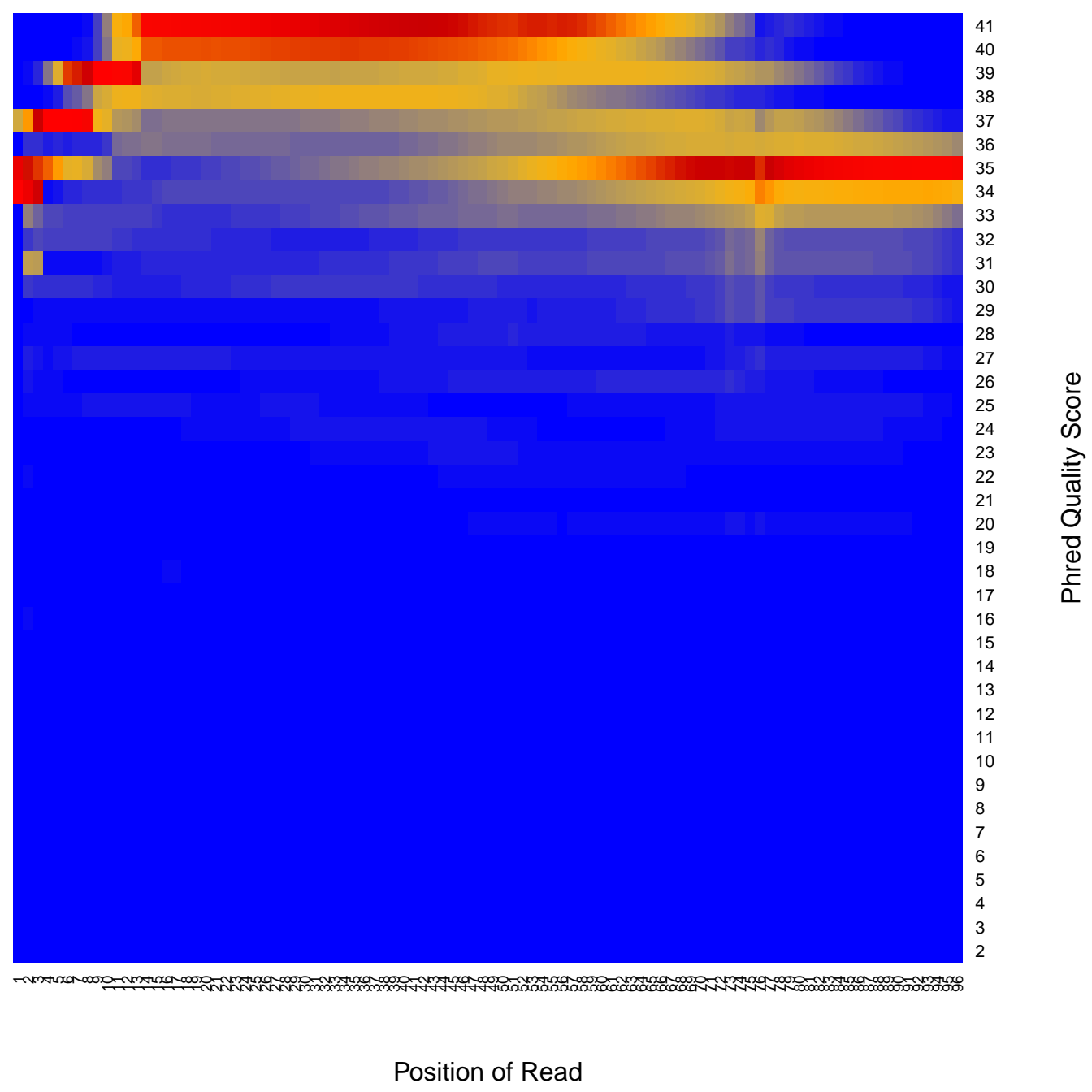

Supplement: S1 Data — This directory contains six subdirectories. The subdirectory “correlation” contains correlation heatmaps among all samples in diencephalon and telencephalon respectively. The subdirectory “mapping_stat” contains read mapping information on genomic features for each sample. The subdirectory “MDS” shows three dimensional MDS plots of the samples. The subdirectory “ReadDuplication” contains read duplication distributions for each sample. The subdirectory “ReadQuality” contains reads quality information for each sample plotted as both boxplots and heatmaps. The subdirectory “RPKMSaturation” contains information about read depth saturation for each sample as assessed by RPKM resamplings. All transcripts were divided into four quantiles based on their expression and a relative difference of observed and real RPKM values are plotted for each sample. (ZIP) [file pgen.1006840.s015.zip › RNASeq/ReadQuality/17T_GAGATTCC-CCTATCCT_L00M_R1_001.readquality.qual.heatmap.pdf]

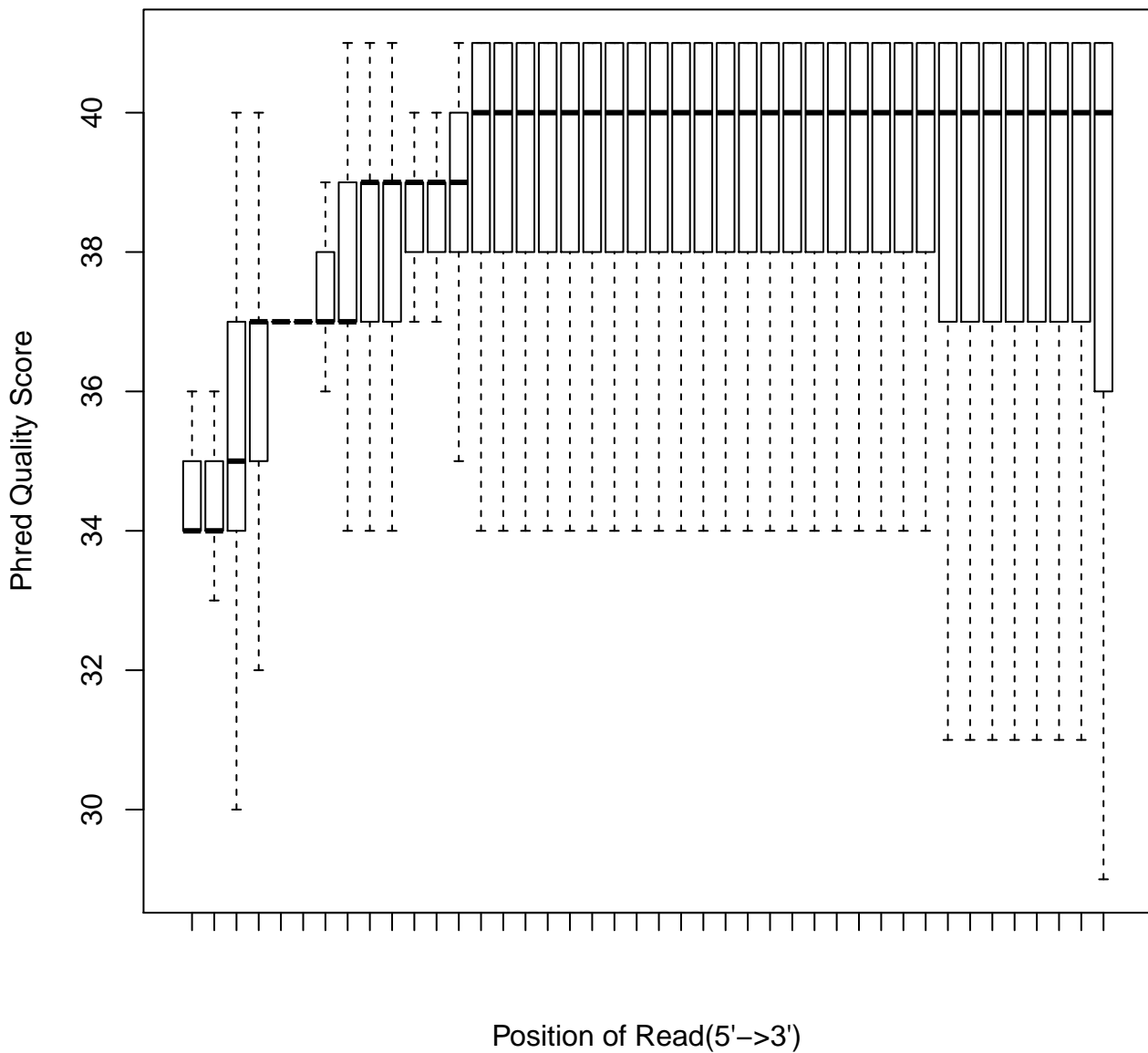

Supplement: S1 Data — This directory contains six subdirectories. The subdirectory “correlation” contains correlation heatmaps among all samples in diencephalon and telencephalon respectively. The subdirectory “mapping_stat” contains read mapping information on genomic features for each sample. The subdirectory “MDS” shows three dimensional MDS plots of the samples. The subdirectory “ReadDuplication” contains read duplication distributions for each sample. The subdirectory “ReadQuality” contains reads quality information for each sample plotted as both boxplots and heatmaps. The subdirectory “RPKMSaturation” contains information about read depth saturation for each sample as assessed by RPKM resamplings. All transcripts were divided into four quantiles based on their expression and a relative difference of observed and real RPKM values are plotted for each sample. (ZIP) [file pgen.1006840.s015.zip › RNASeq/ReadQuality/18D_GAGATTCC-TAATCTTA_L00M_R1_001.readquality.qual.boxplot.pdf]

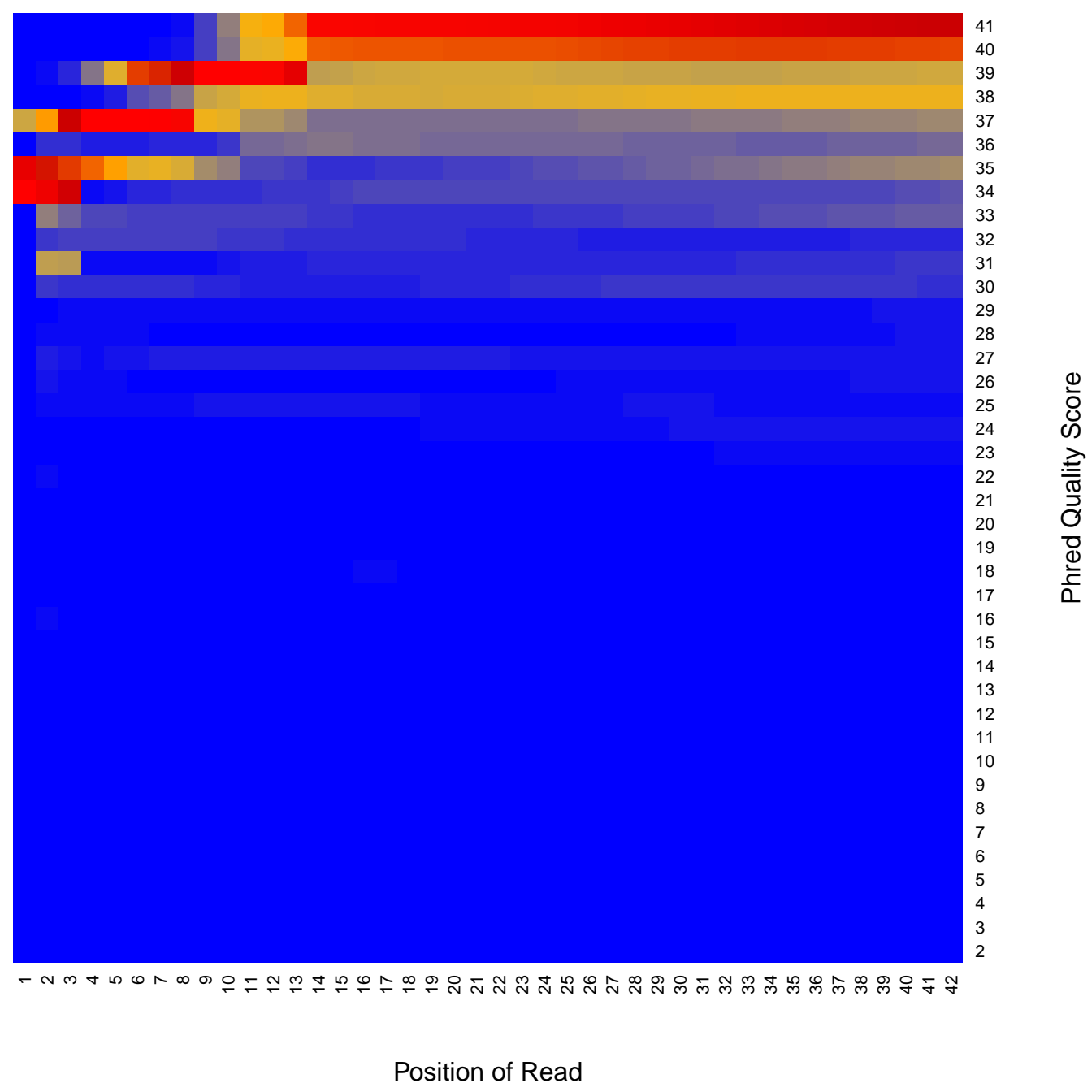

Supplement: S1 Data — This directory contains six subdirectories. The subdirectory “correlation” contains correlation heatmaps among all samples in diencephalon and telencephalon respectively. The subdirectory “mapping_stat” contains read mapping information on genomic features for each sample. The subdirectory “MDS” shows three dimensional MDS plots of the samples. The subdirectory “ReadDuplication” contains read duplication distributions for each sample. The subdirectory “ReadQuality” contains reads quality information for each sample plotted as both boxplots and heatmaps. The subdirectory “RPKMSaturation” contains information about read depth saturation for each sample as assessed by RPKM resamplings. All transcripts were divided into four quantiles based on their expression and a relative difference of observed and real RPKM values are plotted for each sample. (ZIP) [file pgen.1006840.s015.zip › RNASeq/ReadQuality/18D_GAGATTCC-TAATCTTA_L00M_R1_001.readquality.qual.heatmap.pdf]

Phred Quality Score

30 35 40

Position of Read(5'→3')

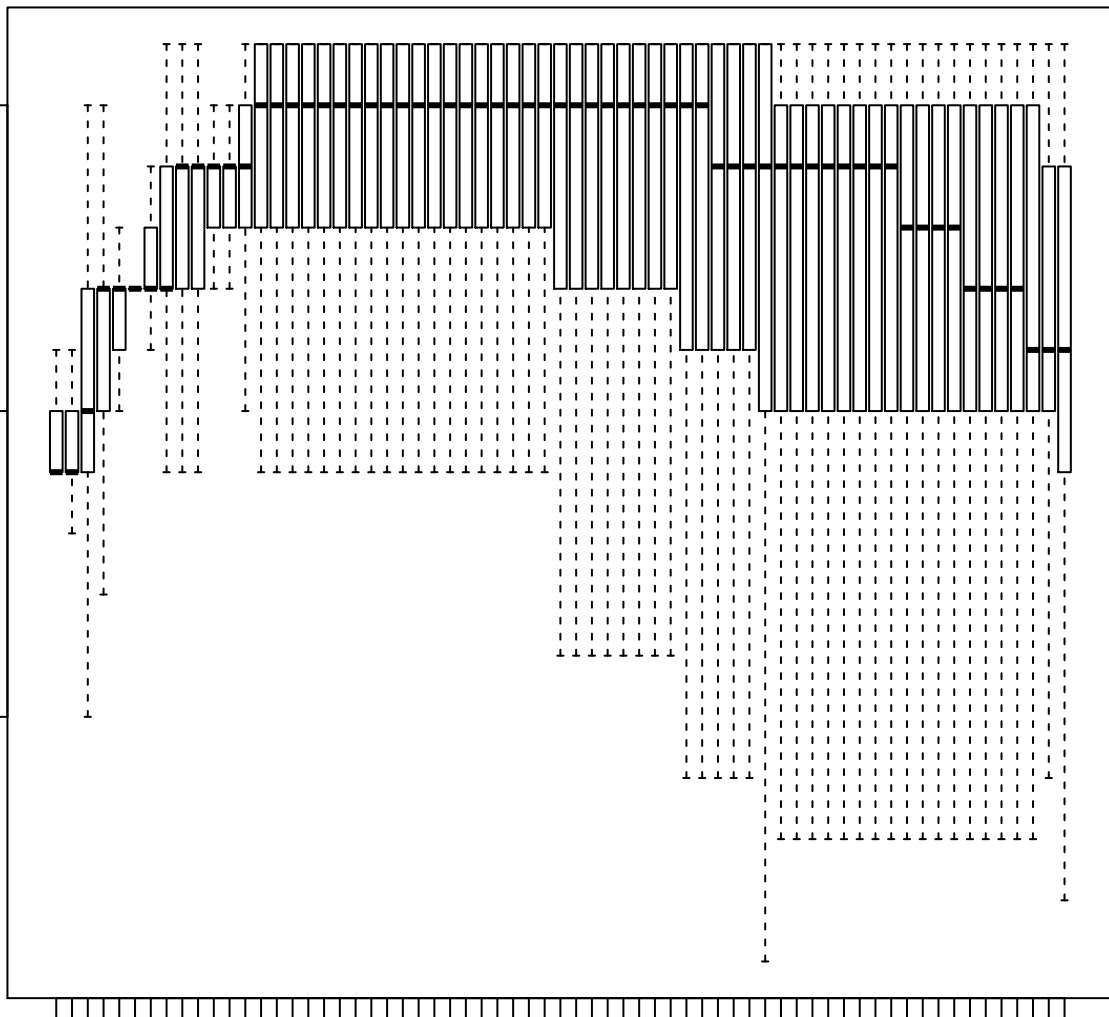

Supplement: S1 Data — This directory contains six subdirectories. The subdirectory “correlation” contains correlation heatmaps among all samples in diencephalon and telencephalon respectively. The subdirectory “mapping_stat” contains read mapping information on genomic features for each sample. The subdirectory “MDS” shows three dimensional MDS plots of the samples. The subdirectory “ReadDuplication” contains read duplication distributions for each sample. The subdirectory “ReadQuality” contains reads quality information for each sample plotted as both boxplots and heatmaps. The subdirectory “RPKMSaturation” contains information about read depth saturation for each sample as assessed by RPKM resamplings. All transcripts were divided into four quantiles based on their expression and a relative difference of observed and real RPKM values are plotted for each sample. (ZIP) [file pgen.1006840.s015.zip › RNASeq/ReadQuality/18T_GAGATTCC-AGGCGAAG_L00M_R1_001.readquality.qual.boxplot.pdf]

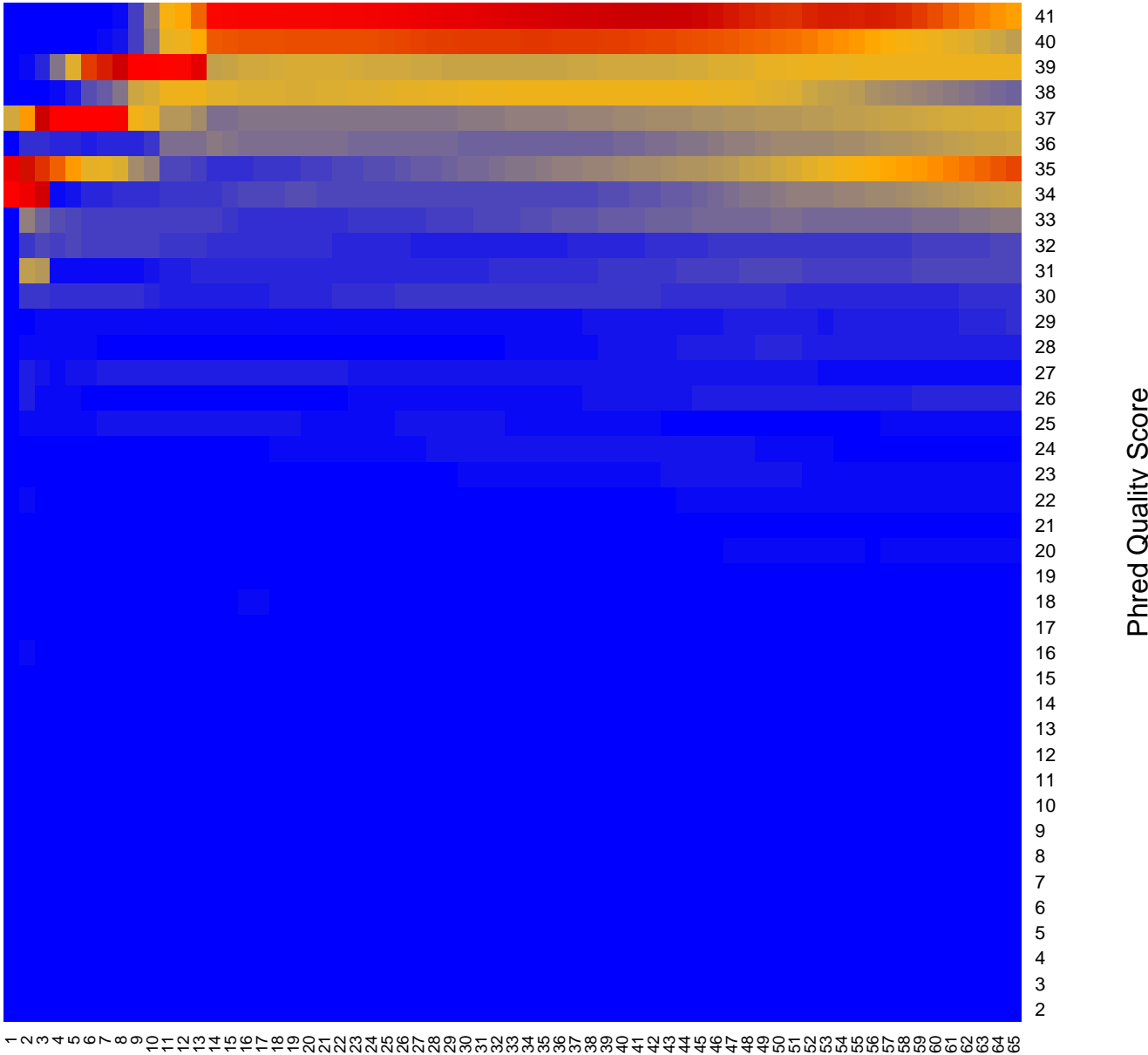

Position of Read

Supplement: S1 Data — This directory contains six subdirectories. The subdirectory “correlation” contains correlation heatmaps among all samples in diencephalon and telencephalon respectively. The subdirectory “mapping_stat” contains read mapping information on genomic features for each sample. The subdirectory “MDS” shows three dimensional MDS plots of the samples. The subdirectory “ReadDuplication” contains read duplication distributions for each sample. The subdirectory “ReadQuality” contains reads quality information for each sample plotted as both boxplots and heatmaps. The subdirectory “RPKMSaturation” contains information about read depth saturation for each sample as assessed by RPKM resamplings. All transcripts were divided into four quantiles based on their expression and a relative difference of observed and real RPKM values are plotted for each sample. (ZIP) [file pgen.1006840.s015.zip › RNASeq/ReadQuality/18T_GAGATTCC-AGGCGAAG_L00M_R1_001.readquality.qual.heatmap.pdf]

Phred Quality Score

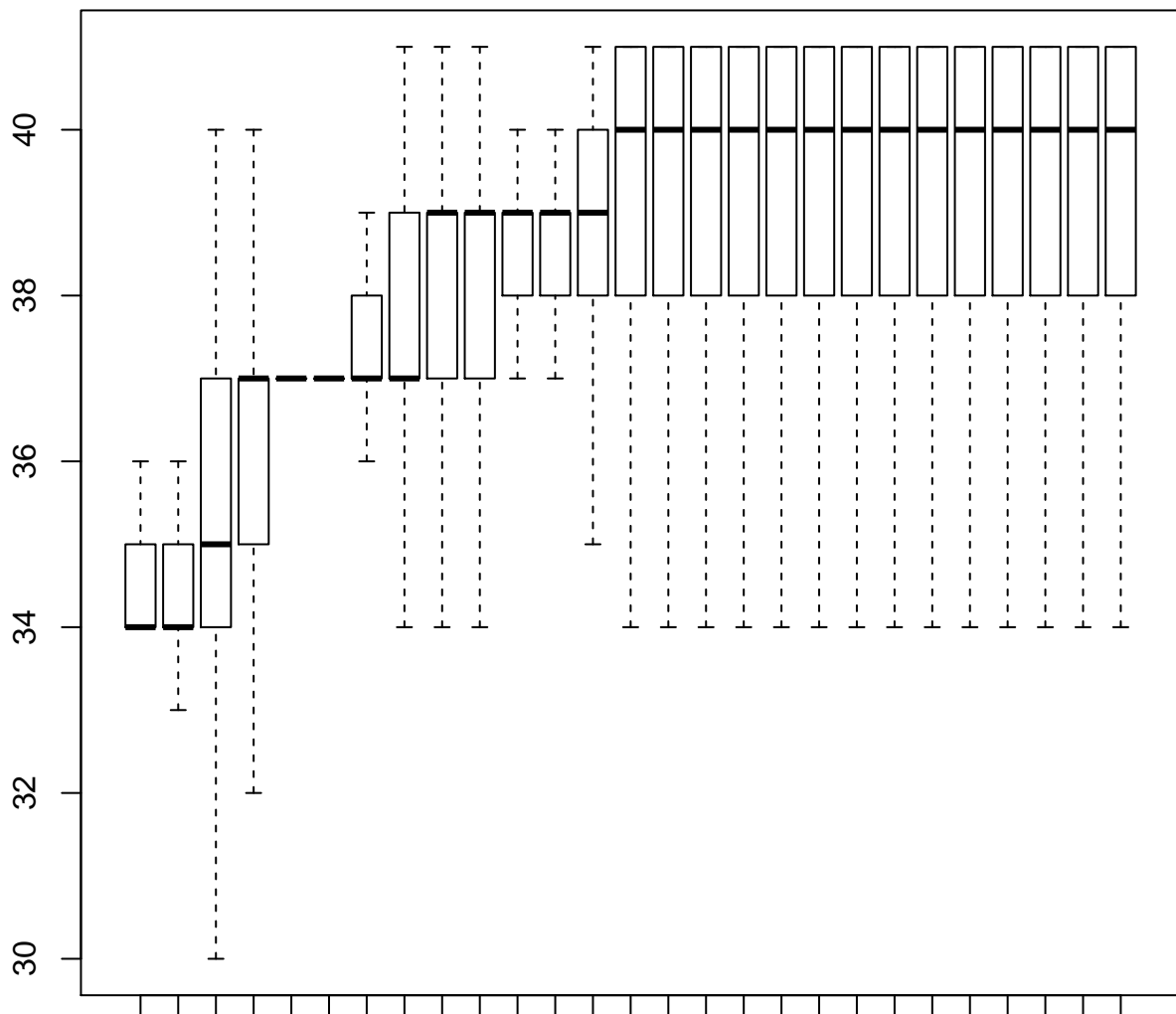

Position of Read(5' to 3')

Supplement: S1 Data — This directory contains six subdirectories. The subdirectory “correlation” contains correlation heatmaps among all samples in diencephalon and telencephalon respectively. The subdirectory “mapping_stat” contains read mapping information on genomic features for each sample. The subdirectory “MDS” shows three dimensional MDS plots of the samples. The subdirectory “ReadDuplication” contains read duplication distributions for each sample. The subdirectory “ReadQuality” contains reads quality information for each sample plotted as both boxplots and heatmaps. The subdirectory “RPKMSaturation” contains information about read depth saturation for each sample as assessed by RPKM resamplings. All transcripts were divided into four quantiles based on their expression and a relative difference of observed and real RPKM values are plotted for each sample. (ZIP) [file pgen.1006840.s015.zip › RNASeq/ReadQuality/19D_GAGATTCC-GTACTGAC_L00M_R1_001.readquality.qual.boxplot.pdf]

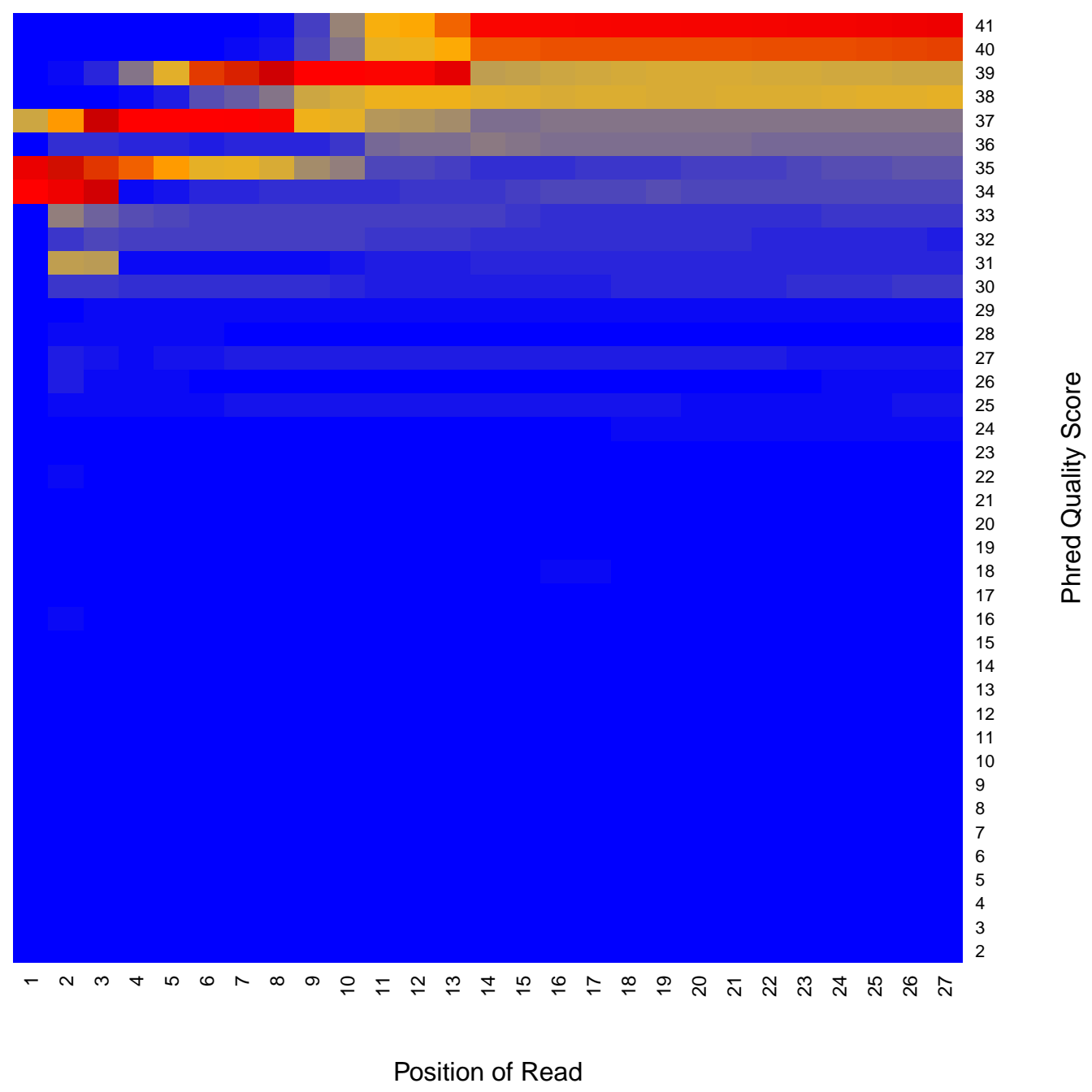

Supplement: S1 Data — This directory contains six subdirectories. The subdirectory “correlation” contains correlation heatmaps among all samples in diencephalon and telencephalon respectively. The subdirectory “mapping_stat” contains read mapping information on genomic features for each sample. The subdirectory “MDS” shows three dimensional MDS plots of the samples. The subdirectory “ReadDuplication” contains read duplication distributions for each sample. The subdirectory “ReadQuality” contains reads quality information for each sample plotted as both boxplots and heatmaps. The subdirectory “RPKMSaturation” contains information about read depth saturation for each sample as assessed by RPKM resamplings. All transcripts were divided into four quantiles based on their expression and a relative difference of observed and real RPKM values are plotted for each sample. (ZIP) [file pgen.1006840.s015.zip › RNASeq/ReadQuality/19D_GAGATTCC-GTACTGAC_L00M_R1_001.readquality.qual.heatmap.pdf]

Phred Quality Score

30 35 40

Position of Read(5'→3')

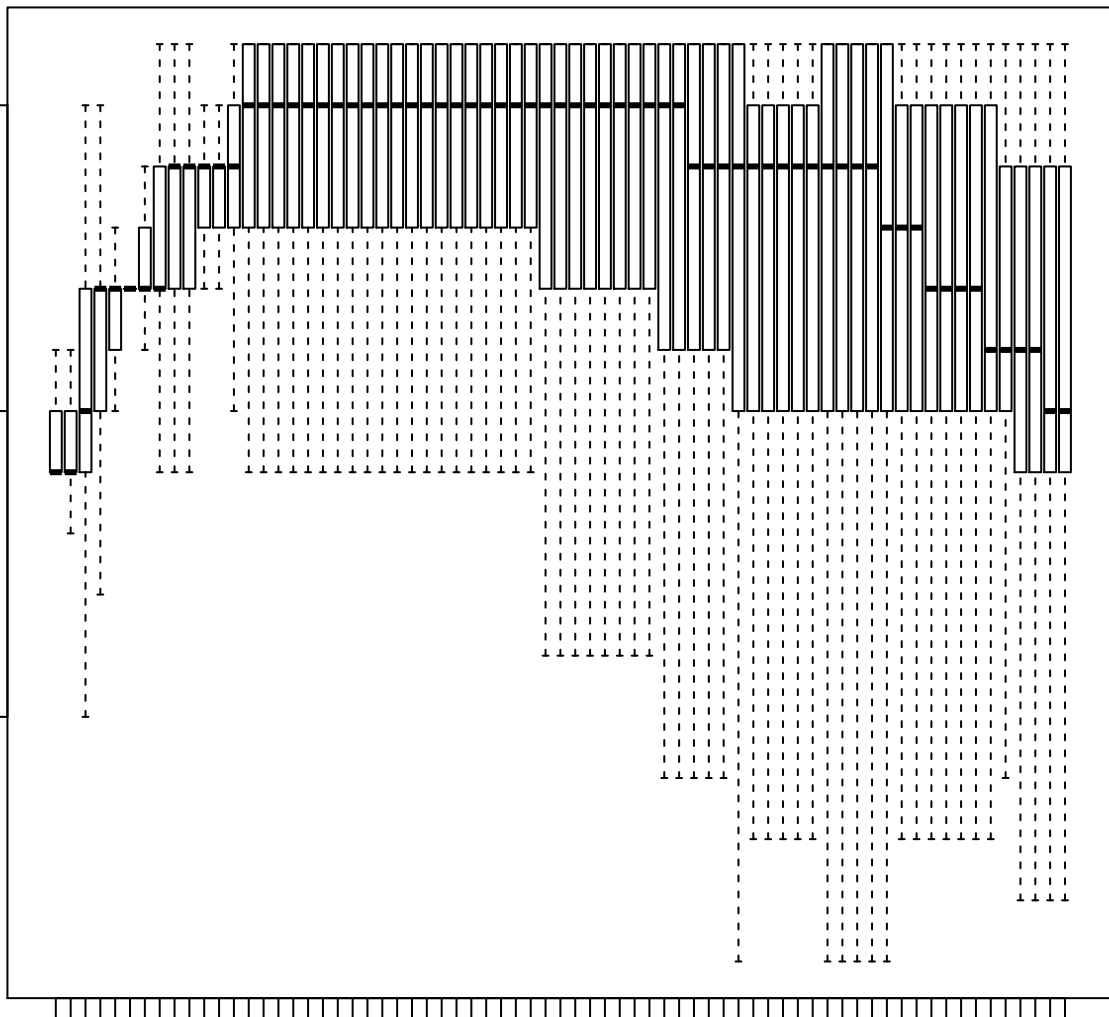

Supplement: S1 Data — This directory contains six subdirectories. The subdirectory “correlation” contains correlation heatmaps among all samples in diencephalon and telencephalon respectively. The subdirectory “mapping_stat” contains read mapping information on genomic features for each sample. The subdirectory “MDS” shows three dimensional MDS plots of the samples. The subdirectory “ReadDuplication” contains read duplication distributions for each sample. The subdirectory “ReadQuality” contains reads quality information for each sample plotted as both boxplots and heatmaps. The subdirectory “RPKMSaturation” contains information about read depth saturation for each sample as assessed by RPKM resamplings. All transcripts were divided into four quantiles based on their expression and a relative difference of observed and real RPKM values are plotted for each sample. (ZIP) [file pgen.1006840.s015.zip › RNASeq/ReadQuality/19T_GAGATTCC-CAGGACGT_L00M_R1_001.readquality.qual.boxplot.pdf]

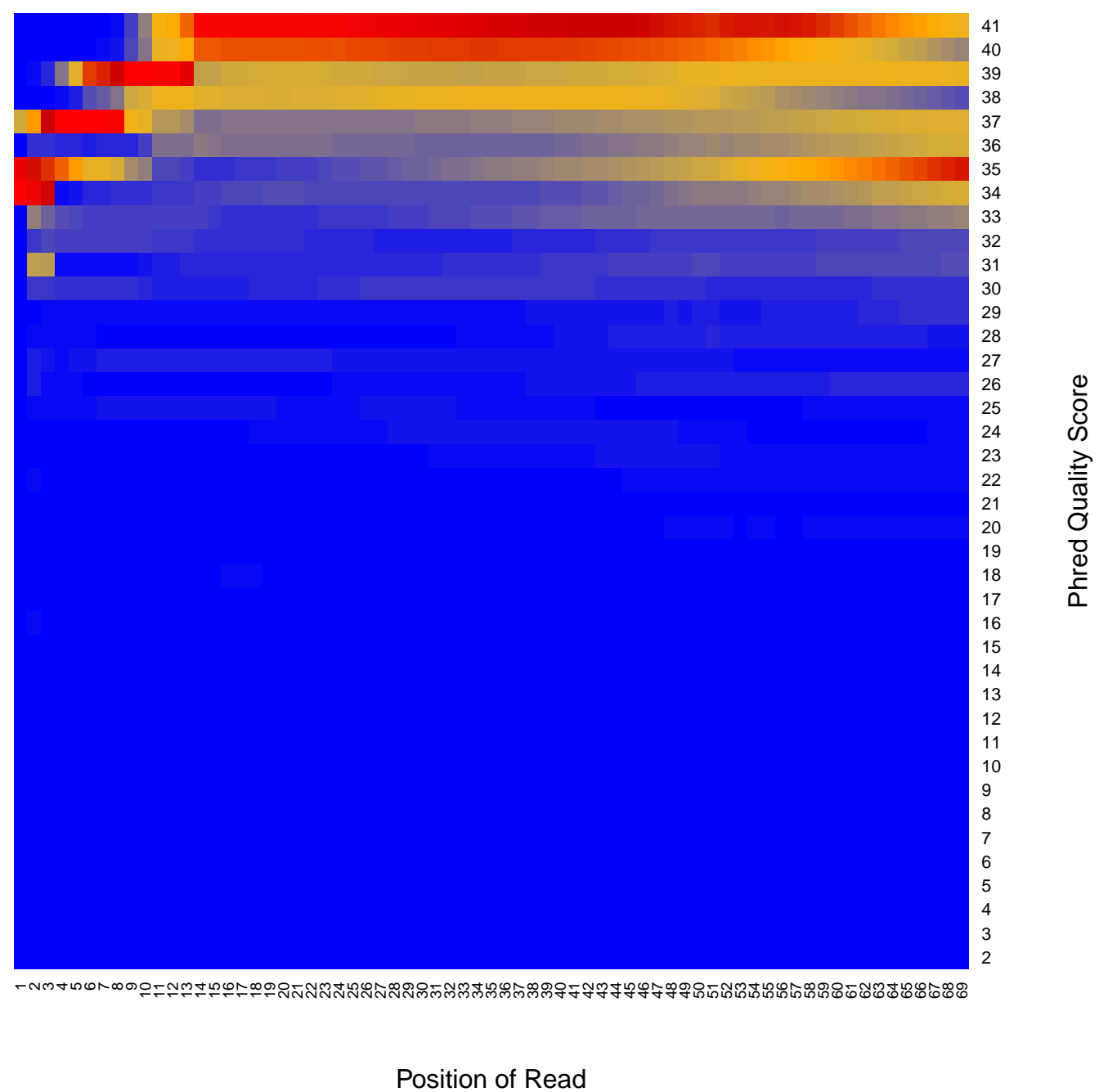

Supplement: S1 Data — This directory contains six subdirectories. The subdirectory “correlation” contains correlation heatmaps among all samples in diencephalon and telencephalon respectively. The subdirectory “mapping_stat” contains read mapping information on genomic features for each sample. The subdirectory “MDS” shows three dimensional MDS plots of the samples. The subdirectory “ReadDuplication” contains read duplication distributions for each sample. The subdirectory “ReadQuality” contains reads quality information for each sample plotted as both boxplots and heatmaps. The subdirectory “RPKMSaturation” contains information about read depth saturation for each sample as assessed by RPKM resamplings. All transcripts were divided into four quantiles based on their expression and a relative difference of observed and real RPKM values are plotted for each sample. (ZIP) [file pgen.1006840.s015.zip › RNASeq/ReadQuality/19T_GAGATTCC-CAGGACGT_L00M_R1_001.readquality.qual.heatmap.pdf]

Phred Quality Score

30  
35  
40

Position of Read(5'→3')

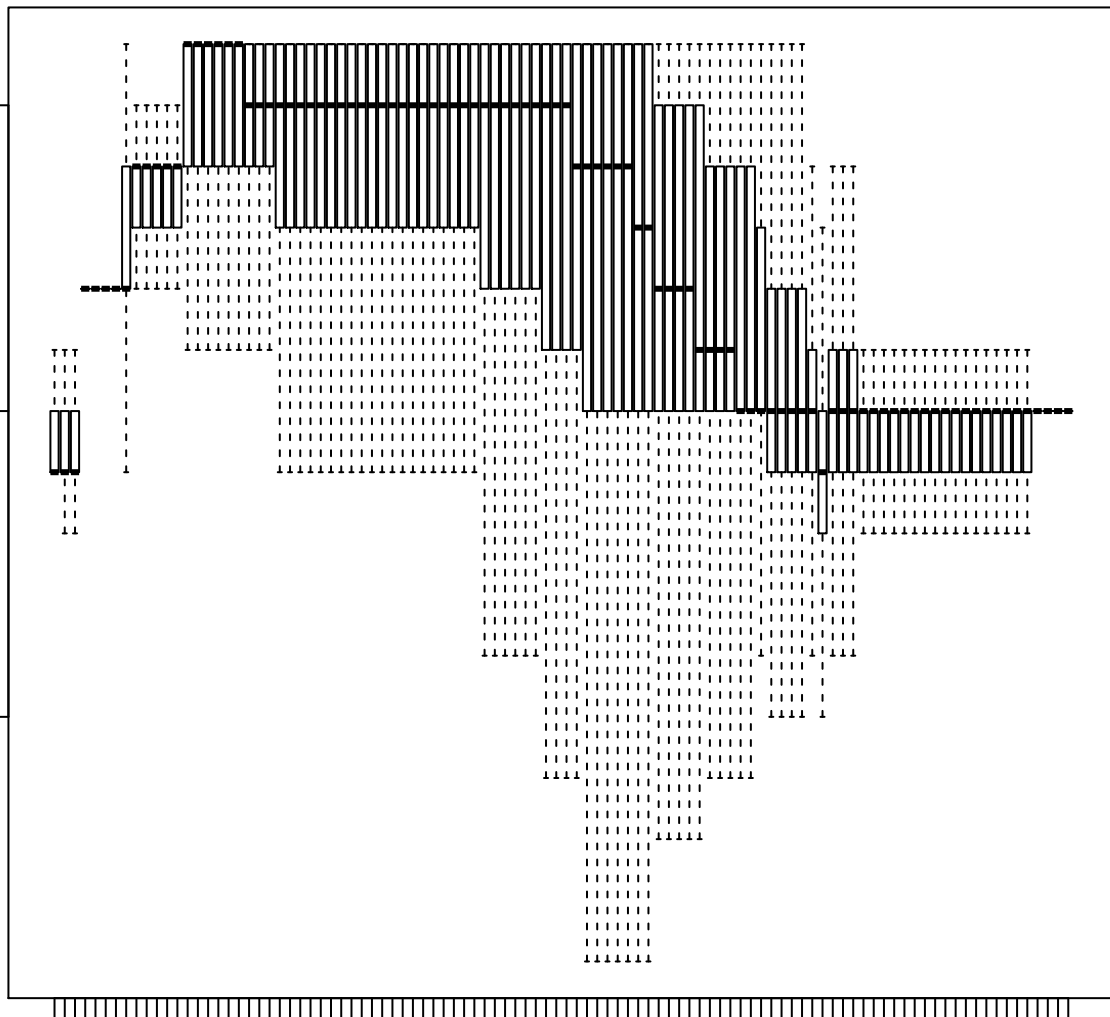

Supplement: S1 Data — This directory contains six subdirectories. The subdirectory “correlation” contains correlation heatmaps among all samples in diencephalon and telencephalon respectively. The subdirectory “mapping_stat” contains read mapping information on genomic features for each sample. The subdirectory “MDS” shows three dimensional MDS plots of the samples. The subdirectory “ReadDuplication” contains read duplication distributions for each sample. The subdirectory “ReadQuality” contains reads quality information for each sample plotted as both boxplots and heatmaps. The subdirectory “RPKMSaturation” contains information about read depth saturation for each sample as assessed by RPKM resamplings. All transcripts were divided into four quantiles based on their expression and a relative difference of observed and real RPKM values are plotted for each sample. (ZIP) [file pgen.1006840.s015.zip › RNASeq/ReadQuality/1D_ATTACTCG-ATAGAGGC_L00M_R1_001.readquality.qual.boxplot.pdf]

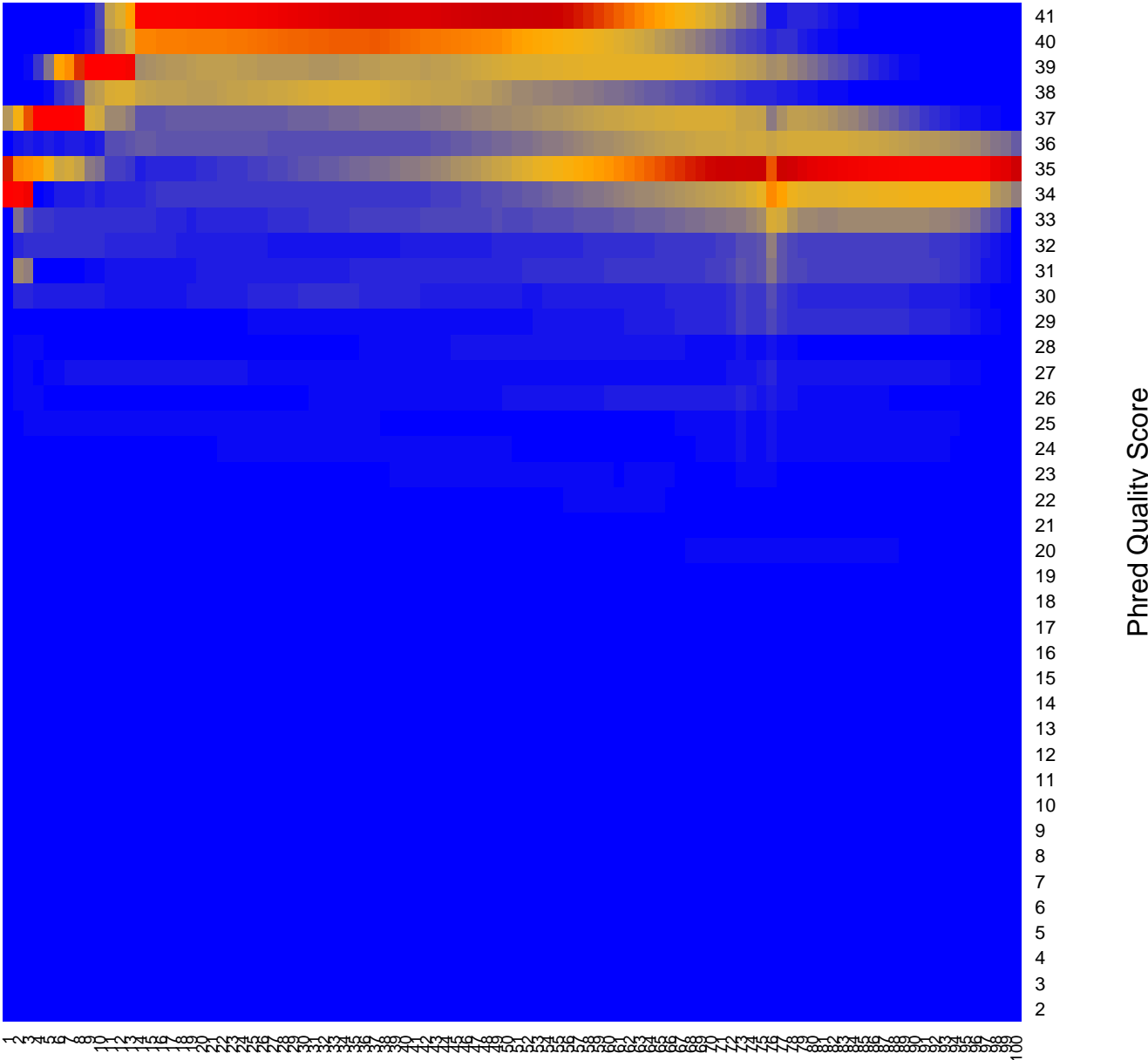

Position of Read

Supplement: S1 Data — This directory contains six subdirectories. The subdirectory “correlation” contains correlation heatmaps among all samples in diencephalon and telencephalon respectively. The subdirectory “mapping_stat” contains read mapping information on genomic features for each sample. The subdirectory “MDS” shows three dimensional MDS plots of the samples. The subdirectory “ReadDuplication” contains read duplication distributions for each sample. The subdirectory “ReadQuality” contains reads quality information for each sample plotted as both boxplots and heatmaps. The subdirectory “RPKMSaturation” contains information about read depth saturation for each sample as assessed by RPKM resamplings. All transcripts were divided into four quantiles based on their expression and a relative difference of observed and real RPKM values are plotted for each sample. (ZIP) [file pgen.1006840.s015.zip › RNASeq/ReadQuality/1D_ATTACTCG-ATAGAGGC_L00M_R1_001.readquality.qual.heatmap.pdf]

Phred Quality Score

40  
35  
30

Position of Read(5'→3')

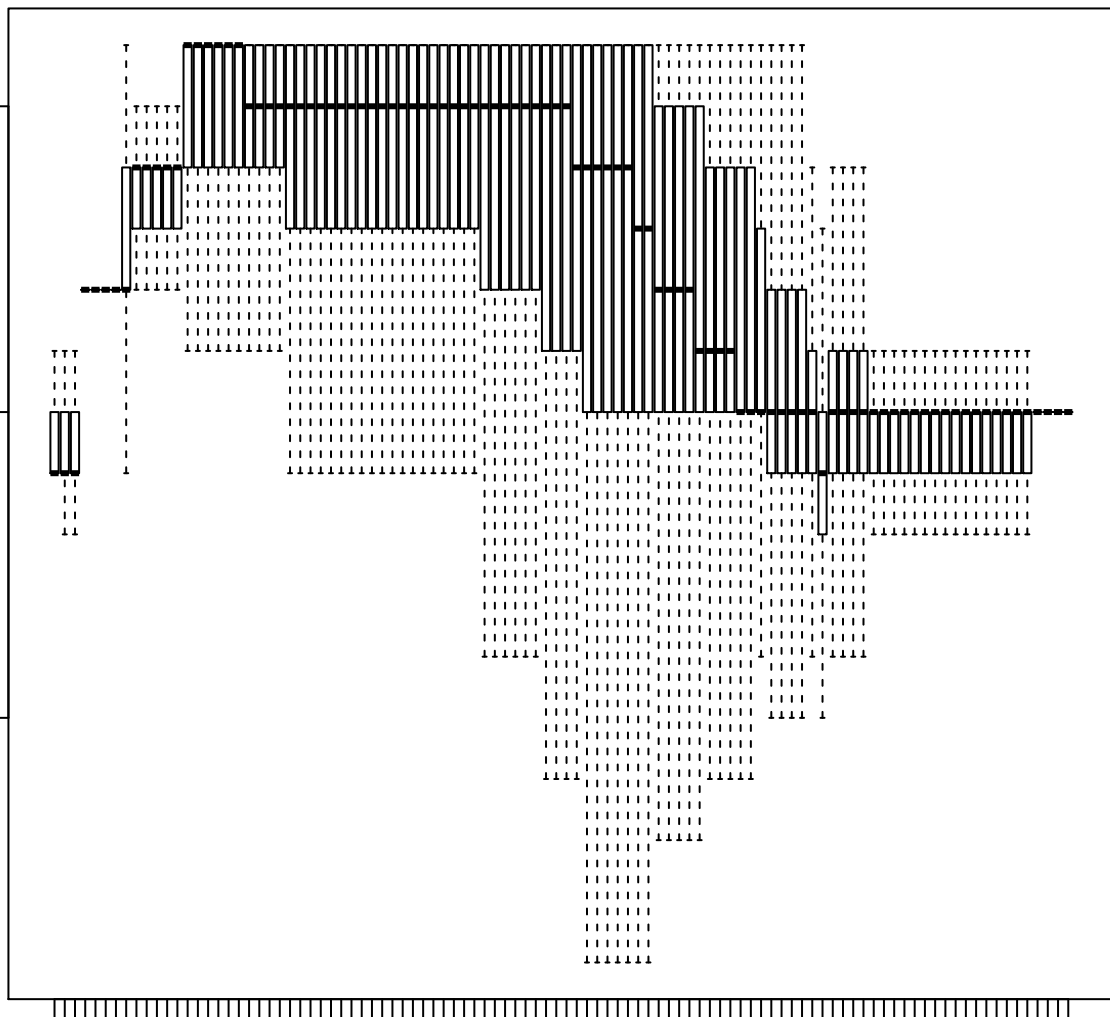

Supplement: S1 Data — This directory contains six subdirectories. The subdirectory “correlation” contains correlation heatmaps among all samples in diencephalon and telencephalon respectively. The subdirectory “mapping_stat” contains read mapping information on genomic features for each sample. The subdirectory “MDS” shows three dimensional MDS plots of the samples. The subdirectory “ReadDuplication” contains read duplication distributions for each sample. The subdirectory “ReadQuality” contains reads quality information for each sample plotted as both boxplots and heatmaps. The subdirectory “RPKMSaturation” contains information about read depth saturation for each sample as assessed by RPKM resamplings. All transcripts were divided into four quantiles based on their expression and a relative difference of observed and real RPKM values are plotted for each sample. (ZIP) [file pgen.1006840.s015.zip › RNASeq/ReadQuality/1T_ATTACTCG-TATAGCCT_L00M_R1_001.readquality.qual.boxplot.pdf]

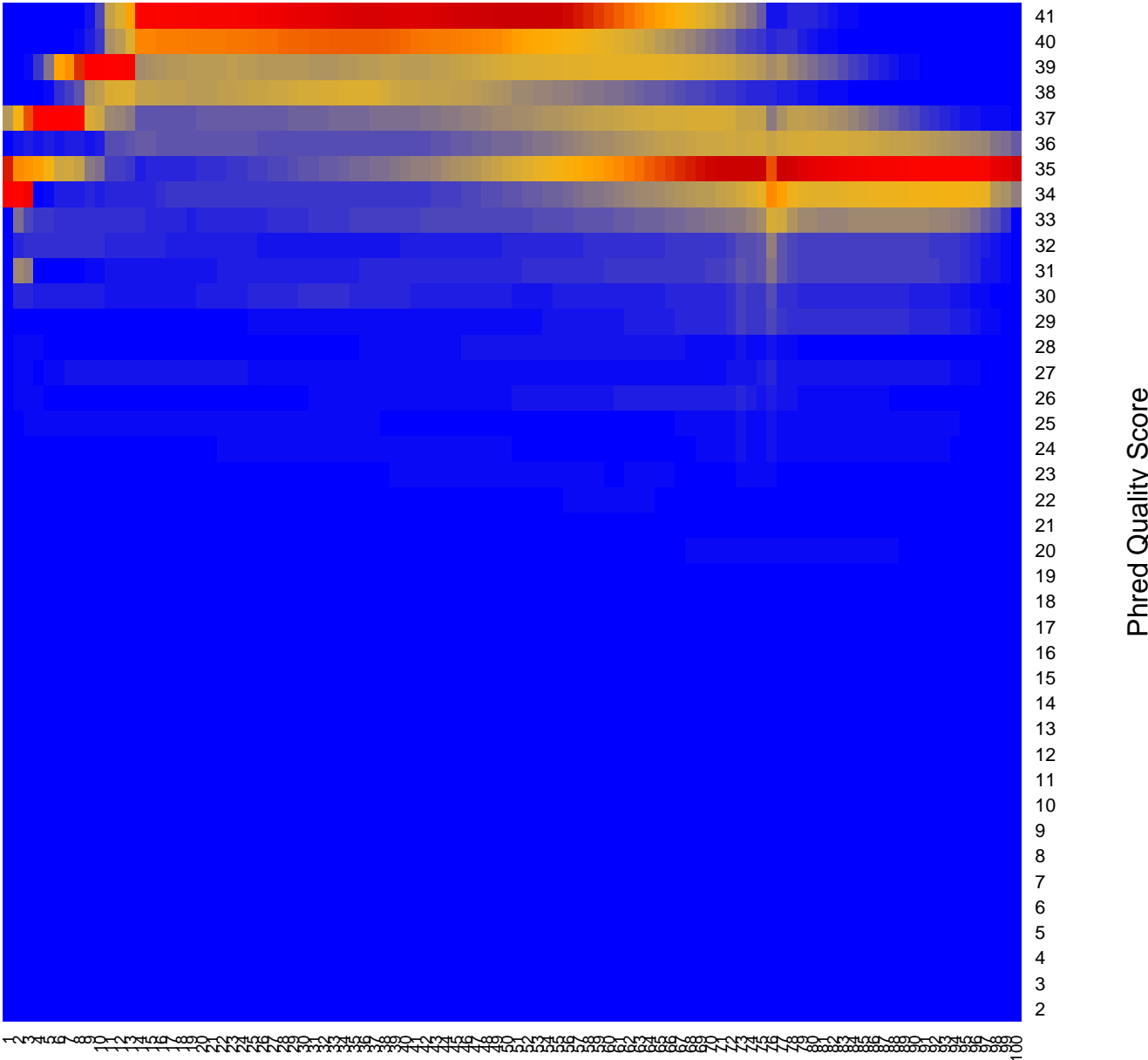

Position of Read

Supplement: S1 Data — This directory contains six subdirectories. The subdirectory “correlation” contains correlation heatmaps among all samples in diencephalon and telencephalon respectively. The subdirectory “mapping_stat” contains read mapping information on genomic features for each sample. The subdirectory “MDS” shows three dimensional MDS plots of the samples. The subdirectory “ReadDuplication” contains read duplication distributions for each sample. The subdirectory “ReadQuality” contains reads quality information for each sample plotted as both boxplots and heatmaps. The subdirectory “RPKMSaturation” contains information about read depth saturation for each sample as assessed by RPKM resamplings. All transcripts were divided into four quantiles based on their expression and a relative difference of observed and real RPKM values are plotted for each sample. (ZIP) [file pgen.1006840.s015.zip › RNASeq/ReadQuality/1T_ATTACTCG-TATAGCCT_L00M_R1_001.readquality.qual.heatmap.pdf]

Phred Quality Score

30  
35  
40

Position of Read(5'→3')

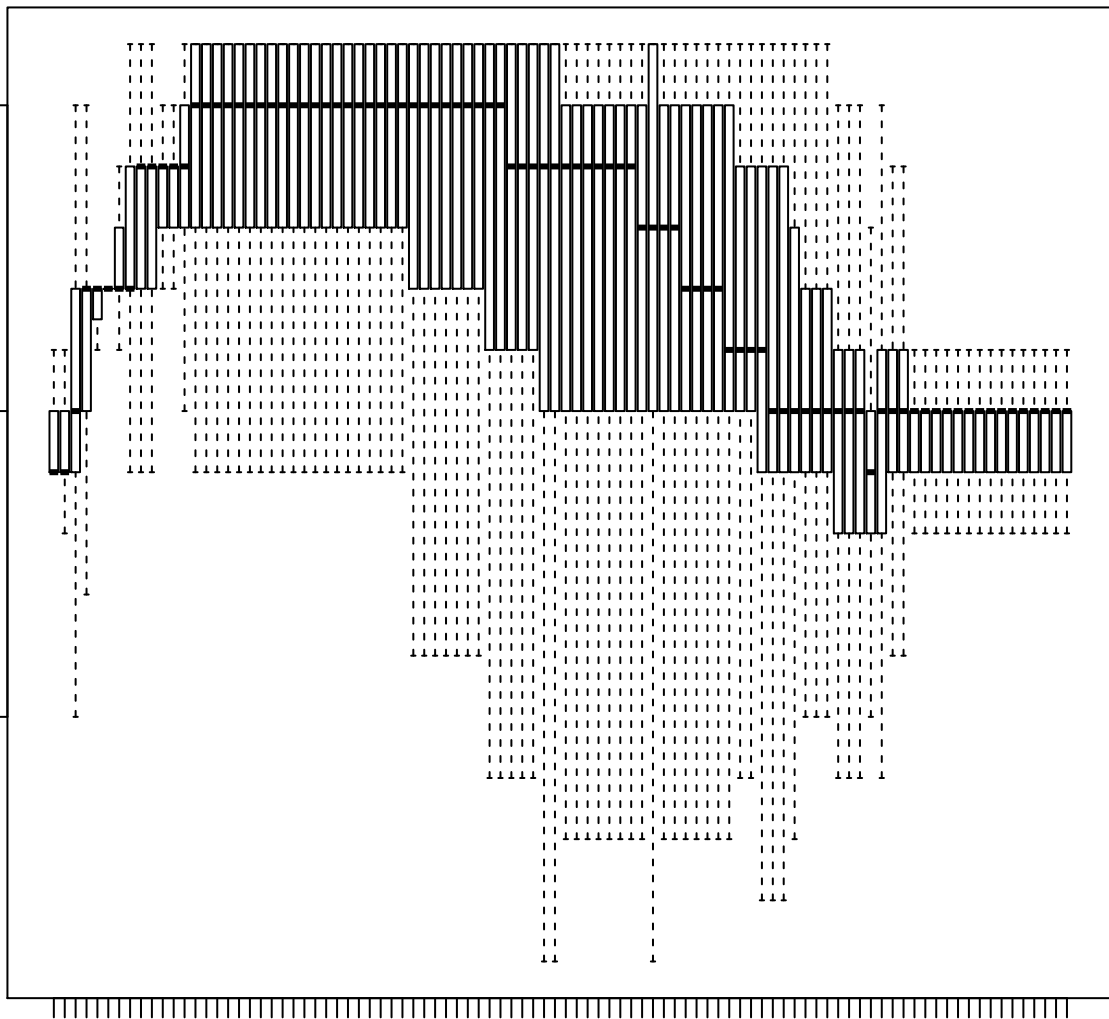

Supplement: S1 Data — This directory contains six subdirectories. The subdirectory “correlation” contains correlation heatmaps among all samples in diencephalon and telencephalon respectively. The subdirectory “mapping_stat” contains read mapping information on genomic features for each sample. The subdirectory “MDS” shows three dimensional MDS plots of the samples. The subdirectory “ReadDuplication” contains read duplication distributions for each sample. The subdirectory “ReadQuality” contains reads quality information for each sample plotted as both boxplots and heatmaps. The subdirectory “RPKMSaturation” contains information about read depth saturation for each sample as assessed by RPKM resamplings. All transcripts were divided into four quantiles based on their expression and a relative difference of observed and real RPKM values are plotted for each sample. (ZIP) [file pgen.1006840.s015.zip › RNASeq/ReadQuality/20D_ATTCAGAA-ATAGAGGC_L00M_R1_001.readquality.qual.boxplot.pdf]

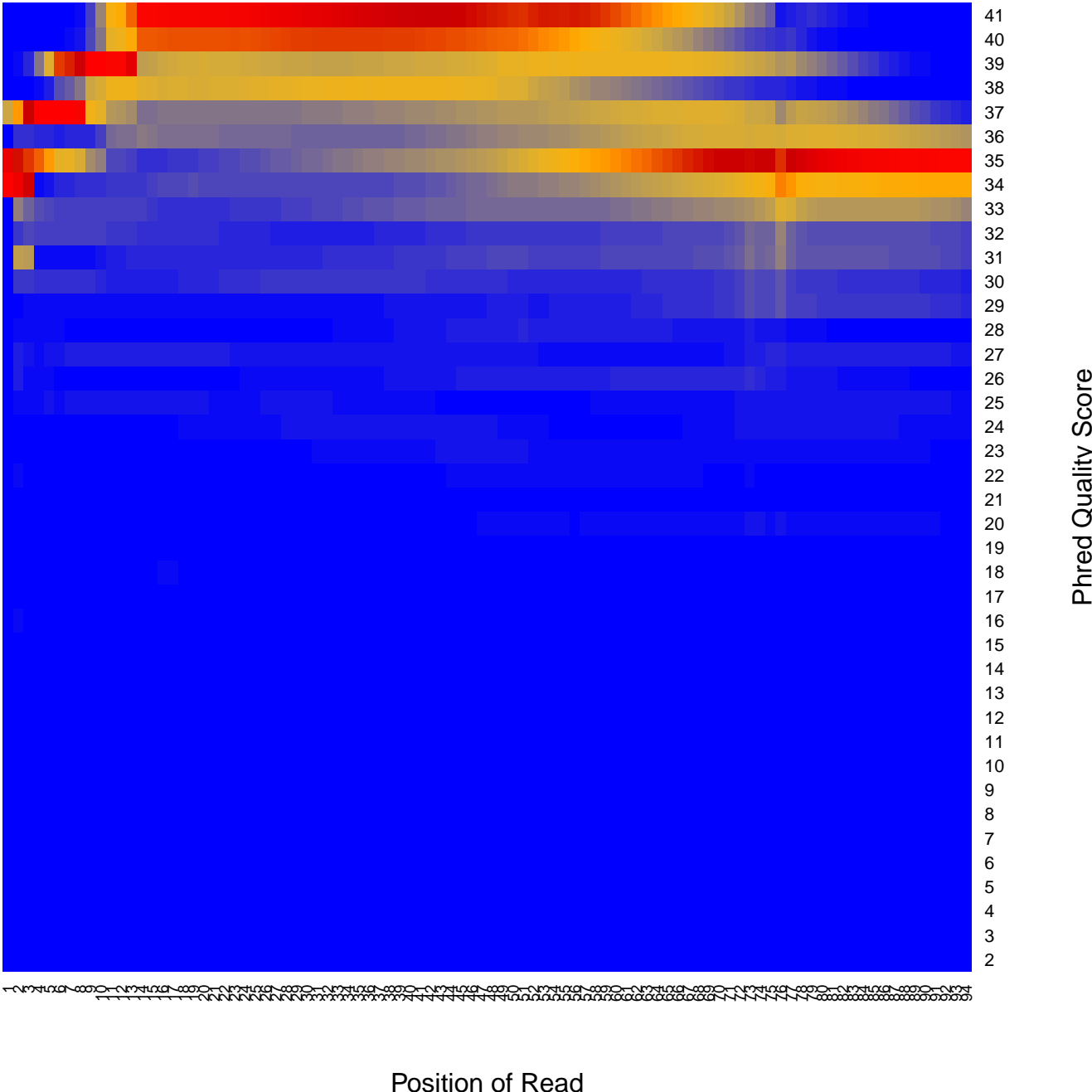

Supplement: S1 Data — This directory contains six subdirectories. The subdirectory “correlation” contains correlation heatmaps among all samples in diencephalon and telencephalon respectively. The subdirectory “mapping_stat” contains read mapping information on genomic features for each sample. The subdirectory “MDS” shows three dimensional MDS plots of the samples. The subdirectory “ReadDuplication” contains read duplication distributions for each sample. The subdirectory “ReadQuality” contains reads quality information for each sample plotted as both boxplots and heatmaps. The subdirectory “RPKMSaturation” contains information about read depth saturation for each sample as assessed by RPKM resamplings. All transcripts were divided into four quantiles based on their expression and a relative difference of observed and real RPKM values are plotted for each sample. (ZIP) [file pgen.1006840.s015.zip › RNASeq/ReadQuality/20D_ATTCAGAA-ATAGAGGC_L00M_R1_001.readquality.qual.heatmap.pdf]
