# Supplementary material for: Temporal dynamics of neurogenomic plasticity in response to social interactions in male threespined sticklebacks
Source: PLoS Genet. 2017 Jul 13;13(7):e1006840. doi: 10.1371/journal.pgen.1006840 (PMC5509087; doi:10.1371/journal.pgen.1006840)
Supplement: S10 Table — These TFs are in the TRN and are enriched in the DAPDEGx with accessibility indicated. Some of the TFs (in bold) were differentially expressed and in a cluster. The general expression pattern of their cluster is indicated. A subset of this table is in Table 1. (DOCX) [file pgen.1006840.s012.docx]

Table S10. Enrichment of TFs in DAPDEG_x_.

| *TFs* | Description | Significance |
| --- | --- | --- |
| *pparg* | peroxisome proliferator activated receptor gamma | P < 0.005 |
| *neurod* | neurogenic differentiation | P < 0.0001 |
| *ikzf1* | IKAROS family zinc finger 1 (Ikaros) | P < 0.0001 |
| *Irf4b* | interferon regulatory factor 4b | P < 0.005 |
| *spi1a* | spleen focus forming virus (SFFV) proviral integration oncogene spi1a | P < 0.0001 |
| *ETV7* | ets variant 7 | P < 0.001 |
| *pbx4* | pre-B-cell leukemia transcription factor 4 | P < 0.05 |
| *mafbb* | v-maf musculoaponeurotic fibrosarcoma oncogene family, protein B, duplicate b | P < 0.01 |
| *cebpb* | CCAAT/enhancer binding protein (C/EBP), beta | P < 0.0001 |
| *homez* | homeodomain leucine zipper gene | P < 0.05 |
| *spi1b* | spleen focus forming virus (SFFV) proviral integration oncogene spi1b | P < 0.0001 |
| *irf8* | interferon regulatory factor 8 | P < 0.0001 |
| *NFATC3* | nuclear factor of activated T-cells, cytoplasmic, calcineurin-dependent 3 | P < 0.05 |
| *TLX2* | T-cell leukemia homeobox 2 | P < 0.05 |
| *tfec* | transcription factor EC | P < 0.0001 |
| *LYL1* | lymphoblastic leukemia associated hematopoiesis regulator 1 | P< 0.05 |
