# Supplementary material for: Temporal dynamics of neurogenomic plasticity in response to social interactions in male threespined sticklebacks
Source: PLoS Genet. 2017 Jul 13;13(7):e1006840. doi: 10.1371/journal.pgen.1006840 (PMC5509087; doi:10.1371/journal.pgen.1006840)
Supplement: S1 Data — This directory contains six subdirectories. The subdirectory “correlation” contains correlation heatmaps among all samples in diencephalon and telencephalon respectively. The subdirectory “mapping_stat” contains read mapping information on genomic features for each sample. The subdirectory “MDS” shows three dimensional MDS plots of the samples. The subdirectory “ReadDuplication” contains read duplication distributions for each sample. The subdirectory “ReadQuality” contains reads quality information for each sample plotted as both boxplots and heatmaps. The subdirectory “RPKMSaturation” contains information about read depth saturation for each sample as assessed by RPKM resamplings. All transcripts were divided into four quantiles based on their expression and a relative difference of observed and real RPKM values are plotted for each sample. (ZIP) [file pgen.1006840.s015.zip › RNASeq/RPKMSaturation/4D_ATTACTCG-GTACTGAC_L00M_R1_001.pdfpkmsaturation.saturation.pdf]

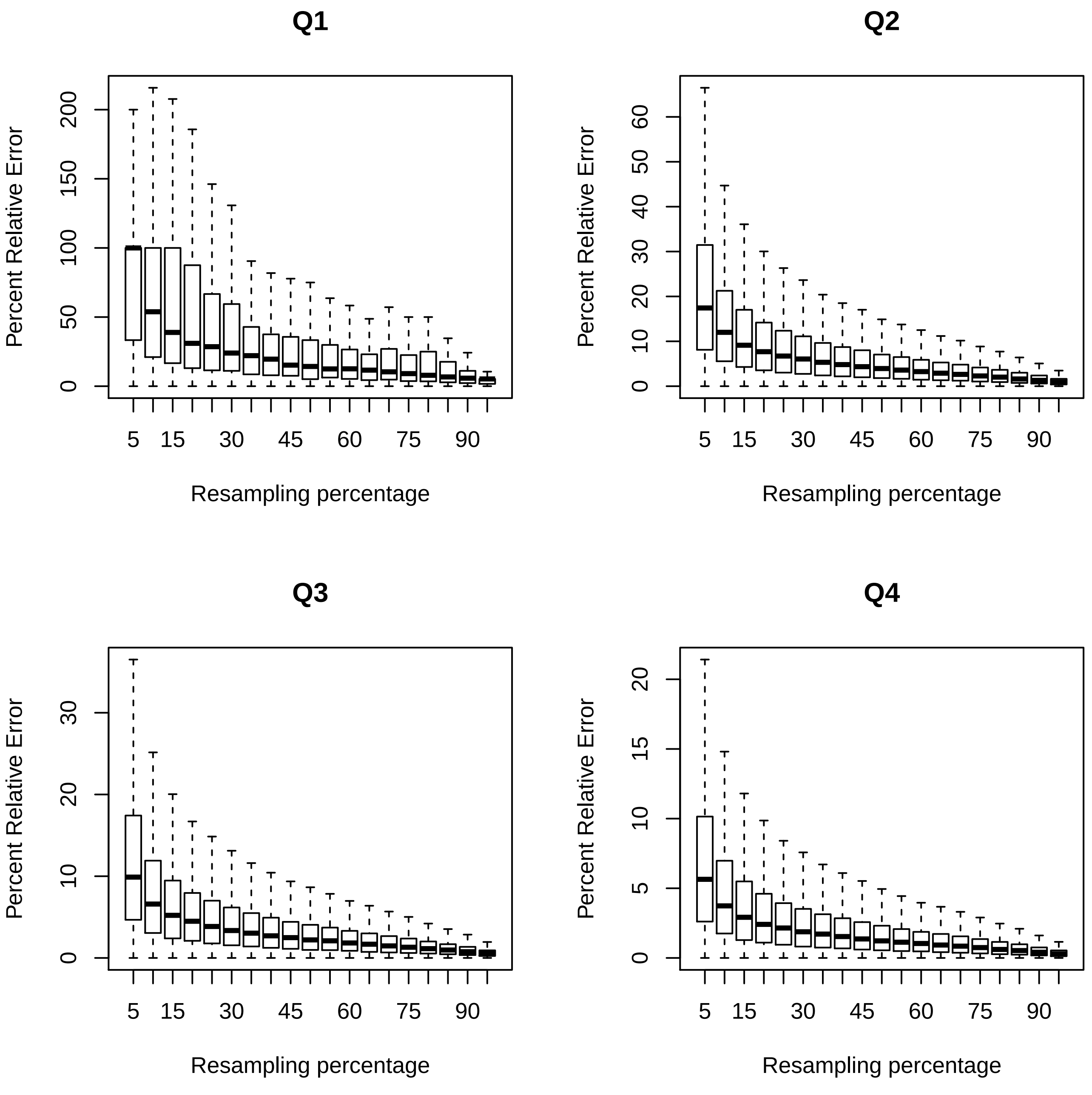
Q1
Percent Relative Error
0
50
100
150
200
5
15
30
45
60
75
90
Resampling percentage
Q2
Percent Relative Error
0
10
20
30
40
50
60
5
15
30
45
60
75
90
Resampling percentage
Q3
Percent Relative Error
0
10
20
30
5
15
30
45
60
75
90
Resampling percentage
Q4
Percent Relative Error
0
5
10
15
20
5
15
30
45
60
75
90
Resampling percentage
